# Correction: Global burden and future projections of non-communicable diseases (2000–2050): Progress toward SDG 3.4 and disparities across regions and risk factors

**DOI:** 10.1371/journal.pone.0358109

**Published:** 2026-09-09

**Authors:** The PLOS One Staff

## Notice of Republication

This article was republished August 28, 2026 to correct the second author’s first name to David.

## Supporting information

S1 File Originally published, uncorrected article. (PDF)

S2 File Republished, corrected article. (PDF)
