# Supplementary material for: Global burden and future projections of non-communicable diseases (2000–2050): Progress toward SDG 3.4 and disparities across regions and risk factors
Source: PLoS One. 2025 Dec 10;20(12):e0336036. doi: 10.1371/journal.pone.0336036 (PMC12694828; doi:10.1371/journal.pone.0336036)
Supplement: S1 File — (PDF) [file pone.0336036.s001.pdf]

Supplementary materials

Table of Contents

Supplementary tables: 3

S0: Distribution of non-communicable disease by level 3

S1: Global burden of NCDs 4

S1A: Global burden of NCDs 2021 4

S1B: Global Burden of NCDs by SDI 2021 10

S1C: Global Burden of NCDs by WHO regions 2021 12

S2: Countries burden 16

S2A: Countries CVDs incidence, mortality and DALYS: 16

S2B: Country Neoplasm incidence, mortality and DALYS 41

S2C: Country CRDs incidence, mortality and DLAYs 75

S2D: Country based Diabetes maleates incidence, mortality and DALYs 117

S3: Risk factors 151

S3A: Metabolic risk factors mortality and DALYS by WHO regions 153

S3B: Metabolic risk factors mortality and DALYS by SDI categories 154

S3C: Behavioural risk factors by WHO regions. 154

S3D: Behavioural risk factors by SDI category 155

S3E: Environmental (Air pollution) risk factor by WHO region 156

S3F: Environmental (Air pollution) risk factor by SDI category 156

S4: Gender analysis 157

S4A: Gender analysis by WHO region 158

S4B: Gender analysis by SDI category 159

S5: Global Burden of NCDs trends 2000 – 2021 160

S6: 2050 projections 160

S6A: country-based burden of CVDs mortality projection 2050 169

S6B: country-based burden of Neoplasm mortality projection 2050 186

S6C: country-based burden of chronic respiratory diseases mortality projection 2050... 203

S5D: country-based burden of diabetes maleates mortality projection 2050 220

Supplementary figures 238

S1 Fig: Metabolic Risk factors Deaths by WHO regions 238

S2 Fig: Metabolic Risk factors DALYs by WHO regions 238

S3 Fig: Metabolic Risk factors Mortality by SDI category 239

S4 Fig: Metabolic Risk factors DALYs by SDI category 239

S5 Fig: Behavioural Risk factors Mortality by WHO regions 240

S6 Fig. Behavioural risk factors DALYs by WHO regions 240

S7 Fig. Behavioural risk factors Mortality by SDI category 241

S8 Fig. behavioural risk factors DALYs by SDI category 241

S9 Fig. Environmental risk factor Mortality by WHO regions 242

S10 Fig. Environmental risk factor DALYs by WHO regions 242

S11 Fig. Environmental risk factor Mortality by SDI category 243

S12 Fig. Environmental risk factor DALYs by SDI category 243

S13 Fig. Gender burden analysis by WHO regions 244

S14 Fig. Gender burden analysis by SDI category 244

Supplementary tables:

S0: Distribution of non-communicable disease by level

**Supplementary 0. Distribution of non-communicable disease by level**		
**Level 1 Causes**	**Level 2 Causes**	**Level 3 Causes**
**Non-communicable diseases**	**Neoplasms**	Lip and oral cavity cancer; Nasopharynx cancer; Other pharynx cancer; Esophageal cancer; Stomach cancer; Colon and rectum cancer; Liver cancer; Gallbladder and biliary tract cancer; Pancreatic cancer; Larynx cancer; Tracheal, bronchus, and lung cancer; Malignant skin melanoma; Non-melanoma skin cancer; Soft tissue and other extraosseous sarcomas; Malignant neoplasm of bone and articular cartilage; Breast cancer; Cervical cancer; Uterine cancer; Ovarian cancer; Prostate cancer; Testicular cancer; Kidney cancer; Bladder cancer; Brain and central nervous system cancer; Eye cancer; Neuroblastoma and other peripheral nervous cell tumors; Thyroid cancer; Mesothelioma; Hodgkin lymphoma; Non-Hodgkin lymphoma; Multiple myeloma; Leukemia; Other malignant neoplasms; Other neoplasms
	**Cardiovascular diseases**	Rheumatic heart disease; ischemic heart disease; Stroke; Hypertensive heart disease; Non-rheumatic valvular heart disease; Cardiomyopathy and myocarditis; Pulmonary Arterial Hypertension; Atrial fibrillation and flutter; Aortic aneurysm; Lower extremity peripheral arterial disease; Endocarditis; Other cardiovascular and circulatory diseases
	**Chronic respiratory diseases**	Chronic obstructive pulmonary disease (COPD); Pneumoconiosis; Asthma; Interstitial lung disease and pulmonary sarcoidosis; Other chronic respiratory diseases
	**Digestive diseases**	Cirrhosis and other chronic liver diseases; Upper digestive system diseases; Appendicitis; Paralytic ileus and intestinal obstruction; Inguinal, femoral, and abdominal hernia; Inflammatory bowel disease; Vascular intestinal disorders; Gallbladder and biliary diseases; Pancreatitis; Other digestive diseases
	**Neurological disorders**	Alzheimer’s disease and other dementias; Parkinson’s disease; Idiopathic epilepsy; Multiple

		sclerosis; Motor neuron disease; Headache disorders; Other neurological disorders
	**Mental disorders**	Schizophrenia; Depressive disorders; Bipolar disorder; anxiety disorders; Eating disorders; Autism spectrum disorders; Attention-deficit/hyperactivity disorder (ADHD); Conduct disorder; Idiopathic developmental intellectual disability; Other mental disorders
	**Substance use disorders**	Alcohol use disorders; Drug use disorders
	**Diabetes and kidney diseases**	Diabetes mellitus; Chronic kidney disease; Acute glomerulonephritis
	**Skin and subcutaneous diseases**	Dermatitis; Psoriasis; Bacterial skin diseases; Scabies; Fungal skin diseases; Viral skin diseases; Acne vulgaris; Alopecia areata; Pruritus; Urticaria; Decubitus ulcer; Other skin and subcutaneous diseases
	**Sense organ diseases**	Blindness and vision loss; Age-related and other hearing loss; Other sense organ diseases
	**Musculoskeletal disorders**	Rheumatoid arthritis; Osteoarthritis; Low back pain; Neck pain; Gout; Other musculoskeletal disorders
	**Other non-communicable diseases**	Congenital birth defects; Urinary diseases and male infertility; Gynecological diseases; Hemoglobinopathies and hemolytic anemias; Endocrine, metabolic, blood, and immune disorders; Oral disorders; Sudden infant death syndrome

S1: Global burden of NCDs

S1A: Global burden of NCDs 2021

measure_name	location_name	cause_id	cause_name	metric_name	year	val
**Incidence (total and breakdown by cause)**						
**All NCD's Incidence**						
Incidence	Global	409	Non-communicable diseases	Number	2021	12364222888

Incidence	Global	409	Non-communicable diseases	Percent	2021	0.349479295
Incidence	Global	409	Non-communicable diseases	Rate	2021	156680.6404
**Breakdown**						
Incidence	Global	973	Substance use disorders	Number	2021	69389099.74
Incidence	Global	973	Substance use disorders	Percent	2021	0.001885325
Incidence	Global	973	Substance use disorders	Rate	2021	879.3054511
Incidence	Global	542	Neurological disorders	Number	2021	823798509.6
Incidence	Global	542	Neurological disorders	Percent	2021	0.022384491
Incidence	Global	542	Neurological disorders	Rate	2021	10439.2552
Incidence	Global	558	Mental disorders	Number	2021	444397716.1
Incidence	Global	558	Mental disorders	Percent	2021	0.012075333
Incidence	Global	558	Mental disorders	Rate	2021	5631.451276
Incidence	Global	640	Other non-communicable diseases	Number	2021	5201596497
Incidence	Global	640	Other non-communicable diseases	Percent	2021	0.14129717
Incidence	Global	640	Other non-communicable diseases	Rate	2021	65915.13899
Incidence	Global	653	Skin and subcutaneous diseases	Number	2021	4693912471
Incidence	Global	653	Skin and subcutaneous diseases	Percent	2021	0.127539386
Incidence	Global	653	Skin and subcutaneous diseases	Rate	2021	59481.71742
Incidence	Global	669	Sense organ diseases	Number	2021	214542.8349
Incidence	Global	669	Sense organ diseases	Percent	2021	5.83E-06
Incidence	Global	669	Sense organ diseases	Rate	2021	2.718707765
Incidence	Global	491	Cardiovascular diseases	Number	2021	66809836.88

Incidence	Global	491	Cardiovascular diseases	Percent	2021	0.001815333
Incidence	Global	491	Cardiovascular diseases	Rate	2021	846.6207802
Incidence	Global	508	Chronic respiratory diseases	Number	2021	55212753.79
Incidence	Global	508	Chronic respiratory diseases	Percent	2021	0.001500388
Incidence	Global	508	Chronic respiratory diseases	Rate	2021	699.6614103
Incidence	Global	974	Diabetes and kidney diseases	Number	2021	44905585.96
Incidence	Global	974	Diabetes and kidney diseases	Percent	2021	0.001220236
Incidence	Global	974	Diabetes and kidney diseases	Rate	2021	569.0479726
Incidence	Global	526	Digestive diseases	Number	2021	530312837.1
Incidence	Global	626	Musculoskeletal disorders	Number	2021	367193430.9
Incidence	Global	626	Musculoskeletal disorders	Percent	2021	0.009978137
Incidence	Global	626	Musculoskeletal disorders	Rate	2021	4653.111031
Incidence	Global	410	Neoplasms	Number	2021	66479607.27
Incidence	Global	410	Neoplasms	Percent	2021	0.001806549
Incidence	Global	410	Neoplasms	Rate	2021	842.4360782
Incidence	Global	526	Digestive diseases	Percent	2021	0.014989487
Incidence	Global	526	Digestive diseases	Rate	2021	6720.176083
**Mortality (total and breakdown by cause)**						
**<u>All NCD's mortality</u>**						
Deaths	Global	409	Non-communicable diseases	Number	2021	43768181.657328695
Deaths	Global	409	Non-communicable diseases	Percent	2021	0.6448684913961369
Deaths	Global	409	Non-communicable diseases	Rate	2021	554.6346740454488
**<u>NCD's mortality breakdown</u>**						
Deaths	Global	410	Neoplasms	Number	2021	9888413.461
Deaths	Global	410	Neoplasms	Percent	2021	0.145688752
Deaths	Global	410	Neoplasms	Rate	2021	125.3069415

Deaths	Global	558	Mental disorders	Number	2021	231.4898294
Deaths	Global	558	Mental disorders	Percent	2021	3.41E-06
Deaths	Global	558	Mental disorders	Rate	2021	0.002933462
Deaths	Global	491	Cardiovascular diseases	Number	2021	19414853.09
Deaths	Global	491	Cardiovascular diseases	Percent	2021	0.286055957
Deaths	Global	491	Cardiovascular diseases	Rate	2021	246.0269151
Deaths	Global	626	Musculoskeletal disorders	Number	2021	118499.499
Deaths	Global	626	Musculoskeletal disorders	Percent	2021	0.001746178
Deaths	Global	626	Musculoskeletal disorders	Rate	2021	1.501637228
Deaths	Global	640	Other non-communicable diseases	Number	2021	1198125.909
Deaths	Global	640	Other non-communicable diseases	Percent	2021	0.017651058
Deaths	Global	640	Other non-communicable diseases	Rate	2021	15.18276858
Deaths	Global	653	Skin and subcutaneous diseases	Number	2021	119128.8526
Deaths	Global	653	Skin and subcutaneous diseases	Percent	2021	0.00175557
Deaths	Global	653	Skin and subcutaneous diseases	Rate	2021	1.509612458
Deaths	Global	526	Digestive diseases	Number	2021	2516332.3
Deaths	Global	526	Digestive diseases	Percent	2021	0.037076365
Deaths	Global	526	Digestive diseases	Rate	2021	31.88720875
Deaths	Global	973	Substance use disorders	Number	2021	295746.6134
Deaths	Global	973	Substance use disorders	Percent	2021	0.004358188
Deaths	Global	973	Substance use disorders	Rate	2021	3.747729978

Deaths	Global	974	Diabetes and kidney diseases	Number	2021	3195033.813
Deaths	Global	974	Diabetes and kidney diseases	Percent	2021	0.047077771
Deaths	Global	974	Diabetes and kidney diseases	Rate	2021	40.48778063
Deaths	Global	542	Neurological disorders	Number	2021	2607635.055
Deaths	Global	542	Neurological disorders	Percent	2021	0.038417504
Deaths	Global	542	Neurological disorders	Rate	2021	33.04420618
Deaths	Global	508	Chronic respiratory diseases	Number	2021	4414181.576
Deaths	Global	508	Chronic respiratory diseases	Percent	2021	0.065037736
Deaths	Global	508	Chronic respiratory diseases	Rate	2021	55.93694019
**DALY's (total and breakdown by cause)**						
**All NCD's DALY's**						
DALYs (Disability-Adjusted Life Years)	Global	409	Non-communicable diseases	Number	2021	1727188945
DALYs (Disability-Adjusted Life Years)	Global	409	Non-communicable diseases	Percent	2021	0.598764882
DALYs (Disability-Adjusted Life Years)	Global	409	Non-communicable diseases	Rate	2021	21887.10705
**Breakdown**						
DALYs (Disability-Adjusted Life Years)	Global	491	Cardiovascular diseases	Number	2021	428327412.3
DALYs (Disability-Adjusted Life Years)	Global	491	Cardiovascular diseases	Percent	2021	0.148805788
DALYs (Disability-Adjusted Life Years)	Global	491	Cardiovascular diseases	Rate	2021	5427.806815
DALYs (Disability-Adjusted Life Years)	Global	508	Chronic respiratory diseases	Number	2021	108503006.9
DALYs (Disability-Adjusted Life Years)	Global	508	Chronic respiratory diseases	Percent	2021	0.037676617
DALYs (Disability-Adjusted Life Years)	Global	508	Chronic respiratory diseases	Rate	2021	1374.960704
DALYs (Disability-Adjusted Life Years)	Global	410	Neoplasms	Number	2021	253309811.4
DALYs (Disability-Adjusted Life Years)	Global	410	Neoplasms	Percent	2021	0.088016938
DALYs (Disability-Adjusted Life Years)	Global	410	Neoplasms	Rate	2021	3209.966678
DALYs (Disability-Adjusted Life Years)	Global	542	Neurological disorders	Number	2021	112028843.9
DALYs (Disability-Adjusted Life Years)	Global	542	Neurological disorders	Percent	2021	0.038732896

DALYs (Disability-Adjusted Life Years)	Global	542	Neurological disorders	Rate	2021	1419.640454
DALYs (Disability-Adjusted Life Years)	Global	558	Mental disorders	Number	2021	155418119
DALYs (Disability-Adjusted Life Years)	Global	558	Mental disorders	Percent	2021	0.053770186
DALYs (Disability-Adjusted Life Years)	Global	558	Mental disorders	Rate	2021	1969.473588
DALYs (Disability-Adjusted Life Years)	Global	669	Sense organ diseases	Number	2021	77327292.99
DALYs (Disability-Adjusted Life Years)	Global	669	Sense organ diseases	Percent	2021	0.026698525
DALYs (Disability-Adjusted Life Years)	Global	669	Sense organ diseases	Rate	2021	979.8990116
DALYs (Disability-Adjusted Life Years)	Global	526	Digestive diseases	Number	2021	90020095.3
DALYs (Disability-Adjusted Life Years)	Global	526	Digestive diseases	Percent	2021	0.031245831
DALYs (Disability-Adjusted Life Years)	Global	526	Digestive diseases	Rate	2021	1140.743442
DALYs (Disability-Adjusted Life Years)	Global	626	Musculoskeletal disorders	Number	2021	161877699.3
DALYs (Disability-Adjusted Life Years)	Global	626	Musculoskeletal disorders	Percent	2021	0.055936624
DALYs (Disability-Adjusted Life Years)	Global	626	Musculoskeletal disorders	Rate	2021	2051.330021
DALYs (Disability-Adjusted Life Years)	Global	973	Substance use disorders	Number	2021	32543145.76
DALYs (Disability-Adjusted Life Years)	Global	973	Substance use disorders	Percent	2021	0.011277407
DALYs (Disability-Adjusted Life Years)	Global	973	Substance use disorders	Rate	2021	412.3899225
DALYs (Disability-Adjusted Life Years)	Global	974	Diabetes and kidney diseases	Number	2021	123704573.8
DALYs (Disability-Adjusted Life Years)	Global	974	Diabetes and kidney diseases	Percent	2021	0.042886363
DALYs (Disability-Adjusted Life Years)	Global	974	Diabetes and kidney diseases	Rate	2021	1567.596445
DALYs (Disability-Adjusted Life Years)	Global	640	Other non-communicable diseases	Number	2021	142184835.3
DALYs (Disability-Adjusted Life Years)	Global	640	Other non-communicable diseases	Percent	2021	0.049230727
DALYs (Disability-Adjusted Life Years)	Global	640	Other non-communicable diseases	Rate	2021	1801.780124

DALYs (Disability-Adjusted Life Years)	Global	653	Skin and subcutaneous diseases	Number	2021	41944109.06
DALYs (Disability-Adjusted Life Years)	Global	653	Skin and subcutaneous diseases	Percent	2021	0.01448698
DALYs (Disability-Adjusted Life Years)	Global	653	Skin and subcutaneous diseases	Rate	2021	531.5198479

S1B: Global Burden of NCDs by SDI 2021

measure	location	sex	age	Cause	Rate
Deaths	High SDI	Both	All ages	Diabetes mellitus	19.09326
Deaths	High SDI	Both	All ages	Cardiovascular diseases	266.5084
Deaths	Low-middle SDI	Both	All ages	Cardiovascular diseases	199.0894
Deaths	Low-middle SDI	Both	All ages	Neoplasms	63.85014
Deaths	Low SDI	Both	All ages	Neoplasms	42.82801
Deaths	High-middle SDI	Both	All ages	Neoplasms	201.0343
Deaths	Low-middle SDI	Both	All ages	Diabetes mellitus	23.52157
Deaths	Low SDI	Both	All ages	Chronic respiratory diseases	32.71428
Deaths	Low SDI	Both	All ages	Diabetes mellitus	14.55883
Deaths	High-middle SDI	Both	All ages	Diabetes mellitus	18.43553
Deaths	Low-middle SDI	Both	All ages	Chronic respiratory diseases	65.71724
Deaths	Middle SDI	Both	All ages	Cardiovascular diseases	259.1184
Deaths	Low SDI	Both	All ages	Cardiovascular diseases	111.4195
Deaths	Middle SDI	Both	All ages	Chronic respiratory diseases	59.60968
Deaths	Middle SDI	Both	All ages	Neoplasms	117.0772
Deaths	Middle SDI	Both	All ages	Diabetes mellitus	24.12748
Deaths	High SDI	Both	All ages	Chronic respiratory diseases	52.92944

Deaths	High-middle SDI	Both	All ages	Chronic respiratory diseases	57.14509
Deaths	High-middle SDI	Both	All ages	Cardiovascular diseases	388.3673
Deaths	High SDI	Both	All ages	Neoplasms	245.3958
DALYs (Disability-Adjusted Life Years)	Low-middle SDI	Both	All ages	Cardiovascular diseases	5142.211
DALYs (Disability-Adjusted Life Years)	Low-middle SDI	Both	All ages	Chronic respiratory diseases	1652.236
DALYs (Disability-Adjusted Life Years)	Low SDI	Both	All ages	Neoplasms	1484.928
DALYs (Disability-Adjusted Life Years)	High-middle SDI	Both	All ages	Neoplasms	4935.233
DALYs (Disability-Adjusted Life Years)	High-middle SDI	Both	All ages	Chronic respiratory diseases	1236.454
DALYs (Disability-Adjusted Life Years)	Middle SDI	Both	All ages	Diabetes mellitus	1102.485
DALYs (Disability-Adjusted Life Years)	High-middle SDI	Both	All ages	Cardiovascular diseases	7505.865
DALYs (Disability-Adjusted Life Years)	High-middle SDI	Both	All ages	Diabetes mellitus	974.0967
DALYs (Disability-Adjusted Life Years)	High SDI	Both	All ages	Chronic respiratory diseases	1382.244
DALYs (Disability-Adjusted Life Years)	High SDI	Both	All ages	Neoplasms	5113.384
DALYs (Disability-Adjusted Life Years)	High SDI	Both	All ages	Cardiovascular diseases	4879.545
DALYs (Disability-Adjusted Life Years)	High SDI	Both	All ages	Diabetes mellitus	1196.55
DALYs (Disability-Adjusted Life Years)	Low-middle SDI	Both	All ages	Diabetes mellitus	987.8646
DALYs (Disability-Adjusted Life Years)	Low-middle SDI	Both	All ages	Neoplasms	1985.187
DALYs (Disability-Adjusted Life Years)	Low SDI	Both	All ages	Chronic respiratory diseases	1057.472
DALYs (Disability-Adjusted Life Years)	Low SDI	Both	All ages	Cardiovascular diseases	3162.341
DALYs (Disability-Adjusted Life Years)	Low SDI	Both	All ages	Diabetes mellitus	633.7402
DALYs (Disability-Adjusted Life Years)	Middle SDI	Both	All ages	Neoplasms	3186.706
DALYs (Disability-Adjusted Life Years)	Middle SDI	Both	All ages	Cardiovascular diseases	5820.562
DALYs (Disability-Adjusted Life Years)	Middle SDI	Both	All ages	Chronic respiratory diseases	1373.402
Incidence	High SDI	Both	All ages	Neoplasms	2483.941

Incidence	Middle SDI	Both	All ages	Diabetes mellitus	322.894
Incidence	High-middle SDI	Both	All ages	Diabetes mellitus	322.894
Incidence	Low SDI	Both	All ages	Neoplasms	237.9505
Incidence	Low-middle SDI	Both	All ages	Diabetes mellitus	278.0319
Incidence	High SDI	Both	All ages	Cardiovascular diseases	1107.888
Incidence	High SDI	Both	All ages	Chronic respiratory diseases	993.7229
Incidence	Middle SDI	Both	All ages	Neoplasms	630.3272
Incidence	Low SDI	Both	All ages	Cardiovascular diseases	444.2298
Incidence	Middle SDI	Both	All ages	Cardiovascular diseases	857.5398
Incidence	High-middle SDI	Both	All ages	Neoplasms	1017.434
Incidence	Low SDI	Both	All ages	Chronic respiratory diseases	819.7198
Incidence	Middle SDI	Both	All ages	Chronic respiratory diseases	632.0702
Incidence	High SDI	Both	All ages	Diabetes mellitus	461.4124
Incidence	Low-middle SDI	Both	All ages	Neoplasms	410.7845
Incidence	High-middle SDI	Both	All ages	Cardiovascular diseases	1195.303
Incidence	Low SDI	Both	All ages	Diabetes mellitus	171.0263
Incidence	High-middle SDI	Both	All ages	Chronic respiratory diseases	610.4903
Incidence	Low-middle SDI	Both	All ages	Cardiovascular diseases	681.1433
Incidence	Low-middle SDI	Both	All ages	Chronic respiratory diseases	608.4159

S1C: Global Burden of NCDs by WHO regions 2021

measure	location	sex	age	cause	Rate
Deaths	African Region	Both	All ages	Neoplasms	45.59254
Deaths	African Region	Both	All ages	Diabetes mellitus	16.94496
Deaths	Region of the Americas	Both	All ages	Neoplasms	151.6154

Deaths	African Region	Both	All ages	Cardiovascular diseases	102.7976
Deaths	African Region	Both	All ages	Chronic respiratory diseases	15.23393
Deaths	Region of the Americas	Both	All ages	Diabetes mellitus	30.26774
Deaths	Region of the Americas	Both	All ages	Chronic respiratory diseases	43.84958
Deaths	Region of the Americas	Both	All ages	Cardiovascular diseases	200.38
Deaths	South-East Asia Region	Both	All ages	Chronic respiratory diseases	82.16835
Deaths	South-East Asia Region	Both	All ages	Cardiovascular diseases	219.3536
Deaths	South-East Asia Region	Both	All ages	Neoplasms	68.26944
Deaths	South-East Asia Region	Both	All ages	Diabetes mellitus	24.57122
Deaths	European Region	Both	All ages	Neoplasms	235.7094
Deaths	European Region	Both	All ages	Cardiovascular diseases	419.6163
Deaths	European Region	Both	All ages	Chronic respiratory diseases	41.83066
Deaths	Eastern Mediterranean Region	Both	All ages	Cardiovascular diseases	189.2116
Deaths	Eastern Mediterranean Region	Both	All ages	Diabetes mellitus	18.28974
Deaths	Eastern Mediterranean Region	Both	All ages	Neoplasms	56.88783
Deaths	European Region	Both	All ages	Diabetes mellitus	21.95952
Deaths	Eastern Mediterranean Region	Both	All ages	Chronic respiratory diseases	21.80764
Deaths	Western Pacific Region	Both	All ages	Cardiovascular diseases	323.7028
Deaths	Western Pacific Region	Both	All ages	Chronic respiratory diseases	79.16171
Deaths	Western Pacific Region	Both	All ages	Neoplasms	191.8787
Deaths	Western Pacific Region	Both	All ages	Diabetes mellitus	14.75882
DALYs (Disability-Adjusted Life Years)	Western Pacific Region	Both	All ages	Chronic respiratory diseases	1605.858
DALYs (Disability-Adjusted Life Years)	African Region	Both	All ages	Neoplasms	1538.737
DALYs (Disability-Adjusted Life Years)	African Region	Both	All ages	Cardiovascular diseases	2841.951

DALYs (Disability-Adjusted Life Years)	African Region	Both	All ages	Diabetes mellitus	674.205
DALYs (Disability-Adjusted Life Years)	Region of the Americas	Both	All ages	Neoplasms	3688.906
DALYs (Disability-Adjusted Life Years)	Region of the Americas	Both	All ages	Cardiovascular diseases	4255.895
DALYs (Disability-Adjusted Life Years)	African Region	Both	All ages	Chronic respiratory diseases	671.8589
DALYs (Disability-Adjusted Life Years)	Region of the Americas	Both	All ages	Chronic respiratory diseases	1224.141
DALYs (Disability-Adjusted Life Years)	South-East Asia Region	Both	All ages	Chronic respiratory diseases	2011.384
DALYs (Disability-Adjusted Life Years)	Region of the Americas	Both	All ages	Diabetes mellitus	1472.085
DALYs (Disability-Adjusted Life Years)	European Region	Both	All ages	Neoplasms	5257.475
DALYs (Disability-Adjusted Life Years)	European Region	Both	All ages	Cardiovascular diseases	7698.928
DALYs (Disability-Adjusted Life Years)	European Region	Both	All ages	Diabetes mellitus	1011.265
DALYs (Disability-Adjusted Life Years)	South-East Asia Region	Both	All ages	Cardiovascular diseases	5617.149
DALYs (Disability-Adjusted Life Years)	Eastern Mediterranean Region	Both	All ages	Neoplasms	1867.783
DALYs (Disability-Adjusted Life Years)	South-East Asia Region	Both	All ages	Neoplasms	2060.041
DALYs (Disability-Adjusted Life Years)	South-East Asia Region	Both	All ages	Diabetes mellitus	1027.445
DALYs (Disability-Adjusted Life Years)	European Region	Both	All ages	Chronic respiratory diseases	1057.839
DALYs (Disability-Adjusted Life Years)	Western Pacific Region	Both	All ages	Cardiovascular diseases	6481.026
DALYs (Disability-Adjusted Life Years)	Western Pacific Region	Both	All ages	Diabetes mellitus	901.8399
DALYs (Disability-Adjusted Life Years)	Eastern Mediterranean Region	Both	All ages	Diabetes mellitus	981.8057
DALYs (Disability-Adjusted Life Years)	Eastern Mediterranean Region	Both	All ages	Cardiovascular diseases	5015.271
DALYs (Disability-Adjusted Life Years)	Eastern Mediterranean Region	Both	All ages	Chronic respiratory diseases	734.7992
DALYs (Disability-Adjusted Life Years)	Western Pacific Region	Both	All ages	Neoplasms	4687.513
Incidence	African Region	Both	All ages	Neoplasms	205.1715
Incidence	Eastern Mediterranean Region	Both	All ages	Diabetes mellitus	387.6134
Incidence	South-East Asia Region	Both	All ages	Neoplasms	507.3475

Incidence	European Region	Both	All ages	Chronic respiratory diseases	728.3822
Incidence	African Region	Both	All ages	Cardiovascular diseases	430.6976
Incidence	Western Pacific Region	Both	All ages	Cardiovascular diseases	1024.063
Incidence	Eastern Mediterranean Region	Both	All ages	Neoplasms	341.8659
Incidence	African Region	Both	All ages	Chronic respiratory diseases	804.9187
Incidence	Western Pacific Region	Both	All ages	Chronic respiratory diseases	619.2842
Incidence	South-East Asia Region	Both	All ages	Cardiovascular diseases	737.2436
Incidence	Region of the Americas	Both	All ages	Diabetes mellitus	452.7114
Incidence	South-East Asia Region	Both	All ages	Chronic respiratory diseases	559.6532
Incidence	European Region	Both	All ages	Diabetes mellitus	323.0867
Incidence	Eastern Mediterranean Region	Both	All ages	Cardiovascular diseases	778.2889
Incidence	Region of the Americas	Both	All ages	Neoplasms	2011.005
Incidence	Eastern Mediterranean Region	Both	All ages	Chronic respiratory diseases	578.9165
Incidence	European Region	Both	All ages	Neoplasms	1261.26
Incidence	Western Pacific Region	Both	All ages	Diabetes mellitus	302.2768
Incidence	African Region	Both	All ages	Diabetes mellitus	160.5863
Incidence	Western Pacific Region	Both	All ages	Neoplasms	951.6521
Incidence	European Region	Both	All ages	Cardiovascular diseases	1318.908
Incidence	South-East Asia Region	Both	All ages	Diabetes mellitus	292.1095
Incidence	Region of the Americas	Both	All ages	Cardiovascular diseases	813.0411
Incidence	Region of the Americas	Both	All ages	Chronic respiratory diseases	1072.28

S2: Countries burden

S2A: Countries CVDs incidence, mortality and DALYS:

measure	location	sex	age	cause	metric	Year	Rate
Deaths	Republic of Tajikistan	Both	All ages	Cardiovascular diseases	Rate	2021	170.9104
Deaths	Republic of Fiji	Both	All ages	Cardiovascular diseases	Rate	2021	304.5465
Deaths	New Zealand	Both	All ages	Cardiovascular diseases	Rate	2021	212.5635
Deaths	Syrian Arab Republic	Both	All ages	Cardiovascular diseases	Rate	2021	369.5221
Deaths	Republic of Seychelles	Both	All ages	Cardiovascular diseases	Rate	2021	229.4465
Deaths	Turkmenistan	Both	All ages	Cardiovascular diseases	Rate	2021	365.2527
Deaths	Principality of Monaco	Both	All ages	Cardiovascular diseases	Rate	2021	395.689
Deaths	Democratic People's Republic of Korea	Both	All ages	Cardiovascular diseases	Rate	2021	403.7955
Deaths	Antigua and Barbuda	Both	All ages	Cardiovascular diseases	Rate	2021	227.0269
Deaths	Republic of Chad	Both	All ages	Cardiovascular diseases	Rate	2021	93.48344
Deaths	Republic of the Congo	Both	All ages	Cardiovascular diseases	Rate	2021	155.2008
Deaths	Republic of Kiribati	Both	All ages	Cardiovascular diseases	Rate	2021	226.829
Deaths	Republic of Cabo Verde	Both	All ages	Cardiovascular diseases	Rate	2021	203.7102
Deaths	Republic of CÃ´te d'Ivoire	Both	All ages	Cardiovascular diseases	Rate	2021	104.5301
Deaths	Republic of Slovenia	Both	All ages	Cardiovascular diseases	Rate	2021	350.0545
Deaths	Republic of Nauru	Both	All ages	Cardiovascular diseases	Rate	2021	352.3582
Deaths	Plurinational State of Bolivia	Both	All ages	Cardiovascular diseases	Rate	2021	120.15
Deaths	People's Democratic Republic of Algeria	Both	All ages	Cardiovascular diseases	Rate	2021	220.9214
Deaths	Republic of Cameroon	Both	All ages	Cardiovascular diseases	Rate	2021	102.4203
Deaths	Federated States of Micronesia	Both	All ages	Cardiovascular diseases	Rate	2021	323.1235

Deaths	Republic of Peru	Both	All ages	Cardiovascular diseases	Rate	2021	83.27492
Deaths	United Arab Emirates	Both	All ages	Cardiovascular diseases	Rate	2021	53.1894
Deaths	Democratic Republic of the Congo	Both	All ages	Cardiovascular diseases	Rate	2021	108.0523
Deaths	Republic of Malta	Both	All ages	Cardiovascular diseases	Rate	2021	295.5056
Deaths	Republic of Uzbekistan	Both	All ages	Cardiovascular diseases	Rate	2021	284.4938
Deaths	Republic of Belarus	Both	All ages	Cardiovascular diseases	Rate	2021	815.2396
Deaths	Republic of Niue	Both	All ages	Cardiovascular diseases	Rate	2021	522.7974
Deaths	Commonwealth of the Bahamas	Both	All ages	Cardiovascular diseases	Rate	2021	214.8481
Deaths	Republic of Turkey	Both	All ages	Cardiovascular diseases	Rate	2021	246.0655
Deaths	Taiwan (Province of China)	Both	All ages	Cardiovascular diseases	Rate	2021	165.6135
Deaths	Republic of Tunisia	Both	All ages	Cardiovascular diseases	Rate	2021	295.6169
Deaths	Independent State of Papua New Guinea	Both	All ages	Cardiovascular diseases	Rate	2021	151.7704
Deaths	Republic of Uganda	Both	All ages	Cardiovascular diseases	Rate	2021	62.61418
Deaths	Republic of Zambia	Both	All ages	Cardiovascular diseases	Rate	2021	99.1149
Deaths	Republic of Austria	Both	All ages	Cardiovascular diseases	Rate	2021	326.0862
Deaths	Republic of the Marshall Islands	Both	All ages	Cardiovascular diseases	Rate	2021	282.4969
Deaths	Republic of Yemen	Both	All ages	Cardiovascular diseases	Rate	2021	175.8238
Deaths	Republic of Estonia	Both	All ages	Cardiovascular diseases	Rate	2021	553.2172
Deaths	Kingdom of the Netherlands	Both	All ages	Cardiovascular diseases	Rate	2021	234.964
Deaths	Federal Republic of Somalia	Both	All ages	Cardiovascular diseases	Rate	2021	67.86197
Deaths	Kingdom of Norway	Both	All ages	Cardiovascular diseases	Rate	2021	201.2848
Deaths	Republic of Albania	Both	All ages	Cardiovascular diseases	Rate	2021	526.7413
Deaths	Republic of Equatorial Guinea	Both	All ages	Cardiovascular diseases	Rate	2021	84.81556
Deaths	Republic of Ecuador	Both	All ages	Cardiovascular diseases	Rate	2021	117.9356

Deaths	Kingdom of Cambodia	Both	All ages	Cardiovascular diseases	Rate	2021	195.148
Deaths	Belize	Both	All ages	Cardiovascular diseases	Rate	2021	104.9535
Deaths	Republic of Palau	Both	All ages	Cardiovascular diseases	Rate	2021	398.4745
Deaths	Arab Republic of Egypt	Both	All ages	Cardiovascular diseases	Rate	2021	260.979
Deaths	Republic of Bulgaria	Both	All ages	Cardiovascular diseases	Rate	2021	1114.149
Deaths	Kingdom of Spain	Both	All ages	Cardiovascular diseases	Rate	2021	253.0996
Deaths	Principality of Andorra	Both	All ages	Cardiovascular diseases	Rate	2021	182.6014
Deaths	Republic of Cuba	Both	All ages	Cardiovascular diseases	Rate	2021	366.2998
Deaths	Republic of Latvia	Both	All ages	Cardiovascular diseases	Rate	2021	797.2532
Deaths	Northern Mariana Islands	Both	All ages	Cardiovascular diseases	Rate	2021	242.5195
Deaths	Islamic Republic of Iran	Both	All ages	Cardiovascular diseases	Rate	2021	198.6763
Deaths	Barbados	Both	All ages	Cardiovascular diseases	Rate	2021	311.9956
Deaths	Gabonese Republic	Both	All ages	Cardiovascular diseases	Rate	2021	153.0836
Deaths	Republic of Colombia	Both	All ages	Cardiovascular diseases	Rate	2021	152.3576
Deaths	Kingdom of Belgium	Both	All ages	Cardiovascular diseases	Rate	2021	230.1972
Deaths	Lao People's Democratic Republic	Both	All ages	Cardiovascular diseases	Rate	2021	212.8261
Deaths	United Republic of Tanzania	Both	All ages	Cardiovascular diseases	Rate	2021	97.36604
Deaths	Bosnia and Herzegovina	Both	All ages	Cardiovascular diseases	Rate	2021	568.2684
Deaths	Republic of Moldova	Both	All ages	Cardiovascular diseases	Rate	2021	609.943
Deaths	Portuguese Republic	Both	All ages	Cardiovascular diseases	Rate	2021	308.0714
Deaths	Republic of Ghana	Both	All ages	Cardiovascular diseases	Rate	2021	140.614
Deaths	Republic of Iraq	Both	All ages	Cardiovascular diseases	Rate	2021	212.3864
Deaths	Republic of the Gambia	Both	All ages	Cardiovascular diseases	Rate	2021	134.9003
Deaths	Republic of Cyprus	Both	All ages	Cardiovascular diseases	Rate	2021	223.7726

Deaths	Kingdom of Tonga	Both	All ages	Cardiovascular diseases	Rate	2021	176.4224
Deaths	Commonwealth of Dominica	Both	All ages	Cardiovascular diseases	Rate	2021	316.5098
Deaths	Independent State of Samoa	Both	All ages	Cardiovascular diseases	Rate	2021	257.6356
Deaths	Union of the Comoros	Both	All ages	Cardiovascular diseases	Rate	2021	136.7785
Deaths	Kingdom of Bahrain	Both	All ages	Cardiovascular diseases	Rate	2021	91.28208
Deaths	Puerto Rico	Both	All ages	Cardiovascular diseases	Rate	2021	247.7963
Deaths	Republic of Costa Rica	Both	All ages	Cardiovascular diseases	Rate	2021	129.1058
Deaths	Kingdom of Sweden	Both	All ages	Cardiovascular diseases	Rate	2021	285.5856
Deaths	Republic of Indonesia	Both	All ages	Cardiovascular diseases	Rate	2021	274.5146
Deaths	Malaysia	Both	All ages	Cardiovascular diseases	Rate	2021	209.916
Deaths	Republic of Guinea	Both	All ages	Cardiovascular diseases	Rate	2021	123.7671
Deaths	Republic of Guinea-Bissau	Both	All ages	Cardiovascular diseases	Rate	2021	132.2854
Deaths	Republic of Lithuania	Both	All ages	Cardiovascular diseases	Rate	2021	741.3917
Deaths	Republic of Burundi	Both	All ages	Cardiovascular diseases	Rate	2021	85.21759
Deaths	Republic of Liberia	Both	All ages	Cardiovascular diseases	Rate	2021	106.0866
Deaths	Republic of Croatia	Both	All ages	Cardiovascular diseases	Rate	2021	555.8308
Deaths	Islamic Republic of Afghanistan	Both	All ages	Cardiovascular diseases	Rate	2021	152.3177
Deaths	Republic of Maldives	Both	All ages	Cardiovascular diseases	Rate	2021	99.27103
Deaths	Kingdom of Denmark	Both	All ages	Cardiovascular diseases	Rate	2021	221.7843
Deaths	Republic of El Salvador	Both	All ages	Cardiovascular diseases	Rate	2021	149.4619
Deaths	Solomon Islands	Both	All ages	Cardiovascular diseases	Rate	2021	232.7239
Deaths	French Republic	Both	All ages	Cardiovascular diseases	Rate	2021	230.0465
Deaths	State of Eritrea	Both	All ages	Cardiovascular diseases	Rate	2021	106.5005
Deaths	Republic of Sudan	Both	All ages	Cardiovascular diseases	Rate	2021	171.5824

Deaths	Republic of Vanuatu	Both	All ages	Cardiovascular diseases	Rate	2021	259.8522
Deaths	People's Republic of Bangladesh	Both	All ages	Cardiovascular diseases	Rate	2021	229.1112
Deaths	Saint Kitts and Nevis	Both	All ages	Cardiovascular diseases	Rate	2021	256.2312
Deaths	Swiss Confederation	Both	All ages	Cardiovascular diseases	Rate	2021	227.4697
Deaths	Republic of Guyana	Both	All ages	Cardiovascular diseases	Rate	2021	278.0295
Deaths	Republic of San Marino	Both	All ages	Cardiovascular diseases	Rate	2021	214.6283
Deaths	Dominican Republic	Both	All ages	Cardiovascular diseases	Rate	2021	222.5514
Deaths	Federal Democratic Republic of Ethiopia	Both	All ages	Cardiovascular diseases	Rate	2021	63.40494
Deaths	Republic of Finland	Both	All ages	Cardiovascular diseases	Rate	2021	407.3518
Deaths	North Macedonia	Both	All ages	Cardiovascular diseases	Rate	2021	610.5557
Deaths	State of Kuwait	Both	All ages	Cardiovascular diseases	Rate	2021	90.34182
Deaths	Republic of South Africa	Both	All ages	Cardiovascular diseases	Rate	2021	173.4349
Deaths	Czech Republic	Both	All ages	Cardiovascular diseases	Rate	2021	440.1087
Deaths	Ukraine	Both	All ages	Cardiovascular diseases	Rate	2021	954.142
Deaths	Republic of Honduras	Both	All ages	Cardiovascular diseases	Rate	2021	172.6222
Deaths	Republic of Guatemala	Both	All ages	Cardiovascular diseases	Rate	2021	92.02017
Deaths	Russian Federation	Both	All ages	Cardiovascular diseases	Rate	2021	648.4162
Deaths	Republic of Namibia	Both	All ages	Cardiovascular diseases	Rate	2021	154.3108
Deaths	Republic of the Philippines	Both	All ages	Cardiovascular diseases	Rate	2021	199.5058
Deaths	Bermuda	Both	All ages	Cardiovascular diseases	Rate	2021	331.359
Deaths	Grenada	Both	All ages	Cardiovascular diseases	Rate	2021	249.8726
Deaths	Tokelau	Both	All ages	Cardiovascular diseases	Rate	2021	411.8005
Deaths	American Samoa	Both	All ages	Cardiovascular diseases	Rate	2021	279.9354
Deaths	Republic of Botswana	Both	All ages	Cardiovascular diseases	Rate	2021	120.7458

Deaths	Tuvalu	Both	All ages	Cardiovascular diseases	Rate	2021	370.059
Deaths	Democratic Socialist Republic of Sri Lanka	Both	All ages	Cardiovascular diseases	Rate	2021	246.0574
Deaths	Hashemite Kingdom of Jordan	Both	All ages	Cardiovascular diseases	Rate	2021	94.54161
Deaths	Lebanese Republic	Both	All ages	Cardiovascular diseases	Rate	2021	195.9253
Deaths	United Mexican States	Both	All ages	Cardiovascular diseases	Rate	2021	146.7526
Deaths	Republic of India	Both	All ages	Cardiovascular diseases	Rate	2021	203.1302
Deaths	Republic of Djibouti	Both	All ages	Cardiovascular diseases	Rate	2021	103.9429
Deaths	Kingdom of Bhutan	Both	All ages	Cardiovascular diseases	Rate	2021	172.0399
Deaths	Kingdom of Morocco	Both	All ages	Cardiovascular diseases	Rate	2021	362.9177
Deaths	Hellenic Republic	Both	All ages	Cardiovascular diseases	Rate	2021	472.6179
Deaths	Jamaica	Both	All ages	Cardiovascular diseases	Rate	2021	228.3652
Deaths	Argentine Republic	Both	All ages	Cardiovascular diseases	Rate	2021	192.1456
Deaths	Republic of Benin	Both	All ages	Cardiovascular diseases	Rate	2021	87.88113
Deaths	Republic of Madagascar	Both	All ages	Cardiovascular diseases	Rate	2021	131.7626
Deaths	Guam	Both	All ages	Cardiovascular diseases	Rate	2021	247.8823
Deaths	Kingdom of Lesotho	Both	All ages	Cardiovascular diseases	Rate	2021	194.88
Deaths	Kingdom of Thailand	Both	All ages	Cardiovascular diseases	Rate	2021	205.0902
Deaths	Georgia	Both	All ages	Cardiovascular diseases	Rate	2021	631.5979
Deaths	Islamic Republic of Mauritania	Both	All ages	Cardiovascular diseases	Rate	2021	129.6043
Deaths	Brunei Darussalam	Both	All ages	Cardiovascular diseases	Rate	2021	109.9139
Deaths	State of Libya	Both	All ages	Cardiovascular diseases	Rate	2021	204.4656
Deaths	Hungary	Both	All ages	Cardiovascular diseases	Rate	2021	603.9638
Deaths	Republic of Haiti	Both	All ages	Cardiovascular diseases	Rate	2021	216.1306
Deaths	Federal Democratic Republic of Nepal	Both	All ages	Cardiovascular diseases	Rate	2021	178.9196

Deaths	Federal Republic of Germany	Both	All ages	Cardiovascular diseases	Rate	2021	373.6903
Deaths	Burkina Faso	Both	All ages	Cardiovascular diseases	Rate	2021	98.32079
Deaths	Republic of the Niger	Both	All ages	Cardiovascular diseases	Rate	2021	70.1954
Deaths	Republic of the Union of Myanmar	Both	All ages	Cardiovascular diseases	Rate	2021	269.5129
Deaths	Republic of Iceland	Both	All ages	Cardiovascular diseases	Rate	2021	200.1918
Deaths	Canada	Both	All ages	Cardiovascular diseases	Rate	2021	201.8206
Deaths	Cook Islands	Both	All ages	Cardiovascular diseases	Rate	2021	337.3435
Deaths	Republic of Mali	Both	All ages	Cardiovascular diseases	Rate	2021	73.65989
Deaths	United Kingdom of Great Britain and Northern Ireland	Both	All ages	Cardiovascular diseases	Rate	2021	235.4216
Deaths	Republic of Azerbaijan	Both	All ages	Cardiovascular diseases	Rate	2021	340.2126
Deaths	Republic of Chile	Both	All ages	Cardiovascular diseases	Rate	2021	144.4916
Deaths	Republic of Kenya	Both	All ages	Cardiovascular diseases	Rate	2021	75.71321
Deaths	Republic of Korea	Both	All ages	Cardiovascular diseases	Rate	2021	149.8639
Deaths	Islamic Republic of Pakistan	Both	All ages	Cardiovascular diseases	Rate	2021	152.3499
Deaths	Montenegro	Both	All ages	Cardiovascular diseases	Rate	2021	700.0658
Deaths	Saint Vincent and the Grenadines	Both	All ages	Cardiovascular diseases	Rate	2021	307.5779
Deaths	Ireland	Both	All ages	Cardiovascular diseases	Rate	2021	167.8367
Deaths	Saint Lucia	Both	All ages	Cardiovascular diseases	Rate	2021	267.2389
Deaths	Democratic Republic of Sao Tome and Principe	Both	All ages	Cardiovascular diseases	Rate	2021	125.4623
Deaths	Republic of Nicaragua	Both	All ages	Cardiovascular diseases	Rate	2021	85.24292
Deaths	Democratic Republic of Timor-Leste	Both	All ages	Cardiovascular diseases	Rate	2021	199.0572
Deaths	Greenland	Both	All ages	Cardiovascular diseases	Rate	2021	179.1212
Deaths	Eastern Republic of Uruguay	Both	All ages	Cardiovascular diseases	Rate	2021	291.9211
Deaths	Republic of Armenia	Both	All ages	Cardiovascular diseases	Rate	2021	451.8903

Deaths	Republic of Mauritius	Both	All ages	Cardiovascular diseases	Rate	2021	279.4267
Deaths	Slovak Republic	Both	All ages	Cardiovascular diseases	Rate	2021	498.3308
Deaths	Federal Republic of Nigeria	Both	All ages	Cardiovascular diseases	Rate	2021	82.58021
Deaths	Republic of Suriname	Both	All ages	Cardiovascular diseases	Rate	2021	231.0934
Deaths	State of Qatar	Both	All ages	Cardiovascular diseases	Rate	2021	35.1931
Deaths	Sultanate of Oman	Both	All ages	Cardiovascular diseases	Rate	2021	98.1845
Deaths	Republic of Panama	Both	All ages	Cardiovascular diseases	Rate	2021	130.4353
Deaths	United States Virgin Islands	Both	All ages	Cardiovascular diseases	Rate	2021	343.7753
Deaths	Kingdom of Saudi Arabia	Both	All ages	Cardiovascular diseases	Rate	2021	145.3173
Deaths	Republic of Serbia	Both	All ages	Cardiovascular diseases	Rate	2021	772.1041
Deaths	Bolivarian Republic of Venezuela	Both	All ages	Cardiovascular diseases	Rate	2021	247.846
Deaths	Japan	Both	All ages	Cardiovascular diseases	Rate	2021	291.2487
Deaths	Republic of Trinidad and Tobago	Both	All ages	Cardiovascular diseases	Rate	2021	288.6581
Deaths	Republic of Italy	Both	All ages	Cardiovascular diseases	Rate	2021	361.5904
Deaths	Republic of Kazakhstan	Both	All ages	Cardiovascular diseases	Rate	2021	343.3875
Deaths	People's Republic of China	Both	All ages	Cardiovascular diseases	Rate	2021	357.4396
Deaths	Kingdom of Eswatini	Both	All ages	Cardiovascular diseases	Rate	2021	139.5557
Deaths	Socialist Republic of Viet Nam	Both	All ages	Cardiovascular diseases	Rate	2021	260.4012
Deaths	Federative Republic of Brazil	Both	All ages	Cardiovascular diseases	Rate	2021	170.0723
Deaths	Republic of Angola	Both	All ages	Cardiovascular diseases	Rate	2021	97.32867
Deaths	Republic of Poland	Both	All ages	Cardiovascular diseases	Rate	2021	441.5827
Deaths	Republic of Mozambique	Both	All ages	Cardiovascular diseases	Rate	2021	108.3821
Deaths	Romania	Both	All ages	Cardiovascular diseases	Rate	2021	770.9972
Deaths	Grand Duchy of Luxembourg	Both	All ages	Cardiovascular diseases	Rate	2021	197.8381

Deaths	Republic of Malawi	Both	All ages	Cardiovascular diseases	Rate	2021	94.4788
Deaths	Republic of Sierra Leone	Both	All ages	Cardiovascular diseases	Rate	2021	127.2245
Deaths	State of Israel	Both	All ages	Cardiovascular diseases	Rate	2021	110.0111
Deaths	Palestine	Both	All ages	Cardiovascular diseases	Rate	2021	125.8062
Deaths	Kyrgyz Republic	Both	All ages	Cardiovascular diseases	Rate	2021	228.4609
Deaths	Australia	Both	All ages	Cardiovascular diseases	Rate	2021	183.2141
Deaths	Republic of South Sudan	Both	All ages	Cardiovascular diseases	Rate	2021	89.96047
Deaths	Togolese Republic	Both	All ages	Cardiovascular diseases	Rate	2021	125.781
Deaths	Republic of Zimbabwe	Both	All ages	Cardiovascular diseases	Rate	2021	129.858
Deaths	Republic of Rwanda	Both	All ages	Cardiovascular diseases	Rate	2021	93.32768
Deaths	Central African Republic	Both	All ages	Cardiovascular diseases	Rate	2021	138.97
Deaths	United States of America	Both	All ages	Cardiovascular diseases	Rate	2021	272.2594
Deaths	Republic of Paraguay	Both	All ages	Cardiovascular diseases	Rate	2021	147.9443
Deaths	Republic of Singapore	Both	All ages	Cardiovascular diseases	Rate	2021	109.8429
Deaths	Mongolia	Both	All ages	Cardiovascular diseases	Rate	2021	206.9652
Deaths	Republic of Senegal	Both	All ages	Cardiovascular diseases	Rate	2021	137.8135
DALYs (Disability-Adjusted Life Years)	Ireland	Both	All ages	Cardiovascular diseases	Rate	2021	2925.543
DALYs (Disability-Adjusted Life Years)	New Zealand	Both	All ages	Cardiovascular diseases	Rate	2021	3630.099
DALYs (Disability-Adjusted Life Years)	Belize	Both	All ages	Cardiovascular diseases	Rate	2021	2649.984
DALYs (Disability-Adjusted Life Years)	Republic of Panama	Both	All ages	Cardiovascular diseases	Rate	2021	2769.532
DALYs (Disability-Adjusted Life Years)	United Arab Emirates	Both	All ages	Cardiovascular diseases	Rate	2021	1977.778
DALYs (Disability-Adjusted Life Years)	Hashemite Kingdom of Jordan	Both	All ages	Cardiovascular diseases	Rate	2021	2473.204
DALYs (Disability-Adjusted Life Years)	Republic of Kenya	Both	All ages	Cardiovascular diseases	Rate	2021	2096.354
DALYs (Disability-Adjusted Life Years)	Republic of Angola	Both	All ages	Cardiovascular diseases	Rate	2021	2837.863

DALYs (Disability-Adjusted Life Years)	Republic of Ecuador	Both	All ages	Cardiovascular diseases	Rate	2021	2628.072
DALYs (Disability-Adjusted Life Years)	Republic of Namibia	Both	All ages	Cardiovascular diseases	Rate	2021	3921.501
DALYs (Disability-Adjusted Life Years)	Lebanese Republic	Both	All ages	Cardiovascular diseases	Rate	2021	3728.019
DALYs (Disability-Adjusted Life Years)	Republic of Malawi	Both	All ages	Cardiovascular diseases	Rate	2021	2762.941
DALYs (Disability-Adjusted Life Years)	Republic of the Congo	Both	All ages	Cardiovascular diseases	Rate	2021	4328.918
DALYs (Disability-Adjusted Life Years)	Kingdom of Tonga	Both	All ages	Cardiovascular diseases	Rate	2021	4305.049
DALYs (Disability-Adjusted Life Years)	Republic of Uzbekistan	Both	All ages	Cardiovascular diseases	Rate	2021	6741.953
DALYs (Disability-Adjusted Life Years)	Republic of Ghana	Both	All ages	Cardiovascular diseases	Rate	2021	3953.952
DALYs (Disability-Adjusted Life Years)	Republic of San Marino	Both	All ages	Cardiovascular diseases	Rate	2021	3774.822
DALYs (Disability-Adjusted Life Years)	Republic of Serbia	Both	All ages	Cardiovascular diseases	Rate	2021	12800.75
DALYs (Disability-Adjusted Life Years)	Canada	Both	All ages	Cardiovascular diseases	Rate	2021	3835.686
DALYs (Disability-Adjusted Life Years)	Democratic Socialist Republic of Sri Lanka	Both	All ages	Cardiovascular diseases	Rate	2021	5281.026
DALYs (Disability-Adjusted Life Years)	Japan	Both	All ages	Cardiovascular diseases	Rate	2021	4695.926
DALYs (Disability-Adjusted Life Years)	Republic of Cyprus	Both	All ages	Cardiovascular diseases	Rate	2021	3839.741
DALYs (Disability-Adjusted Life Years)	Republic of Guyana	Both	All ages	Cardiovascular diseases	Rate	2021	7107.766
DALYs (Disability-Adjusted Life Years)	Cook Islands	Both	All ages	Cardiovascular diseases	Rate	2021	7517.013
DALYs (Disability-Adjusted Life Years)	Kingdom of the Netherlands	Both	All ages	Cardiovascular diseases	Rate	2021	3836.923
DALYs (Disability-Adjusted Life Years)	Republic of Slovenia	Both	All ages	Cardiovascular diseases	Rate	2021	5381.109
DALYs (Disability-Adjusted Life Years)	Republic of Benin	Both	All ages	Cardiovascular diseases	Rate	2021	2398.811
DALYs (Disability-Adjusted Life Years)	Jamaica	Both	All ages	Cardiovascular diseases	Rate	2021	4556.316
DALYs (Disability-Adjusted Life Years)	Republic of India	Both	All ages	Cardiovascular diseases	Rate	2021	5264.945
DALYs (Disability-Adjusted Life Years)	Republic of Finland	Both	All ages	Cardiovascular diseases	Rate	2021	6444.921
DALYs (Disability-Adjusted Life Years)	Republic of El Salvador	Both	All ages	Cardiovascular diseases	Rate	2021	3084.127
DALYs (Disability-Adjusted Life Years)	People's Democratic Republic of Algeria	Both	All ages	Cardiovascular diseases	Rate	2021	4728.869

DALYs (Disability-Adjusted Life Years)	Republic of Cameroon	Both	All ages	Cardiovascular diseases	Rate	2021	3003
DALYs (Disability-Adjusted Life Years)	Republic of Burundi	Both	All ages	Cardiovascular diseases	Rate	2021	2576.97
DALYs (Disability-Adjusted Life Years)	Federal Republic of Somalia	Both	All ages	Cardiovascular diseases	Rate	2021	2282.146
DALYs (Disability-Adjusted Life Years)	Republic of Croatia	Both	All ages	Cardiovascular diseases	Rate	2021	8803.003
DALYs (Disability-Adjusted Life Years)	Kingdom of Cambodia	Both	All ages	Cardiovascular diseases	Rate	2021	4871.667
DALYs (Disability-Adjusted Life Years)	State of Qatar	Both	All ages	Cardiovascular diseases	Rate	2021	1265.975
DALYs (Disability-Adjusted Life Years)	Republic of Fiji	Both	All ages	Cardiovascular diseases	Rate	2021	8625.159
DALYs (Disability-Adjusted Life Years)	Portuguese Republic	Both	All ages	Cardiovascular diseases	Rate	2021	4879.735
DALYs (Disability-Adjusted Life Years)	Republic of Azerbaijan	Both	All ages	Cardiovascular diseases	Rate	2021	7675.244
DALYs (Disability-Adjusted Life Years)	Islamic Republic of Mauritania	Both	All ages	Cardiovascular diseases	Rate	2021	3217.919
DALYs (Disability-Adjusted Life Years)	Principality of Monaco	Both	All ages	Cardiovascular diseases	Rate	2021	6136.397
DALYs (Disability-Adjusted Life Years)	Republic of the Marshall Islands	Both	All ages	Cardiovascular diseases	Rate	2021	9311.49
DALYs (Disability-Adjusted Life Years)	Republic of Uganda	Both	All ages	Cardiovascular diseases	Rate	2021	1876.589
DALYs (Disability-Adjusted Life Years)	Hungary	Both	All ages	Cardiovascular diseases	Rate	2021	10671.26
DALYs (Disability-Adjusted Life Years)	Republic of Colombia	Both	All ages	Cardiovascular diseases	Rate	2021	3166.889
DALYs (Disability-Adjusted Life Years)	Lao People's Democratic Republic	Both	All ages	Cardiovascular diseases	Rate	2021	5780.989
DALYs (Disability-Adjusted Life Years)	Palestine	Both	All ages	Cardiovascular diseases	Rate	2021	3017.38
DALYs (Disability-Adjusted Life Years)	Republic of Kazakhstan	Both	All ages	Cardiovascular diseases	Rate	2021	7694.692
DALYs (Disability-Adjusted Life Years)	Slovak Republic	Both	All ages	Cardiovascular diseases	Rate	2021	9096.746
DALYs (Disability-Adjusted Life Years)	Republic of Zimbabwe	Both	All ages	Cardiovascular diseases	Rate	2021	3828.401
DALYs (Disability-Adjusted Life Years)	Federal Republic of Nigeria	Both	All ages	Cardiovascular diseases	Rate	2021	2299.564
DALYs (Disability-Adjusted Life Years)	Gabonese Republic	Both	All ages	Cardiovascular diseases	Rate	2021	3897.341
DALYs (Disability-Adjusted Life Years)	Republic of Rwanda	Both	All ages	Cardiovascular diseases	Rate	2021	2581.114
DALYs (Disability-Adjusted Life Years)	Democratic People's Republic of Korea	Both	All ages	Cardiovascular diseases	Rate	2021	9475.355

DALYs (Disability-Adjusted Life Years)	Kingdom of Denmark	Both	All ages	Cardiovascular diseases	Rate	2021	3736.346
DALYs (Disability-Adjusted Life Years)	Republic of Lithuania	Both	All ages	Cardiovascular diseases	Rate	2021	12191.77
DALYs (Disability-Adjusted Life Years)	Taiwan (Province of China)	Both	All ages	Cardiovascular diseases	Rate	2021	3624.569
DALYs (Disability-Adjusted Life Years)	People's Republic of Bangladesh	Both	All ages	Cardiovascular diseases	Rate	2021	5455.528
DALYs (Disability-Adjusted Life Years)	Brunei Darussalam	Both	All ages	Cardiovascular diseases	Rate	2021	3157.215
DALYs (Disability-Adjusted Life Years)	Republic of Liberia	Both	All ages	Cardiovascular diseases	Rate	2021	3110.405
DALYs (Disability-Adjusted Life Years)	Republic of Haiti	Both	All ages	Cardiovascular diseases	Rate	2021	6194.161
DALYs (Disability-Adjusted Life Years)	Kingdom of Norway	Both	All ages	Cardiovascular diseases	Rate	2021	3319.301
DALYs (Disability-Adjusted Life Years)	United States Virgin Islands	Both	All ages	Cardiovascular diseases	Rate	2021	6852.678
DALYs (Disability-Adjusted Life Years)	Bosnia and Herzegovina	Both	All ages	Cardiovascular diseases	Rate	2021	10202.67
DALYs (Disability-Adjusted Life Years)	Republic of Costa Rica	Both	All ages	Cardiovascular diseases	Rate	2021	2859.798
DALYs (Disability-Adjusted Life Years)	Democratic Republic of Timor-Leste	Both	All ages	Cardiovascular diseases	Rate	2021	4978.566
DALYs (Disability-Adjusted Life Years)	Republic of Albania	Both	All ages	Cardiovascular diseases	Rate	2021	8933.393
DALYs (Disability-Adjusted Life Years)	United States of America	Both	All ages	Cardiovascular diseases	Rate	2021	5339.031
DALYs (Disability-Adjusted Life Years)	Socialist Republic of Viet Nam	Both	All ages	Cardiovascular diseases	Rate	2021	5845.684
DALYs (Disability-Adjusted Life Years)	Sultanate of Oman	Both	All ages	Cardiovascular diseases	Rate	2021	2806.617
DALYs (Disability-Adjusted Life Years)	Tuvalu	Both	All ages	Cardiovascular diseases	Rate	2021	10036.61
DALYs (Disability-Adjusted Life Years)	Republic of Niue	Both	All ages	Cardiovascular diseases	Rate	2021	12233.5
DALYs (Disability-Adjusted Life Years)	Kingdom of Bhutan	Both	All ages	Cardiovascular diseases	Rate	2021	4009.237
DALYs (Disability-Adjusted Life Years)	Guam	Both	All ages	Cardiovascular diseases	Rate	2021	6924.022
DALYs (Disability-Adjusted Life Years)	Kingdom of Spain	Both	All ages	Cardiovascular diseases	Rate	2021	4178.843
DALYs (Disability-Adjusted Life Years)	Republic of Korea	Both	All ages	Cardiovascular diseases	Rate	2021	2929.055
DALYs (Disability-Adjusted Life Years)	French Republic	Both	All ages	Cardiovascular diseases	Rate	2021	4024.295
DALYs (Disability-Adjusted Life Years)	Republic of Seychelles	Both	All ages	Cardiovascular diseases	Rate	2021	5415.731

DALYs (Disability-Adjusted Life Years)	Republic of Singapore	Both	All ages	Cardiovascular diseases	Rate	2021	2526.627
DALYs (Disability-Adjusted Life Years)	Republic of Mali	Both	All ages	Cardiovascular diseases	Rate	2021	2257.55
DALYs (Disability-Adjusted Life Years)	Saint Lucia	Both	All ages	Cardiovascular diseases	Rate	2021	5451.461
DALYs (Disability-Adjusted Life Years)	Antigua and Barbuda	Both	All ages	Cardiovascular diseases	Rate	2021	4925.498
DALYs (Disability-Adjusted Life Years)	Federal Republic of Germany	Both	All ages	Cardiovascular diseases	Rate	2021	6053.465
DALYs (Disability-Adjusted Life Years)	Republic of Estonia	Both	All ages	Cardiovascular diseases	Rate	2021	8723.315
DALYs (Disability-Adjusted Life Years)	Republic of Armenia	Both	All ages	Cardiovascular diseases	Rate	2021	8566.167
DALYs (Disability-Adjusted Life Years)	Union of the Comoros	Both	All ages	Cardiovascular diseases	Rate	2021	3564.746
DALYs (Disability-Adjusted Life Years)	United Republic of Tanzania	Both	All ages	Cardiovascular diseases	Rate	2021	2663.386
DALYs (Disability-Adjusted Life Years)	Republic of Belarus	Both	All ages	Cardiovascular diseases	Rate	2021	15491.92
DALYs (Disability-Adjusted Life Years)	Republic of Bulgaria	Both	All ages	Cardiovascular diseases	Rate	2021	19749.23
DALYs (Disability-Adjusted Life Years)	Kingdom of Saudi Arabia	Both	All ages	Cardiovascular diseases	Rate	2021	4807.682
DALYs (Disability-Adjusted Life Years)	Republic of Kiribati	Both	All ages	Cardiovascular diseases	Rate	2021	7537.054
DALYs (Disability-Adjusted Life Years)	Federal Democratic Republic of Nepal	Both	All ages	Cardiovascular diseases	Rate	2021	4461.629
DALYs (Disability-Adjusted Life Years)	Swiss Confederation	Both	All ages	Cardiovascular diseases	Rate	2021	3347.021
DALYs (Disability-Adjusted Life Years)	Kingdom of Bahrain	Both	All ages	Cardiovascular diseases	Rate	2021	2587.169
DALYs (Disability-Adjusted Life Years)	Republic of Guatemala	Both	All ages	Cardiovascular diseases	Rate	2021	2192.977
DALYs (Disability-Adjusted Life Years)	Commonwealth of the Bahamas	Both	All ages	Cardiovascular diseases	Rate	2021	5386.514
DALYs (Disability-Adjusted Life Years)	Republic of Suriname	Both	All ages	Cardiovascular diseases	Rate	2021	5601.987
DALYs (Disability-Adjusted Life Years)	Burkina Faso	Both	All ages	Cardiovascular diseases	Rate	2021	2704.828
DALYs (Disability-Adjusted Life Years)	Republic of the Niger	Both	All ages	Cardiovascular diseases	Rate	2021	2102.419
DALYs (Disability-Adjusted Life Years)	Kingdom of Sweden	Both	All ages	Cardiovascular diseases	Rate	2021	4659.966
DALYs (Disability-Adjusted Life Years)	Islamic Republic of Iran	Both	All ages	Cardiovascular diseases	Rate	2021	4367.442
DALYs (Disability-Adjusted Life Years)	Republic of Indonesia	Both	All ages	Cardiovascular diseases	Rate	2021	7281.413

DALYs (Disability-Adjusted Life Years)	Republic of Honduras	Both	All ages	Cardiovascular diseases	Rate	2021	3963.082
DALYs (Disability-Adjusted Life Years)	Arab Republic of Egypt	Both	All ages	Cardiovascular diseases	Rate	2021	7023.74
DALYs (Disability-Adjusted Life Years)	Hellenic Republic	Both	All ages	Cardiovascular diseases	Rate	2021	7207.391
DALYs (Disability-Adjusted Life Years)	Islamic Republic of Pakistan	Both	All ages	Cardiovascular diseases	Rate	2021	4451.399
DALYs (Disability-Adjusted Life Years)	Republic of Djibouti	Both	All ages	Cardiovascular diseases	Rate	2021	3122.488
DALYs (Disability-Adjusted Life Years)	Georgia	Both	All ages	Cardiovascular diseases	Rate	2021	11783.31
DALYs (Disability-Adjusted Life Years)	Saint Vincent and the Grenadines	Both	All ages	Cardiovascular diseases	Rate	2021	6526.583
DALYs (Disability-Adjusted Life Years)	Czech Republic	Both	All ages	Cardiovascular diseases	Rate	2021	7650.706
DALYs (Disability-Adjusted Life Years)	Republic of South Sudan	Both	All ages	Cardiovascular diseases	Rate	2021	2809.278
DALYs (Disability-Adjusted Life Years)	United Mexican States	Both	All ages	Cardiovascular diseases	Rate	2021	3246.218
DALYs (Disability-Adjusted Life Years)	Republic of Nauru	Both	All ages	Cardiovascular diseases	Rate	2021	11518.67
DALYs (Disability-Adjusted Life Years)	Republic of Tunisia	Both	All ages	Cardiovascular diseases	Rate	2021	6067.4
DALYs (Disability-Adjusted Life Years)	Kyrgyz Republic	Both	All ages	Cardiovascular diseases	Rate	2021	5379.574
DALYs (Disability-Adjusted Life Years)	Syrian Arab Republic	Both	All ages	Cardiovascular diseases	Rate	2021	8896.469
DALYs (Disability-Adjusted Life Years)	Malaysia	Both	All ages	Cardiovascular diseases	Rate	2021	5272.834
DALYs (Disability-Adjusted Life Years)	State of Eritrea	Both	All ages	Cardiovascular diseases	Rate	2021	3297.28
DALYs (Disability-Adjusted Life Years)	Australia	Both	All ages	Cardiovascular diseases	Rate	2021	3210.472
DALYs (Disability-Adjusted Life Years)	Republic of Nicaragua	Both	All ages	Cardiovascular diseases	Rate	2021	2038.954
DALYs (Disability-Adjusted Life Years)	Republic of Cabo Verde	Both	All ages	Cardiovascular diseases	Rate	2021	4452.921
DALYs (Disability-Adjusted Life Years)	Republic of Iceland	Both	All ages	Cardiovascular diseases	Rate	2021	3206.534
DALYs (Disability-Adjusted Life Years)	Northern Mariana Islands	Both	All ages	Cardiovascular diseases	Rate	2021	6852.862
DALYs (Disability-Adjusted Life Years)	Republic of Iraq	Both	All ages	Cardiovascular diseases	Rate	2021	5246.275
DALYs (Disability-Adjusted Life Years)	United Kingdom of Great Britain and Northern Ireland	Both	All ages	Cardiovascular diseases	Rate	2021	4097.34
DALYs (Disability-Adjusted Life Years)	Republic of Zambia	Both	All ages	Cardiovascular diseases	Rate	2021	2848.169

DALYs (Disability-Adjusted Life Years)	Federal Democratic Republic of Ethiopia	Both	All ages	Cardiovascular diseases	Rate	2021	1812.125
DALYs (Disability-Adjusted Life Years)	American Samoa	Both	All ages	Cardiovascular diseases	Rate	2021	7592.247
DALYs (Disability-Adjusted Life Years)	Barbados	Both	All ages	Cardiovascular diseases	Rate	2021	6019.627
DALYs (Disability-Adjusted Life Years)	Republic of Latvia	Both	All ages	Cardiovascular diseases	Rate	2021	13715.75
DALYs (Disability-Adjusted Life Years)	Republic of Chad	Both	All ages	Cardiovascular diseases	Rate	2021	2846.691
DALYs (Disability-Adjusted Life Years)	Federated States of Micronesia	Both	All ages	Cardiovascular diseases	Rate	2021	9896.755
DALYs (Disability-Adjusted Life Years)	Republic of Trinidad and Tobago	Both	All ages	Cardiovascular diseases	Rate	2021	6433.451
DALYs (Disability-Adjusted Life Years)	North Macedonia	Both	All ages	Cardiovascular diseases	Rate	2021	11457.37
DALYs (Disability-Adjusted Life Years)	Republic of Tajikistan	Both	All ages	Cardiovascular diseases	Rate	2021	4123.773
DALYs (Disability-Adjusted Life Years)	Principality of Andorra	Both	All ages	Cardiovascular diseases	Rate	2021	3092.972
DALYs (Disability-Adjusted Life Years)	Republic of Maldives	Both	All ages	Cardiovascular diseases	Rate	2021	2502.488
DALYs (Disability-Adjusted Life Years)	Democratic Republic of Sao Tome and Principe	Both	All ages	Cardiovascular diseases	Rate	2021	3450.585
DALYs (Disability-Adjusted Life Years)	Republic of Turkey	Both	All ages	Cardiovascular diseases	Rate	2021	4915.162
DALYs (Disability-Adjusted Life Years)	Mongolia	Both	All ages	Cardiovascular diseases	Rate	2021	5435.787
DALYs (Disability-Adjusted Life Years)	Montenegro	Both	All ages	Cardiovascular diseases	Rate	2021	12372.44
DALYs (Disability-Adjusted Life Years)	Plurinational State of Bolivia	Both	All ages	Cardiovascular diseases	Rate	2021	2940.445
DALYs (Disability-Adjusted Life Years)	Republic of CÃ´te d'Ivoire	Both	All ages	Cardiovascular diseases	Rate	2021	3143.643
DALYs (Disability-Adjusted Life Years)	Republic of Senegal	Both	All ages	Cardiovascular diseases	Rate	2021	3590.925
DALYs (Disability-Adjusted Life Years)	Republic of Poland	Both	All ages	Cardiovascular diseases	Rate	2021	7725.623
DALYs (Disability-Adjusted Life Years)	Turkmenistan	Both	All ages	Cardiovascular diseases	Rate	2021	9001.817
DALYs (Disability-Adjusted Life Years)	Independent State of Papua New Guinea	Both	All ages	Cardiovascular diseases	Rate	2021	4974.911
DALYs (Disability-Adjusted Life Years)	Argentine Republic	Both	All ages	Cardiovascular diseases	Rate	2021	3951.271
DALYs (Disability-Adjusted Life Years)	Commonwealth of Dominica	Both	All ages	Cardiovascular diseases	Rate	2021	6623.883
DALYs (Disability-Adjusted Life Years)	Republic of Madagascar	Both	All ages	Cardiovascular diseases	Rate	2021	4253.786

DALYs (Disability-Adjusted Life Years)	Republic of the Union of Myanmar	Both	All ages	Cardiovascular diseases	Rate	2021	6714.954
DALYs (Disability-Adjusted Life Years)	Republic of Moldova	Both	All ages	Cardiovascular diseases	Rate	2021	12102.5
DALYs (Disability-Adjusted Life Years)	Kingdom of Lesotho	Both	All ages	Cardiovascular diseases	Rate	2021	5160.637
DALYs (Disability-Adjusted Life Years)	Solomon Islands	Both	All ages	Cardiovascular diseases	Rate	2021	7008.661
DALYs (Disability-Adjusted Life Years)	State of Israel	Both	All ages	Cardiovascular diseases	Rate	2021	2003.559
DALYs (Disability-Adjusted Life Years)	Ukraine	Both	All ages	Cardiovascular diseases	Rate	2021	17537.37
DALYs (Disability-Adjusted Life Years)	Republic of Austria	Both	All ages	Cardiovascular diseases	Rate	2021	5201.149
DALYs (Disability-Adjusted Life Years)	State of Kuwait	Both	All ages	Cardiovascular diseases	Rate	2021	2531.7
DALYs (Disability-Adjusted Life Years)	Republic of Italy	Both	All ages	Cardiovascular diseases	Rate	2021	5302.212
DALYs (Disability-Adjusted Life Years)	Central African Republic	Both	All ages	Cardiovascular diseases	Rate	2021	4338.353
DALYs (Disability-Adjusted Life Years)	Republic of Yemen	Both	All ages	Cardiovascular diseases	Rate	2021	4715.707
DALYs (Disability-Adjusted Life Years)	Puerto Rico	Both	All ages	Cardiovascular diseases	Rate	2021	4610.119
DALYs (Disability-Adjusted Life Years)	Republic of Chile	Both	All ages	Cardiovascular diseases	Rate	2021	2962.953
DALYs (Disability-Adjusted Life Years)	Republic of Cuba	Both	All ages	Cardiovascular diseases	Rate	2021	7008.012
DALYs (Disability-Adjusted Life Years)	Togolese Republic	Both	All ages	Cardiovascular diseases	Rate	2021	3657.326
DALYs (Disability-Adjusted Life Years)	Republic of Palau	Both	All ages	Cardiovascular diseases	Rate	2021	11509.82
DALYs (Disability-Adjusted Life Years)	Kingdom of Belgium	Both	All ages	Cardiovascular diseases	Rate	2021	3798.799
DALYs (Disability-Adjusted Life Years)	Dominican Republic	Both	All ages	Cardiovascular diseases	Rate	2021	5262.688
DALYs (Disability-Adjusted Life Years)	Romania	Both	All ages	Cardiovascular diseases	Rate	2021	13332.88
DALYs (Disability-Adjusted Life Years)	Eastern Republic of Uruguay	Both	All ages	Cardiovascular diseases	Rate	2021	5193.297
DALYs (Disability-Adjusted Life Years)	Republic of Botswana	Both	All ages	Cardiovascular diseases	Rate	2021	3197.488
DALYs (Disability-Adjusted Life Years)	Russian Federation	Both	All ages	Cardiovascular diseases	Rate	2021	13074.67
DALYs (Disability-Adjusted Life Years)	Bermuda	Both	All ages	Cardiovascular diseases	Rate	2021	6153.439
DALYs (Disability-Adjusted Life Years)	Republic of the Gambia	Both	All ages	Cardiovascular diseases	Rate	2021	3607.535

DALYs (Disability-Adjusted Life Years)	Grand Duchy of Luxembourg	Both	All ages	Cardiovascular diseases	Rate	2021	3341.818
DALYs (Disability-Adjusted Life Years)	Republic of Peru	Both	All ages	Cardiovascular diseases	Rate	2021	2033.674
DALYs (Disability-Adjusted Life Years)	State of Libya	Both	All ages	Cardiovascular diseases	Rate	2021	5536.071
DALYs (Disability-Adjusted Life Years)	Grenada	Both	All ages	Cardiovascular diseases	Rate	2021	5825.057
DALYs (Disability-Adjusted Life Years)	Bolivarian Republic of Venezuela	Both	All ages	Cardiovascular diseases	Rate	2021	5488.902
DALYs (Disability-Adjusted Life Years)	Independent State of Samoa	Both	All ages	Cardiovascular diseases	Rate	2021	6880.825
DALYs (Disability-Adjusted Life Years)	Republic of South Africa	Both	All ages	Cardiovascular diseases	Rate	2021	4294.232
DALYs (Disability-Adjusted Life Years)	Republic of Sierra Leone	Both	All ages	Cardiovascular diseases	Rate	2021	3661.987
DALYs (Disability-Adjusted Life Years)	Republic of the Philippines	Both	All ages	Cardiovascular diseases	Rate	2021	5463.327
DALYs (Disability-Adjusted Life Years)	Republic of Paraguay	Both	All ages	Cardiovascular diseases	Rate	2021	3377.372
DALYs (Disability-Adjusted Life Years)	Republic of Malta	Both	All ages	Cardiovascular diseases	Rate	2021	4881.821
DALYs (Disability-Adjusted Life Years)	Republic of Vanuatu	Both	All ages	Cardiovascular diseases	Rate	2021	8168
DALYs (Disability-Adjusted Life Years)	Saint Kitts and Nevis	Both	All ages	Cardiovascular diseases	Rate	2021	5869.439
DALYs (Disability-Adjusted Life Years)	Republic of Mauritius	Both	All ages	Cardiovascular diseases	Rate	2021	6503.669
DALYs (Disability-Adjusted Life Years)	Greenland	Both	All ages	Cardiovascular diseases	Rate	2021	4550.183
DALYs (Disability-Adjusted Life Years)	Kingdom of Morocco	Both	All ages	Cardiovascular diseases	Rate	2021	8107.563
DALYs (Disability-Adjusted Life Years)	Democratic Republic of the Congo	Both	All ages	Cardiovascular diseases	Rate	2021	3023.136
DALYs (Disability-Adjusted Life Years)	Federative Republic of Brazil	Both	All ages	Cardiovascular diseases	Rate	2021	4115.689
DALYs (Disability-Adjusted Life Years)	People's Republic of China	Both	All ages	Cardiovascular diseases	Rate	2021	7043.333
DALYs (Disability-Adjusted Life Years)	Republic of Guinea-Bissau	Both	All ages	Cardiovascular diseases	Rate	2021	4117.494
DALYs (Disability-Adjusted Life Years)	Republic of Guinea	Both	All ages	Cardiovascular diseases	Rate	2021	3416.827
DALYs (Disability-Adjusted Life Years)	Islamic Republic of Afghanistan	Both	All ages	Cardiovascular diseases	Rate	2021	4653.779
DALYs (Disability-Adjusted Life Years)	Kingdom of Eswatini	Both	All ages	Cardiovascular diseases	Rate	2021	3991.484
DALYs (Disability-Adjusted Life Years)	Republic of Mozambique	Both	All ages	Cardiovascular diseases	Rate	2021	3184.387

DALYs (Disability-Adjusted Life Years)	Republic of Equatorial Guinea	Both	All ages	Cardiovascular diseases	Rate	2021	2379.031
DALYs (Disability-Adjusted Life Years)	Kingdom of Thailand	Both	All ages	Cardiovascular diseases	Rate	2021	4777.727
DALYs (Disability-Adjusted Life Years)	Tokelau	Both	All ages	Cardiovascular diseases	Rate	2021	9625.285
DALYs (Disability-Adjusted Life Years)	Republic of Sudan	Both	All ages	Cardiovascular diseases	Rate	2021	4702.267
Incidence	Republic of the Philippines	Both	All ages	Cardiovascular diseases	Rate	2021	449.5703
Incidence	Kingdom of Thailand	Both	All ages	Cardiovascular diseases	Rate	2021	838.2877
Incidence	Republic of Indonesia	Both	All ages	Cardiovascular diseases	Rate	2021	624.2695
Incidence	People's Republic of China	Both	All ages	Cardiovascular diseases	Rate	2021	1127.338
Incidence	Federated States of Micronesia	Both	All ages	Cardiovascular diseases	Rate	2021	660.7194
Incidence	Malaysia	Both	All ages	Cardiovascular diseases	Rate	2021	661.3119
Incidence	Socialist Republic of Viet Nam	Both	All ages	Cardiovascular diseases	Rate	2021	643.5876
Incidence	Republic of the Union of Myanmar	Both	All ages	Cardiovascular diseases	Rate	2021	598.1689
Incidence	Taiwan (Province of China)	Both	All ages	Cardiovascular diseases	Rate	2021	1184.294
Incidence	Republic of Azerbaijan	Both	All ages	Cardiovascular diseases	Rate	2021	1095.471
Incidence	Independent State of Samoa	Both	All ages	Cardiovascular diseases	Rate	2021	633.4765
Incidence	Kingdom of Cambodia	Both	All ages	Cardiovascular diseases	Rate	2021	503.9896
Incidence	Republic of Uzbekistan	Both	All ages	Cardiovascular diseases	Rate	2021	1123.345
Incidence	Republic of Kazakhstan	Both	All ages	Cardiovascular diseases	Rate	2021	908.1434
Incidence	Democratic Socialist Republic of Sri Lanka	Both	All ages	Cardiovascular diseases	Rate	2021	716.7975
Incidence	Lao People's Democratic Republic	Both	All ages	Cardiovascular diseases	Rate	2021	473.7743
Incidence	Republic of Fiji	Both	All ages	Cardiovascular diseases	Rate	2021	720.0629
Incidence	Kingdom of Tonga	Both	All ages	Cardiovascular diseases	Rate	2021	677.0924
Incidence	Republic of the Marshall Islands	Both	All ages	Cardiovascular diseases	Rate	2021	573.1617
Incidence	Hungary	Both	All ages	Cardiovascular diseases	Rate	2021	1497.737

Incidence	Republic of Albania	Both	All ages	Cardiovascular diseases	Rate	2021	1132.731
Incidence	Independent State of Papua New Guinea	Both	All ages	Cardiovascular diseases	Rate	2021	399.8156
Incidence	Mongolia	Both	All ages	Cardiovascular diseases	Rate	2021	797.3719
Incidence	Democratic People's Republic of Korea	Both	All ages	Cardiovascular diseases	Rate	2021	998.8098
Incidence	Republic of Maldives	Both	All ages	Cardiovascular diseases	Rate	2021	424.7645
Incidence	Republic of Armenia	Both	All ages	Cardiovascular diseases	Rate	2021	1360.903
Incidence	Republic of Bulgaria	Both	All ages	Cardiovascular diseases	Rate	2021	1715.12
Incidence	Montenegro	Both	All ages	Cardiovascular diseases	Rate	2021	1223.258
Incidence	Democratic Republic of Timor-Leste	Both	All ages	Cardiovascular diseases	Rate	2021	429.169
Incidence	Turkmenistan	Both	All ages	Cardiovascular diseases	Rate	2021	901.7681
Incidence	Solomon Islands	Both	All ages	Cardiovascular diseases	Rate	2021	575.0378
Incidence	Georgia	Both	All ages	Cardiovascular diseases	Rate	2021	1769.455
Incidence	Republic of Belarus	Both	All ages	Cardiovascular diseases	Rate	2021	1692.915
Incidence	Kyrgyz Republic	Both	All ages	Cardiovascular diseases	Rate	2021	693.6774
Incidence	Romania	Both	All ages	Cardiovascular diseases	Rate	2021	1468.51
Incidence	Republic of Kiribati	Both	All ages	Cardiovascular diseases	Rate	2021	642.6856
Incidence	Slovak Republic	Both	All ages	Cardiovascular diseases	Rate	2021	1247.554
Incidence	Republic of Vanuatu	Both	All ages	Cardiovascular diseases	Rate	2021	583.999
Incidence	Czech Republic	Both	All ages	Cardiovascular diseases	Rate	2021	1511.335
Incidence	Republic of Latvia	Both	All ages	Cardiovascular diseases	Rate	2021	1646.868
Incidence	Bosnia and Herzegovina	Both	All ages	Cardiovascular diseases	Rate	2021	1349.751
Incidence	Brunei Darussalam	Both	All ages	Cardiovascular diseases	Rate	2021	392.3751
Incidence	North Macedonia	Both	All ages	Cardiovascular diseases	Rate	2021	1217.953
Incidence	Republic of Korea	Both	All ages	Cardiovascular diseases	Rate	2021	738.1897

Incidence	French Republic	Both	All ages	Cardiovascular diseases	Rate	2021	1079.756
Incidence	Republic of Moldova	Both	All ages	Cardiovascular diseases	Rate	2021	1431.635
Incidence	Republic of Croatia	Both	All ages	Cardiovascular diseases	Rate	2021	1257.575
Incidence	Republic of Austria	Both	All ages	Cardiovascular diseases	Rate	2021	1358.565
Incidence	Republic of Tajikistan	Both	All ages	Cardiovascular diseases	Rate	2021	669.6886
Incidence	Ukraine	Both	All ages	Cardiovascular diseases	Rate	2021	2054.277
Incidence	Hellenic Republic	Both	All ages	Cardiovascular diseases	Rate	2021	1207.846
Incidence	Ireland	Both	All ages	Cardiovascular diseases	Rate	2021	799.6878
Incidence	Republic of Cyprus	Both	All ages	Cardiovascular diseases	Rate	2021	724.7823
Incidence	Kingdom of the Netherlands	Both	All ages	Cardiovascular diseases	Rate	2021	1076.388
Incidence	Republic of Poland	Both	All ages	Cardiovascular diseases	Rate	2021	1010.286
Incidence	Republic of Serbia	Both	All ages	Cardiovascular diseases	Rate	2021	1624.022
Incidence	Republic of Finland	Both	All ages	Cardiovascular diseases	Rate	2021	1434.388
Incidence	Republic of Estonia	Both	All ages	Cardiovascular diseases	Rate	2021	1814.778
Incidence	Republic of Slovenia	Both	All ages	Cardiovascular diseases	Rate	2021	1349.395
Incidence	New Zealand	Both	All ages	Cardiovascular diseases	Rate	2021	963.5196
Incidence	Japan	Both	All ages	Cardiovascular diseases	Rate	2021	1096.085
Incidence	Portuguese Republic	Both	All ages	Cardiovascular diseases	Rate	2021	927.916
Incidence	Republic of Lithuania	Both	All ages	Cardiovascular diseases	Rate	2021	1728.887
Incidence	Republic of Iceland	Both	All ages	Cardiovascular diseases	Rate	2021	899.085
Incidence	Republic of Italy	Both	All ages	Cardiovascular diseases	Rate	2021	1355.053
Incidence	Federal Republic of Germany	Both	All ages	Cardiovascular diseases	Rate	2021	1444.388
Incidence	Principality of Andorra	Both	All ages	Cardiovascular diseases	Rate	2021	884.8376
Incidence	Republic of Chile	Both	All ages	Cardiovascular diseases	Rate	2021	594.7428

Incidence	Republic of Singapore	Both	All ages	Cardiovascular diseases	Rate	2021	632.9986
Incidence	Republic of Malta	Both	All ages	Cardiovascular diseases	Rate	2021	967.7376
Incidence	Russian Federation	Both	All ages	Cardiovascular diseases	Rate	2021	1876.779
Incidence	Kingdom of Belgium	Both	All ages	Cardiovascular diseases	Rate	2021	1074.135
Incidence	Australia	Both	All ages	Cardiovascular diseases	Rate	2021	922.3375
Incidence	Kingdom of Sweden	Both	All ages	Cardiovascular diseases	Rate	2021	1383.566
Incidence	Antigua and Barbuda	Both	All ages	Cardiovascular diseases	Rate	2021	847.7341
Incidence	Kingdom of Denmark	Both	All ages	Cardiovascular diseases	Rate	2021	1064.933
Incidence	United Kingdom of Great Britain and Northern Ireland	Both	All ages	Cardiovascular diseases	Rate	2021	955.8173
Incidence	Grenada	Both	All ages	Cardiovascular diseases	Rate	2021	842.6836
Incidence	Barbados	Both	All ages	Cardiovascular diseases	Rate	2021	1204.696
Incidence	United States of America	Both	All ages	Cardiovascular diseases	Rate	2021	1156.34
Incidence	Kingdom of Spain	Both	All ages	Cardiovascular diseases	Rate	2021	1044.947
Incidence	State of Israel	Both	All ages	Cardiovascular diseases	Rate	2021	681.3118
Incidence	Republic of Haiti	Both	All ages	Cardiovascular diseases	Rate	2021	522.1013
Incidence	Kingdom of Norway	Both	All ages	Cardiovascular diseases	Rate	2021	1114.901
Incidence	Republic of Cuba	Both	All ages	Cardiovascular diseases	Rate	2021	1100.856
Incidence	Plurinational State of Bolivia	Both	All ages	Cardiovascular diseases	Rate	2021	465.1662
Incidence	Argentine Republic	Both	All ages	Cardiovascular diseases	Rate	2021	751.9393
Incidence	Republic of Guatemala	Both	All ages	Cardiovascular diseases	Rate	2021	476.4195
Incidence	Saint Lucia	Both	All ages	Cardiovascular diseases	Rate	2021	1005.763
Incidence	Grand Duchy of Luxembourg	Both	All ages	Cardiovascular diseases	Rate	2021	834.5511
Incidence	Republic of Peru	Both	All ages	Cardiovascular diseases	Rate	2021	499.1491
Incidence	Republic of Suriname	Both	All ages	Cardiovascular diseases	Rate	2021	853.374

Incidence	Eastern Republic of Uruguay	Both	All ages	Cardiovascular diseases	Rate	2021	902.1914
Incidence	Swiss Confederation	Both	All ages	Cardiovascular diseases	Rate	2021	973.417
Incidence	United Mexican States	Both	All ages	Cardiovascular diseases	Rate	2021	617.6379
Incidence	Republic of Colombia	Both	All ages	Cardiovascular diseases	Rate	2021	613.1638
Incidence	Commonwealth of the Bahamas	Both	All ages	Cardiovascular diseases	Rate	2021	780.8541
Incidence	Dominican Republic	Both	All ages	Cardiovascular diseases	Rate	2021	707.9291
Incidence	Jamaica	Both	All ages	Cardiovascular diseases	Rate	2021	834.1461
Incidence	Canada	Both	All ages	Cardiovascular diseases	Rate	2021	1191.567
Incidence	Republic of Panama	Both	All ages	Cardiovascular diseases	Rate	2021	670.5138
Incidence	Republic of Guyana	Both	All ages	Cardiovascular diseases	Rate	2021	709.599
Incidence	Republic of Ecuador	Both	All ages	Cardiovascular diseases	Rate	2021	530.3719
Incidence	Republic of El Salvador	Both	All ages	Cardiovascular diseases	Rate	2021	621.9462
Incidence	Belize	Both	All ages	Cardiovascular diseases	Rate	2021	541.3953
Incidence	Saint Vincent and the Grenadines	Both	All ages	Cardiovascular diseases	Rate	2021	890.8044
Incidence	People's Democratic Republic of Algeria	Both	All ages	Cardiovascular diseases	Rate	2021	968.4065
Incidence	Commonwealth of Dominica	Both	All ages	Cardiovascular diseases	Rate	2021	889.4186
Incidence	Republic of Honduras	Both	All ages	Cardiovascular diseases	Rate	2021	494.6848
Incidence	Hashemite Kingdom of Jordan	Both	All ages	Cardiovascular diseases	Rate	2021	779.9049
Incidence	Arab Republic of Egypt	Both	All ages	Cardiovascular diseases	Rate	2021	874.9741
Incidence	Lebanese Republic	Both	All ages	Cardiovascular diseases	Rate	2021	1344.937
Incidence	Republic of Paraguay	Both	All ages	Cardiovascular diseases	Rate	2021	478.1027
Incidence	Republic of Nicaragua	Both	All ages	Cardiovascular diseases	Rate	2021	515.7622
Incidence	Republic of Costa Rica	Both	All ages	Cardiovascular diseases	Rate	2021	782.6572
Incidence	Republic of Trinidad and Tobago	Both	All ages	Cardiovascular diseases	Rate	2021	1042.673

Incidence	Republic of Iraq	Both	All ages	Cardiovascular diseases	Rate	2021	861.1184
Incidence	Syrian Arab Republic	Both	All ages	Cardiovascular diseases	Rate	2021	1321.608
Incidence	Bolivarian Republic of Venezuela	Both	All ages	Cardiovascular diseases	Rate	2021	693.7093
Incidence	Kingdom of Morocco	Both	All ages	Cardiovascular diseases	Rate	2021	1232.722
Incidence	Federative Republic of Brazil	Both	All ages	Cardiovascular diseases	Rate	2021	600.7915
Incidence	Kingdom of Bahrain	Both	All ages	Cardiovascular diseases	Rate	2021	837.142
Incidence	State of Kuwait	Both	All ages	Cardiovascular diseases	Rate	2021	920.3779
Incidence	Islamic Republic of Afghanistan	Both	All ages	Cardiovascular diseases	Rate	2021	491.5339
Incidence	Republic of Turkey	Both	All ages	Cardiovascular diseases	Rate	2021	1043.083
Incidence	Sultanate of Oman	Both	All ages	Cardiovascular diseases	Rate	2021	611.008
Incidence	Republic of Yemen	Both	All ages	Cardiovascular diseases	Rate	2021	599.8867
Incidence	Kingdom of Bhutan	Both	All ages	Cardiovascular diseases	Rate	2021	725.5439
Incidence	Kingdom of Saudi Arabia	Both	All ages	Cardiovascular diseases	Rate	2021	707.9718
Incidence	Islamic Republic of Iran	Both	All ages	Cardiovascular diseases	Rate	2021	1081.966
Incidence	State of Libya	Both	All ages	Cardiovascular diseases	Rate	2021	980.0623
Incidence	Republic of Tunisia	Both	All ages	Cardiovascular diseases	Rate	2021	1256.559
Incidence	Palestine	Both	All ages	Cardiovascular diseases	Rate	2021	667.6258
Incidence	Federal Democratic Republic of Nepal	Both	All ages	Cardiovascular diseases	Rate	2021	629.2559
Incidence	Central African Republic	Both	All ages	Cardiovascular diseases	Rate	2021	444.0396
Incidence	Democratic Republic of the Congo	Both	All ages	Cardiovascular diseases	Rate	2021	418.3404
Incidence	People's Republic of Bangladesh	Both	All ages	Cardiovascular diseases	Rate	2021	707.7526
Incidence	State of Qatar	Both	All ages	Cardiovascular diseases	Rate	2021	552.0331
Incidence	Union of the Comoros	Both	All ages	Cardiovascular diseases	Rate	2021	538.1251
Incidence	United Arab Emirates	Both	All ages	Cardiovascular diseases	Rate	2021	891.6188

Incidence	Republic of Angola	Both	All ages	Cardiovascular diseases	Rate	2021	389.7788
Incidence	Republic of India	Both	All ages	Cardiovascular diseases	Rate	2021	761.9794
Incidence	Gabonese Republic	Both	All ages	Cardiovascular diseases	Rate	2021	514.1475
Incidence	State of Eritrea	Both	All ages	Cardiovascular diseases	Rate	2021	427.0169
Incidence	Islamic Republic of Pakistan	Both	All ages	Cardiovascular diseases	Rate	2021	604.3963
Incidence	Republic of the Congo	Both	All ages	Cardiovascular diseases	Rate	2021	499.7427
Incidence	Republic of Burundi	Both	All ages	Cardiovascular diseases	Rate	2021	383.108
Incidence	Republic of Kenya	Both	All ages	Cardiovascular diseases	Rate	2021	425.1763
Incidence	Republic of Rwanda	Both	All ages	Cardiovascular diseases	Rate	2021	423.7195
Incidence	Republic of Equatorial Guinea	Both	All ages	Cardiovascular diseases	Rate	2021	378.1704
Incidence	Republic of Malawi	Both	All ages	Cardiovascular diseases	Rate	2021	410.0925
Incidence	Republic of Djibouti	Both	All ages	Cardiovascular diseases	Rate	2021	467.2483
Incidence	Federal Republic of Somalia	Both	All ages	Cardiovascular diseases	Rate	2021	351.2247
Incidence	Republic of Botswana	Both	All ages	Cardiovascular diseases	Rate	2021	563.1207
Incidence	Republic of Uganda	Both	All ages	Cardiovascular diseases	Rate	2021	355.0751
Incidence	Federal Democratic Republic of Ethiopia	Both	All ages	Cardiovascular diseases	Rate	2021	363.0064
Incidence	Republic of Mozambique	Both	All ages	Cardiovascular diseases	Rate	2021	414.4701
Incidence	Republic of Madagascar	Both	All ages	Cardiovascular diseases	Rate	2021	433.0014
Incidence	Republic of Namibia	Both	All ages	Cardiovascular diseases	Rate	2021	491.0656
Incidence	Republic of Seychelles	Both	All ages	Cardiovascular diseases	Rate	2021	678.559
Incidence	Kingdom of Eswatini	Both	All ages	Cardiovascular diseases	Rate	2021	453.5997
Incidence	Republic of Mauritius	Both	All ages	Cardiovascular diseases	Rate	2021	845.065
Incidence	Republic of Cameroon	Both	All ages	Cardiovascular diseases	Rate	2021	366.8795
Incidence	United Republic of Tanzania	Both	All ages	Cardiovascular diseases	Rate	2021	430.6795

Incidence	Republic of Chad	Both	All ages	Cardiovascular diseases	Rate	2021	337.0495
Incidence	Kingdom of Lesotho	Both	All ages	Cardiovascular diseases	Rate	2021	515.4615
Incidence	Republic of Guinea-Bissau	Both	All ages	Cardiovascular diseases	Rate	2021	373.2418
Incidence	Republic of South Africa	Both	All ages	Cardiovascular diseases	Rate	2021	672.6158
Incidence	Republic of Mali	Both	All ages	Cardiovascular diseases	Rate	2021	342.6989
Incidence	Republic of Zambia	Both	All ages	Cardiovascular diseases	Rate	2021	386.8507
Incidence	Burkina Faso	Both	All ages	Cardiovascular diseases	Rate	2021	347.991
Incidence	Republic of the Gambia	Both	All ages	Cardiovascular diseases	Rate	2021	411.264
Incidence	Republic of Guinea	Both	All ages	Cardiovascular diseases	Rate	2021	387.8953
Incidence	Republic of the Niger	Both	All ages	Cardiovascular diseases	Rate	2021	322.1378
Incidence	Republic of Cabo Verde	Both	All ages	Cardiovascular diseases	Rate	2021	619.8405
Incidence	Republic of Zimbabwe	Both	All ages	Cardiovascular diseases	Rate	2021	430.093
Incidence	Togolese Republic	Both	All ages	Cardiovascular diseases	Rate	2021	420.4928
Incidence	Republic of Liberia	Both	All ages	Cardiovascular diseases	Rate	2021	381.8774
Incidence	Republic of CÃ´te d'Ivoire	Both	All ages	Cardiovascular diseases	Rate	2021	389.6933
Incidence	Republic of Benin	Both	All ages	Cardiovascular diseases	Rate	2021	364.8948
Incidence	Democratic Republic of Sao Tome and Principe	Both	All ages	Cardiovascular diseases	Rate	2021	480.2327
Incidence	Republic of Ghana	Both	All ages	Cardiovascular diseases	Rate	2021	470.8292
Incidence	American Samoa	Both	All ages	Cardiovascular diseases	Rate	2021	809.9895
Incidence	Islamic Republic of Mauritania	Both	All ages	Cardiovascular diseases	Rate	2021	434.0653
Incidence	Republic of Sierra Leone	Both	All ages	Cardiovascular diseases	Rate	2021	418.6625
Incidence	Cook Islands	Both	All ages	Cardiovascular diseases	Rate	2021	1071.748
Incidence	Principality of Monaco	Both	All ages	Cardiovascular diseases	Rate	2021	1249.997
Incidence	Puerto Rico	Both	All ages	Cardiovascular diseases	Rate	2021	1279.543

Incidence	Guam	Both	All ages	Cardiovascular diseases	Rate	2021	1018.903
Incidence	Federal Republic of Nigeria	Both	All ages	Cardiovascular diseases	Rate	2021	382.3907
Incidence	Republic of Niue	Both	All ages	Cardiovascular diseases	Rate	2021	1032.123
Incidence	Republic of San Marino	Both	All ages	Cardiovascular diseases	Rate	2021	1113.602
Incidence	Republic of Senegal	Both	All ages	Cardiovascular diseases	Rate	2021	443.8088
Incidence	Bermuda	Both	All ages	Cardiovascular diseases	Rate	2021	1341.009
Incidence	Tuvalu	Both	All ages	Cardiovascular diseases	Rate	2021	669.0046
Incidence	Republic of Palau	Both	All ages	Cardiovascular diseases	Rate	2021	962.8858
Incidence	Greenland	Both	All ages	Cardiovascular diseases	Rate	2021	898.5913
Incidence	Republic of Nauru	Both	All ages	Cardiovascular diseases	Rate	2021	465.5749
Incidence	Saint Kitts and Nevis	Both	All ages	Cardiovascular diseases	Rate	2021	818.2528
Incidence	Republic of South Sudan	Both	All ages	Cardiovascular diseases	Rate	2021	389.6949
Incidence	Tokelau	Both	All ages	Cardiovascular diseases	Rate	2021	815.8795
Incidence	Northern Mariana Islands	Both	All ages	Cardiovascular diseases	Rate	2021	808.7403
Incidence	United States Virgin Islands	Both	All ages	Cardiovascular diseases	Rate	2021	1380.42
Incidence	Republic of Sudan	Both	All ages	Cardiovascular diseases	Rate	2021	642.36

S2B: Country Neoplasm incidence, mortality and DALYS

measure	Country	sex	age	cause	metric	year	Rate
Incidence	People's Republic of China	Both	All ages	Neoplasms	Rate	2021	960
Incidence	Taiwan (Province of China)	Both	All ages	Neoplasms	Rate	2021	1007
Incidence	Malaysia	Both	All ages	Neoplasms	Rate	2021	431
Incidence	Socialist Republic of Viet Nam	Both	All ages	Neoplasms	Rate	2021	438
Incidence	Kingdom of Cambodia	Both	All ages	Neoplasms	Rate	2021	379

Incidence	Republic of the Union of Myanmar	Both	All ages	Neoplasms	Rate	2021	382
Incidence	Republic of Fiji	Both	All ages	Neoplasms	Rate	2021	450
Incidence	Kingdom of Tonga	Both	All ages	Neoplasms	Rate	2021	457
Incidence	Lao People's Democratic Republic	Both	All ages	Neoplasms	Rate	2021	341
Incidence	Republic of Kazakhstan	Both	All ages	Neoplasms	Rate	2021	747
Incidence	Democratic Socialist Republic of Sri Lanka	Both	All ages	Neoplasms	Rate	2021	418
Incidence	Democratic Republic of Timor-Leste	Both	All ages	Neoplasms	Rate	2021	299
Incidence	Republic of the Marshall Islands	Both	All ages	Neoplasms	Rate	2021	423
Incidence	Democratic People's Republic of Korea	Both	All ages	Neoplasms	Rate	2021	694
Incidence	Republic of Armenia	Both	All ages	Neoplasms	Rate	2021	835
Incidence	Independent State of Papua New Guinea	Both	All ages	Neoplasms	Rate	2021	348
Incidence	Mongolia	Both	All ages	Neoplasms	Rate	2021	747
Incidence	Republic of Maldives	Both	All ages	Neoplasms	Rate	2021	303
Incidence	Solomon Islands	Both	All ages	Neoplasms	Rate	2021	370
Incidence	Georgia	Both	All ages	Neoplasms	Rate	2021	826
Incidence	Republic of the Philippines	Both	All ages	Neoplasms	Rate	2021	306
Incidence	Republic of Vanuatu	Both	All ages	Neoplasms	Rate	2021	369
Incidence	Republic of Indonesia	Both	All ages	Neoplasms	Rate	2021	551
Incidence	Republic of Kiribati	Both	All ages	Neoplasms	Rate	2021	410
Incidence	Republic of Bulgaria	Both	All ages	Neoplasms	Rate	2021	1569
Incidence	Romania	Both	All ages	Neoplasms	Rate	2021	1475
Incidence	Kyrgyz Republic	Both	All ages	Neoplasms	Rate	2021	910
Incidence	Republic of Azerbaijan	Both	All ages	Neoplasms	Rate	2021	751
Incidence	Czech Republic	Both	All ages	Neoplasms	Rate	2021	1472

Incidence	Federated States of Micronesia	Both	All ages	Neoplasms	Rate	2021	444
Incidence	Turkmenistan	Both	All ages	Neoplasms	Rate	2021	683
Incidence	Independent State of Samoa	Both	All ages	Neoplasms	Rate	2021	388
Incidence	Kingdom of Thailand	Both	All ages	Neoplasms	Rate	2021	628
Incidence	Republic of Moldova	Both	All ages	Neoplasms	Rate	2021	1164
Incidence	Slovak Republic	Both	All ages	Neoplasms	Rate	2021	1416
Incidence	Republic of Korea	Both	All ages	Neoplasms	Rate	2021	1516
Incidence	North Macedonia	Both	All ages	Neoplasms	Rate	2021	1276
Incidence	Republic of Tajikistan	Both	All ages	Neoplasms	Rate	2021	622
Incidence	Bosnia and Herzegovina	Both	All ages	Neoplasms	Rate	2021	1355
Incidence	Ukraine	Both	All ages	Neoplasms	Rate	2021	1264
Incidence	Republic of Croatia	Both	All ages	Neoplasms	Rate	2021	1603
Incidence	Republic of Estonia	Both	All ages	Neoplasms	Rate	2021	1439
Incidence	Republic of Poland	Both	All ages	Neoplasms	Rate	2021	2184
Incidence	Republic of Serbia	Both	All ages	Neoplasms	Rate	2021	1366
Incidence	Republic of Uzbekistan	Both	All ages	Neoplasms	Rate	2021	671
Incidence	New Zealand	Both	All ages	Neoplasms	Rate	2021	1048
Incidence	Japan	Both	All ages	Neoplasms	Rate	2021	1852
Incidence	Hungary	Both	All ages	Neoplasms	Rate	2021	1535
Incidence	Republic of Slovenia	Both	All ages	Neoplasms	Rate	2021	1451
Incidence	Republic of Albania	Both	All ages	Neoplasms	Rate	2021	1152
Incidence	Montenegro	Both	All ages	Neoplasms	Rate	2021	1360
Incidence	Russian Federation	Both	All ages	Neoplasms	Rate	2021	1346
Incidence	Principality of Andorra	Both	All ages	Neoplasms	Rate	2021	1252

Incidence	Republic of Lithuania	Both	All ages	Neoplasms	Rate	2021	1486
Incidence	Republic of Singapore	Both	All ages	Neoplasms	Rate	2021	1402
Incidence	Australia	Both	All ages	Neoplasms	Rate	2021	1111
Incidence	Republic of Belarus	Both	All ages	Neoplasms	Rate	2021	1289
Incidence	Republic of Latvia	Both	All ages	Neoplasms	Rate	2021	1333
Incidence	Brunei Darussalam	Both	All ages	Neoplasms	Rate	2021	1182
Incidence	Republic of Cyprus	Both	All ages	Neoplasms	Rate	2021	816
Incidence	Ireland	Both	All ages	Neoplasms	Rate	2021	1121
Incidence	Republic of Italy	Both	All ages	Neoplasms	Rate	2021	1534
Incidence	Kingdom of Sweden	Both	All ages	Neoplasms	Rate	2021	1247
Incidence	Republic of Finland	Both	All ages	Neoplasms	Rate	2021	1419
Incidence	Federal Republic of Germany	Both	All ages	Neoplasms	Rate	2021	1535
Incidence	United Kingdom of Great Britain and Northern Ireland	Both	All ages	Neoplasms	Rate	2021	1058
Incidence	Republic of Malta	Both	All ages	Neoplasms	Rate	2021	1081
Incidence	Republic of Iceland	Both	All ages	Neoplasms	Rate	2021	1050
Incidence	United States of America	Both	All ages	Neoplasms	Rate	2021	4878
Incidence	Kingdom of Norway	Both	All ages	Neoplasms	Rate	2021	1387
Incidence	Republic of Cuba	Both	All ages	Neoplasms	Rate	2021	819
Incidence	Kingdom of Belgium	Both	All ages	Neoplasms	Rate	2021	1263
Incidence	Saint Lucia	Both	All ages	Neoplasms	Rate	2021	594
Incidence	Kingdom of Denmark	Both	All ages	Neoplasms	Rate	2021	1328
Incidence	Dominican Republic	Both	All ages	Neoplasms	Rate	2021	370
Incidence	Argentine Republic	Both	All ages	Neoplasms	Rate	2021	749
Incidence	Kingdom of Spain	Both	All ages	Neoplasms	Rate	2021	1175

Incidence	State of Israel	Both	All ages	Neoplasms	Rate	2021	848
Incidence	Eastern Republic of Uruguay	Both	All ages	Neoplasms	Rate	2021	1055
Incidence	Canada	Both	All ages	Neoplasms	Rate	2021	2536
Incidence	Commonwealth of the Bahamas	Both	All ages	Neoplasms	Rate	2021	557
Incidence	Republic of Guyana	Both	All ages	Neoplasms	Rate	2021	382
Incidence	Swiss Confederation	Both	All ages	Neoplasms	Rate	2021	1266
Incidence	Grand Duchy of Luxembourg	Both	All ages	Neoplasms	Rate	2021	1157
Incidence	Belize	Both	All ages	Neoplasms	Rate	2021	341
Incidence	Republic of Austria	Both	All ages	Neoplasms	Rate	2021	1836
Incidence	Commonwealth of Dominica	Both	All ages	Neoplasms	Rate	2021	565
Incidence	French Republic	Both	All ages	Neoplasms	Rate	2021	1366
Incidence	Hellenic Republic	Both	All ages	Neoplasms	Rate	2021	1569
Incidence	Republic of Suriname	Both	All ages	Neoplasms	Rate	2021	436
Incidence	Portuguese Republic	Both	All ages	Neoplasms	Rate	2021	1156
Incidence	Jamaica	Both	All ages	Neoplasms	Rate	2021	535
Incidence	Antigua and Barbuda	Both	All ages	Neoplasms	Rate	2021	593
Incidence	Kingdom of the Netherlands	Both	All ages	Neoplasms	Rate	2021	1346
Incidence	Republic of Colombia	Both	All ages	Neoplasms	Rate	2021	542
Incidence	Republic of Panama	Both	All ages	Neoplasms	Rate	2021	506
Incidence	Grenada	Both	All ages	Neoplasms	Rate	2021	564
Incidence	Republic of Chile	Both	All ages	Neoplasms	Rate	2021	878
Incidence	Saint Vincent and the Grenadines	Both	All ages	Neoplasms	Rate	2021	573
Incidence	Arab Republic of Egypt	Both	All ages	Neoplasms	Rate	2021	341
Incidence	Republic of El Salvador	Both	All ages	Neoplasms	Rate	2021	469

Incidence	Republic of Ecuador	Both	All ages	Neoplasms	Rate	2021	460
Incidence	Republic of Haiti	Both	All ages	Neoplasms	Rate	2021	320
Incidence	Kingdom of Morocco	Both	All ages	Neoplasms	Rate	2021	368
Incidence	Republic of Paraguay	Both	All ages	Neoplasms	Rate	2021	347
Incidence	Barbados	Both	All ages	Neoplasms	Rate	2021	792
Incidence	Republic of Yemen	Both	All ages	Neoplasms	Rate	2021	269
Incidence	Republic of Honduras	Both	All ages	Neoplasms	Rate	2021	359
Incidence	Republic of Trinidad and Tobago	Both	All ages	Neoplasms	Rate	2021	581
Incidence	Republic of Nicaragua	Both	All ages	Neoplasms	Rate	2021	368
Incidence	Republic of Iraq	Both	All ages	Neoplasms	Rate	2021	348
Incidence	Kingdom of Saudi Arabia	Both	All ages	Neoplasms	Rate	2021	356
Incidence	Sultanate of Oman	Both	All ages	Neoplasms	Rate	2021	283
Incidence	State of Kuwait	Both	All ages	Neoplasms	Rate	2021	417
Incidence	Kingdom of Bahrain	Both	All ages	Neoplasms	Rate	2021	399
Incidence	State of Libya	Both	All ages	Neoplasms	Rate	2021	460
Incidence	Republic of Costa Rica	Both	All ages	Neoplasms	Rate	2021	628
Incidence	Bolivarian Republic of Venezuela	Both	All ages	Neoplasms	Rate	2021	579
Incidence	Plurinational State of Bolivia	Both	All ages	Neoplasms	Rate	2021	426
Incidence	Republic of Guatemala	Both	All ages	Neoplasms	Rate	2021	362
Incidence	Republic of Tunisia	Both	All ages	Neoplasms	Rate	2021	466
Incidence	Federative Republic of Brazil	Both	All ages	Neoplasms	Rate	2021	477
Incidence	Islamic Republic of Iran	Both	All ages	Neoplasms	Rate	2021	437
Incidence	United Mexican States	Both	All ages	Neoplasms	Rate	2021	559
Incidence	Palestine	Both	All ages	Neoplasms	Rate	2021	333

Incidence	Republic of Peru	Both	All ages	Neoplasms	Rate	2021	463
Incidence	Federal Democratic Republic of Nepal	Both	All ages	Neoplasms	Rate	2021	271
Incidence	People's Republic of Bangladesh	Both	All ages	Neoplasms	Rate	2021	321
Incidence	United Arab Emirates	Both	All ages	Neoplasms	Rate	2021	362
Incidence	State of Qatar	Both	All ages	Neoplasms	Rate	2021	351
Incidence	Lebanese Republic	Both	All ages	Neoplasms	Rate	2021	554
Incidence	People's Democratic Republic of Algeria	Both	All ages	Neoplasms	Rate	2021	347
Incidence	Gabonese Republic	Both	All ages	Neoplasms	Rate	2021	244
Incidence	Hashemite Kingdom of Jordan	Both	All ages	Neoplasms	Rate	2021	238
Incidence	Republic of Kenya	Both	All ages	Neoplasms	Rate	2021	164
Incidence	Republic of Angola	Both	All ages	Neoplasms	Rate	2021	173
Incidence	Islamic Republic of Afghanistan	Both	All ages	Neoplasms	Rate	2021	263
Incidence	Republic of Uganda	Both	All ages	Neoplasms	Rate	2021	162
Incidence	Republic of the Congo	Both	All ages	Neoplasms	Rate	2021	228
Incidence	Republic of Burundi	Both	All ages	Neoplasms	Rate	2021	139
Incidence	Republic of India	Both	All ages	Neoplasms	Rate	2021	523
Incidence	Republic of Malawi	Both	All ages	Neoplasms	Rate	2021	162
Incidence	Kingdom of Eswatini	Both	All ages	Neoplasms	Rate	2021	453
Incidence	Syrian Arab Republic	Both	All ages	Neoplasms	Rate	2021	411
Incidence	Republic of Djibouti	Both	All ages	Neoplasms	Rate	2021	191
Incidence	Islamic Republic of Pakistan	Both	All ages	Neoplasms	Rate	2021	337
Incidence	Republic of Mozambique	Both	All ages	Neoplasms	Rate	2021	148
Incidence	Republic of Turkey	Both	All ages	Neoplasms	Rate	2021	695
Incidence	Republic of Equatorial Guinea	Both	All ages	Neoplasms	Rate	2021	180

Incidence	Kingdom of Lesotho	Both	All ages	Neoplasms	Rate	2021	477
Incidence	Republic of Madagascar	Both	All ages	Neoplasms	Rate	2021	149
Incidence	Kingdom of Bhutan	Both	All ages	Neoplasms	Rate	2021	318
Incidence	Republic of Seychelles	Both	All ages	Neoplasms	Rate	2021	521
Incidence	Burkina Faso	Both	All ages	Neoplasms	Rate	2021	175
Incidence	Federal Democratic Republic of Ethiopia	Both	All ages	Neoplasms	Rate	2021	155
Incidence	Republic of Zambia	Both	All ages	Neoplasms	Rate	2021	174
Incidence	Union of the Comoros	Both	All ages	Neoplasms	Rate	2021	214
Incidence	Democratic Republic of the Congo	Both	All ages	Neoplasms	Rate	2021	175
Incidence	Central African Republic	Both	All ages	Neoplasms	Rate	2021	188
Incidence	Republic of the Gambia	Both	All ages	Neoplasms	Rate	2021	171
Incidence	Republic of Mauritius	Both	All ages	Neoplasms	Rate	2021	532
Incidence	Republic of Cabo Verde	Both	All ages	Neoplasms	Rate	2021	308
Incidence	United Republic of Tanzania	Both	All ages	Neoplasms	Rate	2021	164
Incidence	Republic of Zimbabwe	Both	All ages	Neoplasms	Rate	2021	436
Incidence	Republic of South Africa	Both	All ages	Neoplasms	Rate	2021	615
Incidence	Republic of Guinea	Both	All ages	Neoplasms	Rate	2021	180
Incidence	State of Eritrea	Both	All ages	Neoplasms	Rate	2021	175
Incidence	Republic of the Niger	Both	All ages	Neoplasms	Rate	2021	142
Incidence	Republic of Rwanda	Both	All ages	Neoplasms	Rate	2021	177
Incidence	Republic of CÃ´te d'Ivoire	Both	All ages	Neoplasms	Rate	2021	166
Incidence	Republic of Benin	Both	All ages	Neoplasms	Rate	2021	164
Incidence	Republic of Botswana	Both	All ages	Neoplasms	Rate	2021	467
Incidence	Republic of Liberia	Both	All ages	Neoplasms	Rate	2021	178

Incidence	Democratic Republic of Sao Tome and Principe	Both	All ages	Neoplasms	Rate	2021	209
Incidence	American Samoa	Both	All ages	Neoplasms	Rate	2021	524
Incidence	Federal Republic of Somalia	Both	All ages	Neoplasms	Rate	2021	131
Incidence	Republic of Namibia	Both	All ages	Neoplasms	Rate	2021	442
Incidence	Islamic Republic of Mauritania	Both	All ages	Neoplasms	Rate	2021	195
Incidence	Republic of Niue	Both	All ages	Neoplasms	Rate	2021	575
Incidence	Cook Islands	Both	All ages	Neoplasms	Rate	2021	657
Incidence	Republic of Sierra Leone	Both	All ages	Neoplasms	Rate	2021	174
Incidence	Tuvalu	Both	All ages	Neoplasms	Rate	2021	440
Incidence	Republic of Palau	Both	All ages	Neoplasms	Rate	2021	638
Incidence	Guam	Both	All ages	Neoplasms	Rate	2021	521
Incidence	Republic of Ghana	Both	All ages	Neoplasms	Rate	2021	198
Incidence	Republic of Guinea-Bissau	Both	All ages	Neoplasms	Rate	2021	182
Incidence	Saint Kitts and Nevis	Both	All ages	Neoplasms	Rate	2021	566
Incidence	Republic of Chad	Both	All ages	Neoplasms	Rate	2021	151
Incidence	Republic of Cameroon	Both	All ages	Neoplasms	Rate	2021	179
Incidence	Republic of Senegal	Both	All ages	Neoplasms	Rate	2021	191
Incidence	Federal Republic of Nigeria	Both	All ages	Neoplasms	Rate	2021	181
Incidence	Republic of South Sudan	Both	All ages	Neoplasms	Rate	2021	162
Incidence	Republic of Mali	Both	All ages	Neoplasms	Rate	2021	174
Incidence	Tokelau	Both	All ages	Neoplasms	Rate	2021	508
Incidence	Bermuda	Both	All ages	Neoplasms	Rate	2021	1064
Incidence	Republic of Nauru	Both	All ages	Neoplasms	Rate	2021	419
Incidence	Togolese Republic	Both	All ages	Neoplasms	Rate	2021	191

Incidence	United States Virgin Islands	Both	All ages	Neoplasms	Rate	2021	687
Incidence	Greenland	Both	All ages	Neoplasms	Rate	2021	2119
Incidence	Northern Mariana Islands	Both	All ages	Neoplasms	Rate	2021	591
Incidence	Republic of Sudan	Both	All ages	Neoplasms	Rate	2021	284
Incidence	Republic of San Marino	Both	All ages	Neoplasms	Rate	2021	1222
Incidence	Puerto Rico	Both	All ages	Neoplasms	Rate	2021	768
Incidence	Principality of Monaco	Both	All ages	Neoplasms	Rate	2021	1860
measure	Country	sex	age	cause	metric	year	Rate
Deaths	Republic of Paraguay	Both	All ages	Neoplasms	Rate	2021	89
Deaths	Mongolia	Both	All ages	Neoplasms	Rate	2021	144
Deaths	Australia	Both	All ages	Neoplasms	Rate	2021	211
Deaths	Republic of Cabo Verde	Both	All ages	Neoplasms	Rate	2021	114
Deaths	Principality of Monaco	Both	All ages	Neoplasms	Rate	2021	543
Deaths	Central African Republic	Both	All ages	Neoplasms	Rate	2021	50
Deaths	Republic of Ecuador	Both	All ages	Neoplasms	Rate	2021	94
Deaths	Republic of the Marshall Islands	Both	All ages	Neoplasms	Rate	2021	79
Deaths	United Republic of Tanzania	Both	All ages	Neoplasms	Rate	2021	52
Deaths	Republic of Turkey	Both	All ages	Neoplasms	Rate	2021	136
Deaths	Kingdom of Norway	Both	All ages	Neoplasms	Rate	2021	235
Deaths	Republic of Peru	Both	All ages	Neoplasms	Rate	2021	98
Deaths	Arab Republic of Egypt	Both	All ages	Neoplasms	Rate	2021	59
Deaths	Republic of Austria	Both	All ages	Neoplasms	Rate	2021	240
Deaths	Republic of Belarus	Both	All ages	Neoplasms	Rate	2021	216
Deaths	Belize	Both	All ages	Neoplasms	Rate	2021	68
Deaths	Kingdom of Cambodia	Both	All ages	Neoplasms	Rate	2021	86

Deaths	Democratic People's Republic of Korea	Both	All ages	Neoplasms	Rate	2021	142
Deaths	Republic of Slovenia	Both	All ages	Neoplasms	Rate	2021	295
Deaths	Republic of Albania	Both	All ages	Neoplasms	Rate	2021	163
Deaths	Syrian Arab Republic	Both	All ages	Neoplasms	Rate	2021	62
Deaths	Kingdom of Belgium	Both	All ages	Neoplasms	Rate	2021	271
Deaths	Swiss Confederation	Both	All ages	Neoplasms	Rate	2021	215
Deaths	Turkmenistan	Both	All ages	Neoplasms	Rate	2021	71
Deaths	Kingdom of Lesotho	Both	All ages	Neoplasms	Rate	2021	108
Deaths	Republic of Guinea	Both	All ages	Neoplasms	Rate	2021	44
Deaths	Republic of Cuba	Both	All ages	Neoplasms	Rate	2021	249
Deaths	Republic of Seychelles	Both	All ages	Neoplasms	Rate	2021	148
Deaths	Taiwan (Province of China)	Both	All ages	Neoplasms	Rate	2021	236
Deaths	Republic of Tajikistan	Both	All ages	Neoplasms	Rate	2021	44
Deaths	Puerto Rico	Both	All ages	Neoplasms	Rate	2021	194
Deaths	Republic of Malta	Both	All ages	Neoplasms	Rate	2021	234
Deaths	Kingdom of Tonga	Both	All ages	Neoplasms	Rate	2021	118
Deaths	Lebanese Republic	Both	All ages	Neoplasms	Rate	2021	123
Deaths	Republic of Moldova	Both	All ages	Neoplasms	Rate	2021	185
Deaths	Republic of Kiribati	Both	All ages	Neoplasms	Rate	2021	77
Deaths	Plurinational State of Bolivia	Both	All ages	Neoplasms	Rate	2021	116
Deaths	Republic of El Salvador	Both	All ages	Neoplasms	Rate	2021	93
Deaths	Republic of Sudan	Both	All ages	Neoplasms	Rate	2021	37
Deaths	Commonwealth of the Bahamas	Both	All ages	Neoplasms	Rate	2021	154
Deaths	Islamic Republic of Afghanistan	Both	All ages	Neoplasms	Rate	2021	47

Deaths	People's Democratic Republic of Algeria	Both	All ages	Neoplasms	Rate	2021	38
Deaths	New Zealand	Both	All ages	Neoplasms	Rate	2021	207
Deaths	Republic of the Congo	Both	All ages	Neoplasms	Rate	2021	64
Deaths	Independent State of Papua New Guinea	Both	All ages	Neoplasms	Rate	2021	41
Deaths	Czech Republic	Both	All ages	Neoplasms	Rate	2021	291
Deaths	Kingdom of the Netherlands	Both	All ages	Neoplasms	Rate	2021	290
Deaths	Russian Federation	Both	All ages	Neoplasms	Rate	2021	215
Deaths	Republic of Niue	Both	All ages	Neoplasms	Rate	2021	159
Deaths	Republic of Guyana	Both	All ages	Neoplasms	Rate	2021	93
Deaths	Republic of Zimbabwe	Both	All ages	Neoplasms	Rate	2021	90
Deaths	Republic of Tunisia	Both	All ages	Neoplasms	Rate	2021	82
Deaths	Republic of the Union of Myanmar	Both	All ages	Neoplasms	Rate	2021	78
Deaths	Republic of Uzbekistan	Both	All ages	Neoplasms	Rate	2021	53
Deaths	Eastern Republic of Uruguay	Both	All ages	Neoplasms	Rate	2021	301
Deaths	Republic of Equatorial Guinea	Both	All ages	Neoplasms	Rate	2021	39
Deaths	Hungary	Both	All ages	Neoplasms	Rate	2021	335
Deaths	Northern Mariana Islands	Both	All ages	Neoplasms	Rate	2021	146
Deaths	French Republic	Both	All ages	Neoplasms	Rate	2021	290
Deaths	Antigua and Barbuda	Both	All ages	Neoplasms	Rate	2021	153
Deaths	Republic of the Niger	Both	All ages	Neoplasms	Rate	2021	25
Deaths	Federal Democratic Republic of Ethiopia	Both	All ages	Neoplasms	Rate	2021	43
Deaths	Republic of Armenia	Both	All ages	Neoplasms	Rate	2021	186
Deaths	Federated States of Micronesia	Both	All ages	Neoplasms	Rate	2021	93
Deaths	Democratic Republic of the Congo	Both	All ages	Neoplasms	Rate	2021	38

Deaths	Kingdom of Spain	Both	All ages	Neoplasms	Rate	2021	259
Deaths	Republic of Chad	Both	All ages	Neoplasms	Rate	2021	33
Deaths	Republic of Zambia	Both	All ages	Neoplasms	Rate	2021	61
Deaths	Republic of Latvia	Both	All ages	Neoplasms	Rate	2021	307
Deaths	Lao People's Democratic Republic	Both	All ages	Neoplasms	Rate	2021	66
Deaths	Republic of Nauru	Both	All ages	Neoplasms	Rate	2021	88
Deaths	Republic of Estonia	Both	All ages	Neoplasms	Rate	2021	290
Deaths	Portuguese Republic	Both	All ages	Neoplasms	Rate	2021	289
Deaths	Republic of CÃ´te d'Ivoire	Both	All ages	Neoplasms	Rate	2021	29
Deaths	Tuvalu	Both	All ages	Neoplasms	Rate	2021	95
Deaths	Brunei Darussalam	Both	All ages	Neoplasms	Rate	2021	100
Deaths	Republic of the Gambia	Both	All ages	Neoplasms	Rate	2021	36
Deaths	Bosnia and Herzegovina	Both	All ages	Neoplasms	Rate	2021	270
Deaths	Federal Republic of Somalia	Both	All ages	Neoplasms	Rate	2021	40
Deaths	United Arab Emirates	Both	All ages	Neoplasms	Rate	2021	40
Deaths	United States Virgin Islands	Both	All ages	Neoplasms	Rate	2021	197
Deaths	Islamic Republic of Pakistan	Both	All ages	Neoplasms	Rate	2021	66
Deaths	Kingdom of Bahrain	Both	All ages	Neoplasms	Rate	2021	52
Deaths	Republic of Colombia	Both	All ages	Neoplasms	Rate	2021	106
Deaths	Principality of Andorra	Both	All ages	Neoplasms	Rate	2021	228
Deaths	Republic of Suriname	Both	All ages	Neoplasms	Rate	2021	116
Deaths	Barbados	Both	All ages	Neoplasms	Rate	2021	252
Deaths	Democratic Republic of Timor-Leste	Both	All ages	Neoplasms	Rate	2021	48
Deaths	Kingdom of Denmark	Both	All ages	Neoplasms	Rate	2021	311

Deaths	Republic of Indonesia	Both	All ages	Neoplasms	Rate	2021	82
Deaths	State of Qatar	Both	All ages	Neoplasms	Rate	2021	26
Deaths	Republic of Uganda	Both	All ages	Neoplasms	Rate	2021	56
Deaths	Republic of Sierra Leone	Both	All ages	Neoplasms	Rate	2021	37
Deaths	Independent State of Samoa	Both	All ages	Neoplasms	Rate	2021	62
Deaths	Cook Islands	Both	All ages	Neoplasms	Rate	2021	173
Deaths	Republic of Yemen	Both	All ages	Neoplasms	Rate	2021	35
Deaths	Republic of Mauritius	Both	All ages	Neoplasms	Rate	2021	133
Deaths	Romania	Both	All ages	Neoplasms	Rate	2021	287
Deaths	Republic of Panama	Both	All ages	Neoplasms	Rate	2021	93
Deaths	Republic of Lithuania	Both	All ages	Neoplasms	Rate	2021	303
Deaths	Solomon Islands	Both	All ages	Neoplasms	Rate	2021	59
Deaths	Union of the Comoros	Both	All ages	Neoplasms	Rate	2021	79
Deaths	Gabonese Republic	Both	All ages	Neoplasms	Rate	2021	74
Deaths	Kingdom of Sweden	Both	All ages	Neoplasms	Rate	2021	242
Deaths	Republic of Ghana	Both	All ages	Neoplasms	Rate	2021	44
Deaths	State of Israel	Both	All ages	Neoplasms	Rate	2021	141
Deaths	Republic of Palau	Both	All ages	Neoplasms	Rate	2021	194
Deaths	Republic of Mozambique	Both	All ages	Neoplasms	Rate	2021	47
Deaths	Islamic Republic of Iran	Both	All ages	Neoplasms	Rate	2021	68
Deaths	Malaysia	Both	All ages	Neoplasms	Rate	2021	98
Deaths	Republic of Bulgaria	Both	All ages	Neoplasms	Rate	2021	332
Deaths	Republic of San Marino	Both	All ages	Neoplasms	Rate	2021	221
Deaths	Republic of Croatia	Both	All ages	Neoplasms	Rate	2021	338

Deaths	Dominican Republic	Both	All ages	Neoplasms	Rate	2021	86
Deaths	Republic of Djibouti	Both	All ages	Neoplasms	Rate	2021	64
Deaths	Hashemite Kingdom of Jordan	Both	All ages	Neoplasms	Rate	2021	40
Deaths	Republic of Cyprus	Both	All ages	Neoplasms	Rate	2021	162
Deaths	Republic of Costa Rica	Both	All ages	Neoplasms	Rate	2021	131
Deaths	Republic of Burundi	Both	All ages	Neoplasms	Rate	2021	40
Deaths	Commonwealth of Dominica	Both	All ages	Neoplasms	Rate	2021	199
Deaths	Republic of Maldives	Both	All ages	Neoplasms	Rate	2021	30
Deaths	United Kingdom of Great Britain and Northern Ireland	Both	All ages	Neoplasms	Rate	2021	276
Deaths	Republic of Guinea-Bissau	Both	All ages	Neoplasms	Rate	2021	47
Deaths	State of Kuwait	Both	All ages	Neoplasms	Rate	2021	33
Deaths	Saint Kitts and Nevis	Both	All ages	Neoplasms	Rate	2021	156
Deaths	Republic of Liberia	Both	All ages	Neoplasms	Rate	2021	38
Deaths	Republic of Iraq	Both	All ages	Neoplasms	Rate	2021	52
Deaths	Republic of Botswana	Both	All ages	Neoplasms	Rate	2021	72
Deaths	Republic of Finland	Both	All ages	Neoplasms	Rate	2021	259
Deaths	Republic of Guatemala	Both	All ages	Neoplasms	Rate	2021	65
Deaths	Argentine Republic	Both	All ages	Neoplasms	Rate	2021	162
Deaths	Republic of Mali	Both	All ages	Neoplasms	Rate	2021	41
Deaths	Kingdom of Bhutan	Both	All ages	Neoplasms	Rate	2021	54
Deaths	Republic of India	Both	All ages	Neoplasms	Rate	2021	60
Deaths	Ukraine	Both	All ages	Neoplasms	Rate	2021	191
Deaths	Bermuda	Both	All ages	Neoplasms	Rate	2021	287
Deaths	North Macedonia	Both	All ages	Neoplasms	Rate	2021	227

Deaths	Tokelau	Both	All ages	Neoplasms	Rate	2021	126
Deaths	American Samoa	Both	All ages	Neoplasms	Rate	2021	134
Deaths	People's Republic of Bangladesh	Both	All ages	Neoplasms	Rate	2021	52
Deaths	Republic of Vanuatu	Both	All ages	Neoplasms	Rate	2021	55
Deaths	Grenada	Both	All ages	Neoplasms	Rate	2021	171
Deaths	Federal Democratic Republic of Nepal	Both	All ages	Neoplasms	Rate	2021	51
Deaths	Democratic Socialist Republic of Sri Lanka	Both	All ages	Neoplasms	Rate	2021	71
Deaths	State of Eritrea	Both	All ages	Neoplasms	Rate	2021	61
Deaths	Republic of Honduras	Both	All ages	Neoplasms	Rate	2021	71
Deaths	Islamic Republic of Mauritania	Both	All ages	Neoplasms	Rate	2021	50
Deaths	Republic of South Africa	Both	All ages	Neoplasms	Rate	2021	108
Deaths	Federal Republic of Germany	Both	All ages	Neoplasms	Rate	2021	302
Deaths	Republic of the Philippines	Both	All ages	Neoplasms	Rate	2021	70
Deaths	Republic of Namibia	Both	All ages	Neoplasms	Rate	2021	60
Deaths	Republic of Madagascar	Both	All ages	Neoplasms	Rate	2021	41
Deaths	Republic of Iceland	Both	All ages	Neoplasms	Rate	2021	203
Deaths	Jamaica	Both	All ages	Neoplasms	Rate	2021	148
Deaths	Kingdom of Eswatini	Both	All ages	Neoplasms	Rate	2021	93
Deaths	Kingdom of Thailand	Both	All ages	Neoplasms	Rate	2021	190
Deaths	State of Libya	Both	All ages	Neoplasms	Rate	2021	97
Deaths	Republic of Haiti	Both	All ages	Neoplasms	Rate	2021	83
Deaths	Hellenic Republic	Both	All ages	Neoplasms	Rate	2021	350
Deaths	Montenegro	Both	All ages	Neoplasms	Rate	2021	258
Deaths	Republic of Chile	Both	All ages	Neoplasms	Rate	2021	156

Deaths	Republic of Poland	Both	All ages	Neoplasms	Rate	2021	324
Deaths	Republic of Nicaragua	Both	All ages	Neoplasms	Rate	2021	49
Deaths	Palestine	Both	All ages	Neoplasms	Rate	2021	50
Deaths	Republic of Kenya	Both	All ages	Neoplasms	Rate	2021	44
Deaths	United Mexican States	Both	All ages	Neoplasms	Rate	2021	85
Deaths	Saint Lucia	Both	All ages	Neoplasms	Rate	2021	179
Deaths	Republic of Kazakhstan	Both	All ages	Neoplasms	Rate	2021	91
Deaths	Kingdom of Morocco	Both	All ages	Neoplasms	Rate	2021	53
Deaths	Japan	Both	All ages	Neoplasms	Rate	2021	363
Deaths	Sultanate of Oman	Both	All ages	Neoplasms	Rate	2021	18
Deaths	Ireland	Both	All ages	Neoplasms	Rate	2021	186
Deaths	Canada	Both	All ages	Neoplasms	Rate	2021	235
Deaths	Republic of Serbia	Both	All ages	Neoplasms	Rate	2021	298
Deaths	Bolivarian Republic of Venezuela	Both	All ages	Neoplasms	Rate	2021	128
Deaths	Republic of Senegal	Both	All ages	Neoplasms	Rate	2021	46
Deaths	Kyrgyz Republic	Both	All ages	Neoplasms	Rate	2021	62
Deaths	Republic of Angola	Both	All ages	Neoplasms	Rate	2021	40
Deaths	Federal Republic of Nigeria	Both	All ages	Neoplasms	Rate	2021	33
Deaths	Greenland	Both	All ages	Neoplasms	Rate	2021	233
Deaths	Republic of Azerbaijan	Both	All ages	Neoplasms	Rate	2021	93
Deaths	Socialist Republic of Viet Nam	Both	All ages	Neoplasms	Rate	2021	99
Deaths	Federative Republic of Brazil	Both	All ages	Neoplasms	Rate	2021	125
Deaths	Republic of South Sudan	Both	All ages	Neoplasms	Rate	2021	54
Deaths	Republic of Korea	Both	All ages	Neoplasms	Rate	2021	194

Deaths	Republic of Italy	Both	All ages	Neoplasms	Rate	2021	316
Deaths	Georgia	Both	All ages	Neoplasms	Rate	2021	240
Deaths	Republic of Malawi	Both	All ages	Neoplasms	Rate	2021	52
Deaths	Saint Vincent and the Grenadines	Both	All ages	Neoplasms	Rate	2021	181
Deaths	People's Republic of China	Both	All ages	Neoplasms	Rate	2021	198
Deaths	United States of America	Both	All ages	Neoplasms	Rate	2021	215
Deaths	Republic of Cameroon	Both	All ages	Neoplasms	Rate	2021	41
Deaths	Democratic Republic of Sao Tome and Principe	Both	All ages	Neoplasms	Rate	2021	50
Deaths	Burkina Faso	Both	All ages	Neoplasms	Rate	2021	42
Deaths	Kingdom of Saudi Arabia	Both	All ages	Neoplasms	Rate	2021	32
Deaths	Guam	Both	All ages	Neoplasms	Rate	2021	109
Deaths	Republic of Trinidad and Tobago	Both	All ages	Neoplasms	Rate	2021	157
Deaths	Grand Duchy of Luxembourg	Both	All ages	Neoplasms	Rate	2021	202
Deaths	Republic of Rwanda	Both	All ages	Neoplasms	Rate	2021	57
Deaths	Republic of Benin	Both	All ages	Neoplasms	Rate	2021	34
Deaths	Republic of Fiji	Both	All ages	Neoplasms	Rate	2021	83
Deaths	Slovak Republic	Both	All ages	Neoplasms	Rate	2021	261
Deaths	Republic of Singapore	Both	All ages	Neoplasms	Rate	2021	113
Deaths	Togolese Republic	Both	All ages	Neoplasms	Rate	2021	42
measure	Country	sex	age	cause	metric	year	Rate
DALYs (Disability-Adjusted Life Years)	Malaysia	Both	All ages	Neoplasms	Rate	2021	2807
DALYs (Disability-Adjusted Life Years)	Federated States of Micronesia	Both	All ages	Neoplasms	Rate	2021	2974
DALYs (Disability-	North Macedonia	Both	All ages	Neoplasms	Rate	2021	5711

Adjusted Life Years)							
DALYs (Disability-Adjusted Life Years)	Kyrgyz Republic	Both	All ages	Neoplasms	Rate	2021	2011
DALYs (Disability-Adjusted Life Years)	Republic of Moldova	Both	All ages	Neoplasms	Rate	2021	4975
DALYs (Disability-Adjusted Life Years)	United States of America	Both	All ages	Neoplasms	Rate	2021	4827
DALYs (Disability-Adjusted Life Years)	State of Israel	Both	All ages	Neoplasms	Rate	2021	2984
DALYs (Disability-Adjusted Life Years)	Argentine Republic	Both	All ages	Neoplasms	Rate	2021	3919
DALYs (Disability-Adjusted Life Years)	Republic of Cuba	Both	All ages	Neoplasms	Rate	2021	5698
DALYs (Disability-Adjusted Life Years)	Plurinational State of Bolivia	Both	All ages	Neoplasms	Rate	2021	3213
DALYs (Disability-Adjusted Life Years)	Republic of Costa Rica	Both	All ages	Neoplasms	Rate	2021	3403
DALYs (Disability-Adjusted Life Years)	Bolivarian Republic of Venezuela	Both	All ages	Neoplasms	Rate	2021	3554
DALYs (Disability-Adjusted Life Years)	Republic of Haiti	Both	All ages	Neoplasms	Rate	2021	2789
DALYs (Disability-Adjusted Life Years)	Sultanate of Oman	Both	All ages	Neoplasms	Rate	2021	615
DALYs (Disability-Adjusted Life Years)	Kingdom of Bhutan	Both	All ages	Neoplasms	Rate	2021	1603

DALYs (Disability-Adjusted Life Years)	Democratic People's Republic of Korea	Both	All ages	Neoplasms	Rate	2021	4181
DALYs (Disability-Adjusted Life Years)	Republic of Yemen	Both	All ages	Neoplasms	Rate	2021	1178
DALYs (Disability-Adjusted Life Years)	Dominican Republic	Both	All ages	Neoplasms	Rate	2021	2354
DALYs (Disability-Adjusted Life Years)	State of Kuwait	Both	All ages	Neoplasms	Rate	2021	1040
DALYs (Disability-Adjusted Life Years)	Democratic Republic of the Congo	Both	All ages	Neoplasms	Rate	2021	1278
DALYs (Disability-Adjusted Life Years)	Republic of South Africa	Both	All ages	Neoplasms	Rate	2021	3131
DALYs (Disability-Adjusted Life Years)	Republic of Mauritius	Both	All ages	Neoplasms	Rate	2021	3563
DALYs (Disability-Adjusted Life Years)	Eastern Republic of Uruguay	Both	All ages	Neoplasms	Rate	2021	6559
DALYs (Disability-Adjusted Life Years)	Federative Republic of Brazil	Both	All ages	Neoplasms	Rate	2021	3344
DALYs (Disability-Adjusted Life Years)	Kingdom of Thailand	Both	All ages	Neoplasms	Rate	2021	4960
DALYs (Disability-Adjusted Life Years)	Republic of Peru	Both	All ages	Neoplasms	Rate	2021	2590
DALYs (Disability-Adjusted Life Years)	Taiwan (Province of China)	Both	All ages	Neoplasms	Rate	2021	5668
DALYs (Disability-	Democratic Republic of Timor-Leste	Both	All ages	Neoplasms	Rate	2021	1489

Adjusted Life Years)							
DALYs (Disability-Adjusted Life Years)	Republic of Mali	Both	All ages	Neoplasms	Rate	2021	1411
DALYs (Disability-Adjusted Life Years)	Brunei Darussalam	Both	All ages	Neoplasms	Rate	2021	3046
DALYs (Disability-Adjusted Life Years)	Republic of Bulgaria	Both	All ages	Neoplasms	Rate	2021	7961
DALYs (Disability-Adjusted Life Years)	Republic of Seychelles	Both	All ages	Neoplasms	Rate	2021	4032
DALYs (Disability-Adjusted Life Years)	People's Republic of China	Both	All ages	Neoplasms	Rate	2021	5006
DALYs (Disability-Adjusted Life Years)	Republic of Albania	Both	All ages	Neoplasms	Rate	2021	3776
DALYs (Disability-Adjusted Life Years)	State of Libya	Both	All ages	Neoplasms	Rate	2021	2986
DALYs (Disability-Adjusted Life Years)	Republic of Belarus	Both	All ages	Neoplasms	Rate	2021	5684
DALYs (Disability-Adjusted Life Years)	Japan	Both	All ages	Neoplasms	Rate	2021	6244
DALYs (Disability-Adjusted Life Years)	Greenland	Both	All ages	Neoplasms	Rate	2021	6367
DALYs (Disability-Adjusted Life Years)	Republic of Armenia	Both	All ages	Neoplasms	Rate	2021	4613
DALYs (Disability-Adjusted Life Years)	Republic of Vanuatu	Both	All ages	Neoplasms	Rate	2021	1803

DALYs (Disability-Adjusted Life Years)	Republic of Guinea	Both	All ages	Neoplasms	Rate	2021	1519
DALYs (Disability-Adjusted Life Years)	Kingdom of Norway	Both	All ages	Neoplasms	Rate	2021	4694
DALYs (Disability-Adjusted Life Years)	Palestine	Both	All ages	Neoplasms	Rate	2021	1568
DALYs (Disability-Adjusted Life Years)	Portuguese Republic	Both	All ages	Neoplasms	Rate	2021	5930
DALYs (Disability-Adjusted Life Years)	Tuvalu	Both	All ages	Neoplasms	Rate	2021	2737
DALYs (Disability-Adjusted Life Years)	People's Republic of Bangladesh	Both	All ages	Neoplasms	Rate	2021	1611
DALYs (Disability-Adjusted Life Years)	Federal Republic of Germany	Both	All ages	Neoplasms	Rate	2021	6148
DALYs (Disability-Adjusted Life Years)	Republic of Nauru	Both	All ages	Neoplasms	Rate	2021	2975
DALYs (Disability-Adjusted Life Years)	Tokelau	Both	All ages	Neoplasms	Rate	2021	3487
DALYs (Disability-Adjusted Life Years)	Gabonese Republic	Both	All ages	Neoplasms	Rate	2021	2251
DALYs (Disability-Adjusted Life Years)	Republic of Singapore	Both	All ages	Neoplasms	Rate	2021	2659
DALYs (Disability-Adjusted Life Years)	Slovak Republic	Both	All ages	Neoplasms	Rate	2021	6299
DALYs (Disability-	Republic of Slovenia	Both	All ages	Neoplasms	Rate	2021	6043

Adjusted Life Years)							
DALYs (Disability-Adjusted Life Years)	Republic of Finland	Both	All ages	Neoplasms	Rate	2021	5159
DALYs (Disability-Adjusted Life Years)	Republic of Rwanda	Both	All ages	Neoplasms	Rate	2021	1949
DALYs (Disability-Adjusted Life Years)	Kingdom of Denmark	Both	All ages	Neoplasms	Rate	2021	6107
DALYs (Disability-Adjusted Life Years)	Republic of Zimbabwe	Both	All ages	Neoplasms	Rate	2021	3044
DALYs (Disability-Adjusted Life Years)	Republic of Liberia	Both	All ages	Neoplasms	Rate	2021	1313
DALYs (Disability-Adjusted Life Years)	Jamaica	Both	All ages	Neoplasms	Rate	2021	3674
DALYs (Disability-Adjusted Life Years)	Republic of El Salvador	Both	All ages	Neoplasms	Rate	2021	2507
DALYs (Disability-Adjusted Life Years)	Republic of Sudan	Both	All ages	Neoplasms	Rate	2021	1319
DALYs (Disability-Adjusted Life Years)	Union of the Comoros	Both	All ages	Neoplasms	Rate	2021	2520
DALYs (Disability-Adjusted Life Years)	Kingdom of Spain	Both	All ages	Neoplasms	Rate	2021	5344
DALYs (Disability-Adjusted Life Years)	United States Virgin Islands	Both	All ages	Neoplasms	Rate	2021	4504
DALYs (Disability-Adjusted Life Years)	Burkina Faso	Both	All ages	Neoplasms	Rate	2021	1407

DALYs (Disability-Adjusted Life Years)	Kingdom of Bahrain	Both	All ages	Neoplasms	Rate	2021	1655
DALYs (Disability-Adjusted Life Years)	Socialist Republic of Viet Nam	Both	All ages	Neoplasms	Rate	2021	2906
DALYs (Disability-Adjusted Life Years)	Kingdom of Cambodia	Both	All ages	Neoplasms	Rate	2021	2662
DALYs (Disability-Adjusted Life Years)	Republic of Korea	Both	All ages	Neoplasms	Rate	2021	4239
DALYs (Disability-Adjusted Life Years)	Republic of Kiribati	Both	All ages	Neoplasms	Rate	2021	2628
DALYs (Disability-Adjusted Life Years)	Federal Democratic Republic of Nepal	Both	All ages	Neoplasms	Rate	2021	1569
DALYs (Disability-Adjusted Life Years)	French Republic	Both	All ages	Neoplasms	Rate	2021	5869
DALYs (Disability-Adjusted Life Years)	Bosnia and Herzegovina	Both	All ages	Neoplasms	Rate	2021	6346
DALYs (Disability-Adjusted Life Years)	Czech Republic	Both	All ages	Neoplasms	Rate	2021	6299
DALYs (Disability-Adjusted Life Years)	State of Eritrea	Both	All ages	Neoplasms	Rate	2021	2240
DALYs (Disability-Adjusted Life Years)	United Republic of Tanzania	Both	All ages	Neoplasms	Rate	2021	1815
DALYs (Disability-Adjusted Life Years)	People's Democratic Republic of Algeria	Both	All ages	Neoplasms	Rate	2021	1143
DALYs (Disability-	State of Qatar	Both	All ages	Neoplasms	Rate	2021	929

Adjusted Life Years)							
DALYs (Disability-Adjusted Life Years)	Republic of Burundi	Both	All ages	Neoplasms	Rate	2021	1464
DALYs (Disability-Adjusted Life Years)	Republic of Cabo Verde	Both	All ages	Neoplasms	Rate	2021	2901
DALYs (Disability-Adjusted Life Years)	Republic of Indonesia	Both	All ages	Neoplasms	Rate	2021	2513
DALYs (Disability-Adjusted Life Years)	Republic of South Sudan	Both	All ages	Neoplasms	Rate	2021	1990
DALYs (Disability-Adjusted Life Years)	Kingdom of Saudi Arabia	Both	All ages	Neoplasms	Rate	2021	1193
DALYs (Disability-Adjusted Life Years)	Republic of Latvia	Both	All ages	Neoplasms	Rate	2021	6924
DALYs (Disability-Adjusted Life Years)	Barbados	Both	All ages	Neoplasms	Rate	2021	5719
DALYs (Disability-Adjusted Life Years)	Georgia	Both	All ages	Neoplasms	Rate	2021	6042
DALYs (Disability-Adjusted Life Years)	Australia	Both	All ages	Neoplasms	Rate	2021	4419
DALYs (Disability-Adjusted Life Years)	Republic of Trinidad and Tobago	Both	All ages	Neoplasms	Rate	2021	4065
DALYs (Disability-Adjusted Life Years)	Republic of Benin	Both	All ages	Neoplasms	Rate	2021	1141
DALYs (Disability-Adjusted Life Years)	Republic of Azerbaijan	Both	All ages	Neoplasms	Rate	2021	2864

DALYs (Disability-Adjusted Life Years)	Kingdom of Sweden	Both	All ages	Neoplasms	Rate	2021	4507
DALYs (Disability-Adjusted Life Years)	Republic of India	Both	All ages	Neoplasms	Rate	2021	1847
DALYs (Disability-Adjusted Life Years)	Federal Republic of Somalia	Both	All ages	Neoplasms	Rate	2021	1555
DALYs (Disability-Adjusted Life Years)	Republic of Guatemala	Both	All ages	Neoplasms	Rate	2021	1902
DALYs (Disability-Adjusted Life Years)	Antigua and Barbuda	Both	All ages	Neoplasms	Rate	2021	3757
DALYs (Disability-Adjusted Life Years)	Republic of Iceland	Both	All ages	Neoplasms	Rate	2021	4333
DALYs (Disability-Adjusted Life Years)	Arab Republic of Egypt	Both	All ages	Neoplasms	Rate	2021	1878
DALYs (Disability-Adjusted Life Years)	Republic of Honduras	Both	All ages	Neoplasms	Rate	2021	2061
DALYs (Disability-Adjusted Life Years)	Republic of Nicaragua	Both	All ages	Neoplasms	Rate	2021	1473
DALYs (Disability-Adjusted Life Years)	Federal Democratic Republic of Ethiopia	Both	All ages	Neoplasms	Rate	2021	1555
DALYs (Disability-Adjusted Life Years)	Republic of Iraq	Both	All ages	Neoplasms	Rate	2021	1689
DALYs (Disability-Adjusted Life Years)	Republic of Maldives	Both	All ages	Neoplasms	Rate	2021	886
DALYs (Disability-	Republic of the Niger	Both	All ages	Neoplasms	Rate	2021	877

Adjusted Life Years)							
DALYs (Disability-Adjusted Life Years)	Swiss Confederation	Both	All ages	Neoplasms	Rate	2021	4259
DALYs (Disability-Adjusted Life Years)	Republic of Turkey	Both	All ages	Neoplasms	Rate	2021	3554
DALYs (Disability-Adjusted Life Years)	Republic of Chad	Both	All ages	Neoplasms	Rate	2021	1145
DALYs (Disability-Adjusted Life Years)	Saint Lucia	Both	All ages	Neoplasms	Rate	2021	4339
DALYs (Disability-Adjusted Life Years)	Islamic Republic of Mauritania	Both	All ages	Neoplasms	Rate	2021	1448
DALYs (Disability-Adjusted Life Years)	Northern Mariana Islands	Both	All ages	Neoplasms	Rate	2021	4191
DALYs (Disability-Adjusted Life Years)	Republic of Estonia	Both	All ages	Neoplasms	Rate	2021	6208
DALYs (Disability-Adjusted Life Years)	Saint Vincent and the Grenadines	Both	All ages	Neoplasms	Rate	2021	4641
DALYs (Disability-Adjusted Life Years)	Republic of Fiji	Both	All ages	Neoplasms	Rate	2021	2579
DALYs (Disability-Adjusted Life Years)	Republic of Croatia	Both	All ages	Neoplasms	Rate	2021	7261
DALYs (Disability-Adjusted Life Years)	Republic of Senegal	Both	All ages	Neoplasms	Rate	2021	1389
DALYs (Disability-Adjusted Life Years)	Republic of Zambia	Both	All ages	Neoplasms	Rate	2021	2222

DALYs (Disability-Adjusted Life Years)	United Kingdom of Great Britain and Northern Ireland	Both	All ages	Neoplasms	Rate	2021	5454
DALYs (Disability-Adjusted Life Years)	Republic of the Philippines	Both	All ages	Neoplasms	Rate	2021	2252
DALYs (Disability-Adjusted Life Years)	Principality of Monaco	Both	All ages	Neoplasms	Rate	2021	11027
DALYs (Disability-Adjusted Life Years)	Lao People's Democratic Republic	Both	All ages	Neoplasms	Rate	2021	2134
DALYs (Disability-Adjusted Life Years)	Saint Kitts and Nevis	Both	All ages	Neoplasms	Rate	2021	4051
DALYs (Disability-Adjusted Life Years)	Republic of Kazakhstan	Both	All ages	Neoplasms	Rate	2021	2709
DALYs (Disability-Adjusted Life Years)	Islamic Republic of Pakistan	Both	All ages	Neoplasms	Rate	2021	2303
DALYs (Disability-Adjusted Life Years)	Hellenic Republic	Both	All ages	Neoplasms	Rate	2021	6875
DALYs (Disability-Adjusted Life Years)	Republic of Madagascar	Both	All ages	Neoplasms	Rate	2021	1548
DALYs (Disability-Adjusted Life Years)	Independent State of Papua New Guinea	Both	All ages	Neoplasms	Rate	2021	1449
DALYs (Disability-Adjusted Life Years)	Republic of the Marshall Islands	Both	All ages	Neoplasms	Rate	2021	2681
DALYs (Disability-Adjusted Life Years)	Central African Republic	Both	All ages	Neoplasms	Rate	2021	1778
DALYs (Disability-	Republic of the Gambia	Both	All ages	Neoplasms	Rate	2021	1267

Adjusted Life Years)							
DALYs (Disability-Adjusted Life Years)	Republic of Suriname	Both	All ages	Neoplasms	Rate	2021	3231
DALYs (Disability-Adjusted Life Years)	Commonwealth of the Bahamas	Both	All ages	Neoplasms	Rate	2021	4338
DALYs (Disability-Adjusted Life Years)	Democratic Republic of Sao Tome and Principe	Both	All ages	Neoplasms	Rate	2021	1477
DALYs (Disability-Adjusted Life Years)	Republic of Lithuania	Both	All ages	Neoplasms	Rate	2021	6927
DALYs (Disability-Adjusted Life Years)	Hungary	Both	All ages	Neoplasms	Rate	2021	7814
DALYs (Disability-Adjusted Life Years)	Republic of Uganda	Both	All ages	Neoplasms	Rate	2021	2023
DALYs (Disability-Adjusted Life Years)	Bermuda	Both	All ages	Neoplasms	Rate	2021	5992
DALYs (Disability-Adjusted Life Years)	Romania	Both	All ages	Neoplasms	Rate	2021	6996
DALYs (Disability-Adjusted Life Years)	Kingdom of Lesotho	Both	All ages	Neoplasms	Rate	2021	3411
DALYs (Disability-Adjusted Life Years)	Togolese Republic	Both	All ages	Neoplasms	Rate	2021	1403
DALYs (Disability-Adjusted Life Years)	Syrian Arab Republic	Both	All ages	Neoplasms	Rate	2021	1844
DALYs (Disability-Adjusted Life Years)	New Zealand	Both	All ages	Neoplasms	Rate	2021	4512

DALYs (Disability-Adjusted Life Years)	Republic of Niue	Both	All ages	Neoplasms	Rate	2021	4163
DALYs (Disability-Adjusted Life Years)	Ireland	Both	All ages	Neoplasms	Rate	2021	4047
DALYs (Disability-Adjusted Life Years)	Republic of Djibouti	Both	All ages	Neoplasms	Rate	2021	2171
DALYs (Disability-Adjusted Life Years)	Republic of the Union of Myanmar	Both	All ages	Neoplasms	Rate	2021	2396
DALYs (Disability-Adjusted Life Years)	Turkmenistan	Both	All ages	Neoplasms	Rate	2021	2430
DALYs (Disability-Adjusted Life Years)	Republic of Cameroon	Both	All ages	Neoplasms	Rate	2021	1383
DALYs (Disability-Adjusted Life Years)	Solomon Islands	Both	All ages	Neoplasms	Rate	2021	2030
DALYs (Disability-Adjusted Life Years)	Grenada	Both	All ages	Neoplasms	Rate	2021	4546
DALYs (Disability-Adjusted Life Years)	Mongolia	Both	All ages	Neoplasms	Rate	2021	4368
DALYs (Disability-Adjusted Life Years)	Federal Republic of Nigeria	Both	All ages	Neoplasms	Rate	2021	1135
DALYs (Disability-Adjusted Life Years)	Belize	Both	All ages	Neoplasms	Rate	2021	1999
DALYs (Disability-Adjusted Life Years)	Islamic Republic of Iran	Both	All ages	Neoplasms	Rate	2021	1926
DALYs (Disability-	Republic of Tunisia	Both	All ages	Neoplasms	Rate	2021	2237

Adjusted Life Years)							
DALYs (Disability-Adjusted Life Years)	Kingdom of Belgium	Both	All ages	Neoplasms	Rate	2021	5517
DALYs (Disability-Adjusted Life Years)	Republic of Malta	Both	All ages	Neoplasms	Rate	2021	4973
DALYs (Disability-Adjusted Life Years)	United Mexican States	Both	All ages	Neoplasms	Rate	2021	2414
DALYs (Disability-Adjusted Life Years)	Islamic Republic of Afghanistan	Both	All ages	Neoplasms	Rate	2021	1841
DALYs (Disability-Adjusted Life Years)	Russian Federation	Both	All ages	Neoplasms	Rate	2021	5466
DALYs (Disability-Adjusted Life Years)	Republic of Botswana	Both	All ages	Neoplasms	Rate	2021	2214
DALYs (Disability-Adjusted Life Years)	Republic of Italy	Both	All ages	Neoplasms	Rate	2021	6096
DALYs (Disability-Adjusted Life Years)	Republic of Paraguay	Both	All ages	Neoplasms	Rate	2021	2460
DALYs (Disability-Adjusted Life Years)	Principality of Andorra	Both	All ages	Neoplasms	Rate	2021	5127
DALYs (Disability-Adjusted Life Years)	Hashemite Kingdom of Jordan	Both	All ages	Neoplasms	Rate	2021	1310
DALYs (Disability-Adjusted Life Years)	Montenegro	Both	All ages	Neoplasms	Rate	2021	6267
DALYs (Disability-Adjusted Life Years)	Republic of Angola	Both	All ages	Neoplasms	Rate	2021	1365

DALYs (Disability-Adjusted Life Years)	Republic of Tajikistan	Both	All ages	Neoplasms	Rate	2021	1610
DALYs (Disability-Adjusted Life Years)	Republic of CÃ´te d'Ivoire	Both	All ages	Neoplasms	Rate	2021	977
DALYs (Disability-Adjusted Life Years)	Kingdom of Morocco	Both	All ages	Neoplasms	Rate	2021	1516
DALYs (Disability-Adjusted Life Years)	United Arab Emirates	Both	All ages	Neoplasms	Rate	2021	1435
DALYs (Disability-Adjusted Life Years)	Republic of Kenya	Both	All ages	Neoplasms	Rate	2021	1491
DALYs (Disability-Adjusted Life Years)	Republic of Palau	Both	All ages	Neoplasms	Rate	2021	5475
DALYs (Disability-Adjusted Life Years)	Commonwealth of Dominica	Both	All ages	Neoplasms	Rate	2021	4991
DALYs (Disability-Adjusted Life Years)	Republic of Ecuador	Both	All ages	Neoplasms	Rate	2021	2475
DALYs (Disability-Adjusted Life Years)	Republic of Sierra Leone	Both	All ages	Neoplasms	Rate	2021	1220
DALYs (Disability-Adjusted Life Years)	Republic of Chile	Both	All ages	Neoplasms	Rate	2021	3606
DALYs (Disability-Adjusted Life Years)	Kingdom of Tonga	Both	All ages	Neoplasms	Rate	2021	3165
DALYs (Disability-Adjusted Life Years)	Puerto Rico	Both	All ages	Neoplasms	Rate	2021	4202
DALYs (Disability-	Democratic Socialist Republic of Sri Lanka	Both	All ages	Neoplasms	Rate	2021	1929

Adjusted Life Years)							
DALYs (Disability-Adjusted Life Years)	Republic of Panama	Both	All ages	Neoplasms	Rate	2021	2479
DALYs (Disability-Adjusted Life Years)	Lebanese Republic	Both	All ages	Neoplasms	Rate	2021	2835
DALYs (Disability-Adjusted Life Years)	Republic of Poland	Both	All ages	Neoplasms	Rate	2021	7166
DALYs (Disability-Adjusted Life Years)	Republic of Austria	Both	All ages	Neoplasms	Rate	2021	4929
DALYs (Disability-Adjusted Life Years)	American Samoa	Both	All ages	Neoplasms	Rate	2021	3732
DALYs (Disability-Adjusted Life Years)	Independent State of Samoa	Both	All ages	Neoplasms	Rate	2021	1914
DALYs (Disability-Adjusted Life Years)	Republic of Malawi	Both	All ages	Neoplasms	Rate	2021	1939
DALYs (Disability-Adjusted Life Years)	Grand Duchy of Luxembourg	Both	All ages	Neoplasms	Rate	2021	4287
DALYs (Disability-Adjusted Life Years)	Republic of Uzbekistan	Both	All ages	Neoplasms	Rate	2021	1851
DALYs (Disability-Adjusted Life Years)	Republic of the Congo	Both	All ages	Neoplasms	Rate	2021	2123
DALYs (Disability-Adjusted Life Years)	Ukraine	Both	All ages	Neoplasms	Rate	2021	5186
DALYs (Disability-Adjusted Life Years)	Cook Islands	Both	All ages	Neoplasms	Rate	2021	3961

DALYs (Disability-Adjusted Life Years)	Canada	Both	All ages	Neoplasms	Rate	2021	4859
DALYs (Disability-Adjusted Life Years)	Republic of Namibia	Both	All ages	Neoplasms	Rate	2021	1974
DALYs (Disability-Adjusted Life Years)	Republic of Colombia	Both	All ages	Neoplasms	Rate	2021	2803
DALYs (Disability-Adjusted Life Years)	Republic of Cyprus	Both	All ages	Neoplasms	Rate	2021	3544
DALYs (Disability-Adjusted Life Years)	Republic of Serbia	Both	All ages	Neoplasms	Rate	2021	7008
DALYs (Disability-Adjusted Life Years)	Republic of Ghana	Both	All ages	Neoplasms	Rate	2021	1411
DALYs (Disability-Adjusted Life Years)	Republic of San Marino	Both	All ages	Neoplasms	Rate	2021	4420
DALYs (Disability-Adjusted Life Years)	Republic of Equatorial Guinea	Both	All ages	Neoplasms	Rate	2021	1329
DALYs (Disability-Adjusted Life Years)	Republic of Guyana	Both	All ages	Neoplasms	Rate	2021	2804
DALYs (Disability-Adjusted Life Years)	Republic of Mozambique	Both	All ages	Neoplasms	Rate	2021	1717
DALYs (Disability-Adjusted Life Years)	Kingdom of Eswatini	Both	All ages	Neoplasms	Rate	2021	3086
DALYs (Disability-Adjusted Life Years)	Kingdom of the Netherlands	Both	All ages	Neoplasms	Rate	2021	5995
DALYs (Disability-	Guam	Both	All ages	Neoplasms	Rate	2021	3094

Adjusted Life Years)							
DALYs (Disability-Adjusted Life Years)	Republic of Guinea-Bissau	Both	All ages	Neoplasms	Rate	2021	1673

S2C: Country CRDs incidence, mortality and DLAYs

measure	Country	sex	age	cause	metric	year	Rate
Incidence	Democratic People's Republic of Korea	Both	All ages	Chronic respiratory diseases	Rate	2021	799
Incidence	Maldives	Both	All ages	Chronic respiratory diseases	Rate	2021	385
Incidence	Philippines	Both	All ages	Chronic respiratory diseases	Rate	2021	864
Incidence	Kiribati	Both	All ages	Chronic respiratory diseases	Rate	2021	661
Incidence	Vanuatu	Both	All ages	Chronic respiratory diseases	Rate	2021	513
Incidence	Indonesia	Both	All ages	Chronic respiratory diseases	Rate	2021	510
Incidence	Thailand	Both	All ages	Chronic respiratory diseases	Rate	2021	583
Incidence	Micronesia (Federated States of)	Both	All ages	Chronic respiratory diseases	Rate	2021	493
Incidence	China	Both	All ages	Chronic respiratory diseases	Rate	2021	594
Incidence	Malaysia	Both	All ages	Chronic respiratory diseases	Rate	2021	498
Incidence	Tajikistan	Both	All ages	Chronic respiratory diseases	Rate	2021	515
Incidence	Croatia	Both	All ages	Chronic respiratory diseases	Rate	2021	864

Incidence	Taiwan	Both	All ages	Chronic respiratory diseases	Rate	2021	743
Incidence	Azerbaijan	Both	All ages	Chronic respiratory diseases	Rate	2021	495
Incidence	Samoa	Both	All ages	Chronic respiratory diseases	Rate	2021	522
Incidence	Uzbekistan	Both	All ages	Chronic respiratory diseases	Rate	2021	642
Incidence	Viet Nam	Both	All ages	Chronic respiratory diseases	Rate	2021	625
Incidence	Cambodia	Both	All ages	Chronic respiratory diseases	Rate	2021	490
Incidence	Myanmar	Both	All ages	Chronic respiratory diseases	Rate	2021	516
Incidence	Kazakhstan	Both	All ages	Chronic respiratory diseases	Rate	2021	483
Incidence	Sri Lanka	Both	All ages	Chronic respiratory diseases	Rate	2021	698
Incidence	Fiji	Both	All ages	Chronic respiratory diseases	Rate	2021	501
Incidence	Albania	Both	All ages	Chronic respiratory diseases	Rate	2021	661
Incidence	Tonga	Both	All ages	Chronic respiratory diseases	Rate	2021	709
Incidence	Bulgaria	Both	All ages	Chronic respiratory diseases	Rate	2021	742
Incidence	Marshall Islands	Both	All ages	Chronic respiratory diseases	Rate	2021	463
Incidence	Lao People's Democratic Republic	Both	All ages	Chronic respiratory diseases	Rate	2021	486
Incidence	Mongolia	Both	All ages	Chronic respiratory diseases	Rate	2021	501

Incidence	Hungary	Both	All ages	Chronic respiratory diseases	Rate	2021	773
Incidence	Turkmenistan	Both	All ages	Chronic respiratory diseases	Rate	2021	416
Incidence	Solomon Islands	Both	All ages	Chronic respiratory diseases	Rate	2021	510
Incidence	Timor-Leste	Both	All ages	Chronic respiratory diseases	Rate	2021	676
Incidence	Slovenia	Both	All ages	Chronic respiratory diseases	Rate	2021	908
Incidence	Papua New Guinea	Both	All ages	Chronic respiratory diseases	Rate	2021	669
Incidence	Montenegro	Both	All ages	Chronic respiratory diseases	Rate	2021	669
Incidence	Russian Federation	Both	All ages	Chronic respiratory diseases	Rate	2021	545
Incidence	Armenia	Both	All ages	Chronic respiratory diseases	Rate	2021	511
Incidence	Kyrgyzstan	Both	All ages	Chronic respiratory diseases	Rate	2021	593
Incidence	Georgia	Both	All ages	Chronic respiratory diseases	Rate	2021	532
Incidence	Czechia	Both	All ages	Chronic respiratory diseases	Rate	2021	698
Incidence	Belarus	Both	All ages	Chronic respiratory diseases	Rate	2021	734
Incidence	Romania	Both	All ages	Chronic respiratory diseases	Rate	2021	825
Incidence	Australia	Both	All ages	Chronic respiratory diseases	Rate	2021	625
Incidence	Bosnia and Herzegovina	Both	All ages	Chronic respiratory diseases	Rate	2021	916

Incidence	Denmark	Both	All ages	Chronic respiratory diseases	Rate	2021	731
Incidence	Latvia	Both	All ages	Chronic respiratory diseases	Rate	2021	615
Incidence	Brunei Darussalam	Both	All ages	Chronic respiratory diseases	Rate	2021	597
Incidence	France	Both	All ages	Chronic respiratory diseases	Rate	2021	693
Incidence	Slovakia	Both	All ages	Chronic respiratory diseases	Rate	2021	624
Incidence	Luxembourg	Both	All ages	Chronic respiratory diseases	Rate	2021	685
Incidence	North Macedonia	Both	All ages	Chronic respiratory diseases	Rate	2021	973
Incidence	Republic of Moldova	Both	All ages	Chronic respiratory diseases	Rate	2021	537
Incidence	Republic of Korea	Both	All ages	Chronic respiratory diseases	Rate	2021	639
Incidence	Greece	Both	All ages	Chronic respiratory diseases	Rate	2021	714
Incidence	Netherlands	Both	All ages	Chronic respiratory diseases	Rate	2021	584
Incidence	Austria	Both	All ages	Chronic respiratory diseases	Rate	2021	681
Incidence	Ukraine	Both	All ages	Chronic respiratory diseases	Rate	2021	556
Incidence	Poland	Both	All ages	Chronic respiratory diseases	Rate	2021	1340
Incidence	Estonia	Both	All ages	Chronic respiratory diseases	Rate	2021	570
Incidence	Cyprus	Both	All ages	Chronic respiratory diseases	Rate	2021	703

Incidence	Ireland	Both	All ages	Chronic respiratory diseases	Rate	2021	726
Incidence	Andorra	Both	All ages	Chronic respiratory diseases	Rate	2021	660
Incidence	Switzerland	Both	All ages	Chronic respiratory diseases	Rate	2021	686
Incidence	Italy	Both	All ages	Chronic respiratory diseases	Rate	2021	697
Incidence	Serbia	Both	All ages	Chronic respiratory diseases	Rate	2021	746
Incidence	Japan	Both	All ages	Chronic respiratory diseases	Rate	2021	732
Incidence	New Zealand	Both	All ages	Chronic respiratory diseases	Rate	2021	771
Incidence	Portugal	Both	All ages	Chronic respiratory diseases	Rate	2021	841
Incidence	Finland	Both	All ages	Chronic respiratory diseases	Rate	2021	688
Incidence	Lithuania	Both	All ages	Chronic respiratory diseases	Rate	2021	593
Incidence	Singapore	Both	All ages	Chronic respiratory diseases	Rate	2021	434
Incidence	Malta	Both	All ages	Chronic respiratory diseases	Rate	2021	694
Incidence	Germany	Both	All ages	Chronic respiratory diseases	Rate	2021	706
Incidence	Dominica	Both	All ages	Chronic respiratory diseases	Rate	2021	932
Incidence	Iceland	Both	All ages	Chronic respiratory diseases	Rate	2021	811
Incidence	Chile	Both	All ages	Chronic respiratory diseases	Rate	2021	759

Incidence	Sweden	Both	All ages	Chronic respiratory diseases	Rate	2021	968
Incidence	Antigua and Barbuda	Both	All ages	Chronic respiratory diseases	Rate	2021	854
Incidence	Israel	Both	All ages	Chronic respiratory diseases	Rate	2021	566
Incidence	Belgium	Both	All ages	Chronic respiratory diseases	Rate	2021	660
Incidence	Norway	Both	All ages	Chronic respiratory diseases	Rate	2021	852
Incidence	Grenada	Both	All ages	Chronic respiratory diseases	Rate	2021	1192
Incidence	United Kingdom	Both	All ages	Chronic respiratory diseases	Rate	2021	1037
Incidence	Barbados	Both	All ages	Chronic respiratory diseases	Rate	2021	1007
Incidence	Haiti	Both	All ages	Chronic respiratory diseases	Rate	2021	1949
Incidence	Cuba	Both	All ages	Chronic respiratory diseases	Rate	2021	1021
Incidence	United States of America	Both	All ages	Chronic respiratory diseases	Rate	2021	1545
Incidence	Spain	Both	All ages	Chronic respiratory diseases	Rate	2021	711
Incidence	Argentina	Both	All ages	Chronic respiratory diseases	Rate	2021	728
Incidence	Trinidad and Tobago	Both	All ages	Chronic respiratory diseases	Rate	2021	630
Incidence	Saint Lucia	Both	All ages	Chronic respiratory diseases	Rate	2021	922
Incidence	Bolivia (Plurinational State of)	Both	All ages	Chronic respiratory diseases	Rate	2021	797

Incidence	Costa Rica	Both	All ages	Chronic respiratory diseases	Rate	2021	962
Incidence	Dominican Republic	Both	All ages	Chronic respiratory diseases	Rate	2021	753
Incidence	Uruguay	Both	All ages	Chronic respiratory diseases	Rate	2021	712
Incidence	Brazil	Both	All ages	Chronic respiratory diseases	Rate	2021	946
Incidence	Guatemala	Both	All ages	Chronic respiratory diseases	Rate	2021	656
Incidence	Guyana	Both	All ages	Chronic respiratory diseases	Rate	2021	1029
Incidence	Peru	Both	All ages	Chronic respiratory diseases	Rate	2021	939
Incidence	Suriname	Both	All ages	Chronic respiratory diseases	Rate	2021	929
Incidence	Canada	Both	All ages	Chronic respiratory diseases	Rate	2021	1011
Incidence	Bahamas	Both	All ages	Chronic respiratory diseases	Rate	2021	857
Incidence	Belize	Both	All ages	Chronic respiratory diseases	Rate	2021	1080
Incidence	Iran (Islamic Republic of)	Both	All ages	Chronic respiratory diseases	Rate	2021	566
Incidence	Colombia	Both	All ages	Chronic respiratory diseases	Rate	2021	742
Incidence	Mexico	Both	All ages	Chronic respiratory diseases	Rate	2021	594
Incidence	Saint Vincent and the Grenadines	Both	All ages	Chronic respiratory diseases	Rate	2021	886
Incidence	Jamaica	Both	All ages	Chronic respiratory diseases	Rate	2021	943

Incidence	Jordan	Both	All ages	Chronic respiratory diseases	Rate	2021	646
Incidence	Panama	Both	All ages	Chronic respiratory diseases	Rate	2021	913
Incidence	El Salvador	Both	All ages	Chronic respiratory diseases	Rate	2021	1066
Incidence	Qatar	Both	All ages	Chronic respiratory diseases	Rate	2021	482
Incidence	Algeria	Both	All ages	Chronic respiratory diseases	Rate	2021	651
Incidence	Syrian Arab Republic	Both	All ages	Chronic respiratory diseases	Rate	2021	765
Incidence	Honduras	Both	All ages	Chronic respiratory diseases	Rate	2021	787
Incidence	Lebanon	Both	All ages	Chronic respiratory diseases	Rate	2021	790
Incidence	Ecuador	Both	All ages	Chronic respiratory diseases	Rate	2021	778
Incidence	Morocco	Both	All ages	Chronic respiratory diseases	Rate	2021	548
Incidence	Egypt	Both	All ages	Chronic respiratory diseases	Rate	2021	645
Incidence	Afghanistan	Both	All ages	Chronic respiratory diseases	Rate	2021	807
Incidence	TÃ¼rkiye	Both	All ages	Chronic respiratory diseases	Rate	2021	876
Incidence	Oman	Both	All ages	Chronic respiratory diseases	Rate	2021	636
Incidence	Nicaragua	Both	All ages	Chronic respiratory diseases	Rate	2021	857
Incidence	Paraguay	Both	All ages	Chronic respiratory diseases	Rate	2021	1054

Incidence	Iraq	Both	All ages	Chronic respiratory diseases	Rate	2021	627
Incidence	Bahrain	Both	All ages	Chronic respiratory diseases	Rate	2021	537
Incidence	Yemen	Both	All ages	Chronic respiratory diseases	Rate	2021	726
Incidence	Bhutan	Both	All ages	Chronic respiratory diseases	Rate	2021	457
Incidence	Kuwait	Both	All ages	Chronic respiratory diseases	Rate	2021	531
Incidence	Venezuela (Bolivarian Republic of)	Both	All ages	Chronic respiratory diseases	Rate	2021	825
Incidence	Central African Republic	Both	All ages	Chronic respiratory diseases	Rate	2021	857
Incidence	Saudi Arabia	Both	All ages	Chronic respiratory diseases	Rate	2021	478
Incidence	Libya	Both	All ages	Chronic respiratory diseases	Rate	2021	578
Incidence	Nepal	Both	All ages	Chronic respiratory diseases	Rate	2021	505
Incidence	Comoros	Both	All ages	Chronic respiratory diseases	Rate	2021	853
Incidence	Democratic Republic of the Congo	Both	All ages	Chronic respiratory diseases	Rate	2021	644
Incidence	Tunisia	Both	All ages	Chronic respiratory diseases	Rate	2021	689
Incidence	Zambia	Both	All ages	Chronic respiratory diseases	Rate	2021	545
Incidence	Mauritius	Both	All ages	Chronic respiratory diseases	Rate	2021	565
Incidence	Palestine	Both	All ages	Chronic respiratory diseases	Rate	2021	720

Incidence	United Arab Emirates	Both	All ages	Chronic respiratory diseases	Rate	2021	883
Incidence	Eritrea	Both	All ages	Chronic respiratory diseases	Rate	2021	900
Incidence	Bangladesh	Both	All ages	Chronic respiratory diseases	Rate	2021	500
Incidence	Botswana	Both	All ages	Chronic respiratory diseases	Rate	2021	660
Incidence	Rwanda	Both	All ages	Chronic respiratory diseases	Rate	2021	1537
Incidence	Somalia	Both	All ages	Chronic respiratory diseases	Rate	2021	1255
Incidence	Gabon	Both	All ages	Chronic respiratory diseases	Rate	2021	579
Incidence	India	Both	All ages	Chronic respiratory diseases	Rate	2021	572
Incidence	Angola	Both	All ages	Chronic respiratory diseases	Rate	2021	680
Incidence	Benin	Both	All ages	Chronic respiratory diseases	Rate	2021	737
Incidence	Malawi	Both	All ages	Chronic respiratory diseases	Rate	2021	899
Incidence	Kenya	Both	All ages	Chronic respiratory diseases	Rate	2021	576
Incidence	Burundi	Both	All ages	Chronic respiratory diseases	Rate	2021	1122
Incidence	Namibia	Both	All ages	Chronic respiratory diseases	Rate	2021	554
Incidence	Cameroon	Both	All ages	Chronic respiratory diseases	Rate	2021	442
Incidence	Congo	Both	All ages	Chronic respiratory diseases	Rate	2021	675

Incidence	Ghana	Both	All ages	Chronic respiratory diseases	Rate	2021	486
Incidence	Eswatini	Both	All ages	Chronic respiratory diseases	Rate	2021	1002
Incidence	Djibouti	Both	All ages	Chronic respiratory diseases	Rate	2021	727
Incidence	Uganda	Both	All ages	Chronic respiratory diseases	Rate	2021	1215
Incidence	Pakistan	Both	All ages	Chronic respiratory diseases	Rate	2021	414
Incidence	Seychelles	Both	All ages	Chronic respiratory diseases	Rate	2021	461
Incidence	Ethiopia	Both	All ages	Chronic respiratory diseases	Rate	2021	603
Incidence	Burkina Faso	Both	All ages	Chronic respiratory diseases	Rate	2021	899
Incidence	Mozambique	Both	All ages	Chronic respiratory diseases	Rate	2021	888
Incidence	Chad	Both	All ages	Chronic respiratory diseases	Rate	2021	691
Incidence	Equatorial Guinea	Both	All ages	Chronic respiratory diseases	Rate	2021	570
Incidence	Guinea-Bissau	Both	All ages	Chronic respiratory diseases	Rate	2021	703
Incidence	Gambia	Both	All ages	Chronic respiratory diseases	Rate	2021	612
Incidence	Lesotho	Both	All ages	Chronic respiratory diseases	Rate	2021	378
Incidence	Senegal	Both	All ages	Chronic respiratory diseases	Rate	2021	528
Incidence	Madagascar	Both	All ages	Chronic respiratory diseases	Rate	2021	1310

Incidence	Mali	Both	All ages	Chronic respiratory diseases	Rate	2021	553
Incidence	United Republic of Tanzania	Both	All ages	Chronic respiratory diseases	Rate	2021	1346
Incidence	Niger	Both	All ages	Chronic respiratory diseases	Rate	2021	916
Incidence	Togo	Both	All ages	Chronic respiratory diseases	Rate	2021	727
Incidence	South Africa	Both	All ages	Chronic respiratory diseases	Rate	2021	428
Incidence	Guinea	Both	All ages	Chronic respiratory diseases	Rate	2021	816
Incidence	Zimbabwe	Both	All ages	Chronic respiratory diseases	Rate	2021	483
Incidence	Greenland	Both	All ages	Chronic respiratory diseases	Rate	2021	1202
Incidence	Northern Mariana Islands	Both	All ages	Chronic respiratory diseases	Rate	2021	416
Incidence	Cabo Verde	Both	All ages	Chronic respiratory diseases	Rate	2021	437
Incidence	Sao Tome and Principe	Both	All ages	Chronic respiratory diseases	Rate	2021	729
Incidence	Puerto Rico	Both	All ages	Chronic respiratory diseases	Rate	2021	1099
Incidence	Liberia	Both	All ages	Chronic respiratory diseases	Rate	2021	567
Incidence	American Samoa	Both	All ages	Chronic respiratory diseases	Rate	2021	433
Incidence	San Marino	Both	All ages	Chronic respiratory diseases	Rate	2021	667
Incidence	Sierra Leone	Both	All ages	Chronic respiratory diseases	Rate	2021	755

Incidence	Monaco	Both	All ages	Chronic respiratory diseases	Rate	2021	734
Incidence	Cook Islands	Both	All ages	Chronic respiratory diseases	Rate	2021	492
Incidence	Niue	Both	All ages	Chronic respiratory diseases	Rate	2021	478
Incidence	CÃ´te d'Ivoire	Both	All ages	Chronic respiratory diseases	Rate	2021	721
Incidence	Mauritania	Both	All ages	Chronic respiratory diseases	Rate	2021	949
Incidence	Tuvalu	Both	All ages	Chronic respiratory diseases	Rate	2021	491
Incidence	Guam	Both	All ages	Chronic respiratory diseases	Rate	2021	444
Incidence	Nigeria	Both	All ages	Chronic respiratory diseases	Rate	2021	993
Incidence	Palau	Both	All ages	Chronic respiratory diseases	Rate	2021	445
Incidence	Saint Kitts and Nevis	Both	All ages	Chronic respiratory diseases	Rate	2021	793
Incidence	Bermuda	Both	All ages	Chronic respiratory diseases	Rate	2021	844
Incidence	South Sudan	Both	All ages	Chronic respiratory diseases	Rate	2021	1097
Incidence	Tokelau	Both	All ages	Chronic respiratory diseases	Rate	2021	495
Incidence	Nauru	Both	All ages	Chronic respiratory diseases	Rate	2021	479
Incidence	United States Virgin Islands	Both	All ages	Chronic respiratory diseases	Rate	2021	942
Incidence	Sudan	Both	All ages	Chronic respiratory diseases	Rate	2021	574
measure	Country	sex	age	cause	metric	year	Rate

Deaths	Luxembourg	Both	All ages	Chronic respiratory diseases	Rate	2021	39
Deaths	Slovakia	Both	All ages	Chronic respiratory diseases	Rate	2021	19
Deaths	Seychelles	Both	All ages	Chronic respiratory diseases	Rate	2021	28
Deaths	Fiji	Both	All ages	Chronic respiratory diseases	Rate	2021	47
Deaths	Syrian Arab Republic	Both	All ages	Chronic respiratory diseases	Rate	2021	26
Deaths	Slovenia	Both	All ages	Chronic respiratory diseases	Rate	2021	34
Deaths	Bahamas	Both	All ages	Chronic respiratory diseases	Rate	2021	15
Deaths	Turkmenistan	Both	All ages	Chronic respiratory diseases	Rate	2021	10
Deaths	Niue	Both	All ages	Chronic respiratory diseases	Rate	2021	76
Deaths	Malta	Both	All ages	Chronic respiratory diseases	Rate	2021	37
Deaths	Uzbekistan	Both	All ages	Chronic respiratory diseases	Rate	2021	8
Deaths	Taiwan	Both	All ages	Chronic respiratory diseases	Rate	2021	39
Deaths	Northern Mariana Islands	Both	All ages	Chronic respiratory diseases	Rate	2021	41
Deaths	Spain	Both	All ages	Chronic respiratory diseases	Rate	2021	81
Deaths	Zambia	Both	All ages	Chronic respiratory diseases	Rate	2021	13
Deaths	Papua New Guinea	Both	All ages	Chronic respiratory diseases	Rate	2021	69

Deaths	Algeria	Both	All ages	Chronic respiratory diseases	Rate	2021	18
Deaths	Netherlands	Both	All ages	Chronic respiratory diseases	Rate	2021	69
Deaths	Equatorial Guinea	Both	All ages	Chronic respiratory diseases	Rate	2021	9
Deaths	Colombia	Both	All ages	Chronic respiratory diseases	Rate	2021	39
Deaths	Kiribati	Both	All ages	Chronic respiratory diseases	Rate	2021	49
Deaths	Tajikistan	Both	All ages	Chronic respiratory diseases	Rate	2021	18
Deaths	Somalia	Both	All ages	Chronic respiratory diseases	Rate	2021	15
Deaths	Latvia	Both	All ages	Chronic respiratory diseases	Rate	2021	18
Deaths	Gambia	Both	All ages	Chronic respiratory diseases	Rate	2021	17
Deaths	Dominican Republic	Both	All ages	Chronic respiratory diseases	Rate	2021	16
Deaths	San Marino	Both	All ages	Chronic respiratory diseases	Rate	2021	24
Deaths	New Zealand	Both	All ages	Chronic respiratory diseases	Rate	2021	48
Deaths	Lao People's Democratic Republic	Both	All ages	Chronic respiratory diseases	Rate	2021	36
Deaths	Democratic People's Republic of Korea	Both	All ages	Chronic respiratory diseases	Rate	2021	121
Deaths	Bulgaria	Both	All ages	Chronic respiratory diseases	Rate	2021	35
Deaths	Tunisia	Both	All ages	Chronic respiratory diseases	Rate	2021	25

Deaths	Argentina	Both	All ages	Chronic respiratory diseases	Rate	2021	38
Deaths	Antigua and Barbuda	Both	All ages	Chronic respiratory diseases	Rate	2021	12
Deaths	Bhutan	Both	All ages	Chronic respiratory diseases	Rate	2021	74
Deaths	Denmark	Both	All ages	Chronic respiratory diseases	Rate	2021	90
Deaths	Democratic Republic of the Congo	Both	All ages	Chronic respiratory diseases	Rate	2021	22
Deaths	Bolivia (Plurinational State of)	Both	All ages	Chronic respiratory diseases	Rate	2021	24
Deaths	Andorra	Both	All ages	Chronic respiratory diseases	Rate	2021	41
Deaths	South Africa	Both	All ages	Chronic respiratory diseases	Rate	2021	35
Deaths	Cabo Verde	Both	All ages	Chronic respiratory diseases	Rate	2021	18
Deaths	Chad	Both	All ages	Chronic respiratory diseases	Rate	2021	15
Deaths	Jamaica	Both	All ages	Chronic respiratory diseases	Rate	2021	21
Deaths	Nauru	Both	All ages	Chronic respiratory diseases	Rate	2021	40
Deaths	Comoros	Both	All ages	Chronic respiratory diseases	Rate	2021	21
Deaths	Estonia	Both	All ages	Chronic respiratory diseases	Rate	2021	17
Deaths	Lithuania	Both	All ages	Chronic respiratory diseases	Rate	2021	22
Deaths	Barbados	Both	All ages	Chronic respiratory diseases	Rate	2021	21

Deaths	Honduras	Both	All ages	Chronic respiratory diseases	Rate	2021	34
Deaths	Austria	Both	All ages	Chronic respiratory diseases	Rate	2021	41
Deaths	Jordan	Both	All ages	Chronic respiratory diseases	Rate	2021	7
Deaths	Djibouti	Both	All ages	Chronic respiratory diseases	Rate	2021	11
Deaths	Bahrain	Both	All ages	Chronic respiratory diseases	Rate	2021	13
Deaths	Saint Lucia	Both	All ages	Chronic respiratory diseases	Rate	2021	39
Deaths	United Arab Emirates	Both	All ages	Chronic respiratory diseases	Rate	2021	8
Deaths	Canada	Both	All ages	Chronic respiratory diseases	Rate	2021	50
Deaths	Liberia	Both	All ages	Chronic respiratory diseases	Rate	2021	14
Deaths	Micronesia (Federated States of)	Both	All ages	Chronic respiratory diseases	Rate	2021	46
Deaths	Congo	Both	All ages	Chronic respiratory diseases	Rate	2021	19
Deaths	Marshall Islands	Both	All ages	Chronic respiratory diseases	Rate	2021	40
Deaths	Samoa	Both	All ages	Chronic respiratory diseases	Rate	2021	50
Deaths	Ecuador	Both	All ages	Chronic respiratory diseases	Rate	2021	21
Deaths	Sweden	Both	All ages	Chronic respiratory diseases	Rate	2021	43
Deaths	American Samoa	Both	All ages	Chronic respiratory diseases	Rate	2021	46

Deaths	Solomon Islands	Both	All ages	Chronic respiratory diseases	Rate	2021	32
Deaths	United States of America	Both	All ages	Chronic respiratory diseases	Rate	2021	70
Deaths	Mexico	Both	All ages	Chronic respiratory diseases	Rate	2021	29
Deaths	Monaco	Both	All ages	Chronic respiratory diseases	Rate	2021	47
Deaths	Norway	Both	All ages	Chronic respiratory diseases	Rate	2021	59
Deaths	Gabon	Both	All ages	Chronic respiratory diseases	Rate	2021	17
Deaths	Iran (Islamic Republic of)	Both	All ages	Chronic respiratory diseases	Rate	2021	18
Deaths	Ghana	Both	All ages	Chronic respiratory diseases	Rate	2021	10
Deaths	CÃ´te d'Ivoire	Both	All ages	Chronic respiratory diseases	Rate	2021	13
Deaths	Sri Lanka	Both	All ages	Chronic respiratory diseases	Rate	2021	60
Deaths	United Republic of Tanzania	Both	All ages	Chronic respiratory diseases	Rate	2021	12
Deaths	Indonesia	Both	All ages	Chronic respiratory diseases	Rate	2021	41
Deaths	Belarus	Both	All ages	Chronic respiratory diseases	Rate	2021	15
Deaths	Kazakhstan	Both	All ages	Chronic respiratory diseases	Rate	2021	48
Deaths	Montenegro	Both	All ages	Chronic respiratory diseases	Rate	2021	10
Deaths	Uganda	Both	All ages	Chronic respiratory diseases	Rate	2021	12

Deaths	TÃ¼rkiye	Both	All ages	Chronic respiratory diseases	Rate	2021	45
Deaths	Burkina Faso	Both	All ages	Chronic respiratory diseases	Rate	2021	11
Deaths	Madagascar	Both	All ages	Chronic respiratory diseases	Rate	2021	25
Deaths	Iceland	Both	All ages	Chronic respiratory diseases	Rate	2021	37
Deaths	Botswana	Both	All ages	Chronic respiratory diseases	Rate	2021	25
Deaths	Morocco	Both	All ages	Chronic respiratory diseases	Rate	2021	27
Deaths	Costa Rica	Both	All ages	Chronic respiratory diseases	Rate	2021	29
Deaths	Republic of Korea	Both	All ages	Chronic respiratory diseases	Rate	2021	31
Deaths	Yemen	Both	All ages	Chronic respiratory diseases	Rate	2021	16
Deaths	Portugal	Both	All ages	Chronic respiratory diseases	Rate	2021	64
Deaths	Cyprus	Both	All ages	Chronic respiratory diseases	Rate	2021	44
Deaths	Singapore	Both	All ages	Chronic respiratory diseases	Rate	2021	11
Deaths	Bosnia and Herzegovina	Both	All ages	Chronic respiratory diseases	Rate	2021	35
Deaths	Malaysia	Both	All ages	Chronic respiratory diseases	Rate	2021	26
Deaths	Palau	Both	All ages	Chronic respiratory diseases	Rate	2021	76
Deaths	Peru	Both	All ages	Chronic respiratory diseases	Rate	2021	23

Deaths	Cambodia	Both	All ages	Chronic respiratory diseases	Rate	2021	32
Deaths	Kuwait	Both	All ages	Chronic respiratory diseases	Rate	2021	3
Deaths	Iraq	Both	All ages	Chronic respiratory diseases	Rate	2021	8
Deaths	Burundi	Both	All ages	Chronic respiratory diseases	Rate	2021	16
Deaths	Belize	Both	All ages	Chronic respiratory diseases	Rate	2021	18
Deaths	Maldives	Both	All ages	Chronic respiratory diseases	Rate	2021	22
Deaths	Sao Tome and Principe	Both	All ages	Chronic respiratory diseases	Rate	2021	38
Deaths	Finland	Both	All ages	Chronic respiratory diseases	Rate	2021	38
Deaths	Croatia	Both	All ages	Chronic respiratory diseases	Rate	2021	49
Deaths	Grenada	Both	All ages	Chronic respiratory diseases	Rate	2021	19
Deaths	Albania	Both	All ages	Chronic respiratory diseases	Rate	2021	32
Deaths	Guatemala	Both	All ages	Chronic respiratory diseases	Rate	2021	14
Deaths	Belgium	Both	All ages	Chronic respiratory diseases	Rate	2021	59
Deaths	Dominica	Both	All ages	Chronic respiratory diseases	Rate	2021	28
Deaths	Chile	Both	All ages	Chronic respiratory diseases	Rate	2021	36
Deaths	Egypt	Both	All ages	Chronic respiratory diseases	Rate	2021	14

Deaths	Cuba	Both	All ages	Chronic respiratory diseases	Rate	2021	45
Deaths	Eswatini	Both	All ages	Chronic respiratory diseases	Rate	2021	33
Deaths	Tokelau	Both	All ages	Chronic respiratory diseases	Rate	2021	66
Deaths	Tonga	Both	All ages	Chronic respiratory diseases	Rate	2021	45
Deaths	Afghanistan	Both	All ages	Chronic respiratory diseases	Rate	2021	20
Deaths	Bangladesh	Both	All ages	Chronic respiratory diseases	Rate	2021	54
Deaths	Saint Kitts and Nevis	Both	All ages	Chronic respiratory diseases	Rate	2021	20
Deaths	Zimbabwe	Both	All ages	Chronic respiratory diseases	Rate	2021	20
Deaths	India	Both	All ages	Chronic respiratory diseases	Rate	2021	94
Deaths	Guinea-Bissau	Both	All ages	Chronic respiratory diseases	Rate	2021	18
Deaths	Switzerland	Both	All ages	Chronic respiratory diseases	Rate	2021	34
Deaths	Ukraine	Both	All ages	Chronic respiratory diseases	Rate	2021	18
Deaths	North Macedonia	Both	All ages	Chronic respiratory diseases	Rate	2021	29
Deaths	Guinea	Both	All ages	Chronic respiratory diseases	Rate	2021	18
Deaths	Germany	Both	All ages	Chronic respiratory diseases	Rate	2021	55
Deaths	Republic of Moldova	Both	All ages	Chronic respiratory diseases	Rate	2021	20

Deaths	Vanuatu	Both	All ages	Chronic respiratory diseases	Rate	2021	46
Deaths	El Salvador	Both	All ages	Chronic respiratory diseases	Rate	2021	24
Deaths	Lesotho	Both	All ages	Chronic respiratory diseases	Rate	2021	49
Deaths	United Kingdom	Both	All ages	Chronic respiratory diseases	Rate	2021	74
Deaths	Puerto Rico	Both	All ages	Chronic respiratory diseases	Rate	2021	58
Deaths	Mali	Both	All ages	Chronic respiratory diseases	Rate	2021	23
Deaths	Palestine	Both	All ages	Chronic respiratory diseases	Rate	2021	9
Deaths	Libya	Both	All ages	Chronic respiratory diseases	Rate	2021	19
Deaths	Namibia	Both	All ages	Chronic respiratory diseases	Rate	2021	34
Deaths	Nicaragua	Both	All ages	Chronic respiratory diseases	Rate	2021	13
Deaths	Eritrea	Both	All ages	Chronic respiratory diseases	Rate	2021	17
Deaths	Greece	Both	All ages	Chronic respiratory diseases	Rate	2021	55
Deaths	Mauritania	Both	All ages	Chronic respiratory diseases	Rate	2021	12
Deaths	Haiti	Both	All ages	Chronic respiratory diseases	Rate	2021	27
Deaths	Russian Federation	Both	All ages	Chronic respiratory diseases	Rate	2021	24
Deaths	Nepal	Both	All ages	Chronic respiratory diseases	Rate	2021	110

Deaths	Philippines	Both	All ages	Chronic respiratory diseases	Rate	2021	29
Deaths	Ireland	Both	All ages	Chronic respiratory diseases	Rate	2021	50
Deaths	Saint Vincent and the Grenadines	Both	All ages	Chronic respiratory diseases	Rate	2021	19
Deaths	Czechia	Both	All ages	Chronic respiratory diseases	Rate	2021	42
Deaths	Oman	Both	All ages	Chronic respiratory diseases	Rate	2021	6
Deaths	Kenya	Both	All ages	Chronic respiratory diseases	Rate	2021	17
Deaths	Bermuda	Both	All ages	Chronic respiratory diseases	Rate	2021	32
Deaths	Serbia	Both	All ages	Chronic respiratory diseases	Rate	2021	44
Deaths	Venezuela (Bolivarian Republic of)	Both	All ages	Chronic respiratory diseases	Rate	2021	26
Deaths	Myanmar	Both	All ages	Chronic respiratory diseases	Rate	2021	93
Deaths	France	Both	All ages	Chronic respiratory diseases	Rate	2021	32
Deaths	Poland	Both	All ages	Chronic respiratory diseases	Rate	2021	28
Deaths	Thailand	Both	All ages	Chronic respiratory diseases	Rate	2021	39
Deaths	Kyrgyzstan	Both	All ages	Chronic respiratory diseases	Rate	2021	17
Deaths	Cameroon	Both	All ages	Chronic respiratory diseases	Rate	2021	13
Deaths	Nigeria	Both	All ages	Chronic respiratory diseases	Rate	2021	9

Deaths	Sudan	Both	All ages	Chronic respiratory diseases	Rate	2021	15
Deaths	Hungary	Both	All ages	Chronic respiratory diseases	Rate	2021	66
Deaths	Japan	Both	All ages	Chronic respiratory diseases	Rate	2021	47
Deaths	Guyana	Both	All ages	Chronic respiratory diseases	Rate	2021	16
Deaths	Azerbaijan	Both	All ages	Chronic respiratory diseases	Rate	2021	16
Deaths	Senegal	Both	All ages	Chronic respiratory diseases	Rate	2021	17
Deaths	Lebanon	Both	All ages	Chronic respiratory diseases	Rate	2021	29
Deaths	Uruguay	Both	All ages	Chronic respiratory diseases	Rate	2021	70
Deaths	Niger	Both	All ages	Chronic respiratory diseases	Rate	2021	12
Deaths	South Sudan	Both	All ages	Chronic respiratory diseases	Rate	2021	19
Deaths	Viet Nam	Both	All ages	Chronic respiratory diseases	Rate	2021	43
Deaths	Ethiopia	Both	All ages	Chronic respiratory diseases	Rate	2021	12
Deaths	Brunei Darussalam	Both	All ages	Chronic respiratory diseases	Rate	2021	21
Deaths	Pakistan	Both	All ages	Chronic respiratory diseases	Rate	2021	35
Deaths	Brazil	Both	All ages	Chronic respiratory diseases	Rate	2021	33
Deaths	Georgia	Both	All ages	Chronic respiratory diseases	Rate	2021	24

Deaths	Italy	Both	All ages	Chronic respiratory diseases	Rate	2021	55
Deaths	Armenia	Both	All ages	Chronic respiratory diseases	Rate	2021	25
Deaths	Malawi	Both	All ages	Chronic respiratory diseases	Rate	2021	14
Deaths	Benin	Both	All ages	Chronic respiratory diseases	Rate	2021	13
Deaths	Paraguay	Both	All ages	Chronic respiratory diseases	Rate	2021	16
Deaths	China	Both	All ages	Chronic respiratory diseases	Rate	2021	94
Deaths	Saudi Arabia	Both	All ages	Chronic respiratory diseases	Rate	2021	13
Deaths	Angola	Both	All ages	Chronic respiratory diseases	Rate	2021	12
Deaths	Guam	Both	All ages	Chronic respiratory diseases	Rate	2021	24
Deaths	Cook Islands	Both	All ages	Chronic respiratory diseases	Rate	2021	43
Deaths	Timor-Leste	Both	All ages	Chronic respiratory diseases	Rate	2021	35
Deaths	Rwanda	Both	All ages	Chronic respiratory diseases	Rate	2021	18
Deaths	Togo	Both	All ages	Chronic respiratory diseases	Rate	2021	17
Deaths	Greenland	Both	All ages	Chronic respiratory diseases	Rate	2021	48
Deaths	Trinidad and Tobago	Both	All ages	Chronic respiratory diseases	Rate	2021	22
Deaths	Israel	Both	All ages	Chronic respiratory diseases	Rate	2021	21

Deaths	Tuvalu	Both	All ages	Chronic respiratory diseases	Rate	2021	50
Deaths	Romania	Both	All ages	Chronic respiratory diseases	Rate	2021	40
Deaths	Panama	Both	All ages	Chronic respiratory diseases	Rate	2021	21
Deaths	Central African Republic	Both	All ages	Chronic respiratory diseases	Rate	2021	27
Deaths	United States Virgin Islands	Both	All ages	Chronic respiratory diseases	Rate	2021	22
Deaths	Sierra Leone	Both	All ages	Chronic respiratory diseases	Rate	2021	15
Deaths	Qatar	Both	All ages	Chronic respiratory diseases	Rate	2021	3
Deaths	Suriname	Both	All ages	Chronic respiratory diseases	Rate	2021	21
Deaths	Mongolia	Both	All ages	Chronic respiratory diseases	Rate	2021	13
Deaths	Mauritius	Both	All ages	Chronic respiratory diseases	Rate	2021	44
Deaths	Australia	Both	All ages	Chronic respiratory diseases	Rate	2021	46
Deaths	Mozambique	Both	All ages	Chronic respiratory diseases	Rate	2021	13
measure	Country	sex	age	cause	metric	year	Rate
DALYs (Disability-Adjusted Life Years)	Brunei Darussalam	Both	All ages	Chronic respiratory diseases	Rate	2021	680
DALYs (Disability-Adjusted Life Years)	Federal Democratic Republic of Ethiopia	Both	All ages	Chronic respiratory diseases	Rate	2021	512
DALYs (Disability-Adjusted Life Years)	Mongolia	Both	All ages	Chronic respiratory diseases	Rate	2021	459

DALYs (Disability-Adjusted Life Years)	Kingdom of Denmark	Both	All ages	Chronic respiratory diseases	Rate	2021	1780
DALYs (Disability-Adjusted Life Years)	Independent State of Papua New Guinea	Both	All ages	Chronic respiratory diseases	Rate	2021	2078
DALYs (Disability-Adjusted Life Years)	Kingdom of Norway	Both	All ages	Chronic respiratory diseases	Rate	2021	1332
DALYs (Disability-Adjusted Life Years)	Montenegro	Both	All ages	Chronic respiratory diseases	Rate	2021	441
DALYs (Disability-Adjusted Life Years)	Republic of Chile	Both	All ages	Chronic respiratory diseases	Rate	2021	914
DALYs (Disability-Adjusted Life Years)	Republic of Senegal	Both	All ages	Chronic respiratory diseases	Rate	2021	629
DALYs (Disability-Adjusted Life Years)	Principality of Andorra	Both	All ages	Chronic respiratory diseases	Rate	2021	1102
DALYs (Disability-Adjusted Life Years)	Republic of Chad	Both	All ages	Chronic respiratory diseases	Rate	2021	648
DALYs (Disability-Adjusted Life Years)	Republic of Haiti	Both	All ages	Chronic respiratory diseases	Rate	2021	1550
DALYs (Disability-Adjusted Life Years)	United States of America	Both	All ages	Chronic respiratory diseases	Rate	2021	2010
DALYs (Disability-Adjusted Life Years)	Republic of Maldives	Both	All ages	Chronic respiratory diseases	Rate	2021	630
DALYs (Disability-Adjusted Life Years)	Republic of Italy	Both	All ages	Chronic respiratory diseases	Rate	2021	1077

DALYs (Disability-Adjusted Life Years)	Russian Federation	Both	All ages	Chronic respiratory diseases	Rate	2021	711
DALYs (Disability-Adjusted Life Years)	Republic of Costa Rica	Both	All ages	Chronic respiratory diseases	Rate	2021	794
DALYs (Disability-Adjusted Life Years)	Commonwealth of Dominica	Both	All ages	Chronic respiratory diseases	Rate	2021	926
DALYs (Disability-Adjusted Life Years)	Republic of Palau	Both	All ages	Chronic respiratory diseases	Rate	2021	2063
DALYs (Disability-Adjusted Life Years)	Sultanate of Oman	Both	All ages	Chronic respiratory diseases	Rate	2021	365
DALYs (Disability-Adjusted Life Years)	Republic of Ecuador	Both	All ages	Chronic respiratory diseases	Rate	2021	564
DALYs (Disability-Adjusted Life Years)	Taiwan (Province of China)	Both	All ages	Chronic respiratory diseases	Rate	2021	920
DALYs (Disability-Adjusted Life Years)	Republic of the Congo	Both	All ages	Chronic respiratory diseases	Rate	2021	757
DALYs (Disability-Adjusted Life Years)	Republic of Colombia	Both	All ages	Chronic respiratory diseases	Rate	2021	868
DALYs (Disability-Adjusted Life Years)	Republic of Zimbabwe	Both	All ages	Chronic respiratory diseases	Rate	2021	762
DALYs (Disability-Adjusted Life Years)	Republic of Rwanda	Both	All ages	Chronic respiratory diseases	Rate	2021	1002
DALYs (Disability-Adjusted Life Years)	Kingdom of Bhutan	Both	All ages	Chronic respiratory diseases	Rate	2021	1625

DALYs (Disability-Adjusted Life Years)	Republic of Seychelles	Both	All ages	Chronic respiratory diseases	Rate	2021	785
DALYs (Disability-Adjusted Life Years)	Socialist Republic of Viet Nam	Both	All ages	Chronic respiratory diseases	Rate	2021	1074
DALYs (Disability-Adjusted Life Years)	Republic of Korea	Both	All ages	Chronic respiratory diseases	Rate	2021	806
DALYs (Disability-Adjusted Life Years)	Lebanese Republic	Both	All ages	Chronic respiratory diseases	Rate	2021	830
DALYs (Disability-Adjusted Life Years)	Palestine	Both	All ages	Chronic respiratory diseases	Rate	2021	435
DALYs (Disability-Adjusted Life Years)	Bosnia and Herzegovina	Both	All ages	Chronic respiratory diseases	Rate	2021	1030
DALYs (Disability-Adjusted Life Years)	People's Republic of Bangladesh	Both	All ages	Chronic respiratory diseases	Rate	2021	1411
DALYs (Disability-Adjusted Life Years)	Democratic People's Republic of Korea	Both	All ages	Chronic respiratory diseases	Rate	2021	2617
DALYs (Disability-Adjusted Life Years)	Republic of Liberia	Both	All ages	Chronic respiratory diseases	Rate	2021	589
DALYs (Disability-Adjusted Life Years)	French Republic	Both	All ages	Chronic respiratory diseases	Rate	2021	855
DALYs (Disability-Adjusted Life Years)	Republic of Guyana	Both	All ages	Chronic respiratory diseases	Rate	2021	724
DALYs (Disability-Adjusted Life Years)	Gabonese Republic	Both	All ages	Chronic respiratory diseases	Rate	2021	608

DALYs (Disability-Adjusted Life Years)	Republic of Armenia	Both	All ages	Chronic respiratory diseases	Rate	2021	611
DALYs (Disability-Adjusted Life Years)	Republic of Mali	Both	All ages	Chronic respiratory diseases	Rate	2021	881
DALYs (Disability-Adjusted Life Years)	Kingdom of Spain	Both	All ages	Chronic respiratory diseases	Rate	2021	1433
DALYs (Disability-Adjusted Life Years)	Republic of Indonesia	Both	All ages	Chronic respiratory diseases	Rate	2021	1235
DALYs (Disability-Adjusted Life Years)	Czech Republic	Both	All ages	Chronic respiratory diseases	Rate	2021	1067
DALYs (Disability-Adjusted Life Years)	Republic of Kiribati	Both	All ages	Chronic respiratory diseases	Rate	2021	1647
DALYs (Disability-Adjusted Life Years)	United States Virgin Islands	Both	All ages	Chronic respiratory diseases	Rate	2021	753
DALYs (Disability-Adjusted Life Years)	Republic of Guatemala	Both	All ages	Chronic respiratory diseases	Rate	2021	491
DALYs (Disability-Adjusted Life Years)	Grenada	Both	All ages	Chronic respiratory diseases	Rate	2021	817
DALYs (Disability-Adjusted Life Years)	Kingdom of Eswatini	Both	All ages	Chronic respiratory diseases	Rate	2021	1285
DALYs (Disability-Adjusted Life Years)	United Republic of Tanzania	Both	All ages	Chronic respiratory diseases	Rate	2021	724
DALYs (Disability-Adjusted Life Years)	Republic of Paraguay	Both	All ages	Chronic respiratory diseases	Rate	2021	586

DALYs (Disability-Adjusted Life Years)	Kingdom of Saudi Arabia	Both	All ages	Chronic respiratory diseases	Rate	2021	562
DALYs (Disability-Adjusted Life Years)	Saint Lucia	Both	All ages	Chronic respiratory diseases	Rate	2021	1134
DALYs (Disability-Adjusted Life Years)	Republic of Equatorial Guinea	Both	All ages	Chronic respiratory diseases	Rate	2021	433
DALYs (Disability-Adjusted Life Years)	Republic of Mozambique	Both	All ages	Chronic respiratory diseases	Rate	2021	657
DALYs (Disability-Adjusted Life Years)	Kingdom of Morocco	Both	All ages	Chronic respiratory diseases	Rate	2021	806
DALYs (Disability-Adjusted Life Years)	Kingdom of Bahrain	Both	All ages	Chronic respiratory diseases	Rate	2021	523
DALYs (Disability-Adjusted Life Years)	Burkina Faso	Both	All ages	Chronic respiratory diseases	Rate	2021	620
DALYs (Disability-Adjusted Life Years)	Republic of Iceland	Both	All ages	Chronic respiratory diseases	Rate	2021	1068
DALYs (Disability-Adjusted Life Years)	Federal Democratic Republic of Nepal	Both	All ages	Chronic respiratory diseases	Rate	2021	2608
DALYs (Disability-Adjusted Life Years)	Kingdom of Cambodia	Both	All ages	Chronic respiratory diseases	Rate	2021	922
DALYs (Disability-Adjusted Life Years)	People's Democratic Republic of Algeria	Both	All ages	Chronic respiratory diseases	Rate	2021	610
DALYs (Disability-Adjusted Life Years)	Republic of Slovenia	Both	All ages	Chronic respiratory diseases	Rate	2021	920

DALYs (Disability-Adjusted Life Years)	Republic of Latvia	Both	All ages	Chronic respiratory diseases	Rate	2021	613
DALYs (Disability-Adjusted Life Years)	Guam	Both	All ages	Chronic respiratory diseases	Rate	2021	776
DALYs (Disability-Adjusted Life Years)	Republic of Fiji	Both	All ages	Chronic respiratory diseases	Rate	2021	1364
DALYs (Disability-Adjusted Life Years)	Republic of El Salvador	Both	All ages	Chronic respiratory diseases	Rate	2021	718
DALYs (Disability-Adjusted Life Years)	Republic of Azerbaijan	Both	All ages	Chronic respiratory diseases	Rate	2021	537
DALYs (Disability-Adjusted Life Years)	Republic of Burundi	Both	All ages	Chronic respiratory diseases	Rate	2021	789
DALYs (Disability-Adjusted Life Years)	Republic of the Niger	Both	All ages	Chronic respiratory diseases	Rate	2021	617
DALYs (Disability-Adjusted Life Years)	Georgia	Both	All ages	Chronic respiratory diseases	Rate	2021	662
DALYs (Disability-Adjusted Life Years)	State of Qatar	Both	All ages	Chronic respiratory diseases	Rate	2021	257
DALYs (Disability-Adjusted Life Years)	Republic of India	Both	All ages	Chronic respiratory diseases	Rate	2021	2261
DALYs (Disability-Adjusted Life Years)	Republic of Albania	Both	All ages	Chronic respiratory diseases	Rate	2021	811
DALYs (Disability-Adjusted Life Years)	Republic of Croatia	Both	All ages	Chronic respiratory diseases	Rate	2021	1131

DALYs (Disability-Adjusted Life Years)	Republic of South Sudan	Both	All ages	Chronic respiratory diseases	Rate	2021	905
DALYs (Disability-Adjusted Life Years)	Federal Republic of Somalia	Both	All ages	Chronic respiratory diseases	Rate	2021	859
DALYs (Disability-Adjusted Life Years)	Republic of Benin	Both	All ages	Chronic respiratory diseases	Rate	2021	589
DALYs (Disability-Adjusted Life Years)	Union of the Comoros	Both	All ages	Chronic respiratory diseases	Rate	2021	816
DALYs (Disability-Adjusted Life Years)	Islamic Republic of Mauritania	Both	All ages	Chronic respiratory diseases	Rate	2021	587
DALYs (Disability-Adjusted Life Years)	Democratic Republic of Timor-Leste	Both	All ages	Chronic respiratory diseases	Rate	2021	1044
DALYs (Disability-Adjusted Life Years)	Australia	Both	All ages	Chronic respiratory diseases	Rate	2021	1137
DALYs (Disability-Adjusted Life Years)	Jamaica	Both	All ages	Chronic respiratory diseases	Rate	2021	723
DALYs (Disability-Adjusted Life Years)	Republic of Guinea-Bissau	Both	All ages	Chronic respiratory diseases	Rate	2021	800
DALYs (Disability-Adjusted Life Years)	Islamic Republic of Afghanistan	Both	All ages	Chronic respiratory diseases	Rate	2021	813
DALYs (Disability-Adjusted Life Years)	Republic of Tunisia	Both	All ages	Chronic respiratory diseases	Rate	2021	786
DALYs (Disability-Adjusted Life Years)	United Kingdom of Great Britain and Northern Ireland	Both	All ages	Chronic respiratory diseases	Rate	2021	1812

DALYs (Disability-Adjusted Life Years)	United Mexican States	Both	All ages	Chronic respiratory diseases	Rate	2021	703
DALYs (Disability-Adjusted Life Years)	Barbados	Both	All ages	Chronic respiratory diseases	Rate	2021	753
DALYs (Disability-Adjusted Life Years)	Republic of Belarus	Both	All ages	Chronic respiratory diseases	Rate	2021	616
DALYs (Disability-Adjusted Life Years)	Swiss Confederation	Both	All ages	Chronic respiratory diseases	Rate	2021	942
DALYs (Disability-Adjusted Life Years)	Republic of Estonia	Both	All ages	Chronic respiratory diseases	Rate	2021	508
DALYs (Disability-Adjusted Life Years)	Republic of Trinidad and Tobago	Both	All ages	Chronic respiratory diseases	Rate	2021	688
DALYs (Disability-Adjusted Life Years)	Republic of Iraq	Both	All ages	Chronic respiratory diseases	Rate	2021	415
DALYs (Disability-Adjusted Life Years)	Tuvalu	Both	All ages	Chronic respiratory diseases	Rate	2021	1388
DALYs (Disability-Adjusted Life Years)	Portuguese Republic	Both	All ages	Chronic respiratory diseases	Rate	2021	1469
DALYs (Disability-Adjusted Life Years)	Republic of Cabo Verde	Both	All ages	Chronic respiratory diseases	Rate	2021	549
DALYs (Disability-Adjusted Life Years)	Commonwealth of the Bahamas	Both	All ages	Chronic respiratory diseases	Rate	2021	619
DALYs (Disability-Adjusted Life Years)	Republic of Finland	Both	All ages	Chronic respiratory diseases	Rate	2021	1055

DALYs (Disability-Adjusted Life Years)	Lao People's Democratic Republic	Both	All ages	Chronic respiratory diseases	Rate	2021	1102
DALYs (Disability-Adjusted Life Years)	Japan	Both	All ages	Chronic respiratory diseases	Rate	2021	1006
DALYs (Disability-Adjusted Life Years)	Republic of Nauru	Both	All ages	Chronic respiratory diseases	Rate	2021	1328
DALYs (Disability-Adjusted Life Years)	Malaysia	Both	All ages	Chronic respiratory diseases	Rate	2021	754
DALYs (Disability-Adjusted Life Years)	Republic of Nicaragua	Both	All ages	Chronic respiratory diseases	Rate	2021	497
DALYs (Disability-Adjusted Life Years)	Principality of Monaco	Both	All ages	Chronic respiratory diseases	Rate	2021	1186
DALYs (Disability-Adjusted Life Years)	Antigua and Barbuda	Both	All ages	Chronic respiratory diseases	Rate	2021	508
DALYs (Disability-Adjusted Life Years)	Republic of Suriname	Both	All ages	Chronic respiratory diseases	Rate	2021	782
DALYs (Disability-Adjusted Life Years)	Republic of Bulgaria	Both	All ages	Chronic respiratory diseases	Rate	2021	999
DALYs (Disability-Adjusted Life Years)	Kyrgyz Republic	Both	All ages	Chronic respiratory diseases	Rate	2021	560
DALYs (Disability-Adjusted Life Years)	Republic of Moldova	Both	All ages	Chronic respiratory diseases	Rate	2021	634
DALYs (Disability-Adjusted Life Years)	Republic of Kazakhstan	Both	All ages	Chronic respiratory diseases	Rate	2021	1253

DALYs (Disability-Adjusted Life Years)	Kingdom of Sweden	Both	All ages	Chronic respiratory diseases	Rate	2021	1204
DALYs (Disability-Adjusted Life Years)	Republic of Turkey	Both	All ages	Chronic respiratory diseases	Rate	2021	1218
DALYs (Disability-Adjusted Life Years)	Federated States of Micronesia	Both	All ages	Chronic respiratory diseases	Rate	2021	1449
DALYs (Disability-Adjusted Life Years)	Republic of Zambia	Both	All ages	Chronic respiratory diseases	Rate	2021	550
DALYs (Disability-Adjusted Life Years)	State of Israel	Both	All ages	Chronic respiratory diseases	Rate	2021	608
DALYs (Disability-Adjusted Life Years)	Islamic Republic of Iran	Both	All ages	Chronic respiratory diseases	Rate	2021	597
DALYs (Disability-Adjusted Life Years)	New Zealand	Both	All ages	Chronic respiratory diseases	Rate	2021	1190
DALYs (Disability-Adjusted Life Years)	North Macedonia	Both	All ages	Chronic respiratory diseases	Rate	2021	949
DALYs (Disability-Adjusted Life Years)	Hungary	Both	All ages	Chronic respiratory diseases	Rate	2021	1680
DALYs (Disability-Adjusted Life Years)	State of Eritrea	Both	All ages	Chronic respiratory diseases	Rate	2021	814
DALYs (Disability-Adjusted Life Years)	Republic of Uganda	Both	All ages	Chronic respiratory diseases	Rate	2021	695
DALYs (Disability-Adjusted Life Years)	Republic of Singapore	Both	All ages	Chronic respiratory diseases	Rate	2021	401

DALYs (Disability-Adjusted Life Years)	Republic of Cameroon	Both	All ages	Chronic respiratory diseases	Rate	2021	550
DALYs (Disability-Adjusted Life Years)	Republic of Honduras	Both	All ages	Chronic respiratory diseases	Rate	2021	1024
DALYs (Disability-Adjusted Life Years)	Argentine Republic	Both	All ages	Chronic respiratory diseases	Rate	2021	992
DALYs (Disability-Adjusted Life Years)	Plurinational State of Bolivia	Both	All ages	Chronic respiratory diseases	Rate	2021	738
DALYs (Disability-Adjusted Life Years)	State of Kuwait	Both	All ages	Chronic respiratory diseases	Rate	2021	284
DALYs (Disability-Adjusted Life Years)	Republic of Lithuania	Both	All ages	Chronic respiratory diseases	Rate	2021	654
DALYs (Disability-Adjusted Life Years)	Solomon Islands	Both	All ages	Chronic respiratory diseases	Rate	2021	1027
DALYs (Disability-Adjusted Life Years)	Hellenic Republic	Both	All ages	Chronic respiratory diseases	Rate	2021	1291
DALYs (Disability-Adjusted Life Years)	Republic of Cuba	Both	All ages	Chronic respiratory diseases	Rate	2021	1207
DALYs (Disability-Adjusted Life Years)	Northern Mariana Islands	Both	All ages	Chronic respiratory diseases	Rate	2021	1148
DALYs (Disability-Adjusted Life Years)	Saint Vincent and the Grenadines	Both	All ages	Chronic respiratory diseases	Rate	2021	662
DALYs (Disability-Adjusted Life Years)	Republic of the Philippines	Both	All ages	Chronic respiratory diseases	Rate	2021	1047

DALYs (Disability-Adjusted Life Years)	Republic of the Marshall Islands	Both	All ages	Chronic respiratory diseases	Rate	2021	1373
DALYs (Disability-Adjusted Life Years)	Romania	Both	All ages	Chronic respiratory diseases	Rate	2021	1134
DALYs (Disability-Adjusted Life Years)	Republic of Austria	Both	All ages	Chronic respiratory diseases	Rate	2021	1064
DALYs (Disability-Adjusted Life Years)	Kingdom of Lesotho	Both	All ages	Chronic respiratory diseases	Rate	2021	1526
DALYs (Disability-Adjusted Life Years)	Republic of the Union of Myanmar	Both	All ages	Chronic respiratory diseases	Rate	2021	2205
DALYs (Disability-Adjusted Life Years)	Federal Republic of Germany	Both	All ages	Chronic respiratory diseases	Rate	2021	1317
DALYs (Disability-Adjusted Life Years)	Arab Republic of Egypt	Both	All ages	Chronic respiratory diseases	Rate	2021	554
DALYs (Disability-Adjusted Life Years)	Republic of Djibouti	Both	All ages	Chronic respiratory diseases	Rate	2021	542
DALYs (Disability-Adjusted Life Years)	Republic of Panama	Both	All ages	Chronic respiratory diseases	Rate	2021	630
DALYs (Disability-Adjusted Life Years)	Syrian Arab Republic	Both	All ages	Chronic respiratory diseases	Rate	2021	918
DALYs (Disability-Adjusted Life Years)	Democratic Republic of Sao Tome and Principe	Both	All ages	Chronic respiratory diseases	Rate	2021	1249
DALYs (Disability-Adjusted Life Years)	Ukraine	Both	All ages	Chronic respiratory diseases	Rate	2021	611

DALYs (Disability-Adjusted Life Years)	Belize	Both	All ages	Chronic respiratory diseases	Rate	2021	768
DALYs (Disability-Adjusted Life Years)	Federal Republic of Nigeria	Both	All ages	Chronic respiratory diseases	Rate	2021	518
DALYs (Disability-Adjusted Life Years)	Grand Duchy of Luxembourg	Both	All ages	Chronic respiratory diseases	Rate	2021	1066
DALYs (Disability-Adjusted Life Years)	Republic of the Gambia	Both	All ages	Chronic respiratory diseases	Rate	2021	677
DALYs (Disability-Adjusted Life Years)	Republic of Poland	Both	All ages	Chronic respiratory diseases	Rate	2021	1049
DALYs (Disability-Adjusted Life Years)	Republic of Niue	Both	All ages	Chronic respiratory diseases	Rate	2021	1791
DALYs (Disability-Adjusted Life Years)	Republic of Yemen	Both	All ages	Chronic respiratory diseases	Rate	2021	642
DALYs (Disability-Adjusted Life Years)	Bolivarian Republic of Venezuela	Both	All ages	Chronic respiratory diseases	Rate	2021	750
DALYs (Disability-Adjusted Life Years)	Central African Republic	Both	All ages	Chronic respiratory diseases	Rate	2021	1133
DALYs (Disability-Adjusted Life Years)	Islamic Republic of Pakistan	Both	All ages	Chronic respiratory diseases	Rate	2021	994
DALYs (Disability-Adjusted Life Years)	Togolese Republic	Both	All ages	Chronic respiratory diseases	Rate	2021	731
DALYs (Disability-Adjusted Life Years)	Republic of Angola	Both	All ages	Chronic respiratory diseases	Rate	2021	538

DALYs (Disability-Adjusted Life Years)	Independent State of Samoa	Both	All ages	Chronic respiratory diseases	Rate	2021	1343
DALYs (Disability-Adjusted Life Years)	Hashemite Kingdom of Jordan	Both	All ages	Chronic respiratory diseases	Rate	2021	388
DALYs (Disability-Adjusted Life Years)	Republic of Malta	Both	All ages	Chronic respiratory diseases	Rate	2021	1060
DALYs (Disability-Adjusted Life Years)	Ireland	Both	All ages	Chronic respiratory diseases	Rate	2021	1198
DALYs (Disability-Adjusted Life Years)	Republic of Kenya	Both	All ages	Chronic respiratory diseases	Rate	2021	635
DALYs (Disability-Adjusted Life Years)	Republic of Sierra Leone	Both	All ages	Chronic respiratory diseases	Rate	2021	682
DALYs (Disability-Adjusted Life Years)	Federative Republic of Brazil	Both	All ages	Chronic respiratory diseases	Rate	2021	899
DALYs (Disability-Adjusted Life Years)	Democratic Republic of the Congo	Both	All ages	Chronic respiratory diseases	Rate	2021	809
DALYs (Disability-Adjusted Life Years)	American Samoa	Both	All ages	Chronic respiratory diseases	Rate	2021	1226
DALYs (Disability-Adjusted Life Years)	Democratic Socialist Republic of Sri Lanka	Both	All ages	Chronic respiratory diseases	Rate	2021	1407
DALYs (Disability-Adjusted Life Years)	Slovak Republic	Both	All ages	Chronic respiratory diseases	Rate	2021	643
DALYs (Disability-Adjusted Life Years)	Eastern Republic of Uruguay	Both	All ages	Chronic respiratory diseases	Rate	2021	1473

DALYs (Disability-Adjusted Life Years)	Republic of Madagascar	Both	All ages	Chronic respiratory diseases	Rate	2021	1320
DALYs (Disability-Adjusted Life Years)	Republic of Guinea	Both	All ages	Chronic respiratory diseases	Rate	2021	741
DALYs (Disability-Adjusted Life Years)	Republic of Botswana	Both	All ages	Chronic respiratory diseases	Rate	2021	915
DALYs (Disability-Adjusted Life Years)	Turkmenistan	Both	All ages	Chronic respiratory diseases	Rate	2021	401
DALYs (Disability-Adjusted Life Years)	People's Republic of China	Both	All ages	Chronic respiratory diseases	Rate	2021	1815
DALYs (Disability-Adjusted Life Years)	Republic of Tajikistan	Both	All ages	Chronic respiratory diseases	Rate	2021	595
DALYs (Disability-Adjusted Life Years)	Republic of Cyprus	Both	All ages	Chronic respiratory diseases	Rate	2021	1135
DALYs (Disability-Adjusted Life Years)	Republic of CÃ´te d'Ivoire	Both	All ages	Chronic respiratory diseases	Rate	2021	607
DALYs (Disability-Adjusted Life Years)	Republic of Peru	Both	All ages	Chronic respiratory diseases	Rate	2021	656
DALYs (Disability-Adjusted Life Years)	Dominican Republic	Both	All ages	Chronic respiratory diseases	Rate	2021	581
DALYs (Disability-Adjusted Life Years)	Puerto Rico	Both	All ages	Chronic respiratory diseases	Rate	2021	1344
DALYs (Disability-Adjusted Life Years)	Republic of South Africa	Both	All ages	Chronic respiratory diseases	Rate	2021	1095

DALYs (Disability-Adjusted Life Years)	Kingdom of Thailand	Both	All ages	Chronic respiratory diseases	Rate	2021	1010
DALYs (Disability-Adjusted Life Years)	United Arab Emirates	Both	All ages	Chronic respiratory diseases	Rate	2021	709
DALYs (Disability-Adjusted Life Years)	Kingdom of Tonga	Both	All ages	Chronic respiratory diseases	Rate	2021	1143
DALYs (Disability-Adjusted Life Years)	Kingdom of Belgium	Both	All ages	Chronic respiratory diseases	Rate	2021	1319
DALYs (Disability-Adjusted Life Years)	Bermuda	Both	All ages	Chronic respiratory diseases	Rate	2021	830
DALYs (Disability-Adjusted Life Years)	Greenland	Both	All ages	Chronic respiratory diseases	Rate	2021	1526
DALYs (Disability-Adjusted Life Years)	Republic of Serbia	Both	All ages	Chronic respiratory diseases	Rate	2021	1111
DALYs (Disability-Adjusted Life Years)	Republic of Malawi	Both	All ages	Chronic respiratory diseases	Rate	2021	666
DALYs (Disability-Adjusted Life Years)	Saint Kitts and Nevis	Both	All ages	Chronic respiratory diseases	Rate	2021	702
DALYs (Disability-Adjusted Life Years)	Cook Islands	Both	All ages	Chronic respiratory diseases	Rate	2021	1010
DALYs (Disability-Adjusted Life Years)	Republic of Mauritius	Both	All ages	Chronic respiratory diseases	Rate	2021	1121
DALYs (Disability-Adjusted Life Years)	Republic of Namibia	Both	All ages	Chronic respiratory diseases	Rate	2021	1078

DALYs (Disability-Adjusted Life Years)	Republic of Vanuatu	Both	All ages	Chronic respiratory diseases	Rate	2021	1481
DALYs (Disability-Adjusted Life Years)	Republic of Uzbekistan	Both	All ages	Chronic respiratory diseases	Rate	2021	416
DALYs (Disability-Adjusted Life Years)	State of Libya	Both	All ages	Chronic respiratory diseases	Rate	2021	683
DALYs (Disability-Adjusted Life Years)	Tokelau	Both	All ages	Chronic respiratory diseases	Rate	2021	1569
DALYs (Disability-Adjusted Life Years)	Republic of San Marino	Both	All ages	Chronic respiratory diseases	Rate	2021	775
DALYs (Disability-Adjusted Life Years)	Republic of Ghana	Both	All ages	Chronic respiratory diseases	Rate	2021	460
DALYs (Disability-Adjusted Life Years)	Canada	Both	All ages	Chronic respiratory diseases	Rate	2021	1200
DALYs (Disability-Adjusted Life Years)	Republic of Sudan	Both	All ages	Chronic respiratory diseases	Rate	2021	582
DALYs (Disability-Adjusted Life Years)	Kingdom of the Netherlands	Both	All ages	Chronic respiratory diseases	Rate	2021	1541

S2D: Country based Diabetes maleates incidence, mortality and DALYs

measure	Country	sex	age	cause	metric	year	Rate
Incidence	Taiwan (Province of China)	Both	All ages	Diabetes and kidney diseases	Rate	2021	991
Incidence	Republic of the Union of Myanmar	Both	All ages	Diabetes and kidney diseases	Rate	2021	672
Incidence	Democratic Socialist Republic of Sri Lanka	Both	All ages	Diabetes and kidney diseases	Rate	2021	1074
Incidence	Republic of the Marshall Islands	Both	All ages	Diabetes and kidney diseases	Rate	2021	1087

Incidence	Kingdom of Cambodia	Both	All ages	Diabetes and kidney diseases	Rate	2021	419
Incidence	Turkmenistan	Both	All ages	Diabetes and kidney diseases	Rate	2021	364
Incidence	Lao People's Democratic Republic	Both	All ages	Diabetes and kidney diseases	Rate	2021	462
Incidence	Solomon Islands	Both	All ages	Diabetes and kidney diseases	Rate	2021	508
Incidence	Democratic Republic of Timor-Leste	Both	All ages	Diabetes and kidney diseases	Rate	2021	397
Incidence	Republic of Armenia	Both	All ages	Diabetes and kidney diseases	Rate	2021	576
Incidence	Independent State of Papua New Guinea	Both	All ages	Diabetes and kidney diseases	Rate	2021	545
Incidence	Georgia	Both	All ages	Diabetes and kidney diseases	Rate	2021	596
Incidence	Republic of Maldives	Both	All ages	Diabetes and kidney diseases	Rate	2021	493
Incidence	Democratic People's Republic of Korea	Both	All ages	Diabetes and kidney diseases	Rate	2021	547
Incidence	Republic of Vanuatu	Both	All ages	Diabetes and kidney diseases	Rate	2021	581
Incidence	Republic of Kiribati	Both	All ages	Diabetes and kidney diseases	Rate	2021	782
Incidence	Republic of Tajikistan	Both	All ages	Diabetes and kidney diseases	Rate	2021	282
Incidence	Kyrgyz Republic	Both	All ages	Diabetes and kidney diseases	Rate	2021	321
Incidence	Bosnia and Herzegovina	Both	All ages	Diabetes and kidney diseases	Rate	2021	992
Incidence	Republic of the Philippines	Both	All ages	Diabetes and kidney diseases	Rate	2021	462
Incidence	People's Republic of China	Both	All ages	Diabetes and kidney diseases	Rate	2021	523
Incidence	Republic of Indonesia	Both	All ages	Diabetes and kidney diseases	Rate	2021	469
Incidence	Federated States of Micronesia	Both	All ages	Diabetes and kidney diseases	Rate	2021	904
Incidence	Republic of Uzbekistan	Both	All ages	Diabetes and kidney diseases	Rate	2021	497
Incidence	Kingdom of Thailand	Both	All ages	Diabetes and kidney diseases	Rate	2021	954
Incidence	Republic of Azerbaijan	Both	All ages	Diabetes and kidney diseases	Rate	2021	508
Incidence	Malaysia	Both	All ages	Diabetes and kidney diseases	Rate	2021	670
Incidence	Independent State of Samoa	Both	All ages	Diabetes and kidney diseases	Rate	2021	796

Incidence	Czech Republic	Both	All ages	Diabetes and kidney diseases	Rate	2021	864
Incidence	Socialist Republic of Viet Nam	Both	All ages	Diabetes and kidney diseases	Rate	2021	534
Incidence	Republic of Kazakhstan	Both	All ages	Diabetes and kidney diseases	Rate	2021	510
Incidence	Republic of Bulgaria	Both	All ages	Diabetes and kidney diseases	Rate	2021	881
Incidence	Kingdom of Tonga	Both	All ages	Diabetes and kidney diseases	Rate	2021	768
Incidence	Mongolia	Both	All ages	Diabetes and kidney diseases	Rate	2021	347
Incidence	Republic of Albania	Both	All ages	Diabetes and kidney diseases	Rate	2021	583
Incidence	Ukraine	Both	All ages	Diabetes and kidney diseases	Rate	2021	447
Incidence	Republic of Estonia	Both	All ages	Diabetes and kidney diseases	Rate	2021	675
Incidence	Republic of Fiji	Both	All ages	Diabetes and kidney diseases	Rate	2021	1090
Incidence	North Macedonia	Both	All ages	Diabetes and kidney diseases	Rate	2021	964
Incidence	Republic of Finland	Both	All ages	Diabetes and kidney diseases	Rate	2021	857
Incidence	Republic of Lithuania	Both	All ages	Diabetes and kidney diseases	Rate	2021	510
Incidence	New Zealand	Both	All ages	Diabetes and kidney diseases	Rate	2021	786
Incidence	Republic of Poland	Both	All ages	Diabetes and kidney diseases	Rate	2021	777
Incidence	Republic of Serbia	Both	All ages	Diabetes and kidney diseases	Rate	2021	933
Incidence	Japan	Both	All ages	Diabetes and kidney diseases	Rate	2021	1192
Incidence	Federal Republic of Germany	Both	All ages	Diabetes and kidney diseases	Rate	2021	935
Incidence	Republic of Croatia	Both	All ages	Diabetes and kidney diseases	Rate	2021	943
Incidence	Republic of Singapore	Both	All ages	Diabetes and kidney diseases	Rate	2021	956
Incidence	Principality of Andorra	Both	All ages	Diabetes and kidney diseases	Rate	2021	715
Incidence	Hungary	Both	All ages	Diabetes and kidney diseases	Rate	2021	857
Incidence	Kingdom of Belgium	Both	All ages	Diabetes and kidney diseases	Rate	2021	806
Incidence	Republic of Malta	Both	All ages	Diabetes and kidney diseases	Rate	2021	1047

Incidence	Republic of Belarus	Both	All ages	Diabetes and kidney diseases	Rate	2021	417
Incidence	Kingdom of Denmark	Both	All ages	Diabetes and kidney diseases	Rate	2021	768
Incidence	Republic of Iceland	Both	All ages	Diabetes and kidney diseases	Rate	2021	594
Incidence	Republic of Slovenia	Both	All ages	Diabetes and kidney diseases	Rate	2021	786
Incidence	Russian Federation	Both	All ages	Diabetes and kidney diseases	Rate	2021	585
Incidence	Montenegro	Both	All ages	Diabetes and kidney diseases	Rate	2021	868
Incidence	State of Israel	Both	All ages	Diabetes and kidney diseases	Rate	2021	693
Incidence	Brunei Darussalam	Both	All ages	Diabetes and kidney diseases	Rate	2021	1028
Incidence	Republic of Moldova	Both	All ages	Diabetes and kidney diseases	Rate	2021	521
Incidence	French Republic	Both	All ages	Diabetes and kidney diseases	Rate	2021	714
Incidence	Romania	Both	All ages	Diabetes and kidney diseases	Rate	2021	657
Incidence	Republic of Austria	Both	All ages	Diabetes and kidney diseases	Rate	2021	778
Incidence	Hellenic Republic	Both	All ages	Diabetes and kidney diseases	Rate	2021	930
Incidence	Australia	Both	All ages	Diabetes and kidney diseases	Rate	2021	788
Incidence	Kingdom of Norway	Both	All ages	Diabetes and kidney diseases	Rate	2021	672
Incidence	United Kingdom of Great Britain and Northern Ireland	Both	All ages	Diabetes and kidney diseases	Rate	2021	815
Incidence	Republic of Korea	Both	All ages	Diabetes and kidney diseases	Rate	2021	1122
Incidence	Slovak Republic	Both	All ages	Diabetes and kidney diseases	Rate	2021	704
Incidence	Grand Duchy of Luxembourg	Both	All ages	Diabetes and kidney diseases	Rate	2021	711
Incidence	Republic of Latvia	Both	All ages	Diabetes and kidney diseases	Rate	2021	588
Incidence	Republic of Cyprus	Both	All ages	Diabetes and kidney diseases	Rate	2021	885
Incidence	Argentine Republic	Both	All ages	Diabetes and kidney diseases	Rate	2021	669
Incidence	Kingdom of the Netherlands	Both	All ages	Diabetes and kidney diseases	Rate	2021	784
Incidence	Dominican Republic	Both	All ages	Diabetes and kidney diseases	Rate	2021	664

Incidence	Ireland	Both	All ages	Diabetes and kidney diseases	Rate	2021	641
Incidence	Kingdom of Spain	Both	All ages	Diabetes and kidney diseases	Rate	2021	983
Incidence	Eastern Republic of Uruguay	Both	All ages	Diabetes and kidney diseases	Rate	2021	754
Incidence	Republic of Guyana	Both	All ages	Diabetes and kidney diseases	Rate	2021	1005
Incidence	Commonwealth of the Bahamas	Both	All ages	Diabetes and kidney diseases	Rate	2021	875
Incidence	Republic of El Salvador	Both	All ages	Diabetes and kidney diseases	Rate	2021	708
Incidence	Canada	Both	All ages	Diabetes and kidney diseases	Rate	2021	1005
Incidence	Jamaica	Both	All ages	Diabetes and kidney diseases	Rate	2021	803
Incidence	Belize	Both	All ages	Diabetes and kidney diseases	Rate	2021	647
Incidence	Republic of Ecuador	Both	All ages	Diabetes and kidney diseases	Rate	2021	701
Incidence	Republic of Italy	Both	All ages	Diabetes and kidney diseases	Rate	2021	900
Incidence	Republic of Honduras	Both	All ages	Diabetes and kidney diseases	Rate	2021	567
Incidence	Commonwealth of Dominica	Both	All ages	Diabetes and kidney diseases	Rate	2021	1048
Incidence	Saint Vincent and the Grenadines	Both	All ages	Diabetes and kidney diseases	Rate	2021	1080
Incidence	Portuguese Republic	Both	All ages	Diabetes and kidney diseases	Rate	2021	1053
Incidence	Swiss Confederation	Both	All ages	Diabetes and kidney diseases	Rate	2021	966
Incidence	Republic of Trinidad and Tobago	Both	All ages	Diabetes and kidney diseases	Rate	2021	1254
Incidence	Antigua and Barbuda	Both	All ages	Diabetes and kidney diseases	Rate	2021	1090
Incidence	Republic of Nicaragua	Both	All ages	Diabetes and kidney diseases	Rate	2021	744
Incidence	Republic of Chile	Both	All ages	Diabetes and kidney diseases	Rate	2021	883
Incidence	Grenada	Both	All ages	Diabetes and kidney diseases	Rate	2021	1083
Incidence	Plurinational State of Bolivia	Both	All ages	Diabetes and kidney diseases	Rate	2021	522
Incidence	Kingdom of Sweden	Both	All ages	Diabetes and kidney diseases	Rate	2021	678
Incidence	Bolivarian Republic of Venezuela	Both	All ages	Diabetes and kidney diseases	Rate	2021	815

Incidence	Republic of Haiti	Both	All ages	Diabetes and kidney diseases	Rate	2021	610
Incidence	Republic of Paraguay	Both	All ages	Diabetes and kidney diseases	Rate	2021	601
Incidence	Barbados	Both	All ages	Diabetes and kidney diseases	Rate	2021	1188
Incidence	Republic of Costa Rica	Both	All ages	Diabetes and kidney diseases	Rate	2021	925
Incidence	Republic of Suriname	Both	All ages	Diabetes and kidney diseases	Rate	2021	969
Incidence	United States of America	Both	All ages	Diabetes and kidney diseases	Rate	2021	1168
Incidence	Republic of Iraq	Both	All ages	Diabetes and kidney diseases	Rate	2021	923
Incidence	Republic of Cuba	Both	All ages	Diabetes and kidney diseases	Rate	2021	846
Incidence	Saint Lucia	Both	All ages	Diabetes and kidney diseases	Rate	2021	1173
Incidence	Republic of Guatemala	Both	All ages	Diabetes and kidney diseases	Rate	2021	692
Incidence	Republic of Peru	Both	All ages	Diabetes and kidney diseases	Rate	2021	464
Incidence	State of Kuwait	Both	All ages	Diabetes and kidney diseases	Rate	2021	1158
Incidence	Kingdom of Saudi Arabia	Both	All ages	Diabetes and kidney diseases	Rate	2021	920
Incidence	United Mexican States	Both	All ages	Diabetes and kidney diseases	Rate	2021	950
Incidence	State of Libya	Both	All ages	Diabetes and kidney diseases	Rate	2021	924
Incidence	Kingdom of Bahrain	Both	All ages	Diabetes and kidney diseases	Rate	2021	1216
Incidence	Republic of Colombia	Both	All ages	Diabetes and kidney diseases	Rate	2021	677
Incidence	Republic of Panama	Both	All ages	Diabetes and kidney diseases	Rate	2021	738
Incidence	People's Republic of Bangladesh	Both	All ages	Diabetes and kidney diseases	Rate	2021	424
Incidence	Federative Republic of Brazil	Both	All ages	Diabetes and kidney diseases	Rate	2021	646
Incidence	United Arab Emirates	Both	All ages	Diabetes and kidney diseases	Rate	2021	1023
Incidence	Republic of Angola	Both	All ages	Diabetes and kidney diseases	Rate	2021	259
Incidence	Republic of Tunisia	Both	All ages	Diabetes and kidney diseases	Rate	2021	1026
Incidence	Republic of Burundi	Both	All ages	Diabetes and kidney diseases	Rate	2021	157

Incidence	Islamic Republic of Iran	Both	All ages	Diabetes and kidney diseases	Rate	2021	792
Incidence	Palestine	Both	All ages	Diabetes and kidney diseases	Rate	2021	628
Incidence	Republic of India	Both	All ages	Diabetes and kidney diseases	Rate	2021	430
Incidence	Hashemite Kingdom of Jordan	Both	All ages	Diabetes and kidney diseases	Rate	2021	924
Incidence	Syrian Arab Republic	Both	All ages	Diabetes and kidney diseases	Rate	2021	891
Incidence	State of Qatar	Both	All ages	Diabetes and kidney diseases	Rate	2021	980
Incidence	Islamic Republic of Pakistan	Both	All ages	Diabetes and kidney diseases	Rate	2021	399
Incidence	Republic of Equatorial Guinea	Both	All ages	Diabetes and kidney diseases	Rate	2021	284
Incidence	Lebanese Republic	Both	All ages	Diabetes and kidney diseases	Rate	2021	1021
Incidence	People's Democratic Republic of Algeria	Both	All ages	Diabetes and kidney diseases	Rate	2021	834
Incidence	Republic of Djibouti	Both	All ages	Diabetes and kidney diseases	Rate	2021	216
Incidence	Republic of the Congo	Both	All ages	Diabetes and kidney diseases	Rate	2021	323
Incidence	Islamic Republic of Afghanistan	Both	All ages	Diabetes and kidney diseases	Rate	2021	597
Incidence	Arab Republic of Egypt	Both	All ages	Diabetes and kidney diseases	Rate	2021	658
Incidence	Kingdom of Bhutan	Both	All ages	Diabetes and kidney diseases	Rate	2021	408
Incidence	Federal Democratic Republic of Ethiopia	Both	All ages	Diabetes and kidney diseases	Rate	2021	178
Incidence	Republic of Mozambique	Both	All ages	Diabetes and kidney diseases	Rate	2021	174
Incidence	Kingdom of Morocco	Both	All ages	Diabetes and kidney diseases	Rate	2021	1051
Incidence	Federal Democratic Republic of Nepal	Both	All ages	Diabetes and kidney diseases	Rate	2021	469
Incidence	Republic of Seychelles	Both	All ages	Diabetes and kidney diseases	Rate	2021	1090
Incidence	Central African Republic	Both	All ages	Diabetes and kidney diseases	Rate	2021	315
Incidence	Union of the Comoros	Both	All ages	Diabetes and kidney diseases	Rate	2021	295
Incidence	Republic of Yemen	Both	All ages	Diabetes and kidney diseases	Rate	2021	348
Incidence	Kingdom of Lesotho	Both	All ages	Diabetes and kidney diseases	Rate	2021	368

Incidence	Sultanate of Oman	Both	All ages	Diabetes and kidney diseases	Rate	2021	613
Incidence	Republic of Turkey	Both	All ages	Diabetes and kidney diseases	Rate	2021	863
Incidence	United Republic of Tanzania	Both	All ages	Diabetes and kidney diseases	Rate	2021	173
Incidence	Republic of Madagascar	Both	All ages	Diabetes and kidney diseases	Rate	2021	157
Incidence	State of Eritrea	Both	All ages	Diabetes and kidney diseases	Rate	2021	220
Incidence	Burkina Faso	Both	All ages	Diabetes and kidney diseases	Rate	2021	231
Incidence	Republic of Guinea	Both	All ages	Diabetes and kidney diseases	Rate	2021	237
Incidence	Democratic Republic of the Congo	Both	All ages	Diabetes and kidney diseases	Rate	2021	232
Incidence	Republic of South Africa	Both	All ages	Diabetes and kidney diseases	Rate	2021	538
Incidence	Republic of Cabo Verde	Both	All ages	Diabetes and kidney diseases	Rate	2021	471
Incidence	Gabonese Republic	Both	All ages	Diabetes and kidney diseases	Rate	2021	412
Incidence	Republic of Liberia	Both	All ages	Diabetes and kidney diseases	Rate	2021	283
Incidence	Republic of Mauritius	Both	All ages	Diabetes and kidney diseases	Rate	2021	1501
Incidence	Republic of CÃ´te d'Ivoire	Both	All ages	Diabetes and kidney diseases	Rate	2021	286
Incidence	Republic of Zimbabwe	Both	All ages	Diabetes and kidney diseases	Rate	2021	262
Incidence	Republic of Sierra Leone	Both	All ages	Diabetes and kidney diseases	Rate	2021	260
Incidence	Republic of Zambia	Both	All ages	Diabetes and kidney diseases	Rate	2021	219
Incidence	Islamic Republic of Mauritania	Both	All ages	Diabetes and kidney diseases	Rate	2021	246
Incidence	Republic of Rwanda	Both	All ages	Diabetes and kidney diseases	Rate	2021	169
Incidence	Republic of Botswana	Both	All ages	Diabetes and kidney diseases	Rate	2021	396
Incidence	Federal Republic of Somalia	Both	All ages	Diabetes and kidney diseases	Rate	2021	153
Incidence	Republic of Kenya	Both	All ages	Diabetes and kidney diseases	Rate	2021	155
Incidence	Republic of Benin	Both	All ages	Diabetes and kidney diseases	Rate	2021	290
Incidence	Federal Republic of Nigeria	Both	All ages	Diabetes and kidney diseases	Rate	2021	217

Incidence	Republic of Namibia	Both	All ages	Diabetes and kidney diseases	Rate	2021	311
Incidence	Republic of Ghana	Both	All ages	Diabetes and kidney diseases	Rate	2021	299
Incidence	Kingdom of Eswatini	Both	All ages	Diabetes and kidney diseases	Rate	2021	410
Incidence	Republic of Cameroon	Both	All ages	Diabetes and kidney diseases	Rate	2021	293
Incidence	Republic of Malawi	Both	All ages	Diabetes and kidney diseases	Rate	2021	144
Incidence	Guam	Both	All ages	Diabetes and kidney diseases	Rate	2021	758
Incidence	Republic of Senegal	Both	All ages	Diabetes and kidney diseases	Rate	2021	351
Incidence	Bermuda	Both	All ages	Diabetes and kidney diseases	Rate	2021	1085
Incidence	Republic of Palau	Both	All ages	Diabetes and kidney diseases	Rate	2021	1417
Incidence	Republic of Guinea-Bissau	Both	All ages	Diabetes and kidney diseases	Rate	2021	273
Incidence	Republic of Chad	Both	All ages	Diabetes and kidney diseases	Rate	2021	209
Incidence	Republic of Uganda	Both	All ages	Diabetes and kidney diseases	Rate	2021	145
Incidence	Saint Kitts and Nevis	Both	All ages	Diabetes and kidney diseases	Rate	2021	1114
Incidence	Republic of Mali	Both	All ages	Diabetes and kidney diseases	Rate	2021	328
Incidence	Republic of the Niger	Both	All ages	Diabetes and kidney diseases	Rate	2021	205
Incidence	Togolese Republic	Both	All ages	Diabetes and kidney diseases	Rate	2021	234
Incidence	Republic of the Gambia	Both	All ages	Diabetes and kidney diseases	Rate	2021	261
Incidence	Republic of Nauru	Both	All ages	Diabetes and kidney diseases	Rate	2021	795
Incidence	Greenland	Both	All ages	Diabetes and kidney diseases	Rate	2021	544
Incidence	Tokelau	Both	All ages	Diabetes and kidney diseases	Rate	2021	1020
Incidence	Northern Mariana Islands	Both	All ages	Diabetes and kidney diseases	Rate	2021	919
Incidence	United States Virgin Islands	Both	All ages	Diabetes and kidney diseases	Rate	2021	1471
Incidence	Democratic Republic of Sao Tome and Principe	Both	All ages	Diabetes and kidney diseases	Rate	2021	361
Incidence	American Samoa	Both	All ages	Diabetes and kidney diseases	Rate	2021	1238

Incidence	Puerto Rico	Both	All ages	Diabetes and kidney diseases	Rate	2021	1360
Incidence	Principality of Monaco	Both	All ages	Diabetes and kidney diseases	Rate	2021	867
Incidence	Republic of San Marino	Both	All ages	Diabetes and kidney diseases	Rate	2021	778
Incidence	Republic of Niue	Both	All ages	Diabetes and kidney diseases	Rate	2021	1218
Incidence	Cook Islands	Both	All ages	Diabetes and kidney diseases	Rate	2021	1311
Incidence	Tuvalu	Both	All ages	Diabetes and kidney diseases	Rate	2021	737
Incidence	Republic of South Sudan	Both	All ages	Diabetes and kidney diseases	Rate	2021	167
Incidence	Republic of Sudan	Both	All ages	Diabetes and kidney diseases	Rate	2021	450
measure	Country	sex	age	cause	metric	year	Rate
Deaths	Republic of Angola	Both	All ages	Diabetes and kidney diseases	Rate	2021	27
Deaths	Kyrgyz Republic	Both	All ages	Diabetes and kidney diseases	Rate	2021	13
Deaths	Federative Republic of Brazil	Both	All ages	Diabetes and kidney diseases	Rate	2021	50
Deaths	Republic of Paraguay	Both	All ages	Diabetes and kidney diseases	Rate	2021	64
Deaths	Republic of Cameroon	Both	All ages	Diabetes and kidney diseases	Rate	2021	36
Deaths	Republic of Kiribati	Both	All ages	Diabetes and kidney diseases	Rate	2021	122
Deaths	Plurinational State of Bolivia	Both	All ages	Diabetes and kidney diseases	Rate	2021	71
Deaths	Republic of Tunisia	Both	All ages	Diabetes and kidney diseases	Rate	2021	49
Deaths	Republic of Malta	Both	All ages	Diabetes and kidney diseases	Rate	2021	66
Deaths	Federal Republic of Somalia	Both	All ages	Diabetes and kidney diseases	Rate	2021	27
Deaths	Barbados	Both	All ages	Diabetes and kidney diseases	Rate	2021	155
Deaths	Kingdom of Bahrain	Both	All ages	Diabetes and kidney diseases	Rate	2021	61
Deaths	Kingdom of the Netherlands	Both	All ages	Diabetes and kidney diseases	Rate	2021	44
Deaths	Republic of Fiji	Both	All ages	Diabetes and kidney diseases	Rate	2021	231
Deaths	Independent State of Samoa	Both	All ages	Diabetes and kidney diseases	Rate	2021	86
Deaths	Principality of Andorra	Both	All ages	Diabetes and kidney diseases	Rate	2021	37

Deaths	Australia	Both	All ages	Diabetes and kidney diseases	Rate	2021	36
Deaths	Republic of Slovenia	Both	All ages	Diabetes and kidney diseases	Rate	2021	36
Deaths	Republic of Tajikistan	Both	All ages	Diabetes and kidney diseases	Rate	2021	10
Deaths	Gabonese Republic	Both	All ages	Diabetes and kidney diseases	Rate	2021	72
Deaths	People's Republic of China	Both	All ages	Diabetes and kidney diseases	Rate	2021	27
Deaths	Republic of Rwanda	Both	All ages	Diabetes and kidney diseases	Rate	2021	32
Deaths	Republic of Palau	Both	All ages	Diabetes and kidney diseases	Rate	2021	164
Deaths	Republic of Costa Rica	Both	All ages	Diabetes and kidney diseases	Rate	2021	56
Deaths	Solomon Islands	Both	All ages	Diabetes and kidney diseases	Rate	2021	67
Deaths	Central African Republic	Both	All ages	Diabetes and kidney diseases	Rate	2021	40
Deaths	Republic of Ghana	Both	All ages	Diabetes and kidney diseases	Rate	2021	41
Deaths	Republic of Austria	Both	All ages	Diabetes and kidney diseases	Rate	2021	60
Deaths	Kingdom of Norway	Both	All ages	Diabetes and kidney diseases	Rate	2021	27
Deaths	New Zealand	Both	All ages	Diabetes and kidney diseases	Rate	2021	31
Deaths	Republic of Cabo Verde	Both	All ages	Diabetes and kidney diseases	Rate	2021	49
Deaths	Syrian Arab Republic	Both	All ages	Diabetes and kidney diseases	Rate	2021	50
Deaths	Turkmenistan	Both	All ages	Diabetes and kidney diseases	Rate	2021	32
Deaths	Republic of Seychelles	Both	All ages	Diabetes and kidney diseases	Rate	2021	72
Deaths	Malaysia	Both	All ages	Diabetes and kidney diseases	Rate	2021	40
Deaths	Republic of Botswana	Both	All ages	Diabetes and kidney diseases	Rate	2021	45
Deaths	Republic of Croatia	Both	All ages	Diabetes and kidney diseases	Rate	2021	69
Deaths	Republic of Lithuania	Both	All ages	Diabetes and kidney diseases	Rate	2021	33
Deaths	Kingdom of Sweden	Both	All ages	Diabetes and kidney diseases	Rate	2021	43
Deaths	Republic of Maldives	Both	All ages	Diabetes and kidney diseases	Rate	2021	27

Deaths	Republic of the Congo	Both	All ages	Diabetes and kidney diseases	Rate	2021	51
Deaths	Taiwan (Province of China)	Both	All ages	Diabetes and kidney diseases	Rate	2021	82
Deaths	Antigua and Barbuda	Both	All ages	Diabetes and kidney diseases	Rate	2021	116
Deaths	Republic of Belarus	Both	All ages	Diabetes and kidney diseases	Rate	2021	12
Deaths	Commonwealth of the Bahamas	Both	All ages	Diabetes and kidney diseases	Rate	2021	74
Deaths	Democratic People's Republic of Korea	Both	All ages	Diabetes and kidney diseases	Rate	2021	34
Deaths	Grenada	Both	All ages	Diabetes and kidney diseases	Rate	2021	143
Deaths	Republic of Turkey	Both	All ages	Diabetes and kidney diseases	Rate	2021	57
Deaths	United Republic of Tanzania	Both	All ages	Diabetes and kidney diseases	Rate	2021	30
Deaths	Republic of Equatorial Guinea	Both	All ages	Diabetes and kidney diseases	Rate	2021	36
Deaths	Principality of Monaco	Both	All ages	Diabetes and kidney diseases	Rate	2021	43
Deaths	State of Eritrea	Both	All ages	Diabetes and kidney diseases	Rate	2021	33
Deaths	State of Kuwait	Both	All ages	Diabetes and kidney diseases	Rate	2021	20
Deaths	Independent State of Papua New Guinea	Both	All ages	Diabetes and kidney diseases	Rate	2021	46
Deaths	Federal Democratic Republic of Nepal	Both	All ages	Diabetes and kidney diseases	Rate	2021	34
Deaths	Federated States of Micronesia	Both	All ages	Diabetes and kidney diseases	Rate	2021	114
Deaths	Republic of Chad	Both	All ages	Diabetes and kidney diseases	Rate	2021	21
Deaths	Republic of Albania	Both	All ages	Diabetes and kidney diseases	Rate	2021	24
Deaths	Democratic Republic of the Congo	Both	All ages	Diabetes and kidney diseases	Rate	2021	33
Deaths	Republic of Nauru	Both	All ages	Diabetes and kidney diseases	Rate	2021	103
Deaths	Republic of Finland	Both	All ages	Diabetes and kidney diseases	Rate	2021	26
Deaths	Republic of the Marshall Islands	Both	All ages	Diabetes and kidney diseases	Rate	2021	124
Deaths	Republic of Nicaragua	Both	All ages	Diabetes and kidney diseases	Rate	2021	62
Deaths	Republic of Ecuador	Both	All ages	Diabetes and kidney diseases	Rate	2021	61

Deaths	Republic of Uzbekistan	Both	All ages	Diabetes and kidney diseases	Rate	2021	30
Deaths	Republic of Estonia	Both	All ages	Diabetes and kidney diseases	Rate	2021	64
Deaths	People's Democratic Republic of Algeria	Both	All ages	Diabetes and kidney diseases	Rate	2021	35
Deaths	Republic of Niue	Both	All ages	Diabetes and kidney diseases	Rate	2021	222
Deaths	Republic of Uganda	Both	All ages	Diabetes and kidney diseases	Rate	2021	26
Deaths	United Arab Emirates	Both	All ages	Diabetes and kidney diseases	Rate	2021	16
Deaths	Tokelau	Both	All ages	Diabetes and kidney diseases	Rate	2021	132
Deaths	Belize	Both	All ages	Diabetes and kidney diseases	Rate	2021	68
Deaths	Kingdom of Cambodia	Both	All ages	Diabetes and kidney diseases	Rate	2021	38
Deaths	Republic of Mali	Both	All ages	Diabetes and kidney diseases	Rate	2021	26
Deaths	Republic of Peru	Both	All ages	Diabetes and kidney diseases	Rate	2021	45
Deaths	Kingdom of Eswatini	Both	All ages	Diabetes and kidney diseases	Rate	2021	75
Deaths	Republic of Colombia	Both	All ages	Diabetes and kidney diseases	Rate	2021	33
Deaths	Kingdom of Thailand	Both	All ages	Diabetes and kidney diseases	Rate	2021	84
Deaths	Bermuda	Both	All ages	Diabetes and kidney diseases	Rate	2021	76
Deaths	Republic of CÃ´te d'Ivoire	Both	All ages	Diabetes and kidney diseases	Rate	2021	29
Deaths	Kingdom of Spain	Both	All ages	Diabetes and kidney diseases	Rate	2021	53
Deaths	Republic of the Gambia	Both	All ages	Diabetes and kidney diseases	Rate	2021	33
Deaths	Republic of Latvia	Both	All ages	Diabetes and kidney diseases	Rate	2021	47
Deaths	Republic of Zimbabwe	Both	All ages	Diabetes and kidney diseases	Rate	2021	41
Deaths	Northern Mariana Islands	Both	All ages	Diabetes and kidney diseases	Rate	2021	110
Deaths	Portuguese Republic	Both	All ages	Diabetes and kidney diseases	Rate	2021	82
Deaths	Kingdom of Belgium	Both	All ages	Diabetes and kidney diseases	Rate	2021	37
Deaths	Palestine	Both	All ages	Diabetes and kidney diseases	Rate	2021	37

Deaths	Republic of Poland	Both	All ages	Diabetes and kidney diseases	Rate	2021	40
Deaths	Republic of Indonesia	Both	All ages	Diabetes and kidney diseases	Rate	2021	41
Deaths	Bosnia and Herzegovina	Both	All ages	Diabetes and kidney diseases	Rate	2021	82
Deaths	Republic of Yemen	Both	All ages	Diabetes and kidney diseases	Rate	2021	13
Deaths	Republic of Zambia	Both	All ages	Diabetes and kidney diseases	Rate	2021	32
Deaths	Republic of Cuba	Both	All ages	Diabetes and kidney diseases	Rate	2021	47
Deaths	Arab Republic of Egypt	Both	All ages	Diabetes and kidney diseases	Rate	2021	58
Deaths	Kingdom of Denmark	Both	All ages	Diabetes and kidney diseases	Rate	2021	53
Deaths	Swiss Confederation	Both	All ages	Diabetes and kidney diseases	Rate	2021	43
Deaths	Islamic Republic of Mauritania	Both	All ages	Diabetes and kidney diseases	Rate	2021	34
Deaths	Sultanate of Oman	Both	All ages	Diabetes and kidney diseases	Rate	2021	28
Deaths	Islamic Republic of Iran	Both	All ages	Diabetes and kidney diseases	Rate	2021	33
Deaths	Republic of Moldova	Both	All ages	Diabetes and kidney diseases	Rate	2021	21
Deaths	Republic of Malawi	Both	All ages	Diabetes and kidney diseases	Rate	2021	32
Deaths	Republic of Guinea	Both	All ages	Diabetes and kidney diseases	Rate	2021	30
Deaths	Ireland	Both	All ages	Diabetes and kidney diseases	Rate	2021	23
Deaths	Republic of Cyprus	Both	All ages	Diabetes and kidney diseases	Rate	2021	64
Deaths	Saint Vincent and the Grenadines	Both	All ages	Diabetes and kidney diseases	Rate	2021	132
Deaths	Dominican Republic	Both	All ages	Diabetes and kidney diseases	Rate	2021	48
Deaths	Republic of Burundi	Both	All ages	Diabetes and kidney diseases	Rate	2021	26
Deaths	Republic of Iraq	Both	All ages	Diabetes and kidney diseases	Rate	2021	44
Deaths	Republic of Bulgaria	Both	All ages	Diabetes and kidney diseases	Rate	2021	72
Deaths	Union of the Comoros	Both	All ages	Diabetes and kidney diseases	Rate	2021	50
Deaths	Lao People's Democratic Republic	Both	All ages	Diabetes and kidney diseases	Rate	2021	45

Deaths	Republic of Chile	Both	All ages	Diabetes and kidney diseases	Rate	2021	43
Deaths	Russian Federation	Both	All ages	Diabetes and kidney diseases	Rate	2021	37
Deaths	Czech Republic	Both	All ages	Diabetes and kidney diseases	Rate	2021	56
Deaths	Kingdom of Tonga	Both	All ages	Diabetes and kidney diseases	Rate	2021	97
Deaths	Republic of Djibouti	Both	All ages	Diabetes and kidney diseases	Rate	2021	36
Deaths	Kingdom of Lesotho	Both	All ages	Diabetes and kidney diseases	Rate	2021	75
Deaths	Saint Kitts and Nevis	Both	All ages	Diabetes and kidney diseases	Rate	2021	111
Deaths	Islamic Republic of Afghanistan	Both	All ages	Diabetes and kidney diseases	Rate	2021	27
Deaths	Hashemite Kingdom of Jordan	Both	All ages	Diabetes and kidney diseases	Rate	2021	33
Deaths	Republic of El Salvador	Both	All ages	Diabetes and kidney diseases	Rate	2021	110
Deaths	Republic of Guinea-Bissau	Both	All ages	Diabetes and kidney diseases	Rate	2021	32
Deaths	Republic of Liberia	Both	All ages	Diabetes and kidney diseases	Rate	2021	32
Deaths	Hungary	Both	All ages	Diabetes and kidney diseases	Rate	2021	52
Deaths	Commonwealth of Dominica	Both	All ages	Diabetes and kidney diseases	Rate	2021	147
Deaths	Republic of Sudan	Both	All ages	Diabetes and kidney diseases	Rate	2021	21
Deaths	Puerto Rico	Both	All ages	Diabetes and kidney diseases	Rate	2021	176
Deaths	Republic of Vanuatu	Both	All ages	Diabetes and kidney diseases	Rate	2021	63
Deaths	Republic of South Sudan	Both	All ages	Diabetes and kidney diseases	Rate	2021	40
Deaths	Republic of Senegal	Both	All ages	Diabetes and kidney diseases	Rate	2021	39
Deaths	American Samoa	Both	All ages	Diabetes and kidney diseases	Rate	2021	164
Deaths	French Republic	Both	All ages	Diabetes and kidney diseases	Rate	2021	42
Deaths	Argentine Republic	Both	All ages	Diabetes and kidney diseases	Rate	2021	54
Deaths	United Kingdom of Great Britain and Northern Ireland	Both	All ages	Diabetes and kidney diseases	Rate	2021	24
Deaths	Republic of the Union of Myanmar	Both	All ages	Diabetes and kidney diseases	Rate	2021	65

Deaths	Ukraine	Both	All ages	Diabetes and kidney diseases	Rate	2021	9
Deaths	Republic of Guatemala	Both	All ages	Diabetes and kidney diseases	Rate	2021	78
Deaths	Republic of San Marino	Both	All ages	Diabetes and kidney diseases	Rate	2021	25
Deaths	United Mexican States	Both	All ages	Diabetes and kidney diseases	Rate	2021	118
Deaths	Republic of Guyana	Both	All ages	Diabetes and kidney diseases	Rate	2021	113
Deaths	Kingdom of Bhutan	Both	All ages	Diabetes and kidney diseases	Rate	2021	40
Deaths	Republic of South Africa	Both	All ages	Diabetes and kidney diseases	Rate	2021	81
Deaths	People's Republic of Bangladesh	Both	All ages	Diabetes and kidney diseases	Rate	2021	34
Deaths	Lebanese Republic	Both	All ages	Diabetes and kidney diseases	Rate	2021	72
Deaths	Republic of Namibia	Both	All ages	Diabetes and kidney diseases	Rate	2021	48
Deaths	Republic of Honduras	Both	All ages	Diabetes and kidney diseases	Rate	2021	32
Deaths	Jamaica	Both	All ages	Diabetes and kidney diseases	Rate	2021	111
Deaths	Republic of Iceland	Both	All ages	Diabetes and kidney diseases	Rate	2021	20
Deaths	Federal Democratic Republic of Ethiopia	Both	All ages	Diabetes and kidney diseases	Rate	2021	29
Deaths	Federal Republic of Germany	Both	All ages	Diabetes and kidney diseases	Rate	2021	72
Deaths	Republic of Madagascar	Both	All ages	Diabetes and kidney diseases	Rate	2021	21
Deaths	Republic of India	Both	All ages	Diabetes and kidney diseases	Rate	2021	36
Deaths	Republic of Kazakhstan	Both	All ages	Diabetes and kidney diseases	Rate	2021	19
Deaths	Brunei Darussalam	Both	All ages	Diabetes and kidney diseases	Rate	2021	52
Deaths	Hellenic Republic	Both	All ages	Diabetes and kidney diseases	Rate	2021	87
Deaths	Republic of the Niger	Both	All ages	Diabetes and kidney diseases	Rate	2021	16
Deaths	Kingdom of Morocco	Both	All ages	Diabetes and kidney diseases	Rate	2021	52
Deaths	Republic of Armenia	Both	All ages	Diabetes and kidney diseases	Rate	2021	36
Deaths	Saint Lucia	Both	All ages	Diabetes and kidney diseases	Rate	2021	129

Deaths	Democratic Socialist Republic of Sri Lanka	Both	All ages	Diabetes and kidney diseases	Rate	2021	78
Deaths	Democratic Republic of Timor-Leste	Both	All ages	Diabetes and kidney diseases	Rate	2021	30
Deaths	Republic of Haiti	Both	All ages	Diabetes and kidney diseases	Rate	2021	58
Deaths	Romania	Both	All ages	Diabetes and kidney diseases	Rate	2021	32
Deaths	Republic of the Philippines	Both	All ages	Diabetes and kidney diseases	Rate	2021	56
Deaths	Tuvalu	Both	All ages	Diabetes and kidney diseases	Rate	2021	110
Deaths	Montenegro	Both	All ages	Diabetes and kidney diseases	Rate	2021	56
Deaths	Republic of Azerbaijan	Both	All ages	Diabetes and kidney diseases	Rate	2021	27
Deaths	Eastern Republic of Uruguay	Both	All ages	Diabetes and kidney diseases	Rate	2021	63
Deaths	State of Libya	Both	All ages	Diabetes and kidney diseases	Rate	2021	48
Deaths	Federal Republic of Nigeria	Both	All ages	Diabetes and kidney diseases	Rate	2021	24
Deaths	Democratic Republic of Sao Tome and Principe	Both	All ages	Diabetes and kidney diseases	Rate	2021	40
Deaths	Georgia	Both	All ages	Diabetes and kidney diseases	Rate	2021	49
Deaths	Republic of Panama	Both	All ages	Diabetes and kidney diseases	Rate	2021	62
Deaths	Canada	Both	All ages	Diabetes and kidney diseases	Rate	2021	38
Deaths	North Macedonia	Both	All ages	Diabetes and kidney diseases	Rate	2021	62
Deaths	State of Israel	Both	All ages	Diabetes and kidney diseases	Rate	2021	55
Deaths	Islamic Republic of Pakistan	Both	All ages	Diabetes and kidney diseases	Rate	2021	33
Deaths	Burkina Faso	Both	All ages	Diabetes and kidney diseases	Rate	2021	29
Deaths	Japan	Both	All ages	Diabetes and kidney diseases	Rate	2021	49
Deaths	Republic of Suriname	Both	All ages	Diabetes and kidney diseases	Rate	2021	89
Deaths	United States of America	Both	All ages	Diabetes and kidney diseases	Rate	2021	63
Deaths	Republic of Kenya	Both	All ages	Diabetes and kidney diseases	Rate	2021	29
Deaths	Republic of Singapore	Both	All ages	Diabetes and kidney diseases	Rate	2021	17

Deaths	Cook Islands	Both	All ages	Diabetes and kidney diseases	Rate	2021	194
Deaths	Republic of Mauritius	Both	All ages	Diabetes and kidney diseases	Rate	2021	263
Deaths	United States Virgin Islands	Both	All ages	Diabetes and kidney diseases	Rate	2021	99
Deaths	Bolivarian Republic of Venezuela	Both	All ages	Diabetes and kidney diseases	Rate	2021	92
Deaths	Republic of Korea	Both	All ages	Diabetes and kidney diseases	Rate	2021	40
Deaths	Guam	Both	All ages	Diabetes and kidney diseases	Rate	2021	57
Deaths	Republic of Benin	Both	All ages	Diabetes and kidney diseases	Rate	2021	27
Deaths	State of Qatar	Both	All ages	Diabetes and kidney diseases	Rate	2021	19
Deaths	Republic of Italy	Both	All ages	Diabetes and kidney diseases	Rate	2021	64
Deaths	Republic of Mozambique	Both	All ages	Diabetes and kidney diseases	Rate	2021	28
Deaths	Grand Duchy of Luxembourg	Both	All ages	Diabetes and kidney diseases	Rate	2021	36
Deaths	Republic of Sierra Leone	Both	All ages	Diabetes and kidney diseases	Rate	2021	24
Deaths	Greenland	Both	All ages	Diabetes and kidney diseases	Rate	2021	24
Deaths	Mongolia	Both	All ages	Diabetes and kidney diseases	Rate	2021	15
Deaths	Togolese Republic	Both	All ages	Diabetes and kidney diseases	Rate	2021	27
Deaths	Kingdom of Saudi Arabia	Both	All ages	Diabetes and kidney diseases	Rate	2021	46
Deaths	Slovak Republic	Both	All ages	Diabetes and kidney diseases	Rate	2021	31
Deaths	Republic of Serbia	Both	All ages	Diabetes and kidney diseases	Rate	2021	74
Deaths	Socialist Republic of Viet Nam	Both	All ages	Diabetes and kidney diseases	Rate	2021	55
Deaths	Republic of Trinidad and Tobago	Both	All ages	Diabetes and kidney diseases	Rate	2021	188
measure	Country	sex	age	cause	metric	year	Rate
DALYs (Disability-Adjusted Life Years)	Republic of Cabo Verde	Both	All ages	Diabetes and kidney diseases	Rate	2021	1664
DALYs (Disability-Adjusted Life Years)	Northern Mariana Islands	Both	All ages	Diabetes and kidney diseases	Rate	2021	4098

DALYs (Disability-Adjusted Life Years)	Democratic Republic of Sao Tome and Principe	Both	All ages	Diabetes and kidney diseases	Rate	2021	1509
DALYs (Disability-Adjusted Life Years)	Federated States of Micronesia	Both	All ages	Diabetes and kidney diseases	Rate	2021	4465
DALYs (Disability-Adjusted Life Years)	Malaysia	Both	All ages	Diabetes and kidney diseases	Rate	2021	1741
DALYs (Disability-Adjusted Life Years)	State of Israel	Both	All ages	Diabetes and kidney diseases	Rate	2021	1411
DALYs (Disability-Adjusted Life Years)	Kyrgyz Republic	Both	All ages	Diabetes and kidney diseases	Rate	2021	819
DALYs (Disability-Adjusted Life Years)	Republic of Moldova	Both	All ages	Diabetes and kidney diseases	Rate	2021	1391
DALYs (Disability-Adjusted Life Years)	Argentine Republic	Both	All ages	Diabetes and kidney diseases	Rate	2021	1634
DALYs (Disability-Adjusted Life Years)	North Macedonia	Both	All ages	Diabetes and kidney diseases	Rate	2021	2394
DALYs (Disability-Adjusted Life Years)	Republic of Mozambique	Both	All ages	Diabetes and kidney diseases	Rate	2021	1116
DALYs (Disability-Adjusted Life Years)	Ukraine	Both	All ages	Diabetes and kidney diseases	Rate	2021	925
DALYs (Disability-Adjusted Life Years)	Republic of Tajikistan	Both	All ages	Diabetes and kidney diseases	Rate	2021	659
DALYs (Disability-Adjusted Life Years)	Federative Republic of Brazil	Both	All ages	Diabetes and kidney diseases	Rate	2021	1817
DALYs (Disability-	Republic of Poland	Both	All ages	Diabetes and kidney diseases	Rate	2021	1715

Adjusted Life Years)							
DALYs (Disability-Adjusted Life Years)	Kingdom of the Netherlands	Both	All ages	Diabetes and kidney diseases	Rate	2021	1282
DALYs (Disability-Adjusted Life Years)	Eastern Republic of Uruguay	Both	All ages	Diabetes and kidney diseases	Rate	2021	1771
DALYs (Disability-Adjusted Life Years)	Dominican Republic	Both	All ages	Diabetes and kidney diseases	Rate	2021	2128
DALYs (Disability-Adjusted Life Years)	Republic of Austria	Both	All ages	Diabetes and kidney diseases	Rate	2021	1373
DALYs (Disability-Adjusted Life Years)	Grand Duchy of Luxembourg	Both	All ages	Diabetes and kidney diseases	Rate	2021	1138
DALYs (Disability-Adjusted Life Years)	Republic of Guinea	Both	All ages	Diabetes and kidney diseases	Rate	2021	1109
DALYs (Disability-Adjusted Life Years)	State of Libya	Both	All ages	Diabetes and kidney diseases	Rate	2021	2046
DALYs (Disability-Adjusted Life Years)	Kingdom of Eswatini	Both	All ages	Diabetes and kidney diseases	Rate	2021	2517
DALYs (Disability-Adjusted Life Years)	People's Republic of China	Both	All ages	Diabetes and kidney diseases	Rate	2021	1265
DALYs (Disability-Adjusted Life Years)	Republic of Albania	Both	All ages	Diabetes and kidney diseases	Rate	2021	1080
DALYs (Disability-Adjusted Life Years)	Slovak Republic	Both	All ages	Diabetes and kidney diseases	Rate	2021	1373
DALYs (Disability-Adjusted Life Years)	Republic of Guyana	Both	All ages	Diabetes and kidney diseases	Rate	2021	4561

DALYs (Disability-Adjusted Life Years)	Palestine	Both	All ages	Diabetes and kidney diseases	Rate	2021	1384
DALYs (Disability-Adjusted Life Years)	Democratic Republic of the Congo	Both	All ages	Diabetes and kidney diseases	Rate	2021	1298
DALYs (Disability-Adjusted Life Years)	Democratic Republic of Timor-Leste	Both	All ages	Diabetes and kidney diseases	Rate	2021	1255
DALYs (Disability-Adjusted Life Years)	Republic of Colombia	Both	All ages	Diabetes and kidney diseases	Rate	2021	1428
DALYs (Disability-Adjusted Life Years)	Democratic People's Republic of Korea	Both	All ages	Diabetes and kidney diseases	Rate	2021	1549
DALYs (Disability-Adjusted Life Years)	Kingdom of Denmark	Both	All ages	Diabetes and kidney diseases	Rate	2021	1342
DALYs (Disability-Adjusted Life Years)	Guam	Both	All ages	Diabetes and kidney diseases	Rate	2021	2591
DALYs (Disability-Adjusted Life Years)	Republic of Mauritius	Both	All ages	Diabetes and kidney diseases	Rate	2021	8125
DALYs (Disability-Adjusted Life Years)	Republic of Guinea-Bissau	Both	All ages	Diabetes and kidney diseases	Rate	2021	1360
DALYs (Disability-Adjusted Life Years)	Brunei Darussalam	Both	All ages	Diabetes and kidney diseases	Rate	2021	2453
DALYs (Disability-Adjusted Life Years)	Canada	Both	All ages	Diabetes and kidney diseases	Rate	2021	1568
DALYs (Disability-Adjusted Life Years)	Republic of South Africa	Both	All ages	Diabetes and kidney diseases	Rate	2021	2551
DALYs (Disability-	Republic of Haiti	Both	All ages	Diabetes and kidney diseases	Rate	2021	2492

Adjusted Life Years)							
DALYs (Disability-Adjusted Life Years)	Republic of Liberia	Both	All ages	Diabetes and kidney diseases	Rate	2021	1327
DALYs (Disability-Adjusted Life Years)	Republic of Peru	Both	All ages	Diabetes and kidney diseases	Rate	2021	1353
DALYs (Disability-Adjusted Life Years)	Tuvalu	Both	All ages	Diabetes and kidney diseases	Rate	2021	3887
DALYs (Disability-Adjusted Life Years)	Greenland	Both	All ages	Diabetes and kidney diseases	Rate	2021	1005
DALYs (Disability-Adjusted Life Years)	Taiwan (Province of China)	Both	All ages	Diabetes and kidney diseases	Rate	2021	2602
DALYs (Disability-Adjusted Life Years)	Republic of Rwanda	Both	All ages	Diabetes and kidney diseases	Rate	2021	1081
DALYs (Disability-Adjusted Life Years)	Republic of Slovenia	Both	All ages	Diabetes and kidney diseases	Rate	2021	1427
DALYs (Disability-Adjusted Life Years)	United States of America	Both	All ages	Diabetes and kidney diseases	Rate	2021	2421
DALYs (Disability-Adjusted Life Years)	Kingdom of Norway	Both	All ages	Diabetes and kidney diseases	Rate	2021	999
DALYs (Disability-Adjusted Life Years)	Kingdom of Thailand	Both	All ages	Diabetes and kidney diseases	Rate	2021	2912
DALYs (Disability-Adjusted Life Years)	People's Republic of Bangladesh	Both	All ages	Diabetes and kidney diseases	Rate	2021	1339
DALYs (Disability-Adjusted Life Years)	Republic of Zimbabwe	Both	All ages	Diabetes and kidney diseases	Rate	2021	1465

DALYs (Disability-Adjusted Life Years)	United States Virgin Islands	Both	All ages	Diabetes and kidney diseases	Rate	2021	4585
DALYs (Disability-Adjusted Life Years)	Japan	Both	All ages	Diabetes and kidney diseases	Rate	2021	1815
DALYs (Disability-Adjusted Life Years)	Kingdom of Bahrain	Both	All ages	Diabetes and kidney diseases	Rate	2021	2586
DALYs (Disability-Adjusted Life Years)	Republic of Sudan	Both	All ages	Diabetes and kidney diseases	Rate	2021	935
DALYs (Disability-Adjusted Life Years)	Republic of Finland	Both	All ages	Diabetes and kidney diseases	Rate	2021	1342
DALYs (Disability-Adjusted Life Years)	Sultanate of Oman	Both	All ages	Diabetes and kidney diseases	Rate	2021	1244
DALYs (Disability-Adjusted Life Years)	Gabonese Republic	Both	All ages	Diabetes and kidney diseases	Rate	2021	2468
DALYs (Disability-Adjusted Life Years)	Republic of El Salvador	Both	All ages	Diabetes and kidney diseases	Rate	2021	3374
DALYs (Disability-Adjusted Life Years)	Republic of Vanuatu	Both	All ages	Diabetes and kidney diseases	Rate	2021	2606
DALYs (Disability-Adjusted Life Years)	State of Qatar	Both	All ages	Diabetes and kidney diseases	Rate	2021	1302
DALYs (Disability-Adjusted Life Years)	People's Democratic Republic of Algeria	Both	All ages	Diabetes and kidney diseases	Rate	2021	1528
DALYs (Disability-Adjusted Life Years)	Jamaica	Both	All ages	Diabetes and kidney diseases	Rate	2021	3285
DALYs (Disability-	Republic of Armenia	Both	All ages	Diabetes and kidney diseases	Rate	2021	1585

Adjusted Life Years)							
DALYs (Disability-Adjusted Life Years)	Bosnia and Herzegovina	Both	All ages	Diabetes and kidney diseases	Rate	2021	2814
DALYs (Disability-Adjusted Life Years)	Portuguese Republic	Both	All ages	Diabetes and kidney diseases	Rate	2021	2222
DALYs (Disability-Adjusted Life Years)	Kingdom of Bhutan	Both	All ages	Diabetes and kidney diseases	Rate	2021	1385
DALYs (Disability-Adjusted Life Years)	French Republic	Both	All ages	Diabetes and kidney diseases	Rate	2021	1052
DALYs (Disability-Adjusted Life Years)	Saint Lucia	Both	All ages	Diabetes and kidney diseases	Rate	2021	4494
DALYs (Disability-Adjusted Life Years)	Republic of Korea	Both	All ages	Diabetes and kidney diseases	Rate	2021	2033
DALYs (Disability-Adjusted Life Years)	Kingdom of Saudi Arabia	Both	All ages	Diabetes and kidney diseases	Rate	2021	2130
DALYs (Disability-Adjusted Life Years)	Islamic Republic of Iran	Both	All ages	Diabetes and kidney diseases	Rate	2021	1364
DALYs (Disability-Adjusted Life Years)	Republic of Seychelles	Both	All ages	Diabetes and kidney diseases	Rate	2021	3069
DALYs (Disability-Adjusted Life Years)	Republic of Mali	Both	All ages	Diabetes and kidney diseases	Rate	2021	1194
DALYs (Disability-Adjusted Life Years)	Republic of Costa Rica	Both	All ages	Diabetes and kidney diseases	Rate	2021	2130
DALYs (Disability-Adjusted Life Years)	Republic of Bulgaria	Both	All ages	Diabetes and kidney diseases	Rate	2021	2560

DALYs (Disability-Adjusted Life Years)	Republic of Guatemala	Both	All ages	Diabetes and kidney diseases	Rate	2021	2853
DALYs (Disability-Adjusted Life Years)	Republic of Singapore	Both	All ages	Diabetes and kidney diseases	Rate	2021	1339
DALYs (Disability-Adjusted Life Years)	Georgia	Both	All ages	Diabetes and kidney diseases	Rate	2021	2073
DALYs (Disability-Adjusted Life Years)	Antigua and Barbuda	Both	All ages	Diabetes and kidney diseases	Rate	2021	3949
DALYs (Disability-Adjusted Life Years)	Socialist Republic of Viet Nam	Both	All ages	Diabetes and kidney diseases	Rate	2021	1774
DALYs (Disability-Adjusted Life Years)	Syrian Arab Republic	Both	All ages	Diabetes and kidney diseases	Rate	2021	1973
DALYs (Disability-Adjusted Life Years)	Republic of Tunisia	Both	All ages	Diabetes and kidney diseases	Rate	2021	2002
DALYs (Disability-Adjusted Life Years)	Union of the Comoros	Both	All ages	Diabetes and kidney diseases	Rate	2021	1660
DALYs (Disability-Adjusted Life Years)	Republic of Kiribati	Both	All ages	Diabetes and kidney diseases	Rate	2021	4628
DALYs (Disability-Adjusted Life Years)	Federal Republic of Germany	Both	All ages	Diabetes and kidney diseases	Rate	2021	1691
DALYs (Disability-Adjusted Life Years)	Republic of Belarus	Both	All ages	Diabetes and kidney diseases	Rate	2021	787
DALYs (Disability-Adjusted Life Years)	Republic of Zambia	Both	All ages	Diabetes and kidney diseases	Rate	2021	1292
DALYs (Disability-	Kingdom of Sweden	Both	All ages	Diabetes and kidney diseases	Rate	2021	1252

Adjusted Life Years)							
DALYs (Disability-Adjusted Life Years)	Kingdom of Spain	Both	All ages	Diabetes and kidney diseases	Rate	2021	1759
DALYs (Disability-Adjusted Life Years)	United Mexican States	Both	All ages	Diabetes and kidney diseases	Rate	2021	3956
DALYs (Disability-Adjusted Life Years)	Islamic Republic of Mauritania	Both	All ages	Diabetes and kidney diseases	Rate	2021	1081
DALYs (Disability-Adjusted Life Years)	Republic of Indonesia	Both	All ages	Diabetes and kidney diseases	Rate	2021	1698
DALYs (Disability-Adjusted Life Years)	Federal Democratic Republic of Nepal	Both	All ages	Diabetes and kidney diseases	Rate	2021	1485
DALYs (Disability-Adjusted Life Years)	Burkina Faso	Both	All ages	Diabetes and kidney diseases	Rate	2021	1162
DALYs (Disability-Adjusted Life Years)	Arab Republic of Egypt	Both	All ages	Diabetes and kidney diseases	Rate	2021	2029
DALYs (Disability-Adjusted Life Years)	Republic of Suriname	Both	All ages	Diabetes and kidney diseases	Rate	2021	3687
DALYs (Disability-Adjusted Life Years)	Islamic Republic of Pakistan	Both	All ages	Diabetes and kidney diseases	Rate	2021	1438
DALYs (Disability-Adjusted Life Years)	Federal Republic of Somalia	Both	All ages	Diabetes and kidney diseases	Rate	2021	1081
DALYs (Disability-Adjusted Life Years)	State of Eritrea	Both	All ages	Diabetes and kidney diseases	Rate	2021	1289
DALYs (Disability-Adjusted Life Years)	Republic of Croatia	Both	All ages	Diabetes and kidney diseases	Rate	2021	2130

DALYs (Disability-Adjusted Life Years)	Republic of Latvia	Both	All ages	Diabetes and kidney diseases	Rate	2021	1775
DALYs (Disability-Adjusted Life Years)	Republic of India	Both	All ages	Diabetes and kidney diseases	Rate	2021	1425
DALYs (Disability-Adjusted Life Years)	Barbados	Both	All ages	Diabetes and kidney diseases	Rate	2021	4656
DALYs (Disability-Adjusted Life Years)	Republic of Benin	Both	All ages	Diabetes and kidney diseases	Rate	2021	1148
DALYs (Disability-Adjusted Life Years)	United Republic of Tanzania	Both	All ages	Diabetes and kidney diseases	Rate	2021	1041
DALYs (Disability-Adjusted Life Years)	Saint Vincent and the Grenadines	Both	All ages	Diabetes and kidney diseases	Rate	2021	4665
DALYs (Disability-Adjusted Life Years)	Republic of Burundi	Both	All ages	Diabetes and kidney diseases	Rate	2021	944
DALYs (Disability-Adjusted Life Years)	Republic of Fiji	Both	All ages	Diabetes and kidney diseases	Rate	2021	7594
DALYs (Disability-Adjusted Life Years)	Republic of Iceland	Both	All ages	Diabetes and kidney diseases	Rate	2021	848
DALYs (Disability-Adjusted Life Years)	Republic of Cameroon	Both	All ages	Diabetes and kidney diseases	Rate	2021	1415
DALYs (Disability-Adjusted Life Years)	Republic of Uganda	Both	All ages	Diabetes and kidney diseases	Rate	2021	925
DALYs (Disability-Adjusted Life Years)	Republic of Azerbaijan	Both	All ages	Diabetes and kidney diseases	Rate	2021	1383
DALYs (Disability-	Republic of South Sudan	Both	All ages	Diabetes and kidney diseases	Rate	2021	1443

Adjusted Life Years)							
DALYs (Disability-Adjusted Life Years)	Principality of Monaco	Both	All ages	Diabetes and kidney diseases	Rate	2021	1355
DALYs (Disability-Adjusted Life Years)	Republic of Kazakhstan	Both	All ages	Diabetes and kidney diseases	Rate	2021	1173
DALYs (Disability-Adjusted Life Years)	Czech Republic	Both	All ages	Diabetes and kidney diseases	Rate	2021	1975
DALYs (Disability-Adjusted Life Years)	Swiss Confederation	Both	All ages	Diabetes and kidney diseases	Rate	2021	1510
DALYs (Disability-Adjusted Life Years)	Republic of Djibouti	Both	All ages	Diabetes and kidney diseases	Rate	2021	1279
DALYs (Disability-Adjusted Life Years)	Hungary	Both	All ages	Diabetes and kidney diseases	Rate	2021	1900
DALYs (Disability-Adjusted Life Years)	Republic of Estonia	Both	All ages	Diabetes and kidney diseases	Rate	2021	1983
DALYs (Disability-Adjusted Life Years)	Lao People's Democratic Republic	Both	All ages	Diabetes and kidney diseases	Rate	2021	1807
DALYs (Disability-Adjusted Life Years)	Kingdom of Cambodia	Both	All ages	Diabetes and kidney diseases	Rate	2021	1516
DALYs (Disability-Adjusted Life Years)	Republic of the Niger	Both	All ages	Diabetes and kidney diseases	Rate	2021	723
DALYs (Disability-Adjusted Life Years)	Republic of Niue	Both	All ages	Diabetes and kidney diseases	Rate	2021	7250
DALYs (Disability-Adjusted Life Years)	Republic of Panama	Both	All ages	Diabetes and kidney diseases	Rate	2021	2154

DALYs (Disability-Adjusted Life Years)	Republic of Honduras	Both	All ages	Diabetes and kidney diseases	Rate	2021	1438
DALYs (Disability-Adjusted Life Years)	Republic of Nauru	Both	All ages	Diabetes and kidney diseases	Rate	2021	4120
DALYs (Disability-Adjusted Life Years)	Republic of Cuba	Both	All ages	Diabetes and kidney diseases	Rate	2021	2008
DALYs (Disability-Adjusted Life Years)	Commonwealth of the Bahamas	Both	All ages	Diabetes and kidney diseases	Rate	2021	3052
DALYs (Disability-Adjusted Life Years)	Mongolia	Both	All ages	Diabetes and kidney diseases	Rate	2021	886
DALYs (Disability-Adjusted Life Years)	Republic of Trinidad and Tobago	Both	All ages	Diabetes and kidney diseases	Rate	2021	6250
DALYs (Disability-Adjusted Life Years)	Hellenic Republic	Both	All ages	Diabetes and kidney diseases	Rate	2021	2116
DALYs (Disability-Adjusted Life Years)	Republic of Chad	Both	All ages	Diabetes and kidney diseases	Rate	2021	906
DALYs (Disability-Adjusted Life Years)	United Kingdom of Great Britain and Northern Ireland	Both	All ages	Diabetes and kidney diseases	Rate	2021	1270
DALYs (Disability-Adjusted Life Years)	Federal Republic of Nigeria	Both	All ages	Diabetes and kidney diseases	Rate	2021	921
DALYs (Disability-Adjusted Life Years)	United Arab Emirates	Both	All ages	Diabetes and kidney diseases	Rate	2021	1157
DALYs (Disability-Adjusted Life Years)	Republic of Lithuania	Both	All ages	Diabetes and kidney diseases	Rate	2021	1416
DALYs (Disability-	Federal Democratic Republic of Ethiopia	Both	All ages	Diabetes and kidney diseases	Rate	2021	1003

Adjusted Life Years)							
DALYs (Disability-Adjusted Life Years)	Republic of Maldives	Both	All ages	Diabetes and kidney diseases	Rate	2021	1160
DALYs (Disability-Adjusted Life Years)	Principality of Andorra	Both	All ages	Diabetes and kidney diseases	Rate	2021	1269
DALYs (Disability-Adjusted Life Years)	Australia	Both	All ages	Diabetes and kidney diseases	Rate	2021	1116
DALYs (Disability-Adjusted Life Years)	Republic of Turkey	Both	All ages	Diabetes and kidney diseases	Rate	2021	1874
DALYs (Disability-Adjusted Life Years)	Republic of Nicaragua	Both	All ages	Diabetes and kidney diseases	Rate	2021	2446
DALYs (Disability-Adjusted Life Years)	Hashemite Kingdom of Jordan	Both	All ages	Diabetes and kidney diseases	Rate	2021	1606
DALYs (Disability-Adjusted Life Years)	Republic of Iraq	Both	All ages	Diabetes and kidney diseases	Rate	2021	1984
DALYs (Disability-Adjusted Life Years)	Republic of the Marshall Islands	Both	All ages	Diabetes and kidney diseases	Rate	2021	5431
DALYs (Disability-Adjusted Life Years)	Belize	Both	All ages	Diabetes and kidney diseases	Rate	2021	2556
DALYs (Disability-Adjusted Life Years)	Republic of Sierra Leone	Both	All ages	Diabetes and kidney diseases	Rate	2021	1024
DALYs (Disability-Adjusted Life Years)	Republic of Botswana	Both	All ages	Diabetes and kidney diseases	Rate	2021	1549
DALYs (Disability-Adjusted Life Years)	Republic of Palau	Both	All ages	Diabetes and kidney diseases	Rate	2021	6530

DALYs (Disability-Adjusted Life Years)	Plurinational State of Bolivia	Both	All ages	Diabetes and kidney diseases	Rate	2021	2192
DALYs (Disability-Adjusted Life Years)	Ireland	Both	All ages	Diabetes and kidney diseases	Rate	2021	948
DALYs (Disability-Adjusted Life Years)	New Zealand	Both	All ages	Diabetes and kidney diseases	Rate	2021	1156
DALYs (Disability-Adjusted Life Years)	Turkmenistan	Both	All ages	Diabetes and kidney diseases	Rate	2021	1503
DALYs (Disability-Adjusted Life Years)	Solomon Islands	Both	All ages	Diabetes and kidney diseases	Rate	2021	2704
DALYs (Disability-Adjusted Life Years)	Russian Federation	Both	All ages	Diabetes and kidney diseases	Rate	2021	1401
DALYs (Disability-Adjusted Life Years)	Bermuda	Both	All ages	Diabetes and kidney diseases	Rate	2021	2563
DALYs (Disability-Adjusted Life Years)	Republic of CÃ´te d'Ivoire	Both	All ages	Diabetes and kidney diseases	Rate	2021	1202
DALYs (Disability-Adjusted Life Years)	Republic of the Gambia	Both	All ages	Diabetes and kidney diseases	Rate	2021	1235
DALYs (Disability-Adjusted Life Years)	Commonwealth of Dominica	Both	All ages	Diabetes and kidney diseases	Rate	2021	4739
DALYs (Disability-Adjusted Life Years)	Republic of Madagascar	Both	All ages	Diabetes and kidney diseases	Rate	2021	848
DALYs (Disability-Adjusted Life Years)	Republic of Angola	Both	All ages	Diabetes and kidney diseases	Rate	2021	1194
DALYs (Disability-	Montenegro	Both	All ages	Diabetes and kidney diseases	Rate	2021	2027

Adjusted Life Years)							
DALYs (Disability-Adjusted Life Years)	Republic of Senegal	Both	All ages	Diabetes and kidney diseases	Rate	2021	1455
DALYs (Disability-Adjusted Life Years)	Central African Republic	Both	All ages	Diabetes and kidney diseases	Rate	2021	1766
DALYs (Disability-Adjusted Life Years)	Republic of Yemen	Both	All ages	Diabetes and kidney diseases	Rate	2021	638
DALYs (Disability-Adjusted Life Years)	State of Kuwait	Both	All ages	Diabetes and kidney diseases	Rate	2021	1566
DALYs (Disability-Adjusted Life Years)	Republic of the Philippines	Both	All ages	Diabetes and kidney diseases	Rate	2021	2043
DALYs (Disability-Adjusted Life Years)	Bolivarian Republic of Venezuela	Both	All ages	Diabetes and kidney diseases	Rate	2021	3021
DALYs (Disability-Adjusted Life Years)	Republic of the Congo	Both	All ages	Diabetes and kidney diseases	Rate	2021	1937
DALYs (Disability-Adjusted Life Years)	Republic of Malta	Both	All ages	Diabetes and kidney diseases	Rate	2021	2060
DALYs (Disability-Adjusted Life Years)	Republic of Italy	Both	All ages	Diabetes and kidney diseases	Rate	2021	1676
DALYs (Disability-Adjusted Life Years)	Independent State of Samoa	Both	All ages	Diabetes and kidney diseases	Rate	2021	3343
DALYs (Disability-Adjusted Life Years)	Independent State of Papua New Guinea	Both	All ages	Diabetes and kidney diseases	Rate	2021	2140
DALYs (Disability-Adjusted Life Years)	Republic of Chile	Both	All ages	Diabetes and kidney diseases	Rate	2021	1531

DALYs (Disability-Adjusted Life Years)	Republic of Namibia	Both	All ages	Diabetes and kidney diseases	Rate	2021	1525
DALYs (Disability-Adjusted Life Years)	Togolese Republic	Both	All ages	Diabetes and kidney diseases	Rate	2021	1067
DALYs (Disability-Adjusted Life Years)	Republic of Ghana	Both	All ages	Diabetes and kidney diseases	Rate	2021	1509
DALYs (Disability-Adjusted Life Years)	Islamic Republic of Afghanistan	Both	All ages	Diabetes and kidney diseases	Rate	2021	1408
DALYs (Disability-Adjusted Life Years)	Romania	Both	All ages	Diabetes and kidney diseases	Rate	2021	1365
DALYs (Disability-Adjusted Life Years)	Republic of Serbia	Both	All ages	Diabetes and kidney diseases	Rate	2021	2476
DALYs (Disability-Adjusted Life Years)	Republic of the Union of Myanmar	Both	All ages	Diabetes and kidney diseases	Rate	2021	2513
DALYs (Disability-Adjusted Life Years)	Republic of Ecuador	Both	All ages	Diabetes and kidney diseases	Rate	2021	2020
DALYs (Disability-Adjusted Life Years)	Saint Kitts and Nevis	Both	All ages	Diabetes and kidney diseases	Rate	2021	4035
DALYs (Disability-Adjusted Life Years)	Lebanese Republic	Both	All ages	Diabetes and kidney diseases	Rate	2021	2373
DALYs (Disability-Adjusted Life Years)	Kingdom of Lesotho	Both	All ages	Diabetes and kidney diseases	Rate	2021	2360
DALYs (Disability-Adjusted Life Years)	Puerto Rico	Both	All ages	Diabetes and kidney diseases	Rate	2021	5270
DALYs (Disability-	Republic of Kenya	Both	All ages	Diabetes and kidney diseases	Rate	2021	957

Adjusted Life Years)							
DALYs (Disability-Adjusted Life Years)	Kingdom of Tonga	Both	All ages	Diabetes and kidney diseases	Rate	2021	3332
DALYs (Disability-Adjusted Life Years)	Grenada	Both	All ages	Diabetes and kidney diseases	Rate	2021	4821
DALYs (Disability-Adjusted Life Years)	American Samoa	Both	All ages	Diabetes and kidney diseases	Rate	2021	6257
DALYs (Disability-Adjusted Life Years)	Democratic Socialist Republic of Sri Lanka	Both	All ages	Diabetes and kidney diseases	Rate	2021	3100
DALYs (Disability-Adjusted Life Years)	Republic of Equatorial Guinea	Both	All ages	Diabetes and kidney diseases	Rate	2021	1428
DALYs (Disability-Adjusted Life Years)	Kingdom of Belgium	Both	All ages	Diabetes and kidney diseases	Rate	2021	1334
DALYs (Disability-Adjusted Life Years)	Republic of Uzbekistan	Both	All ages	Diabetes and kidney diseases	Rate	2021	1506
DALYs (Disability-Adjusted Life Years)	Republic of Malawi	Both	All ages	Diabetes and kidney diseases	Rate	2021	1133
DALYs (Disability-Adjusted Life Years)	Republic of San Marino	Both	All ages	Diabetes and kidney diseases	Rate	2021	1101
DALYs (Disability-Adjusted Life Years)	Kingdom of Morocco	Both	All ages	Diabetes and kidney diseases	Rate	2021	2325
DALYs (Disability-Adjusted Life Years)	Tokelau	Both	All ages	Diabetes and kidney diseases	Rate	2021	4793
DALYs (Disability-Adjusted Life Years)	Cook Islands	Both	All ages	Diabetes and kidney diseases	Rate	2021	6504

DALYs (Disability-Adjusted Life Years)	Republic of Cyprus	Both	All ages	Diabetes and kidney diseases	Rate	2021	1758
DALYs (Disability-Adjusted Life Years)	Republic of Paraguay	Both	All ages	Diabetes and kidney diseases	Rate	2021	2077

S3: Risk factors

measure	location	sex	age	Cause	Risk factor	Rate	
**Global Metabolic risk factors Mortality**							
Deaths	Global	Both	All ages	CRD	High body-mass index	0.8	
Deaths	Global	Both	All ages	CVD	High systolic blood pressure	131.5	
Deaths	Global	Both	All ages	CVD	High body-mass index	24.1	
Deaths	Global	Both	All ages	Neo	High body-mass index	4.5	
Deaths	Global	Both	All ages	DM	High fasting plasma glucose	21.0	
Deaths	Global	Both	All ages	CVD	High LDL cholesterol	46.2	
Deaths	Global	Both	All ages	DM	High body-mass index	9.2	
Deaths	Global	Both	All ages	Neo	High fasting plasma glucose	4.2	
Deaths	Global	Both	All ages	CVD	High fasting plasma glucose	28.0	
**Global Metabolic risk factors DALYs**							
DALYs (Disability-Adjusted Life Years)	Global	Both	All ages	CVD	High fasting plasma glucose	544.3	
DALYs (Disability-Adjusted Life Years)	Global	Both	All ages	Neo	High fasting plasma glucose	89.4	
DALYs (Disability-Adjusted Life Years)	Global	Both	All ages	CVD	High body-mass index	575.7	
DALYs (Disability-Adjusted Life Years)	Global	Both	All ages	CVD	High systolic blood pressure	2718.4	
DALYs (Disability-Adjusted Life Years)	Global	Both	All ages	Neo	High body-mass index	112.7	
DALYs (Disability-Adjusted Life Years)	Global	Both	All ages	CVD	High LDL cholesterol	1111.7	
DALYs (Disability-Adjusted Life Years)	Global	Both	All ages	DM	High fasting plasma glucose	1000.2	

DALYs (Disability-Adjusted Life Years)	Global	Both	All ages	DM	High body-mass index	498.1	
DALYs (Disability-Adjusted Life Years)	Global	Both	All ages	CRD	High body-mass index	41.6	
**Global Behavioural risk factors mortality**							
Deaths	Global	Both	All ages	CRD	Tobacco	19.59	
Deaths	Global	Both	All ages	CVD	Tobacco	36.01	
Deaths	Global	Both	All ages	Neo	Low physical activity	1.11	
Deaths	Global	Both	All ages	DM	High alcohol use	0.36	
Deaths	Global	Both	All ages	CVD	Dietary risks	73.93	
Deaths	Global	Both	All ages	Neo	Tobacco	26.74	
Deaths	Global	Both	All ages	CVD	Low physical activity	4.71	
Deaths	Global	Both	All ages	CVD	High alcohol use	4.89	
Deaths	Global	Both	All ages	Neo	High alcohol use	4.35	
Deaths	Global	Both	All ages	Neo	Dietary risks	8.49	
Deaths	Global	Both	All ages	DM	Tobacco	2.07	
Deaths	Global	Both	All ages	DM	Dietary risks	4.83	
Deaths	Global	Both	All ages	DM	Low physical activity	1.89	
**Global Behavioural risk factors DALYs**							
DALYs (Disability-Adjusted Life Years)	Global	Both	All ages	CVD	Tobacco	934.0	
DALYs (Disability-Adjusted Life Years)	Global	Both	All ages	CVD	High alcohol use	117.6	
DALYs (Disability-Adjusted Life Years)	Global	Both	All ages	CVD	Low physical activity	92.4	
DALYs (Disability-Adjusted Life Years)	Global	Both	All ages	Neo	High alcohol use	118.4	
DALYs (Disability-Adjusted Life Years)	Global	Both	All ages	CVD	Dietary risks	1700.3	
DALYs (Disability-Adjusted Life Years)	Global	Both	All ages	Neo	Tobacco	641.2	
DALYs (Disability-Adjusted Life Years)	Global	Both	All ages	Neo	Low physical activity	22.1	
DALYs (Disability-Adjusted Life Years)	Global	Both	All ages	Neo	Dietary risks	207.9	

DALYs (Disability-Adjusted Life Years)	Global	Both	All ages	DM	Tobacco	112.8	
DALYs (Disability-Adjusted Life Years)	Global	Both	All ages	DM	Dietary risks	242.6	
DALYs (Disability-Adjusted Life Years)	Global	Both	All ages	DM	High alcohol use	17.2	
DALYs (Disability-Adjusted Life Years)	Global	Both	All ages	CRD	Tobacco	418.4	
DALYs (Disability-Adjusted Life Years)	Global	Both	All ages	DM	Low physical activity	70.0	
**Global Environmental (Air pollution) risk factors Moartality**							
Deaths	Global	Both	All ages	CRD	Air pollution	22.58	
Deaths	Global	Both	All ages	CVD	Air pollution	56.80	
Deaths	Global	Both	All ages	Neo	Air pollution	4.74	
Deaths	Global	Both	All ages	DM	Air pollution	3.57	
**Global Environmental (Air polluyion) risk factors DALYs**							
DALYs (Disability-Adjusted Life Years)	Global	Both	All ages	CVD	Air pollution	1262.62	
DALYs (Disability-Adjusted Life Years)	Global	Both	All ages	Neo	Air pollution	113.214	
DALYs (Disability-Adjusted Life Years)	Global	Both	All ages	DM	Air pollution	163.527	
DALYs (Disability-Adjusted Life Years)	Global	Both	All ages	CRD	Air pollution	477.6446	

S3A: Metabolic risk factors mortality and DALYS by WHO regions

Deaths Attributable to Metabolic Risk Factors for NCDs by WHO Region (Rate per 100,000, 2021)								
Region	CV + BMI	CV + LDL	CV + FPG	CV + SBP	DM + BMI	DM + FPG	Neo + BMI	Neo + F
African Region	12.09	13.7	8.53	58.4	7.64	16.94	1.26	0.81
Region of the Americas	30.86	39.15	28.08	97.32	17.05	30.26	8.83	7.21
Eastern Mediterranean Region	33.19	45.76	27.02	103.7	9.92	18.28	2.73	1.82
European Region	55.47	95.11	48.25	223.72	11.44	21.96	12.99	9.49
South-East Asia Region	12.11	39.18	24.79	114.57	7.76	24.56	1.23	1.56
Western Pacific Region	21.89	53.59	33.88	178.33	5.79	14.76	4.22	5.57
DALYs Attributable to Metabolic Risk Factors for NCDs by WHO Region (Rate per 100,000, 2021)								
African Region	329.35	381.35	179.69	1409.98	320.89	674.04	35.78	19.69
Region of the Americas	721.76	898.31	518.46	1894.37	901.54	1472	215.93	152.35
Eastern Mediterranean Region	921.54	1319.92	599.88	2486.9	587.31	981.68	80.64	46.77
European Region	1089.41	1885.28	801.23	3912.15	579.8	1011.24	285.45	186.24
South-East Asia Region	349.41	1121.16	547.79	2721.35	384.57	1027.25	36	38.59
Western Pacific Region	503.8	1200.89	627.86	3462.29	427.97	901.77	112.88	119.7

CV: Cardiovascular diseases DM: Diabetes mellitus Neo: Neoplasms CR: Chronic respiratory diseases BMI: High body-mas
High LDL cholesterol FPG: High fasting plasma glucose SBP: High systolic blood pressure

S3B: Metabolic risk factors mortality and DALYS by SDI categories

Deaths Attributable to Metabolic Risk Factors for NCDs by SDI Category (Rate per 100,000, 2021)									
SDI Category	CV + LDL	CV + BMI	CV + FPG	CV + SBP	DM + BMI	DM + FPG	Neo + BMI	Neo + FPG	CR + B
Low SDI	16.05	8.58	9.57	56.75	5.01	14.55	0.72	0.64	0.79
Low-middle SDI	35.94	17.36	22.53	105.67	9.07	23.51	1.68	1.45	1.28
Middle SDI	47.16	23.19	28.56	143.75	10.94	24.12	3.29	3.06	0.77
High-middle SDI	82.52	41.88	44.96	215.85	9.16	18.43	8.04	6.8	0.42
High SDI	49.48	32.74	35.18	124.9	9.61	19.09	11.93	11.82	0.42
DALYs Attributable to Metabolic Risk Factors for NCDs by SDI Category (Rate per 100,000, 2021)									
Low SDI	473.93	250.14	214.34	1412.32	242.58	633.52	21.71	16.08	36.4
Low-middle SDI	1021.3	481.19	493.16	2497.79	435.81	987.7	48.3	36.2	45.53
Middle SDI	1192.29	584.64	576.28	3052.04	563.84	1102.38	92.74	72.41	33.94
High-middle SDI	1742.58	884.4	797.86	3999.29	540.28	974.03	198.97	147.97	30.06
High SDI	987.27	683.8	595.86	2161.71	669.54	1196.51	260.2	225.26	70.85

S3C: Behavioural risk factors by WHO regions.

Deaths Attributable to behavioural Risk Factors for NCDs by WHO regions (Rate per 100,000, 2021)													
WHO Region	CV + Tob	CV + Alc	CV + Diet	CV + Low PA	CR + Tob	DM + Tob	DM + Alc	DM + Diet	DM + Low PA	Neo + Tob	Neo + Alc	Neo + Diet	Neo + Low PA
African Region	7.29	2.13	29.99	1.19	2.1	1.02	0.23	3.88	1.12	2.82	1.57	2.91	0.23
Region of the Americas	21.98	3.2	53.9	3.02	12.69	2.34	0.83	7.98	2.58	25.64	4.46	10.11	1.35
South-East Asia Region	31.63	1.27	75.47	4.6	24.68	2.48	0.25	4.04	2.32	11.41	2.33	4.32	0.5
European Region	50.15	12.42	122.62	8.32	13.81	1.95	0.7	7.94	2.38	47.68	9.47	17.82	2.98
Eastern Mediterranean Region	29.87	0.11	63.78	4.81	6.57	2.04	0.02	4.89	1.78	9.44	0.53	3.23	0.44
Western Pacific Region	61.16	9.55	90.05	6.08	36.27	2.14	0.28	2.94	1.28	54.43	7.08	12.8	1.47
DALYS Attributable to behavioural Risk Factors for NCDs by WHO regions (Rate per 100,000, 2021)													

African Region	221.27	54.64	791.26	28.67	64.25	43.12	6.3	152.21	35.77	80.56	49.1	87.96	5.96
Region of the Americas	575.21	75.91	1154.04	55.22	318.45	133.15	42.91	430.83	101.67	594.34	119.21	244.33	27.27
South-East Asia Region	903.36	31.71	2021.88	97.68	550.05	106.74	10.69	167.11	71	305.33	71.29	122.63	11.84
European Region	1238.08	314.99	2286.43	131.61	328.79	118.27	30.46	367.24	81.71	1137.8	235.08	379.75	51.54
Eastern Mediterranean Region	897.94	2.13	1746.01	121.72	177.86	117.69	1.07	263.56	82.63	258.84	16.65	98.77	12.42
Western Pacific Region	1456.51	219.21	1902.07	114.88	682.79	143.83	16.81	205.03	60.23	1264.66	190.94	307.48	28.92
CV: Cardiovascular diseases CR: Chronic respiratory diseases DM: Diabetes mellitus Neo: Neoplasms Tob: Tobacco use Alc: High alcohol use Diet: Dietary risks Low PA: Low physical activity													

S3D: Behavioural risk factors by SDI category

Deaths Attributable to behavioural Risk Factors for NCDs by SDI category (Rate per 100,000, 2021)											
SDI Category	CV + Tob	CV + Alc	CV + Diet	CR + Tob	DM + Tob	DM + Alc	DM + Diet	Neo + Tob	Neo + Alc	Neo + Drug	Neo + Diet
Low SDI	10.16	1.26	38.01	7.17	0.96	0.11	3.38	3.3	1.21	0.11	2.58
Low-middle SDI	28.78	1.56	66.15	20.68	2.35	0.23	4.66	9.89	1.81	0.27	3.9
Middle SDI	42.22	5.17	78.46	22.92	2.46	0.41	4.6	26.42	4.14	0.75	7.72
High-middle SDI	62.62	11.32	112.5	24.71	2.17	0.39	5.36	52.8	7.16	1.31	13.81
High SDI	29.51	6.12	67.99	16.83	1.69	0.7	6.5	49.87	9.13	2.16	17.92
DALYS Attributable to behavioural Risk Factors for NCDs by SDI category (Rate per 100,000, 2021)											
Low SDI	307.51	31.84	1040.19	171.3	42.96	2.91	144.43	93.2	37.91	3.14	77.78
Low-middle SDI	827.33	39.61	1760.22	461.82	103.5	8.79	198.31	268.73	55.95	7.71	113.64

Middle SDI	1091.44	120.58	1845.93	462.25	127.47	18	222.76	650.79	118.88	19.47	204.69
High-middle SDI	1518.97	284.87	2257.85	490.43	142.02	20.63	282.59	1277.05	194.7	32.06	327.64
High SDI	710.49	135.45	1276.8	410.96	132.22	40.81	416.91	1074.96	217.8	48.67	370.14

S3E: Environmental (Air pollution) risk factor by WHO region

Deaths Attributable to environmental risk factor (Air pollution) for NCDs by WHO category (Rate per 100,000, 2021)				
WHO Region	Chronic Respiratory Diseases	Neoplasms	Cardiovascular Diseases	Diabetes Mellitus
African Region	5.94	0.95	31.75	3.17
Region of the Americas	5.48	1.75	16.46	3.73
South-East Asia Region	43.93	2.44	69.31	4.72
European Region	6.29	4.49	44.67	2.91
Eastern Mediterranean Region	9.47	2.06	54.93	3.53
Western Pacific Region	32.12	12.23	87.13	2.79
DALYS Attributable to environmental risk factor (Air pollution) for NCDs by WHO category (Rate per 100,000, 2021)				
African Region	178.54	26.45	796.71	118.41
Region of the Americas	112.38	39.69	345.25	164.31
South-East Asia Region	968.46	65.86	1716.58	191.91
European Region	126.16	103.57	807.43	130.43
Eastern Mediterranean Region	251.07	57.12	1397.04	183.4
Western Pacific Region	590.94	281.79	1726.04	166.38

S3F: Environmental (Air pollution) risk factor by SDI category

Deaths Attributable to environmental risk factor (Air pollution) for NCDs by SDI category (Rate per 100,000, 2021)				
SDI Category	Chronic Respiratory Diseases	Neoplasms	Cardiovascular Diseases	Diabetes Mellitus
Low SDI	18.06	1.03	39.76	2.78
Low-middle SDI	35.45	2.11	63.86	4.39

Middle SDI	23.82	6.07	67.29	4.31
High-middle SDI	18.63	9.55	70.74	2.99
High SDI	6.62	4.44	21.72	1.98
DALYS Attributable to environmental risk factor (Air pollution) for NCDs by SDI category (Rate per 100,000, 2021)				
Low SDI	440.92	29.05	1022.52	113.04
Low-middle SDI	786.6	57.93	1581.39	178.72
Middle SDI	479.23	146.85	1462.8	194.94
High-middle SDI	344.74	222.68	1343.39	159.56
High SDI	128.87	90.47	403.95	122.41

S4: Gender analysis

measure	sex	cause	Rate
Deaths	Male	Chronic respiratory diseases	62.39356
Deaths	Female	Chronic respiratory diseases	49.43528
Deaths	Male	Cardiovascular diseases	258.5535
Deaths	Female	Cardiovascular diseases	233.413
Deaths	Male	Neoplasms	141.4718
Deaths	Female	Neoplasms	109.0294
Deaths	Male	Diabetes mellitus	20.11285
Deaths	Female	Diabetes mellitus	21.87936
DALYs (Disability-Adjusted Life Years)	Male	Diabetes mellitus	1012.789
DALYs (Disability-Adjusted Life Years)	Female	Diabetes mellitus	987.7585
DALYs (Disability-Adjusted Life Years)	Male	Neoplasms	3577.894
DALYs (Disability-Adjusted Life Years)	Female	Neoplasms	2839.473
DALYs (Disability-Adjusted Life Years)	Male	Cardiovascular diseases	6158.769
DALYs (Disability-Adjusted Life Years)	Female	Cardiovascular diseases	4691.745
DALYs (Disability-Adjusted Life Years)	Male	Chronic respiratory diseases	1502.233
DALYs (Disability-Adjusted Life Years)	Female	Chronic respiratory diseases	1246.801
Incidence	Male	Diabetes mellitus	320.6258
Incidence	Female	Diabetes mellitus	298.7656
Incidence	Male	Cardiovascular diseases	872.7755

Incidence	Female	Cardiovascular diseases	820.2836
Incidence	Male	Neoplasms	683.5159
Incidence	Female	Neoplasms	1002.465
Incidence	Male	Chronic respiratory diseases	707.0774
Incidence	Female	Chronic respiratory diseases	692.1937

S4A: Gender analysis by WHO region

Table 5: Gender differences in NCD burden by WHO regions 2021						
Metrics	Location	Sex	Neo	CVD	CRD	DM
Incidence	AFRO	Male	148.7	433.9	832	166
		Female	259.9	427.6	778.7	155.3
	AMRO	Male	1839.4	867.9	1052.7	473.5
		Female	2176.2	760.3	1091.1	432.7
	SAERO	Male	364.2	784.2	571.7	303.1
		Female	654.4	689	547.3	280.8
	EURO	Male	1026.1	1330.5	694.9	343.1
		Female	1484.1	1307.9	760.1	304.1
	EMRO	Male	251.2	886.1	581.9	392.6
		Female	439.7	661.9	575.7	382.2
	WPRO	Male	752.1	1002.9	655	309
		Female	1158.1	1046	582.3	295.3
Deaths	AFRO	Male	43.4	104.4	16.7	17.7
		Female	47.7	101.2	13.8	16.2
	AMRO	Male	160.4	212.4	45	30.5
		Female	143.1	188.8	42.8	30
	SAERO	Male	70.3	239.8	86.9	23.3
		Female	66.2	198.3	77.3	25.9
	EURO	Male	269.4	395.5	49.4	19.7
		Female	203.8	442.5	34.7	24.1
	EMRO	Male	58.9	198.2	26.4	16.4
		Female	54.7	179.5	16.9	20.4
	WPRO	Male	236.4	353	92.3	14
		Female	145.8	293.4	65.5	15.6
DALYS	AFRO	Male	1410.8	3066.8	702.4	722.3
		Female	1662.9	2623.8	642.3	627.6
	AMRO	Male	3834	4900.3	1233.2	1526.4
		Female	3549.2	3635.5	1215.4	1419.8
	SAERO	Male	2054.4	6409.9	2156.8	1023.5

		Female	2065.8	4802.4	1862	1031.5
	EURO	Male	6101.8	8498.5	1177.5	1005.3
		Female	4457.5	6941.4	944.4	1016.9
	EMRO	Male	1838.4	5470.9	827.4	942.7
		Female	1899.5	4523.3	634.8	1024
	WPRO	Male	5809.5	7539.9	1831.7	920.8
		Female	3526.6	5385.4	1372.2	882.2

S4B: Gender analysis by SDI category

Metrics	Location	Sex	Neo	DM	CVD	CRD
Incidence	High SDI	Male	2197.2	519.6	1166.2	956.9
		Female	2769.2	403.5	1049.9	1030.4
	High-middle SDI	Male	800.2	331.6	1163.2	616
		Female	1235	314.2	1227.5	605
	Middle SDI	Male	485.1	319.6	883.2	657.5
		Female	777.6	326.3	831.5	606.3
	Low-middle SDI	Male	298.5	284	732.2	616.4
		Female	524.3	272	629.5	600.4
	Low SDI	Male	172.6	178.8	467	834.6
		Female	303.4	163.2	421.5	804.8
Mortality	High SDI	Male	273.1	20.4	266.3	56.9
		Female	217.8	17.8	266.7	48.9
	High-middle SDI	Male	239.9	16.4	385.1	66.6
		Female	162.1	20.5	391.6	47.7
	Middle SDI	Male	136.9	22.4	286.5	69
		Female	96.9	25.8	231.3	50.1
	Low-middle SDI	Male	64.7	22.4	214.3	71
		Female	63	24.7	183.7	60.4
	Low SDI	Male	40.5	15.2	117.6	33.6
		Female	45.2	14	105.2	31.8
DALYS	High SDI	Male	5754.2	1327.5	5525.6	1422.4
		Female	4475.7	1066.2	4236.6	1342.3
	High-middle SDI	Male	5941.8	957.7	8369.4	1392.2
		Female	3927	990.6	6640.9	1080.5
	Middle SDI	Male	3656.8	1082.1	6786.5	1559.2
		Female	2709.8	1123.2	4840.5	1184.9
	Low-middle SDI	Male	1935.7	980.6	5782	1796.1
		Female	2035.2	995.2	4495.5	1506.8
	Low SDI	Male	1352.7	670.5	3455.7	1076.7
		Female	1617.3	596.9	2868.5	1038.2

S5: Global Burden of NCDs trends 2000 – 2021

Measure	Disease	2000	2005	2010	2015	2021	AAPC (%)
Incidence	Cardiovascular Diseases (CVD)	686.38	714.97	736.47	773.73	846.62	1.01
	Neoplasms (Neo)	683.33	726.75	774.12	803.86	842.44	1.01
	Chronic Respiratory Diseases (CRD)	754.17	709.86	732.19	697.16	699.66	-0.36
	Diabetes Mellitus (DM)	188.25	211.64	239.31	271.42	309.73	2.41
Deaths	Cardiovascular Diseases (CVD)	235.51	239.08	235.7	237.69	246.03	0.21
	Neoplasms (Neo)	112.29	113.91	115.16	118.57	125.31	0.53
	Chronic Respiratory Diseases (CRD)	56.13	54.42	52.86	53.48	55.94	-0.02
	Diabetes Mellitus (DM)	14.71	16.03	16.89	18.6	20.99	1.72
DALYs	Cardiovascular Diseases (CVD)	5551.49	5571.66	5389.25	5330.03	5427.81	-0.11
	Neoplasms (Neo)	3164.66	3133.12	3095.31	3119.75	3209.97	0.07
	Chronic Respiratory Diseases (CRD)	1478.22	1402.79	1356.39	1336.09	1374.96	-0.34
	Diabetes Mellitus (DM)	630.62	696.1	758.78	853.48	1000.32	2.22

S6: 2050 projections

Location	Year	Age	Sex	Cause of death or injury	Measure	Value
Afghanistan	2050	All ages	Both	Non-communicable diseases	DALYs	12896342
Angola	2050	All ages	Both	Non-communicable diseases	DALYs	10760604
Albania	2050	All ages	Both	Non-communicable diseases	DALYs	739037
Andorra	2050	All ages	Both	Non-communicable diseases	DALYs	25789.81
United Arab Emirates	2050	All ages	Both	Non-communicable diseases	DALYs	6013420
Argentina	2050	All ages	Both	Non-communicable diseases	DALYs	13200949
Armenia	2050	All ages	Both	Non-communicable diseases	DALYs	767205
American Samoa	2050	All ages	Both	Non-communicable diseases	DALYs	18766.05
Antigua and Barbuda	2050	All ages	Both	Non-communicable diseases	DALYs	30701.94
Australia	2050	All ages	Both	Non-communicable diseases	DALYs	8639505

Austria	2050	All ages	Both	Non-communicable diseases	DALYs	2732982
Azerbaijan	2050	All ages	Both	Non-communicable diseases	DALYs	3363671
Burundi	2050	All ages	Both	Non-communicable diseases	DALYs	3700350
Belgium	2050	All ages	Both	Non-communicable diseases	DALYs	3436886
Benin	2050	All ages	Both	Non-communicable diseases	DALYs	4312109
Burkina Faso	2050	All ages	Both	Non-communicable diseases	DALYs	7345455
Bangladesh	2050	All ages	Both	Non-communicable diseases	DALYs	47261843
Bulgaria	2050	All ages	Both	Non-communicable diseases	DALYs	2106238
Bahrain	2050	All ages	Both	Non-communicable diseases	DALYs	671644.2
Bahamas	2050	All ages	Both	Non-communicable diseases	DALYs	143702
Bosnia and Herzegovina	2050	All ages	Both	Non-communicable diseases	DALYs	932366.1
Belarus	2050	All ages	Both	Non-communicable diseases	DALYs	3008385
Belize	2050	All ages	Both	Non-communicable diseases	DALYs	149193
Bermuda	2050	All ages	Both	Non-communicable diseases	DALYs	19037.21
Bolivia (Plurinational State of)	2050	All ages	Both	Non-communicable diseases	DALYs	3749299
Brazil	2050	All ages	Both	Non-communicable diseases	DALYs	71910216
Barbados	2050	All ages	Both	Non-communicable diseases	DALYs	97317.68
Brunei Darussalam	2050	All ages	Both	Non-communicable diseases	DALYs	141487.6
Bhutan	2050	All ages	Both	Non-communicable diseases	DALYs	221336.7
Botswana	2050	All ages	Both	Non-communicable diseases	DALYs	714688.9
Central African Republic	2050	All ages	Both	Non-communicable diseases	DALYs	1458201
Canada	2050	All ages	Both	Non-communicable diseases	DALYs	13133940
Switzerland	2050	All ages	Both	Non-communicable diseases	DALYs	2682616
Chile	2050	All ages	Both	Non-communicable diseases	DALYs	5870615

CÃ´te d'Ivoire	2050	All ages	Both	Non-communicable diseases	DALYs	8046680
Cameroon	2050	All ages	Both	Non-communicable diseases	DALYs	9896971
Democratic Republic of the Congo	2050	All ages	Both	Non-communicable diseases	DALYs	27758487
Congo	2050	All ages	Both	Non-communicable diseases	DALYs	1862753
Cook Islands	2050	All ages	Both	Non-communicable diseases	DALYs	5773.878
Colombia	2050	All ages	Both	Non-communicable diseases	DALYs	14039562
Comoros	2050	All ages	Both	Non-communicable diseases	DALYs	210508.3
Cabo Verde	2050	All ages	Both	Non-communicable diseases	DALYs	168446.5
Costa Rica	2050	All ages	Both	Non-communicable diseases	DALYs	1420572
Cuba	2050	All ages	Both	Non-communicable diseases	DALYs	3298115
Cyprus	2050	All ages	Both	Non-communicable diseases	DALYs	416589.2
Czechia	2050	All ages	Both	Non-communicable diseases	DALYs	3096651
Germany	2050	All ages	Both	Non-communicable diseases	DALYs	26903097
Djibouti	2050	All ages	Both	Non-communicable diseases	DALYs	426376.8
Dominica	2050	All ages	Both	Non-communicable diseases	DALYs	22664.93
Denmark	2050	All ages	Both	Non-communicable diseases	DALYs	1744402
Dominican Republic	2050	All ages	Both	Non-communicable diseases	DALYs	3312099
Algeria	2050	All ages	Both	Non-communicable diseases	DALYs	13408583
Ecuador	2050	All ages	Both	Non-communicable diseases	DALYs	5359803
Eritrea	2050	All ages	Both	Non-communicable diseases	DALYs	1817250
Spain	2050	All ages	Both	Non-communicable diseases	DALYs	14713015
Estonia	2050	All ages	Both	Non-communicable diseases	DALYs	389641.4
Ethiopia	2050	All ages	Both	Non-communicable diseases	DALYs	31863109
Finland	2050	All ages	Both	Non-communicable diseases	DALYs	1599813

Fiji	2050	All ages	Both	Non-communicable diseases	DALYs	296937.5
France	2050	All ages	Both	Non-communicable diseases	DALYs	18369122
Micronesia (Federated States of)	2050	All ages	Both	Non-communicable diseases	DALYs	35240.07
Gabon	2050	All ages	Both	Non-communicable diseases	DALYs	632507.9
United Kingdom	2050	All ages	Both	Non-communicable diseases	DALYs	20899431
Georgia	2050	All ages	Both	Non-communicable diseases	DALYs	1055305
Ghana	2050	All ages	Both	Non-communicable diseases	DALYs	10602971
Guinea	2050	All ages	Both	Non-communicable diseases	DALYs	4102596
Gambia	2050	All ages	Both	Non-communicable diseases	DALYs	655097.1
Guinea-Bissau	2050	All ages	Both	Non-communicable diseases	DALYs	691675.8
Equatorial Guinea	2050	All ages	Both	Non-communicable diseases	DALYs	599941.2
Greece	2050	All ages	Both	Non-communicable diseases	DALYs	2948382
Grenada	2050	All ages	Both	Non-communicable diseases	DALYs	33986.21
Greenland	2050	All ages	Both	Non-communicable diseases	DALYs	18148.65
Guatemala	2050	All ages	Both	Non-communicable diseases	DALYs	4593831
Guam	2050	All ages	Both	Non-communicable diseases	DALYs	40987
Guyana	2050	All ages	Both	Non-communicable diseases	DALYs	202719.8
Honduras	2050	All ages	Both	Non-communicable diseases	DALYs	3191168
Croatia	2050	All ages	Both	Non-communicable diseases	DALYs	1156896
Haiti	2050	All ages	Both	Non-communicable diseases	DALYs	4435076
Hungary	2050	All ages	Both	Non-communicable diseases	DALYs	3121539
Indonesia	2050	All ages	Both	Non-communicable diseases	DALYs	87870565
India	2050	All ages	Both	Non-communicable diseases	DALYs	4.16E+08
Ireland	2050	All ages	Both	Non-communicable diseases	DALYs	1485641

Iran (Islamic Republic of)	2050	All ages	Both	Non-communicable diseases	DALYs	28026370
Iraq	2050	All ages	Both	Non-communicable diseases	DALYs	13394895
Iceland	2050	All ages	Both	Non-communicable diseases	DALYs	108322.8
Israel	2050	All ages	Both	Non-communicable diseases	DALYs	2514518
Italy	2050	All ages	Both	Non-communicable diseases	DALYs	17374575
Jamaica	2050	All ages	Both	Non-communicable diseases	DALYs	837837.1
Jordan	2050	All ages	Both	Non-communicable diseases	DALYs	4434460
Japan	2050	All ages	Both	Non-communicable diseases	DALYs	33023026
Kazakhstan	2050	All ages	Both	Non-communicable diseases	DALYs	5711180
Kenya	2050	All ages	Both	Non-communicable diseases	DALYs	13429580
Kyrgyzstan	2050	All ages	Both	Non-communicable diseases	DALYs	1914315
Cambodia	2050	All ages	Both	Non-communicable diseases	DALYs	5307548
Kiribati	2050	All ages	Both	Non-communicable diseases	DALYs	42856.39
Saint Kitts and Nevis	2050	All ages	Both	Non-communicable diseases	DALYs	19969.67
Republic of Korea	2050	All ages	Both	Non-communicable diseases	DALYs	14916617
Kuwait	2050	All ages	Both	Non-communicable diseases	DALYs	1792596
Lao People's Democratic Republic	2050	All ages	Both	Non-communicable diseases	DALYs	2291767
Lebanon	2050	All ages	Both	Non-communicable diseases	DALYs	1673632
Liberia	2050	All ages	Both	Non-communicable diseases	DALYs	1696682
Libya	2050	All ages	Both	Non-communicable diseases	DALYs	2611239
Saint Lucia	2050	All ages	Both	Non-communicable diseases	DALYs	61859.56
Sri Lanka	2050	All ages	Both	Non-communicable diseases	DALYs	5916550
Lesotho	2050	All ages	Both	Non-communicable diseases	DALYs	568555.8
Lithuania	2050	All ages	Both	Non-communicable diseases	DALYs	788872.6

Luxembourg	2050	All ages	Both	Non-communicable diseases	DALYs	231126.1
Latvia	2050	All ages	Both	Non-communicable diseases	DALYs	529988
Morocco	2050	All ages	Both	Non-communicable diseases	DALYs	12331238
Monaco	2050	All ages	Both	Non-communicable diseases	DALYs	10909.43
Republic of Moldova	2050	All ages	Both	Non-communicable diseases	DALYs	1034488
Madagascar	2050	All ages	Both	Non-communicable diseases	DALYs	8944682
Maldives	2050	All ages	Both	Non-communicable diseases	DALYs	167289
Mexico	2050	All ages	Both	Non-communicable diseases	DALYs	43949470
Marshall Islands	2050	All ages	Both	Non-communicable diseases	DALYs	23860.6
North Macedonia	2050	All ages	Both	Non-communicable diseases	DALYs	702581.4
Mali	2050	All ages	Both	Non-communicable diseases	DALYs	8102441
Malta	2050	All ages	Both	Non-communicable diseases	DALYs	141514.2
Myanmar	2050	All ages	Both	Non-communicable diseases	DALYs	17545239
Montenegro	2050	All ages	Both	Non-communicable diseases	DALYs	183892
Mongolia	2050	All ages	Both	Non-communicable diseases	DALYs	1309313
Northern Mariana Islands	2050	All ages	Both	Non-communicable diseases	DALYs	16954.11
Mozambique	2050	All ages	Both	Non-communicable diseases	DALYs	9372910
Mauritania	2050	All ages	Both	Non-communicable diseases	DALYs	1227716
Mauritius	2050	All ages	Both	Non-communicable diseases	DALYs	412824.1
Malawi	2050	All ages	Both	Non-communicable diseases	DALYs	5712215
Malaysia	2050	All ages	Both	Non-communicable diseases	DALYs	9759581
Namibia	2050	All ages	Both	Non-communicable diseases	DALYs	715047.2
Niger	2050	All ages	Both	Non-communicable diseases	DALYs	8384691
Nigeria	2050	All ages	Both	Non-communicable diseases	DALYs	65499014

Nicaragua	2050	All ages	Both	Non-communicable diseases	DALYs	1917137
Niue	2050	All ages	Both	Non-communicable diseases	DALYs	618.1874
Netherlands	2050	All ages	Both	Non-communicable diseases	DALYs	5108392
Norway	2050	All ages	Both	Non-communicable diseases	DALYs	1583561
Nepal	2050	All ages	Both	Non-communicable diseases	DALYs	8369809
Nauru	2050	All ages	Both	Non-communicable diseases	DALYs	5116.981
New Zealand	2050	All ages	Both	Non-communicable diseases	DALYs	1721240
Oman	2050	All ages	Both	Non-communicable diseases	DALYs	1809918
Pakistan	2050	All ages	Both	Non-communicable diseases	DALYs	69614079
Panama	2050	All ages	Both	Non-communicable diseases	DALYs	1344833
Peru	2050	All ages	Both	Non-communicable diseases	DALYs	9064867
Philippines	2050	All ages	Both	Non-communicable diseases	DALYs	35178763
Palau	2050	All ages	Both	Non-communicable diseases	DALYs	6982.679
Papua New Guinea	2050	All ages	Both	Non-communicable diseases	DALYs	4229482
Poland	2050	All ages	Both	Non-communicable diseases	DALYs	11590029
Puerto Rico	2050	All ages	Both	Non-communicable diseases	DALYs	891829.2
Democratic People's Republic of Korea	2050	All ages	Both	Non-communicable diseases	DALYs	8407957
Portugal	2050	All ages	Both	Non-communicable diseases	DALYs	3175699
Paraguay	2050	All ages	Both	Non-communicable diseases	DALYs	2135040
Palestine	2050	All ages	Both	Non-communicable diseases	DALYs	1520952
Qatar	2050	All ages	Both	Non-communicable diseases	DALYs	1633901
Romania	2050	All ages	Both	Non-communicable diseases	DALYs	5540790
Russian Federation	2050	All ages	Both	Non-communicable diseases	DALYs	47563102
Rwanda	2050	All ages	Both	Non-communicable diseases	DALYs	4266901

Saudi Arabia	2050	All ages	Both	Non-communicable diseases	DALYs	16024486
Senegal	2050	All ages	Both	Non-communicable diseases	DALYs	4518795
Singapore	2050	All ages	Both	Non-communicable diseases	DALYs	1677978
Solomon Islands	2050	All ages	Both	Non-communicable diseases	DALYs	234538.7
Sierra Leone	2050	All ages	Both	Non-communicable diseases	DALYs	2522711
El Salvador	2050	All ages	Both	Non-communicable diseases	DALYs	1614761
San Marino	2050	All ages	Both	Non-communicable diseases	DALYs	9154.661
Somalia	2050	All ages	Both	Non-communicable diseases	DALYs	6710004
Serbia	2050	All ages	Both	Non-communicable diseases	DALYs	2737849
South Sudan	2050	All ages	Both	Non-communicable diseases	DALYs	3412622
Sao Tome and Principe	2050	All ages	Both	Non-communicable diseases	DALYs	55149.47
Suriname	2050	All ages	Both	Non-communicable diseases	DALYs	191978.4
Slovakia	2050	All ages	Both	Non-communicable diseases	DALYs	1663679
Slovenia	2050	All ages	Both	Non-communicable diseases	DALYs	608482
Sweden	2050	All ages	Both	Non-communicable diseases	DALYs	3146475
Eswatini	2050	All ages	Both	Non-communicable diseases	DALYs	328893
Seychelles	2050	All ages	Both	Non-communicable diseases	DALYs	39023.11
Syrian Arab Republic	2050	All ages	Both	Non-communicable diseases	DALYs	4634986
Chad	2050	All ages	Both	Non-communicable diseases	DALYs	6476918
Togo	2050	All ages	Both	Non-communicable diseases	DALYs	2440454
Thailand	2050	All ages	Both	Non-communicable diseases	DALYs	20180188
Tajikistan	2050	All ages	Both	Non-communicable diseases	DALYs	2827534
Tokelau	2050	All ages	Both	Non-communicable diseases	DALYs	485.5558
Turkmenistan	2050	All ages	Both	Non-communicable diseases	DALYs	1681392

Timor-Leste	2050	All ages	Both	Non-communicable diseases	DALYs	363094.4
Tonga	2050	All ages	Both	Non-communicable diseases	DALYs	28920.92
Trinidad and Tobago	2050	All ages	Both	Non-communicable diseases	DALYs	406630.3
Tunisia	2050	All ages	Both	Non-communicable diseases	DALYs	3706264
Turkey	2050	All ages	Both	Non-communicable diseases	DALYs	25546408
Tuvalu	2050	All ages	Both	Non-communicable diseases	DALYs	4731.282
Taiwan (Province of China)	2050	All ages	Both	Non-communicable diseases	DALYs	7414676
United Republic of Tanzania	2050	All ages	Both	Non-communicable diseases	DALYs	17586237
Uganda	2050	All ages	Both	Non-communicable diseases	DALYs	13155380
Ukraine	2050	All ages	Both	Non-communicable diseases	DALYs	13772907
Uruguay	2050	All ages	Both	Non-communicable diseases	DALYs	996174.8
United States of America	2050	All ages	Both	Non-communicable diseases	DALYs	1.19E+08
Uzbekistan	2050	All ages	Both	Non-communicable diseases	DALYs	10488758
Saint Vincent and the Grenadines	2050	All ages	Both	Non-communicable diseases	DALYs	36317.29
Venezuela (Bolivarian Republic of)	2050	All ages	Both	Non-communicable diseases	DALYs	8245756
United States Virgin Islands	2050	All ages	Both	Non-communicable diseases	DALYs	25577.95
Viet Nam	2050	All ages	Both	Non-communicable diseases	DALYs	32841423
Vanuatu	2050	All ages	Both	Non-communicable diseases	DALYs	116447.7
Samoa	2050	All ages	Both	Non-communicable diseases	DALYs	68859.23
Yemen	2050	All ages	Both	Non-communicable diseases	DALYs	10833593
South Africa	2050	All ages	Both	Non-communicable diseases	DALYs	17480627
Zambia	2050	All ages	Both	Non-communicable diseases	DALYs	6895952
Zimbabwe	2050	All ages	Both	Non-communicable diseases	DALYs	4434474
Egypt	2050	All ages	Both	Non-communicable diseases	DALYs	40948162

Sudan	2050	All ages	Both	Non-communicable diseases	DALYs	14082048
China	2050	All ages	Both	Non-communicable diseases	DALYs	4.41E+08
						2.44E+09

S6A: country-based burden of CVDs mortality projection 2050

Country	Cause of death	Measure	Rate
Afghanistan	Cardiovascular diseases	Deaths per 100,000	144.7859
Angola	Cardiovascular diseases	Deaths per 100,000	103.42
Albania	Cardiovascular diseases	Deaths per 100,000	774.6019
Andorra	Cardiovascular diseases	Deaths per 100,000	405.4582
United Arab Emirates	Cardiovascular diseases	Deaths per 100,000	325.3679
Argentina	Cardiovascular diseases	Deaths per 100,000	237.8296
Armenia	Cardiovascular diseases	Deaths per 100,000	589.1922
American Samoa	Cardiovascular diseases	Deaths per 100,000	269.9445
Antigua and Barbuda	Cardiovascular diseases	Deaths per 100,000	381.0828
Australia	Cardiovascular diseases	Deaths per 100,000	210.2542
Austria	Cardiovascular diseases	Deaths per 100,000	377.2452
Azerbaijan	Cardiovascular diseases	Deaths per 100,000	611.8386
Burundi	Cardiovascular diseases	Deaths per 100,000	100.5574
Belgium	Cardiovascular diseases	Deaths per 100,000	245.218
Benin	Cardiovascular diseases	Deaths per 100,000	81.42638
Burkina Faso	Cardiovascular diseases	Deaths per 100,000	77.10866
Bangladesh	Cardiovascular diseases	Deaths per 100,000	340.726
Bulgaria	Cardiovascular diseases	Deaths per 100,000	1051.618
Bahrain	Cardiovascular diseases	Deaths per 100,000	208.1229

Bahamas	Cardiovascular diseases	Deaths per 100,000	390.4939
Bosnia and Herzegovina	Cardiovascular diseases	Deaths per 100,000	759.1026
Belarus	Cardiovascular diseases	Deaths per 100,000	803.5896
Belize	Cardiovascular diseases	Deaths per 100,000	164.5744
Bermuda	Cardiovascular diseases	Deaths per 100,000	517.9357
Bolivia (Plurinational State of)	Cardiovascular diseases	Deaths per 100,000	148.6953
Brazil	Cardiovascular diseases	Deaths per 100,000	230.6811
Barbados	Cardiovascular diseases	Deaths per 100,000	413.8336
Brunei Darussalam	Cardiovascular diseases	Deaths per 100,000	263.581
Bhutan	Cardiovascular diseases	Deaths per 100,000	225.9134
Botswana	Cardiovascular diseases	Deaths per 100,000	191.49
Central African Republic	Cardiovascular diseases	Deaths per 100,000	165.7879
Canada	Cardiovascular diseases	Deaths per 100,000	235.6644
Switzerland	Cardiovascular diseases	Deaths per 100,000	307.9495
Chile	Cardiovascular diseases	Deaths per 100,000	205.4616
CÃ´te d'Ivoire	Cardiovascular diseases	Deaths per 100,000	122.4999
Cameroon	Cardiovascular diseases	Deaths per 100,000	124.255
Democratic Republic of the Congo	Cardiovascular diseases	Deaths per 100,000	129.3798
Congo	Cardiovascular diseases	Deaths per 100,000	222.5691
Cook Islands	Cardiovascular diseases	Deaths per 100,000	410.5707
Colombia	Cardiovascular diseases	Deaths per 100,000	237.3668
Comoros	Cardiovascular diseases	Deaths per 100,000	191.1824
Cabo Verde	Cardiovascular diseases	Deaths per 100,000	280.7533
Costa Rica	Cardiovascular diseases	Deaths per 100,000	211.9824

Cuba	Cardiovascular diseases	Deaths per 100,000	532.4303
Cyprus	Cardiovascular diseases	Deaths per 100,000	327.2358
Czechia	Cardiovascular diseases	Deaths per 100,000	474.583
Germany	Cardiovascular diseases	Deaths per 100,000	400.9515
Djibouti	Cardiovascular diseases	Deaths per 100,000	162.8064
Dominica	Cardiovascular diseases	Deaths per 100,000	419.2709
Denmark	Cardiovascular diseases	Deaths per 100,000	206.8229
Dominican Republic	Cardiovascular diseases	Deaths per 100,000	310.3492
Algeria	Cardiovascular diseases	Deaths per 100,000	362.0409
Ecuador	Cardiovascular diseases	Deaths per 100,000	174.995
Eritrea	Cardiovascular diseases	Deaths per 100,000	139.2519
Spain	Cardiovascular diseases	Deaths per 100,000	290.563
Estonia	Cardiovascular diseases	Deaths per 100,000	554.8411
Ethiopia	Cardiovascular diseases	Deaths per 100,000	63.86019
Finland	Cardiovascular diseases	Deaths per 100,000	438.6293
Fiji	Cardiovascular diseases	Deaths per 100,000	352.9087
France	Cardiovascular diseases	Deaths per 100,000	293.9552
Micronesia (Federated States of)	Cardiovascular diseases	Deaths per 100,000	445.6034
Gabon	Cardiovascular diseases	Deaths per 100,000	169.1965
United Kingdom	Cardiovascular diseases	Deaths per 100,000	240.2685
Georgia	Cardiovascular diseases	Deaths per 100,000	566.8909
Ghana	Cardiovascular diseases	Deaths per 100,000	168.4208
Guinea	Cardiovascular diseases	Deaths per 100,000	101.4609
Gambia	Cardiovascular diseases	Deaths per 100,000	139.0466

Guinea-Bissau	Cardiovascular diseases	Deaths per 100,000	134.3077
Equatorial Guinea	Cardiovascular diseases	Deaths per 100,000	116.2187
Greece	Cardiovascular diseases	Deaths per 100,000	496.6122
Grenada	Cardiovascular diseases	Deaths per 100,000	361.7369
Greenland	Cardiovascular diseases	Deaths per 100,000	284.951
Guatemala	Cardiovascular diseases	Deaths per 100,000	150.9637
Guam	Cardiovascular diseases	Deaths per 100,000	312.7189
Guyana	Cardiovascular diseases	Deaths per 100,000	371.6581
Honduras	Cardiovascular diseases	Deaths per 100,000	226.936
Croatia	Cardiovascular diseases	Deaths per 100,000	667.1621
Haiti	Cardiovascular diseases	Deaths per 100,000	279.4412
Hungary	Cardiovascular diseases	Deaths per 100,000	548.9844
Indonesia	Cardiovascular diseases	Deaths per 100,000	369.6414
India	Cardiovascular diseases	Deaths per 100,000	235.4891
Ireland	Cardiovascular diseases	Deaths per 100,000	207.5108
Iran (Islamic Republic of)	Cardiovascular diseases	Deaths per 100,000	338.732
Iraq	Cardiovascular diseases	Deaths per 100,000	254.9737
Iceland	Cardiovascular diseases	Deaths per 100,000	214.2582
Israel	Cardiovascular diseases	Deaths per 100,000	122.0453
Italy	Cardiovascular diseases	Deaths per 100,000	484.1019
Jamaica	Cardiovascular diseases	Deaths per 100,000	368.8239
Jordan	Cardiovascular diseases	Deaths per 100,000	159.8859
Japan	Cardiovascular diseases	Deaths per 100,000	305.3799
Kazakhstan	Cardiovascular diseases	Deaths per 100,000	388.9481

Kenya	Cardiovascular diseases	Deaths per 100,000	107.8816
Kyrgyzstan	Cardiovascular diseases	Deaths per 100,000	386.5138
Cambodia	Cardiovascular diseases	Deaths per 100,000	243.3262
Kiribati	Cardiovascular diseases	Deaths per 100,000	261.2245
Saint Kitts and Nevis	Cardiovascular diseases	Deaths per 100,000	425.3614
Republic of Korea	Cardiovascular diseases	Deaths per 100,000	307.595
Kuwait	Cardiovascular diseases	Deaths per 100,000	224.5173
Lao People's Democratic Republic	Cardiovascular diseases	Deaths per 100,000	255.9718
Lebanon	Cardiovascular diseases	Deaths per 100,000	234.1429
Liberia	Cardiovascular diseases	Deaths per 100,000	157.3478
Libya	Cardiovascular diseases	Deaths per 100,000	427.2724
Saint Lucia	Cardiovascular diseases	Deaths per 100,000	400.3562
Sri Lanka	Cardiovascular diseases	Deaths per 100,000	386.8235
Lesotho	Cardiovascular diseases	Deaths per 100,000	195.261
Lithuania	Cardiovascular diseases	Deaths per 100,000	809.9596
Luxembourg	Cardiovascular diseases	Deaths per 100,000	219.9604
Latvia	Cardiovascular diseases	Deaths per 100,000	796.3137
Morocco	Cardiovascular diseases	Deaths per 100,000	521.5994
Monaco	Cardiovascular diseases	Deaths per 100,000	436.3845
Republic of Moldova	Cardiovascular diseases	Deaths per 100,000	941.5599
Madagascar	Cardiovascular diseases	Deaths per 100,000	164.9089
Maldives	Cardiovascular diseases	Deaths per 100,000	183.366
Mexico	Cardiovascular diseases	Deaths per 100,000	221.8885
Marshall Islands	Cardiovascular diseases	Deaths per 100,000	368.4408

North Macedonia	Cardiovascular diseases	Deaths per 100,000	856.0841
Mali	Cardiovascular diseases	Deaths per 100,000	63.91856
Malta	Cardiovascular diseases	Deaths per 100,000	272.7476
Myanmar	Cardiovascular diseases	Deaths per 100,000	321.5822
Montenegro	Cardiovascular diseases	Deaths per 100,000	808.9954
Mongolia	Cardiovascular diseases	Deaths per 100,000	247.8961
Northern Mariana Islands	Cardiovascular diseases	Deaths per 100,000	383.5306
Mozambique	Cardiovascular diseases	Deaths per 100,000	103.722
Mauritania	Cardiovascular diseases	Deaths per 100,000	128.197
Mauritius	Cardiovascular diseases	Deaths per 100,000	360.5942
Malawi	Cardiovascular diseases	Deaths per 100,000	103.0715
Malaysia	Cardiovascular diseases	Deaths per 100,000	217.765
Namibia	Cardiovascular diseases	Deaths per 100,000	190.8041
Niger	Cardiovascular diseases	Deaths per 100,000	71.15686
Nigeria	Cardiovascular diseases	Deaths per 100,000	93.8006
Nicaragua	Cardiovascular diseases	Deaths per 100,000	214.9196
Niue	Cardiovascular diseases	Deaths per 100,000	437.3861
Netherlands	Cardiovascular diseases	Deaths per 100,000	288.1889
Norway	Cardiovascular diseases	Deaths per 100,000	219.5738
Nepal	Cardiovascular diseases	Deaths per 100,000	220.9981
Nauru	Cardiovascular diseases	Deaths per 100,000	356.9256
New Zealand	Cardiovascular diseases	Deaths per 100,000	249.9466
Oman	Cardiovascular diseases	Deaths per 100,000	284.0841
Pakistan	Cardiovascular diseases	Deaths per 100,000	204.9051

Panama	Cardiovascular diseases	Deaths per 100,000	163.2656
Peru	Cardiovascular diseases	Deaths per 100,000	123.177
Philippines	Cardiovascular diseases	Deaths per 100,000	242.2994
Palau	Cardiovascular diseases	Deaths per 100,000	541.3285
Papua New Guinea	Cardiovascular diseases	Deaths per 100,000	181.8296
Poland	Cardiovascular diseases	Deaths per 100,000	517.7232
Puerto Rico	Cardiovascular diseases	Deaths per 100,000	362.6618
Democratic People's Republic of Korea	Cardiovascular diseases	Deaths per 100,000	592.9232
Portugal	Cardiovascular diseases	Deaths per 100,000	295.326
Paraguay	Cardiovascular diseases	Deaths per 100,000	224.573
Palestine	Cardiovascular diseases	Deaths per 100,000	206.2094
Qatar	Cardiovascular diseases	Deaths per 100,000	140.9994
Romania	Cardiovascular diseases	Deaths per 100,000	798.7099
Russian Federation	Cardiovascular diseases	Deaths per 100,000	616.846
Rwanda	Cardiovascular diseases	Deaths per 100,000	97.53278
Saudi Arabia	Cardiovascular diseases	Deaths per 100,000	392.6447
Senegal	Cardiovascular diseases	Deaths per 100,000	124.2303
Singapore	Cardiovascular diseases	Deaths per 100,000	162.5857
Solomon Islands	Cardiovascular diseases	Deaths per 100,000	271.3683
Sierra Leone	Cardiovascular diseases	Deaths per 100,000	121.0468
El Salvador	Cardiovascular diseases	Deaths per 100,000	250.9582
San Marino	Cardiovascular diseases	Deaths per 100,000	397.7382
Somalia	Cardiovascular diseases	Deaths per 100,000	78.10557
Serbia	Cardiovascular diseases	Deaths per 100,000	736.9834

South Sudan	Cardiovascular diseases	Deaths per 100,000	77.15403
Sao Tome and Principe	Cardiovascular diseases	Deaths per 100,000	196.6725
Suriname	Cardiovascular diseases	Deaths per 100,000	304.8548
Slovakia	Cardiovascular diseases	Deaths per 100,000	591.4675
Slovenia	Cardiovascular diseases	Deaths per 100,000	448.0425
Sweden	Cardiovascular diseases	Deaths per 100,000	256.3128
Eswatini	Cardiovascular diseases	Deaths per 100,000	175.9212
Seychelles	Cardiovascular diseases	Deaths per 100,000	287.8461
Syrian Arab Republic	Cardiovascular diseases	Deaths per 100,000	524.7975
Chad	Cardiovascular diseases	Deaths per 100,000	65.07703
Togo	Cardiovascular diseases	Deaths per 100,000	153.1866
Thailand	Cardiovascular diseases	Deaths per 100,000	309.0475
Tajikistan	Cardiovascular diseases	Deaths per 100,000	319.3147
Tokelau	Cardiovascular diseases	Deaths per 100,000	346.9786
Turkmenistan	Cardiovascular diseases	Deaths per 100,000	428.4451
Timor-Leste	Cardiovascular diseases	Deaths per 100,000	191.5666
Tonga	Cardiovascular diseases	Deaths per 100,000	156.1308
Trinidad and Tobago	Cardiovascular diseases	Deaths per 100,000	480.4462
Tunisia	Cardiovascular diseases	Deaths per 100,000	490.6098
Turkey	Cardiovascular diseases	Deaths per 100,000	332.6081
Tuvalu	Cardiovascular diseases	Deaths per 100,000	359.6018
Taiwan (Province of China)	Cardiovascular diseases	Deaths per 100,000	286.6508
United Republic of Tanzania	Cardiovascular diseases	Deaths per 100,000	103.9095
Uganda	Cardiovascular diseases	Deaths per 100,000	57.89265

Ukraine	Cardiovascular diseases	Deaths per 100,000	1203.77
Uruguay	Cardiovascular diseases	Deaths per 100,000	271.1903
United States of America	Cardiovascular diseases	Deaths per 100,000	318.7065
Uzbekistan	Cardiovascular diseases	Deaths per 100,000	458.7862
Saint Vincent and the Grenadines	Cardiovascular diseases	Deaths per 100,000	471.1235
Venezuela (Bolivarian Republic of)	Cardiovascular diseases	Deaths per 100,000	538.6562
United States Virgin Islands	Cardiovascular diseases	Deaths per 100,000	719.5802
Viet Nam	Cardiovascular diseases	Deaths per 100,000	399.5762
Vanuatu	Cardiovascular diseases	Deaths per 100,000	270.6016
Samoa	Cardiovascular diseases	Deaths per 100,000	248.168
Yemen	Cardiovascular diseases	Deaths per 100,000	238.9347
South Africa	Cardiovascular diseases	Deaths per 100,000	234.9353
Zambia	Cardiovascular diseases	Deaths per 100,000	116.6172
Zimbabwe	Cardiovascular diseases	Deaths per 100,000	151.5633
Egypt	Cardiovascular diseases	Deaths per 100,000	308.9751
Sudan	Cardiovascular diseases	Deaths per 100,000	235.3846
China	Cardiovascular diseases	Deaths per 100,000	557.1183

Afghanistan	2050	Cardiovascular diseases	DALYs per 100,000	4052
Angola	2050	Cardiovascular diseases	DALYs per 100,000	2708
Albania	2050	Cardiovascular diseases	DALYs per 100,000	10801
Andorra	2050	Cardiovascular diseases	DALYs per 100,000	5695
United Arab Emirates	2050	Cardiovascular diseases	DALYs per 100,000	6706
Argentina	2050	Cardiovascular diseases	DALYs per 100,000	4482
Armenia	2050	Cardiovascular diseases	DALYs per 100,000	8872

American Samoa	2050	Cardiovascular diseases	DALYs per 100,000	6298
Antigua and Barbuda	2050	Cardiovascular diseases	DALYs per 100,000	6830
Australia	2050	Cardiovascular diseases	DALYs per 100,000	3168
Austria	2050	Cardiovascular diseases	DALYs per 100,000	5406
Azerbaijan	2050	Cardiovascular diseases	DALYs per 100,000	10561
Burundi	2050	Cardiovascular diseases	DALYs per 100,000	2767
Belgium	2050	Cardiovascular diseases	DALYs per 100,000	3593
Benin	2050	Cardiovascular diseases	DALYs per 100,000	2152
Burkina Faso	2050	Cardiovascular diseases	DALYs per 100,000	2059
Bangladesh	2050	Cardiovascular diseases	DALYs per 100,000	6595
Bulgaria	2050	Cardiovascular diseases	DALYs per 100,000	16635
Bahrain	2050	Cardiovascular diseases	DALYs per 100,000	4026
Bahamas	2050	Cardiovascular diseases	DALYs per 100,000	7695
Bosnia and Herzegovina	2050	Cardiovascular diseases	DALYs per 100,000	10925
Belarus	2050	Cardiovascular diseases	DALYs per 100,000	12751
Belize	2050	Cardiovascular diseases	DALYs per 100,000	3632
Bermuda	2050	Cardiovascular diseases	DALYs per 100,000	7370
Bolivia (Plurinational State of)	2050	Cardiovascular diseases	DALYs per 100,000	3235
Brazil	2050	Cardiovascular diseases	DALYs per 100,000	4583
Barbados	2050	Cardiovascular diseases	DALYs per 100,000	6870
Brunei Darussalam	2050	Cardiovascular diseases	DALYs per 100,000	5270
Bhutan	2050	Cardiovascular diseases	DALYs per 100,000	4728
Botswana	2050	Cardiovascular diseases	DALYs per 100,000	4456
Central African Republic	2050	Cardiovascular diseases	DALYs per 100,000	4476

Canada	2050	Cardiovascular diseases	DALYs per 100,000	3744
Switzerland	2050	Cardiovascular diseases	DALYs per 100,000	3972
Chile	2050	Cardiovascular diseases	DALYs per 100,000	3374
CÃ´te d'Ivoire	2050	Cardiovascular diseases	DALYs per 100,000	3197
Cameroon	2050	Cardiovascular diseases	DALYs per 100,000	3340
Democratic Republic of the Congo	2050	Cardiovascular diseases	DALYs per 100,000	3278
Congo	2050	Cardiovascular diseases	DALYs per 100,000	5502
Cook Islands	2050	Cardiovascular diseases	DALYs per 100,000	7490
Colombia	2050	Cardiovascular diseases	DALYs per 100,000	4031
Comoros	2050	Cardiovascular diseases	DALYs per 100,000	4409
Cabo Verde	2050	Cardiovascular diseases	DALYs per 100,000	5880
Costa Rica	2050	Cardiovascular diseases	DALYs per 100,000	3755
Cuba	2050	Cardiovascular diseases	DALYs per 100,000	8224
Cyprus	2050	Cardiovascular diseases	DALYs per 100,000	4537
Czechia	2050	Cardiovascular diseases	DALYs per 100,000	7138
Germany	2050	Cardiovascular diseases	DALYs per 100,000	5726
Djibouti	2050	Cardiovascular diseases	DALYs per 100,000	4087
Dominica	2050	Cardiovascular diseases	DALYs per 100,000	7739
Denmark	2050	Cardiovascular diseases	DALYs per 100,000	3103
Dominican Republic	2050	Cardiovascular diseases	DALYs per 100,000	6015
Algeria	2050	Cardiovascular diseases	DALYs per 100,000	6109
Ecuador	2050	Cardiovascular diseases	DALYs per 100,000	3253
Eritrea	2050	Cardiovascular diseases	DALYs per 100,000	3682
Spain	2050	Cardiovascular diseases	DALYs per 100,000	4328

Estonia	2050	Cardiovascular diseases	DALYs per 100,000	7588
Ethiopia	2050	Cardiovascular diseases	DALYs per 100,000	1772
Finland	2050	Cardiovascular diseases	DALYs per 100,000	5944
Fiji	2050	Cardiovascular diseases	DALYs per 100,000	8204
France	2050	Cardiovascular diseases	DALYs per 100,000	4609
Micronesia (Federated States of)	2050	Cardiovascular diseases	DALYs per 100,000	11343
Gabon	2050	Cardiovascular diseases	DALYs per 100,000	4043
United Kingdom	2050	Cardiovascular diseases	DALYs per 100,000	3689
Georgia	2050	Cardiovascular diseases	DALYs per 100,000	9203
Ghana	2050	Cardiovascular diseases	DALYs per 100,000	4372
Guinea	2050	Cardiovascular diseases	DALYs per 100,000	2756
Gambia	2050	Cardiovascular diseases	DALYs per 100,000	3644
Guinea-Bissau	2050	Cardiovascular diseases	DALYs per 100,000	3916
Equatorial Guinea	2050	Cardiovascular diseases	DALYs per 100,000	3164
Greece	2050	Cardiovascular diseases	DALYs per 100,000	6816
Grenada	2050	Cardiovascular diseases	DALYs per 100,000	7049
Greenland	2050	Cardiovascular diseases	DALYs per 100,000	4817
Guatemala	2050	Cardiovascular diseases	DALYs per 100,000	2897
Guam	2050	Cardiovascular diseases	DALYs per 100,000	6574
Guyana	2050	Cardiovascular diseases	DALYs per 100,000	7446
Honduras	2050	Cardiovascular diseases	DALYs per 100,000	4774
Croatia	2050	Cardiovascular diseases	DALYs per 100,000	9130
Haiti	2050	Cardiovascular diseases	DALYs per 100,000	6783
Hungary	2050	Cardiovascular diseases	DALYs per 100,000	8339

Indonesia	2050	Cardiovascular diseases	DALYs per 100,000	7565
India	2050	Cardiovascular diseases	DALYs per 100,000	5155
Ireland	2050	Cardiovascular diseases	DALYs per 100,000	2972
Iran (Islamic Republic of)	2050	Cardiovascular diseases	DALYs per 100,000	5921
Iraq	2050	Cardiovascular diseases	DALYs per 100,000	5312
Iceland	2050	Cardiovascular diseases	DALYs per 100,000	2991
Israel	2050	Cardiovascular diseases	DALYs per 100,000	2043
Italy	2050	Cardiovascular diseases	DALYs per 100,000	6411
Jamaica	2050	Cardiovascular diseases	DALYs per 100,000	6876
Jordan	2050	Cardiovascular diseases	DALYs per 100,000	3111
Japan	2050	Cardiovascular diseases	DALYs per 100,000	4457
Kazakhstan	2050	Cardiovascular diseases	DALYs per 100,000	6903
Kenya	2050	Cardiovascular diseases	DALYs per 100,000	2684
Kyrgyzstan	2050	Cardiovascular diseases	DALYs per 100,000	7068
Cambodia	2050	Cardiovascular diseases	DALYs per 100,000	5147
Kiribati	2050	Cardiovascular diseases	DALYs per 100,000	7520
Saint Kitts and Nevis	2050	Cardiovascular diseases	DALYs per 100,000	7446
Republic of Korea	2050	Cardiovascular diseases	DALYs per 100,000	4505
Kuwait	2050	Cardiovascular diseases	DALYs per 100,000	4504
Lao People's Democratic Republic	2050	Cardiovascular diseases	DALYs per 100,000	5722
Lebanon	2050	Cardiovascular diseases	DALYs per 100,000	4342
Liberia	2050	Cardiovascular diseases	DALYs per 100,000	4058
Libya	2050	Cardiovascular diseases	DALYs per 100,000	8960
Saint Lucia	2050	Cardiovascular diseases	DALYs per 100,000	6840

Sri Lanka	2050	Cardiovascular diseases	DALYs per 100,000	6281
Lesotho	2050	Cardiovascular diseases	DALYs per 100,000	5342
Lithuania	2050	Cardiovascular diseases	DALYs per 100,000	11376
Luxembourg	2050	Cardiovascular diseases	DALYs per 100,000	3359
Latvia	2050	Cardiovascular diseases	DALYs per 100,000	11691
Morocco	2050	Cardiovascular diseases	DALYs per 100,000	9944
Monaco	2050	Cardiovascular diseases	DALYs per 100,000	6034
Republic of Moldova	2050	Cardiovascular diseases	DALYs per 100,000	14790
Madagascar	2050	Cardiovascular diseases	DALYs per 100,000	4551
Maldives	2050	Cardiovascular diseases	DALYs per 100,000	3746
Mexico	2050	Cardiovascular diseases	DALYs per 100,000	4055
Marshall Islands	2050	Cardiovascular diseases	DALYs per 100,000	10336
North Macedonia	2050	Cardiovascular diseases	DALYs per 100,000	13546
Mali	2050	Cardiovascular diseases	DALYs per 100,000	1815
Malta	2050	Cardiovascular diseases	DALYs per 100,000	3785
Myanmar	2050	Cardiovascular diseases	DALYs per 100,000	6597
Montenegro	2050	Cardiovascular diseases	DALYs per 100,000	12256
Mongolia	2050	Cardiovascular diseases	DALYs per 100,000	5188
Northern Mariana Islands	2050	Cardiovascular diseases	DALYs per 100,000	7731
Mozambique	2050	Cardiovascular diseases	DALYs per 100,000	2947
Mauritania	2050	Cardiovascular diseases	DALYs per 100,000	3151
Mauritius	2050	Cardiovascular diseases	DALYs per 100,000	6296
Malawi	2050	Cardiovascular diseases	DALYs per 100,000	2927
Malaysia	2050	Cardiovascular diseases	DALYs per 100,000	4789

Namibia	2050	Cardiovascular diseases	DALYs per 100,000	4529
Niger	2050	Cardiovascular diseases	DALYs per 100,000	1892
Nigeria	2050	Cardiovascular diseases	DALYs per 100,000	2344
Nicaragua	2050	Cardiovascular diseases	DALYs per 100,000	3735
Niue	2050	Cardiovascular diseases	DALYs per 100,000	9221
Netherlands	2050	Cardiovascular diseases	DALYs per 100,000	4085
Norway	2050	Cardiovascular diseases	DALYs per 100,000	3312
Nepal	2050	Cardiovascular diseases	DALYs per 100,000	4523
Nauru	2050	Cardiovascular diseases	DALYs per 100,000	11183
New Zealand	2050	Cardiovascular diseases	DALYs per 100,000	3596
Oman	2050	Cardiovascular diseases	DALYs per 100,000	6180
Pakistan	2050	Cardiovascular diseases	DALYs per 100,000	4914
Panama	2050	Cardiovascular diseases	DALYs per 100,000	2931
Peru	2050	Cardiovascular diseases	DALYs per 100,000	2507
Philippines	2050	Cardiovascular diseases	DALYs per 100,000	5446
Palau	2050	Cardiovascular diseases	DALYs per 100,000	11583
Papua New Guinea	2050	Cardiovascular diseases	DALYs per 100,000	4952
Poland	2050	Cardiovascular diseases	DALYs per 100,000	7648
Puerto Rico	2050	Cardiovascular diseases	DALYs per 100,000	5497
Democratic People's Republic of Korea	2050	Cardiovascular diseases	DALYs per 100,000	11023
Portugal	2050	Cardiovascular diseases	DALYs per 100,000	4135
Paraguay	2050	Cardiovascular diseases	DALYs per 100,000	4556
Palestine	2050	Cardiovascular diseases	DALYs per 100,000	4173
Qatar	2050	Cardiovascular diseases	DALYs per 100,000	2983

Romania	2050	Cardiovascular diseases	DALYs per 100,000	11958
Russian Federation	2050	Cardiovascular diseases	DALYs per 100,000	10203
Rwanda	2050	Cardiovascular diseases	DALYs per 100,000	2453
Saudi Arabia	2050	Cardiovascular diseases	DALYs per 100,000	9107
Senegal	2050	Cardiovascular diseases	DALYs per 100,000	3089
Singapore	2050	Cardiovascular diseases	DALYs per 100,000	2849
Solomon Islands	2050	Cardiovascular diseases	DALYs per 100,000	7315
Sierra Leone	2050	Cardiovascular diseases	DALYs per 100,000	3244
El Salvador	2050	Cardiovascular diseases	DALYs per 100,000	4510
San Marino	2050	Cardiovascular diseases	DALYs per 100,000	5841
Somalia	2050	Cardiovascular diseases	DALYs per 100,000	2471
Serbia	2050	Cardiovascular diseases	DALYs per 100,000	11197
South Sudan	2050	Cardiovascular diseases	DALYs per 100,000	2239
Sao Tome and Principe	2050	Cardiovascular diseases	DALYs per 100,000	4770
Suriname	2050	Cardiovascular diseases	DALYs per 100,000	5818
Slovakia	2050	Cardiovascular diseases	DALYs per 100,000	9071
Slovenia	2050	Cardiovascular diseases	DALYs per 100,000	5823
Sweden	2050	Cardiovascular diseases	DALYs per 100,000	3916
Eswatini	2050	Cardiovascular diseases	DALYs per 100,000	4678
Seychelles	2050	Cardiovascular diseases	DALYs per 100,000	5559
Syrian Arab Republic	2050	Cardiovascular diseases	DALYs per 100,000	9759
Chad	2050	Cardiovascular diseases	DALYs per 100,000	1949
Togo	2050	Cardiovascular diseases	DALYs per 100,000	3873
Thailand	2050	Cardiovascular diseases	DALYs per 100,000	5320

Tajikistan	2050	Cardiovascular diseases	DALYs per 100,000	5933
Tokelau	2050	Cardiovascular diseases	DALYs per 100,000	7628
Turkmenistan	2050	Cardiovascular diseases	DALYs per 100,000	8482
Timor-Leste	2050	Cardiovascular diseases	DALYs per 100,000	4330
Tonga	2050	Cardiovascular diseases	DALYs per 100,000	3543
Trinidad and Tobago	2050	Cardiovascular diseases	DALYs per 100,000	8087
Tunisia	2050	Cardiovascular diseases	DALYs per 100,000	8045
Turkey	2050	Cardiovascular diseases	DALYs per 100,000	5467
Tuvalu	2050	Cardiovascular diseases	DALYs per 100,000	9329
Taiwan (Province of China)	2050	Cardiovascular diseases	DALYs per 100,000	5009
United Republic of Tanzania	2050	Cardiovascular diseases	DALYs per 100,000	2664
Uganda	2050	Cardiovascular diseases	DALYs per 100,000	1653
Ukraine	2050	Cardiovascular diseases	DALYs per 100,000	18688
Uruguay	2050	Cardiovascular diseases	DALYs per 100,000	4584
United States of America	2050	Cardiovascular diseases	DALYs per 100,000	5247
Uzbekistan	2050	Cardiovascular diseases	DALYs per 100,000	8458
Saint Vincent and the Grenadines	2050	Cardiovascular diseases	DALYs per 100,000	8342
Venezuela (Bolivarian Republic of)	2050	Cardiovascular diseases	DALYs per 100,000	10766
United States Virgin Islands	2050	Cardiovascular diseases	DALYs per 100,000	9377
Viet Nam	2050	Cardiovascular diseases	DALYs per 100,000	7690
Vanuatu	2050	Cardiovascular diseases	DALYs per 100,000	7509
Samoa	2050	Cardiovascular diseases	DALYs per 100,000	6059
Yemen	2050	Cardiovascular diseases	DALYs per 100,000	5754
South Africa	2050	Cardiovascular diseases	DALYs per 100,000	5093

Zambia	2050	Cardiovascular diseases	DALYs per 100,000	3161
Zimbabwe	2050	Cardiovascular diseases	DALYs per 100,000	4179
Egypt	2050	Cardiovascular diseases	DALYs per 100,000	7263
Sudan	2050	Cardiovascular diseases	DALYs per 100,000	5652
China	2050	Cardiovascular diseases	DALYs per 100,000	8606

S6B: country-based burden of Neoplasm mortality projection 2050

Country	Year	Age	Sex	Cause of death or injury	Measure	Rate
Afghanistan	2050	All ages	Both	Neoplasms	Deaths per 100,000	64.9
Angola	2050	All ages	Both	Neoplasms	Deaths per 100,000	64.4
Albania	2050	All ages	Both	Neoplasms	Deaths per 100,000	325
Andorra	2050	All ages	Both	Neoplasms	Deaths per 100,000	577.3
United Arab Emirates	2050	All ages	Both	Neoplasms	Deaths per 100,000	255.4
Argentina	2050	All ages	Both	Neoplasms	Deaths per 100,000	283.2
Armenia	2050	All ages	Both	Neoplasms	Deaths per 100,000	342.8
American Samoa	2050	All ages	Both	Neoplasms	Deaths per 100,000	215.4
Antigua and Barbuda	2050	All ages	Both	Neoplasms	Deaths per 100,000	328
Australia	2050	All ages	Both	Neoplasms	Deaths per 100,000	296.2
Austria	2050	All ages	Both	Neoplasms	Deaths per 100,000	335.6
Azerbaijan	2050	All ages	Both	Neoplasms	Deaths per 100,000	184.2
Burundi	2050	All ages	Both	Neoplasms	Deaths per 100,000	60.1
Belgium	2050	All ages	Both	Neoplasms	Deaths per 100,000	346.9
Benin	2050	All ages	Both	Neoplasms	Deaths per 100,000	50.1
Burkina Faso	2050	All ages	Both	Neoplasms	Deaths per 100,000	51.6

Bangladesh	2050	All ages	Both	Neoplasms	Deaths per 100,000	100.7
Bulgaria	2050	All ages	Both	Neoplasms	Deaths per 100,000	436.1
Bahrain	2050	All ages	Both	Neoplasms	Deaths per 100,000	219.1
Bahamas	2050	All ages	Both	Neoplasms	Deaths per 100,000	298.3
Bosnia and Herzegovina	2050	All ages	Both	Neoplasms	Deaths per 100,000	509
Belarus	2050	All ages	Both	Neoplasms	Deaths per 100,000	344
Belize	2050	All ages	Both	Neoplasms	Deaths per 100,000	130.5
Bermuda	2050	All ages	Both	Neoplasms	Deaths per 100,000	557.5
Bolivia (Plurinational State of)	2050	All ages	Both	Neoplasms	Deaths per 100,000	193.3
Brazil	2050	All ages	Both	Neoplasms	Deaths per 100,000	225.1
Barbados	2050	All ages	Both	Neoplasms	Deaths per 100,000	416.7
Brunei Darussalam	2050	All ages	Both	Neoplasms	Deaths per 100,000	321.8
Bhutan	2050	All ages	Both	Neoplasms	Deaths per 100,000	123.7
Botswana	2050	All ages	Both	Neoplasms	Deaths per 100,000	165.6
Central African Republic	2050	All ages	Both	Neoplasms	Deaths per 100,000	74.1
Canada	2050	All ages	Both	Neoplasms	Deaths per 100,000	335.1
Switzerland	2050	All ages	Both	Neoplasms	Deaths per 100,000	327.4
Chile	2050	All ages	Both	Neoplasms	Deaths per 100,000	283
CÃ´te d'Ivoire	2050	All ages	Both	Neoplasms	Deaths per 100,000	60.5
Cameroon	2050	All ages	Both	Neoplasms	Deaths per 100,000	71.7
Democratic Republic of the Congo	2050	All ages	Both	Neoplasms	Deaths per 100,000	64.5
Congo	2050	All ages	Both	Neoplasms	Deaths per 100,000	121.4
Cook Islands	2050	All ages	Both	Neoplasms	Deaths per 100,000	276.9
Colombia	2050	All ages	Both	Neoplasms	Deaths per 100,000	200.3

Comoros	2050	All ages	Both	Neoplasms	Deaths per 100,000	151
Cabo Verde	2050	All ages	Both	Neoplasms	Deaths per 100,000	268
Costa Rica	2050	All ages	Both	Neoplasms	Deaths per 100,000	268.5
Cuba	2050	All ages	Both	Neoplasms	Deaths per 100,000	455.1
Cyprus	2050	All ages	Both	Neoplasms	Deaths per 100,000	286
Czechia	2050	All ages	Both	Neoplasms	Deaths per 100,000	432.4
Germany	2050	All ages	Both	Neoplasms	Deaths per 100,000	389.3
Djibouti	2050	All ages	Both	Neoplasms	Deaths per 100,000	140.3
Dominica	2050	All ages	Both	Neoplasms	Deaths per 100,000	360.1
Denmark	2050	All ages	Both	Neoplasms	Deaths per 100,000	386.6
Dominican Republic	2050	All ages	Both	Neoplasms	Deaths per 100,000	177.7
Algeria	2050	All ages	Both	Neoplasms	Deaths per 100,000	86.5
Ecuador	2050	All ages	Both	Neoplasms	Deaths per 100,000	172.9
Eritrea	2050	All ages	Both	Neoplasms	Deaths per 100,000	109.1
Spain	2050	All ages	Both	Neoplasms	Deaths per 100,000	351.7
Estonia	2050	All ages	Both	Neoplasms	Deaths per 100,000	394.9
Ethiopia	2050	All ages	Both	Neoplasms	Deaths per 100,000	75.4
Finland	2050	All ages	Both	Neoplasms	Deaths per 100,000	371.6
Fiji	2050	All ages	Both	Neoplasms	Deaths per 100,000	148.9
France	2050	All ages	Both	Neoplasms	Deaths per 100,000	434.1
Micronesia (Federated States of)	2050	All ages	Both	Neoplasms	Deaths per 100,000	166
Gabon	2050	All ages	Both	Neoplasms	Deaths per 100,000	123.4
United Kingdom	2050	All ages	Both	Neoplasms	Deaths per 100,000	402.8
Georgia	2050	All ages	Both	Neoplasms	Deaths per 100,000	337.8

Ghana	2050	All ages	Both	Neoplasms	Deaths per 100,000	82.6
Guinea	2050	All ages	Both	Neoplasms	Deaths per 100,000	57.7
Gambia	2050	All ages	Both	Neoplasms	Deaths per 100,000	61.7
Guinea-Bissau	2050	All ages	Both	Neoplasms	Deaths per 100,000	75.3
Equatorial Guinea	2050	All ages	Both	Neoplasms	Deaths per 100,000	83.5
Greece	2050	All ages	Both	Neoplasms	Deaths per 100,000	558.8
Grenada	2050	All ages	Both	Neoplasms	Deaths per 100,000	361.9
Greenland	2050	All ages	Both	Neoplasms	Deaths per 100,000	391.5
Guatemala	2050	All ages	Both	Neoplasms	Deaths per 100,000	111.5
Guam	2050	All ages	Both	Neoplasms	Deaths per 100,000	167.4
Guyana	2050	All ages	Both	Neoplasms	Deaths per 100,000	206.5
Honduras	2050	All ages	Both	Neoplasms	Deaths per 100,000	125.4
Croatia	2050	All ages	Both	Neoplasms	Deaths per 100,000	590.1
Haiti	2050	All ages	Both	Neoplasms	Deaths per 100,000	140.6
Hungary	2050	All ages	Both	Neoplasms	Deaths per 100,000	417.2
Indonesia	2050	All ages	Both	Neoplasms	Deaths per 100,000	170.8
India	2050	All ages	Both	Neoplasms	Deaths per 100,000	118.4
Ireland	2050	All ages	Both	Neoplasms	Deaths per 100,000	284.4
Iran (Islamic Republic of)	2050	All ages	Both	Neoplasms	Deaths per 100,000	174.2
Iraq	2050	All ages	Both	Neoplasms	Deaths per 100,000	110.1
Iceland	2050	All ages	Both	Neoplasms	Deaths per 100,000	274.6
Israel	2050	All ages	Both	Neoplasms	Deaths per 100,000	201.3
Italy	2050	All ages	Both	Neoplasms	Deaths per 100,000	518.8
Jamaica	2050	All ages	Both	Neoplasms	Deaths per 100,000	305.4

Jordan	2050	All ages	Both	Neoplasms	Deaths per 100,000	86.2
Japan	2050	All ages	Both	Neoplasms	Deaths per 100,000	501.7
Kazakhstan	2050	All ages	Both	Neoplasms	Deaths per 100,000	135.3
Kenya	2050	All ages	Both	Neoplasms	Deaths per 100,000	87.2
Kyrgyzstan	2050	All ages	Both	Neoplasms	Deaths per 100,000	120.7
Cambodia	2050	All ages	Both	Neoplasms	Deaths per 100,000	160.4
Kiribati	2050	All ages	Both	Neoplasms	Deaths per 100,000	115.6
Saint Kitts and Nevis	2050	All ages	Both	Neoplasms	Deaths per 100,000	379.2
Republic of Korea	2050	All ages	Both	Neoplasms	Deaths per 100,000	472.8
Kuwait	2050	All ages	Both	Neoplasms	Deaths per 100,000	126.3
Lao People's Democratic Republic	2050	All ages	Both	Neoplasms	Deaths per 100,000	140.9
Lebanon	2050	All ages	Both	Neoplasms	Deaths per 100,000	247.1
Liberia	2050	All ages	Both	Neoplasms	Deaths per 100,000	76.4
Libya	2050	All ages	Both	Neoplasms	Deaths per 100,000	312.9
Saint Lucia	2050	All ages	Both	Neoplasms	Deaths per 100,000	338.9
Sri Lanka	2050	All ages	Both	Neoplasms	Deaths per 100,000	141.9
Lesotho	2050	All ages	Both	Neoplasms	Deaths per 100,000	159.9
Lithuania	2050	All ages	Both	Neoplasms	Deaths per 100,000	542.4
Luxembourg	2050	All ages	Both	Neoplasms	Deaths per 100,000	290.5
Latvia	2050	All ages	Both	Neoplasms	Deaths per 100,000	483.6
Morocco	2050	All ages	Both	Neoplasms	Deaths per 100,000	105.9
Monaco	2050	All ages	Both	Neoplasms	Deaths per 100,000	790.7
Republic of Moldova	2050	All ages	Both	Neoplasms	Deaths per 100,000	364.2
Madagascar	2050	All ages	Both	Neoplasms	Deaths per 100,000	66.3

Maldives	2050	All ages	Both	Neoplasms	Deaths per 100,000	92.6
Mexico	2050	All ages	Both	Neoplasms	Deaths per 100,000	166.4
Marshall Islands	2050	All ages	Both	Neoplasms	Deaths per 100,000	138.2
North Macedonia	2050	All ages	Both	Neoplasms	Deaths per 100,000	464.6
Mali	2050	All ages	Both	Neoplasms	Deaths per 100,000	48.9
Malta	2050	All ages	Both	Neoplasms	Deaths per 100,000	300.5
Myanmar	2050	All ages	Both	Neoplasms	Deaths per 100,000	134.6
Montenegro	2050	All ages	Both	Neoplasms	Deaths per 100,000	407.8
Mongolia	2050	All ages	Both	Neoplasms	Deaths per 100,000	258.5
Northern Mariana Islands	2050	All ages	Both	Neoplasms	Deaths per 100,000	290.1
Mozambique	2050	All ages	Both	Neoplasms	Deaths per 100,000	74.1
Mauritania	2050	All ages	Both	Neoplasms	Deaths per 100,000	78.6
Mauritius	2050	All ages	Both	Neoplasms	Deaths per 100,000	245.9
Malawi	2050	All ages	Both	Neoplasms	Deaths per 100,000	88
Malaysia	2050	All ages	Both	Neoplasms	Deaths per 100,000	192.6
Namibia	2050	All ages	Both	Neoplasms	Deaths per 100,000	103.9
Niger	2050	All ages	Both	Neoplasms	Deaths per 100,000	25.7
Nigeria	2050	All ages	Both	Neoplasms	Deaths per 100,000	57.1
Nicaragua	2050	All ages	Both	Neoplasms	Deaths per 100,000	119.2
Niue	2050	All ages	Both	Neoplasms	Deaths per 100,000	230.9
Netherlands	2050	All ages	Both	Neoplasms	Deaths per 100,000	383.4
Norway	2050	All ages	Both	Neoplasms	Deaths per 100,000	338.2
Nepal	2050	All ages	Both	Neoplasms	Deaths per 100,000	101.2
Nauru	2050	All ages	Both	Neoplasms	Deaths per 100,000	120.8

New Zealand	2050	All ages	Both	Neoplasms	Deaths per 100,000	297.7
Oman	2050	All ages	Both	Neoplasms	Deaths per 100,000	76.8
Pakistan	2050	All ages	Both	Neoplasms	Deaths per 100,000	130.3
Panama	2050	All ages	Both	Neoplasms	Deaths per 100,000	165.8
Peru	2050	All ages	Both	Neoplasms	Deaths per 100,000	185.9
Philippines	2050	All ages	Both	Neoplasms	Deaths per 100,000	126.6
Palau	2050	All ages	Both	Neoplasms	Deaths per 100,000	402.3
Papua New Guinea	2050	All ages	Both	Neoplasms	Deaths per 100,000	58.2
Poland	2050	All ages	Both	Neoplasms	Deaths per 100,000	552.1
Puerto Rico	2050	All ages	Both	Neoplasms	Deaths per 100,000	356.6
Democratic People's Republic of Korea	2050	All ages	Both	Neoplasms	Deaths per 100,000	237
Portugal	2050	All ages	Both	Neoplasms	Deaths per 100,000	408.5
Paraguay	2050	All ages	Both	Neoplasms	Deaths per 100,000	193.7
Palestine	2050	All ages	Both	Neoplasms	Deaths per 100,000	119
Qatar	2050	All ages	Both	Neoplasms	Deaths per 100,000	162.9
Romania	2050	All ages	Both	Neoplasms	Deaths per 100,000	406.2
Russian Federation	2050	All ages	Both	Neoplasms	Deaths per 100,000	304.2
Rwanda	2050	All ages	Both	Neoplasms	Deaths per 100,000	94.2
Saudi Arabia	2050	All ages	Both	Neoplasms	Deaths per 100,000	133.9
Senegal	2050	All ages	Both	Neoplasms	Deaths per 100,000	75.7
Singapore	2050	All ages	Both	Neoplasms	Deaths per 100,000	258.8
Solomon Islands	2050	All ages	Both	Neoplasms	Deaths per 100,000	87.4
Sierra Leone	2050	All ages	Both	Neoplasms	Deaths per 100,000	59.3
El Salvador	2050	All ages	Both	Neoplasms	Deaths per 100,000	190.3

San Marino	2050	All ages	Both	Neoplasms	Deaths per 100,000	446.1
Somalia	2050	All ages	Both	Neoplasms	Deaths per 100,000	51.3
Serbia	2050	All ages	Both	Neoplasms	Deaths per 100,000	475.4
South Sudan	2050	All ages	Both	Neoplasms	Deaths per 100,000	60.7
Sao Tome and Principe	2050	All ages	Both	Neoplasms	Deaths per 100,000	119.1
Suriname	2050	All ages	Both	Neoplasms	Deaths per 100,000	203.5
Slovakia	2050	All ages	Both	Neoplasms	Deaths per 100,000	429.9
Slovenia	2050	All ages	Both	Neoplasms	Deaths per 100,000	422.7
Sweden	2050	All ages	Both	Neoplasms	Deaths per 100,000	293.3
Eswatini	2050	All ages	Both	Neoplasms	Deaths per 100,000	167.6
Seychelles	2050	All ages	Both	Neoplasms	Deaths per 100,000	261
Syrian Arab Republic	2050	All ages	Both	Neoplasms	Deaths per 100,000	103.4
Chad	2050	All ages	Both	Neoplasms	Deaths per 100,000	34.2
Togo	2050	All ages	Both	Neoplasms	Deaths per 100,000	78.9
Thailand	2050	All ages	Both	Neoplasms	Deaths per 100,000	372.3
Tajikistan	2050	All ages	Both	Neoplasms	Deaths per 100,000	71.7
Tokelau	2050	All ages	Both	Neoplasms	Deaths per 100,000	192.1
Turkmenistan	2050	All ages	Both	Neoplasms	Deaths per 100,000	104.2
Timor-Leste	2050	All ages	Both	Neoplasms	Deaths per 100,000	80.4
Tonga	2050	All ages	Both	Neoplasms	Deaths per 100,000	156.2
Trinidad and Tobago	2050	All ages	Both	Neoplasms	Deaths per 100,000	326.8
Tunisia	2050	All ages	Both	Neoplasms	Deaths per 100,000	178.6
Turkey	2050	All ages	Both	Neoplasms	Deaths per 100,000	295.4
Tuvalu	2050	All ages	Both	Neoplasms	Deaths per 100,000	124.9

Taiwan (Province of China)	2050	All ages	Both	Neoplasms	Deaths per 100,000	492.7
United Republic of Tanzania	2050	All ages	Both	Neoplasms	Deaths per 100,000	85.4
Uganda	2050	All ages	Both	Neoplasms	Deaths per 100,000	91.9
Ukraine	2050	All ages	Both	Neoplasms	Deaths per 100,000	300.8
Uruguay	2050	All ages	Both	Neoplasms	Deaths per 100,000	439.6
United States of America	2050	All ages	Both	Neoplasms	Deaths per 100,000	312.5
Uzbekistan	2050	All ages	Both	Neoplasms	Deaths per 100,000	88.3
Saint Vincent and the Grenadines	2050	All ages	Both	Neoplasms	Deaths per 100,000	350.3
Venezuela (Bolivarian Republic of)	2050	All ages	Both	Neoplasms	Deaths per 100,000	215.9
United States Virgin Islands	2050	All ages	Both	Neoplasms	Deaths per 100,000	435.4
Viet Nam	2050	All ages	Both	Neoplasms	Deaths per 100,000	217.5
Vanuatu	2050	All ages	Both	Neoplasms	Deaths per 100,000	77.9
Samoa	2050	All ages	Both	Neoplasms	Deaths per 100,000	75.5
Yemen	2050	All ages	Both	Neoplasms	Deaths per 100,000	66.1
South Africa	2050	All ages	Both	Neoplasms	Deaths per 100,000	192.9
Zambia	2050	All ages	Both	Neoplasms	Deaths per 100,000	103.4
Zimbabwe	2050	All ages	Both	Neoplasms	Deaths per 100,000	133.5
Egypt	2050	All ages	Both	Neoplasms	Deaths per 100,000	103.4
Sudan	2050	All ages	Both	Neoplasms	Deaths per 100,000	68.7
China	2050	All ages	Both	Neoplasms	Deaths per 100,000	405
Afghanistan	2050	All ages	Both	Neoplasms	DALYs per 100,000	2179
Angola	2050	All ages	Both	Neoplasms	DALYs per 100,000	1902
Albania	2050	All ages	Both	Neoplasms	DALYs per 100,000	6009
Andorra	2050	All ages	Both	Neoplasms	DALYs per 100,000	9306

United Arab Emirates	2050	All ages	Both	Neoplasms	DALYs per 100,000	5400
Argentina	2050	All ages	Both	Neoplasms	DALYs per 100,000	5935
Armenia	2050	All ages	Both	Neoplasms	DALYs per 100,000	6807
American Samoa	2050	All ages	Both	Neoplasms	DALYs per 100,000	4871
Antigua and Barbuda	2050	All ages	Both	Neoplasms	DALYs per 100,000	6329
Australia	2050	All ages	Both	Neoplasms	DALYs per 100,000	5099
Austria	2050	All ages	Both	Neoplasms	DALYs per 100,000	5636
Azerbaijan	2050	All ages	Both	Neoplasms	DALYs per 100,000	4394
Burundi	2050	All ages	Both	Neoplasms	DALYs per 100,000	1887
Belgium	2050	All ages	Both	Neoplasms	DALYs per 100,000	5935
Benin	2050	All ages	Both	Neoplasms	DALYs per 100,000	1449
Burkina Faso	2050	All ages	Both	Neoplasms	DALYs per 100,000	1564
Bangladesh	2050	All ages	Both	Neoplasms	DALYs per 100,000	2484
Bulgaria	2050	All ages	Both	Neoplasms	DALYs per 100,000	9194
Bahrain	2050	All ages	Both	Neoplasms	DALYs per 100,000	4501
Bahamas	2050	All ages	Both	Neoplasms	DALYs per 100,000	6392
Bosnia and Herzegovina	2050	All ages	Both	Neoplasms	DALYs per 100,000	9353
Belarus	2050	All ages	Both	Neoplasms	DALYs per 100,000	7392
Belize	2050	All ages	Both	Neoplasms	DALYs per 100,000	3177
Bermuda	2050	All ages	Both	Neoplasms	DALYs per 100,000	8446
Bolivia (Plurinational State of)	2050	All ages	Both	Neoplasms	DALYs per 100,000	4510
Brazil	2050	All ages	Both	Neoplasms	DALYs per 100,000	4702
Barbados	2050	All ages	Both	Neoplasms	DALYs per 100,000	7645
Brunei Darussalam	2050	All ages	Both	Neoplasms	DALYs per 100,000	6823

Bhutan	2050	All ages	Both	Neoplasms	DALYs per 100,000	3001
Botswana	2050	All ages	Both	Neoplasms	DALYs per 100,000	4070
Central African Republic	2050	All ages	Both	Neoplasms	DALYs per 100,000	2292
Canada	2050	All ages	Both	Neoplasms	DALYs per 100,000	5487
Switzerland	2050	All ages	Both	Neoplasms	DALYs per 100,000	5230
Chile	2050	All ages	Both	Neoplasms	DALYs per 100,000	5062
CÃ´te d'Ivoire	2050	All ages	Both	Neoplasms	DALYs per 100,000	1604
Cameroon	2050	All ages	Both	Neoplasms	DALYs per 100,000	2041
Democratic Republic of the Congo	2050	All ages	Both	Neoplasms	DALYs per 100,000	1862
Congo	2050	All ages	Both	Neoplasms	DALYs per 100,000	3357
Cook Islands	2050	All ages	Both	Neoplasms	DALYs per 100,000	5059
Colombia	2050	All ages	Both	Neoplasms	DALYs per 100,000	4060
Comoros	2050	All ages	Both	Neoplasms	DALYs per 100,000	3875
Cabo Verde	2050	All ages	Both	Neoplasms	DALYs per 100,000	5806
Costa Rica	2050	All ages	Both	Neoplasms	DALYs per 100,000	5339
Cuba	2050	All ages	Both	Neoplasms	DALYs per 100,000	8093
Cyprus	2050	All ages	Both	Neoplasms	DALYs per 100,000	5164
Czechia	2050	All ages	Both	Neoplasms	DALYs per 100,000	7731
Germany	2050	All ages	Both	Neoplasms	DALYs per 100,000	6683
Djibouti	2050	All ages	Both	Neoplasms	DALYs per 100,000	3705
Dominica	2050	All ages	Both	Neoplasms	DALYs per 100,000	7345
Denmark	2050	All ages	Both	Neoplasms	DALYs per 100,000	6400
Dominican Republic	2050	All ages	Both	Neoplasms	DALYs per 100,000	3789
Algeria	2050	All ages	Both	Neoplasms	DALYs per 100,000	1857

Ecuador	2050	All ages	Both	Neoplasms	DALYs per 100,000	3622
Eritrea	2050	All ages	Both	Neoplasms	DALYs per 100,000	3218
Spain	2050	All ages	Both	Neoplasms	DALYs per 100,000	5937
Estonia	2050	All ages	Both	Neoplasms	DALYs per 100,000	7116
Ethiopia	2050	All ages	Both	Neoplasms	DALYs per 100,000	2209
Finland	2050	All ages	Both	Neoplasms	DALYs per 100,000	6104
Fiji	2050	All ages	Both	Neoplasms	DALYs per 100,000	3636
France	2050	All ages	Both	Neoplasms	DALYs per 100,000	7209
Micronesia (Federated States of)	2050	All ages	Both	Neoplasms	DALYs per 100,000	4248
Gabon	2050	All ages	Both	Neoplasms	DALYs per 100,000	3306
United Kingdom	2050	All ages	Both	Neoplasms	DALYs per 100,000	6669
Georgia	2050	All ages	Both	Neoplasms	DALYs per 100,000	7401
Ghana	2050	All ages	Both	Neoplasms	DALYs per 100,000	2233
Guinea	2050	All ages	Both	Neoplasms	DALYs per 100,000	1790
Gambia	2050	All ages	Both	Neoplasms	DALYs per 100,000	1889
Guinea-Bissau	2050	All ages	Both	Neoplasms	DALYs per 100,000	2372
Equatorial Guinea	2050	All ages	Both	Neoplasms	DALYs per 100,000	2483
Greece	2050	All ages	Both	Neoplasms	DALYs per 100,000	9436
Grenada	2050	All ages	Both	Neoplasms	DALYs per 100,000	7486
Greenland	2050	All ages	Both	Neoplasms	DALYs per 100,000	7619
Guatemala	2050	All ages	Both	Neoplasms	DALYs per 100,000	2625
Guam	2050	All ages	Both	Neoplasms	DALYs per 100,000	3485
Guyana	2050	All ages	Both	Neoplasms	DALYs per 100,000	4626
Honduras	2050	All ages	Both	Neoplasms	DALYs per 100,000	3197

Croatia	2050	All ages	Both	Neoplasms	DALYs per 100,000	10386
Haiti	2050	All ages	Both	Neoplasms	DALYs per 100,000	3810
Hungary	2050	All ages	Both	Neoplasms	DALYs per 100,000	8284
Indonesia	2050	All ages	Both	Neoplasms	DALYs per 100,000	3976
India	2050	All ages	Both	Neoplasms	DALYs per 100,000	3039
Ireland	2050	All ages	Both	Neoplasms	DALYs per 100,000	4864
Iran (Islamic Republic of)	2050	All ages	Both	Neoplasms	DALYs per 100,000	3582
Iraq	2050	All ages	Both	Neoplasms	DALYs per 100,000	2808
Iceland	2050	All ages	Both	Neoplasms	DALYs per 100,000	4771
Israel	2050	All ages	Both	Neoplasms	DALYs per 100,000	3555
Italy	2050	All ages	Both	Neoplasms	DALYs per 100,000	8202
Jamaica	2050	All ages	Both	Neoplasms	DALYs per 100,000	6424
Jordan	2050	All ages	Both	Neoplasms	DALYs per 100,000	2014
Japan	2050	All ages	Both	Neoplasms	DALYs per 100,000	7367
Kazakhstan	2050	All ages	Both	Neoplasms	DALYs per 100,000	3208
Kenya	2050	All ages	Both	Neoplasms	DALYs per 100,000	2452
Kyrgyzstan	2050	All ages	Both	Neoplasms	DALYs per 100,000	3002
Cambodia	2050	All ages	Both	Neoplasms	DALYs per 100,000	4154
Kiribati	2050	All ages	Both	Neoplasms	DALYs per 100,000	3310
Saint Kitts and Nevis	2050	All ages	Both	Neoplasms	DALYs per 100,000	7151
Republic of Korea	2050	All ages	Both	Neoplasms	DALYs per 100,000	6984
Kuwait	2050	All ages	Both	Neoplasms	DALYs per 100,000	2609
Lao People's Democratic Republic	2050	All ages	Both	Neoplasms	DALYs per 100,000	3669
Lebanon	2050	All ages	Both	Neoplasms	DALYs per 100,000	5068

Liberia	2050	All ages	Both	Neoplasms	DALYs per 100,000	2131
Libya	2050	All ages	Both	Neoplasms	DALYs per 100,000	7135
Saint Lucia	2050	All ages	Both	Neoplasms	DALYs per 100,000	6370
Sri Lanka	2050	All ages	Both	Neoplasms	DALYs per 100,000	2893
Lesotho	2050	All ages	Both	Neoplasms	DALYs per 100,000	4888
Lithuania	2050	All ages	Both	Neoplasms	DALYs per 100,000	10061
Luxembourg	2050	All ages	Both	Neoplasms	DALYs per 100,000	5169
Latvia	2050	All ages	Both	Neoplasms	DALYs per 100,000	8998
Morocco	2050	All ages	Both	Neoplasms	DALYs per 100,000	2481
Monaco	2050	All ages	Both	Neoplasms	DALYs per 100,000	12980
Republic of Moldova	2050	All ages	Both	Neoplasms	DALYs per 100,000	8016
Madagascar	2050	All ages	Both	Neoplasms	DALYs per 100,000	2056
Maldives	2050	All ages	Both	Neoplasms	DALYs per 100,000	1953
Mexico	2050	All ages	Both	Neoplasms	DALYs per 100,000	3762
Marshall Islands	2050	All ages	Both	Neoplasms	DALYs per 100,000	3838
North Macedonia	2050	All ages	Both	Neoplasms	DALYs per 100,000	9455
Mali	2050	All ages	Both	Neoplasms	DALYs per 100,000	1514
Malta	2050	All ages	Both	Neoplasms	DALYs per 100,000	5337
Myanmar	2050	All ages	Both	Neoplasms	DALYs per 100,000	3311
Montenegro	2050	All ages	Both	Neoplasms	DALYs per 100,000	8274
Mongolia	2050	All ages	Both	Neoplasms	DALYs per 100,000	6350
Northern Mariana Islands	2050	All ages	Both	Neoplasms	DALYs per 100,000	5849
Mozambique	2050	All ages	Both	Neoplasms	DALYs per 100,000	2305
Mauritania	2050	All ages	Both	Neoplasms	DALYs per 100,000	2062

Mauritius	2050	All ages	Both	Neoplasms	DALYs per 100,000	4908
Malawi	2050	All ages	Both	Neoplasms	DALYs per 100,000	2805
Malaysia	2050	All ages	Both	Neoplasms	DALYs per 100,000	4601
Namibia	2050	All ages	Both	Neoplasms	DALYs per 100,000	2950
Niger	2050	All ages	Both	Neoplasms	DALYs per 100,000	841
Nigeria	2050	All ages	Both	Neoplasms	DALYs per 100,000	1539
Nicaragua	2050	All ages	Both	Neoplasms	DALYs per 100,000	2674
Niue	2050	All ages	Both	Neoplasms	DALYs per 100,000	5083
Netherlands	2050	All ages	Both	Neoplasms	DALYs per 100,000	6544
Norway	2050	All ages	Both	Neoplasms	DALYs per 100,000	5616
Nepal	2050	All ages	Both	Neoplasms	DALYs per 100,000	2563
Nauru	2050	All ages	Both	Neoplasms	DALYs per 100,000	3726
New Zealand	2050	All ages	Both	Neoplasms	DALYs per 100,000	5313
Oman	2050	All ages	Both	Neoplasms	DALYs per 100,000	1816
Pakistan	2050	All ages	Both	Neoplasms	DALYs per 100,000	3561
Panama	2050	All ages	Both	Neoplasms	DALYs per 100,000	3495
Peru	2050	All ages	Both	Neoplasms	DALYs per 100,000	3821
Philippines	2050	All ages	Both	Neoplasms	DALYs per 100,000	3318
Palau	2050	All ages	Both	Neoplasms	DALYs per 100,000	8129
Papua New Guinea	2050	All ages	Both	Neoplasms	DALYs per 100,000	1689
Poland	2050	All ages	Both	Neoplasms	DALYs per 100,000	10002
Puerto Rico	2050	All ages	Both	Neoplasms	DALYs per 100,000	5926
Democratic People's Republic of Korea	2050	All ages	Both	Neoplasms	DALYs per 100,000	5590
Portugal	2050	All ages	Both	Neoplasms	DALYs per 100,000	6856

Paraguay	2050	All ages	Both	Neoplasms	DALYs per 100,000	4429
Palestine	2050	All ages	Both	Neoplasms	DALYs per 100,000	2920
Qatar	2050	All ages	Both	Neoplasms	DALYs per 100,000	3520
Romania	2050	All ages	Both	Neoplasms	DALYs per 100,000	8310
Russian Federation	2050	All ages	Both	Neoplasms	DALYs per 100,000	6309
Rwanda	2050	All ages	Both	Neoplasms	DALYs per 100,000	2675
Saudi Arabia	2050	All ages	Both	Neoplasms	DALYs per 100,000	3323
Senegal	2050	All ages	Both	Neoplasms	DALYs per 100,000	1991
Singapore	2050	All ages	Both	Neoplasms	DALYs per 100,000	4264
Solomon Islands	2050	All ages	Both	Neoplasms	DALYs per 100,000	2614
Sierra Leone	2050	All ages	Both	Neoplasms	DALYs per 100,000	1688
El Salvador	2050	All ages	Both	Neoplasms	DALYs per 100,000	4152
San Marino	2050	All ages	Both	Neoplasms	DALYs per 100,000	6665
Somalia	2050	All ages	Both	Neoplasms	DALYs per 100,000	1800
Serbia	2050	All ages	Both	Neoplasms	DALYs per 100,000	9863
South Sudan	2050	All ages	Both	Neoplasms	DALYs per 100,000	1968
Sao Tome and Principe	2050	All ages	Both	Neoplasms	DALYs per 100,000	2842
Suriname	2050	All ages	Both	Neoplasms	DALYs per 100,000	4329
Slovakia	2050	All ages	Both	Neoplasms	DALYs per 100,000	8360
Slovenia	2050	All ages	Both	Neoplasms	DALYs per 100,000	6893
Sweden	2050	All ages	Both	Neoplasms	DALYs per 100,000	4754
Eswatini	2050	All ages	Both	Neoplasms	DALYs per 100,000	4863
Seychelles	2050	All ages	Both	Neoplasms	DALYs per 100,000	5538
Syrian Arab Republic	2050	All ages	Both	Neoplasms	DALYs per 100,000	2336

Chad	2050	All ages	Both	Neoplasms	DALYs per 100,000	1128
Togo	2050	All ages	Both	Neoplasms	DALYs per 100,000	2113
Thailand	2050	All ages	Both	Neoplasms	DALYs per 100,000	6867
Tajikistan	2050	All ages	Both	Neoplasms	DALYs per 100,000	2016
Tokelau	2050	All ages	Both	Neoplasms	DALYs per 100,000	4510
Turkmenistan	2050	All ages	Both	Neoplasms	DALYs per 100,000	2881
Timor-Leste	2050	All ages	Both	Neoplasms	DALYs per 100,000	2185
Tonga	2050	All ages	Both	Neoplasms	DALYs per 100,000	3636
Trinidad and Tobago	2050	All ages	Both	Neoplasms	DALYs per 100,000	6190
Tunisia	2050	All ages	Both	Neoplasms	DALYs per 100,000	3750
Turkey	2050	All ages	Both	Neoplasms	DALYs per 100,000	5960
Tuvalu	2050	All ages	Both	Neoplasms	DALYs per 100,000	3233
Taiwan (Province of China)	2050	All ages	Both	Neoplasms	DALYs per 100,000	8632
United Republic of Tanzania	2050	All ages	Both	Neoplasms	DALYs per 100,000	2489
Uganda	2050	All ages	Both	Neoplasms	DALYs per 100,000	2823
Ukraine	2050	All ages	Both	Neoplasms	DALYs per 100,000	6848
Uruguay	2050	All ages	Both	Neoplasms	DALYs per 100,000	8475
United States of America	2050	All ages	Both	Neoplasms	DALYs per 100,000	5727
Uzbekistan	2050	All ages	Both	Neoplasms	DALYs per 100,000	2368
Saint Vincent and the Grenadines	2050	All ages	Both	Neoplasms	DALYs per 100,000	7033
Venezuela (Bolivarian Republic of)	2050	All ages	Both	Neoplasms	DALYs per 100,000	4596
United States Virgin Islands	2050	All ages	Both	Neoplasms	DALYs per 100,000	6577
Viet Nam	2050	All ages	Both	Neoplasms	DALYs per 100,000	5271
Vanuatu	2050	All ages	Both	Neoplasms	DALYs per 100,000	2164

Samoa	2050	All ages	Both	Neoplasms	DALYs per 100,000	2070
Yemen	2050	All ages	Both	Neoplasms	DALYs per 100,000	1867
South Africa	2050	All ages	Both	Neoplasms	DALYs per 100,000	4596
Zambia	2050	All ages	Both	Neoplasms	DALYs per 100,000	3275
Zimbabwe	2050	All ages	Both	Neoplasms	DALYs per 100,000	4000
Egypt	2050	All ages	Both	Neoplasms	DALYs per 100,000	2801
Sudan	2050	All ages	Both	Neoplasms	DALYs per 100,000	1968
China	2050	All ages	Both	Neoplasms	DALYs per 100,000	7560

S6C: country-based burden of chronic respiratory diseases mortality projection 2050

Country	Year	Measure	Rate
Afghanistan	2050	Deaths per 100,000	14.8
Angola	2050	Deaths per 100,000	11.7
Albania	2050	Deaths per 100,000	66.9
Andorra	2050	Deaths per 100,000	126.1
United Arab Emirates	2050	Deaths per 100,000	58.1
Argentina	2050	Deaths per 100,000	66.1
Armenia	2050	Deaths per 100,000	63.1
American Samoa	2050	Deaths per 100,000	73.5
Antigua and Barbuda	2050	Deaths per 100,000	25.4
Australia	2050	Deaths per 100,000	64.6
Austria	2050	Deaths per 100,000	58.7
Azerbaijan	2050	Deaths per 100,000	38.9
Burundi	2050	Deaths per 100,000	15.4

Belgium	2050	Deaths per 100,000	73.1
Benin	2050	Deaths per 100,000	9.2
Burkina Faso	2050	Deaths per 100,000	6.9
Bangladesh	2050	Deaths per 100,000	86.1
Bulgaria	2050	Deaths per 100,000	46
Bahrain	2050	Deaths per 100,000	62.6
Bahamas	2050	Deaths per 100,000	32.1
Bosnia and Herzegovina	2050	Deaths per 100,000	72.5
Belarus	2050	Deaths per 100,000	25.1
Belize	2050	Deaths per 100,000	32.2
Bermuda	2050	Deaths per 100,000	70.7
Bolivia (Plurinational State of)	2050	Deaths per 100,000	43.9
Brazil	2050	Deaths per 100,000	59.8
Barbados	2050	Deaths per 100,000	33
Brunei Darussalam	2050	Deaths per 100,000	96.6
Bhutan	2050	Deaths per 100,000	121.6
Botswana	2050	Deaths per 100,000	46.6
Central African Republic	2050	Deaths per 100,000	25.9
Canada	2050	Deaths per 100,000	78.9
Switzerland	2050	Deaths per 100,000	57.7
Chile	2050	Deaths per 100,000	86.3
CÃ´te d'Ivoire	2050	Deaths per 100,000	13.3
Cameroon	2050	Deaths per 100,000	14
Democratic Republic of the Congo	2050	Deaths per 100,000	23.7

Congo	2050	Deaths per 100,000	30.7
Cook Islands	2050	Deaths per 100,000	78
Colombia	2050	Deaths per 100,000	92.7
Comoros	2050	Deaths per 100,000	28.6
Cabo Verde	2050	Deaths per 100,000	28.5
Costa Rica	2050	Deaths per 100,000	74.9
Cuba	2050	Deaths per 100,000	80
Cyprus	2050	Deaths per 100,000	93.3
Czechia	2050	Deaths per 100,000	61.7
Germany	2050	Deaths per 100,000	71.6
Djibouti	2050	Deaths per 100,000	19.7
Dominica	2050	Deaths per 100,000	48.9
Denmark	2050	Deaths per 100,000	115.2
Dominican Republic	2050	Deaths per 100,000	23.8
Algeria	2050	Deaths per 100,000	36.9
Ecuador	2050	Deaths per 100,000	48
Eritrea	2050	Deaths per 100,000	21.9
Spain	2050	Deaths per 100,000	123.2
Estonia	2050	Deaths per 100,000	23.1
Ethiopia	2050	Deaths per 100,000	10.1
Finland	2050	Deaths per 100,000	55.1
Fiji	2050	Deaths per 100,000	67.6
France	2050	Deaths per 100,000	52.1
Micronesia (Federated States of)	2050	Deaths per 100,000	79.5

Gabon	2050	Deaths per 100,000	23.2
United Kingdom	2050	Deaths per 100,000	104.3
Georgia	2050	Deaths per 100,000	30.3
Ghana	2050	Deaths per 100,000	12.9
Guinea	2050	Deaths per 100,000	12
Gambia	2050	Deaths per 100,000	15.7
Guinea-Bissau	2050	Deaths per 100,000	16.3
Equatorial Guinea	2050	Deaths per 100,000	14.9
Greece	2050	Deaths per 100,000	86.9
Grenada	2050	Deaths per 100,000	36
Greenland	2050	Deaths per 100,000	109.8
Guatemala	2050	Deaths per 100,000	23.5
Guam	2050	Deaths per 100,000	55.1
Guyana	2050	Deaths per 100,000	26.9
Honduras	2050	Deaths per 100,000	46.7
Croatia	2050	Deaths per 100,000	94.7
Haiti	2050	Deaths per 100,000	26.8
Hungary	2050	Deaths per 100,000	78.8
Indonesia	2050	Deaths per 100,000	83.3
India	2050	Deaths per 100,000	145.4
Ireland	2050	Deaths per 100,000	86.7
Iran (Islamic Republic of)	2050	Deaths per 100,000	46.3
Iraq	2050	Deaths per 100,000	13.6
Iceland	2050	Deaths per 100,000	55.3

Israel	2050	Deaths per 100,000	28.7
Italy	2050	Deaths per 100,000	102.7
Jamaica	2050	Deaths per 100,000	36.8
Jordan	2050	Deaths per 100,000	18
Japan	2050	Deaths per 100,000	70.3
Kazakhstan	2050	Deaths per 100,000	76.2
Kenya	2050	Deaths per 100,000	24.6
Kyrgyzstan	2050	Deaths per 100,000	45.5
Cambodia	2050	Deaths per 100,000	42.1
Kiribati	2050	Deaths per 100,000	69.5
Saint Kitts and Nevis	2050	Deaths per 100,000	49.5
Republic of Korea	2050	Deaths per 100,000	94
Kuwait	2050	Deaths per 100,000	12.5
Lao People's Democratic Republic	2050	Deaths per 100,000	55.9
Lebanon	2050	Deaths per 100,000	52.9
Liberia	2050	Deaths per 100,000	17.1
Libya	2050	Deaths per 100,000	53.8
Saint Lucia	2050	Deaths per 100,000	68
Sri Lanka	2050	Deaths per 100,000	156
Lesotho	2050	Deaths per 100,000	49.4
Lithuania	2050	Deaths per 100,000	42.5
Luxembourg	2050	Deaths per 100,000	60.3
Latvia	2050	Deaths per 100,000	28.2
Morocco	2050	Deaths per 100,000	40.8

Monaco	2050	Deaths per 100,000	81.4
Republic of Moldova	2050	Deaths per 100,000	52.1
Madagascar	2050	Deaths per 100,000	28.8
Maldives	2050	Deaths per 100,000	67
Mexico	2050	Deaths per 100,000	59.4
Marshall Islands	2050	Deaths per 100,000	63.5
North Macedonia	2050	Deaths per 100,000	63.6
Mali	2050	Deaths per 100,000	16.1
Malta	2050	Deaths per 100,000	51.2
Myanmar	2050	Deaths per 100,000	125.2
Montenegro	2050	Deaths per 100,000	15.4
Mongolia	2050	Deaths per 100,000	23.7
Northern Mariana Islands	2050	Deaths per 100,000	113.1
Mozambique	2050	Deaths per 100,000	10.1
Mauritania	2050	Deaths per 100,000	12.9
Mauritius	2050	Deaths per 100,000	99.6
Malawi	2050	Deaths per 100,000	14.7
Malaysia	2050	Deaths per 100,000	44.9
Namibia	2050	Deaths per 100,000	42.4
Niger	2050	Deaths per 100,000	8.2
Nigeria	2050	Deaths per 100,000	10.5
Nicaragua	2050	Deaths per 100,000	39.4
Niue	2050	Deaths per 100,000	99.7
Netherlands	2050	Deaths per 100,000	103.1

Norway	2050	Deaths per 100,000	86.3
Nepal	2050	Deaths per 100,000	143.1
Nauru	2050	Deaths per 100,000	44.9
New Zealand	2050	Deaths per 100,000	73.8
Oman	2050	Deaths per 100,000	24.9
Pakistan	2050	Deaths per 100,000	50.7
Panama	2050	Deaths per 100,000	42.3
Peru	2050	Deaths per 100,000	53.8
Philippines	2050	Deaths per 100,000	38.7
Palau	2050	Deaths per 100,000	181.9
Papua New Guinea	2050	Deaths per 100,000	74.9
Poland	2050	Deaths per 100,000	42.7
Puerto Rico	2050	Deaths per 100,000	139.3
Democratic People's Republic of Korea	2050	Deaths per 100,000	270
Portugal	2050	Deaths per 100,000	99.7
Paraguay	2050	Deaths per 100,000	31.2
Palestine	2050	Deaths per 100,000	19.7
Qatar	2050	Deaths per 100,000	18.3
Romania	2050	Deaths per 100,000	57.9
Russian Federation	2050	Deaths per 100,000	39.6
Rwanda	2050	Deaths per 100,000	19.1
Saudi Arabia	2050	Deaths per 100,000	57.2
Senegal	2050	Deaths per 100,000	17
Singapore	2050	Deaths per 100,000	32.1

Solomon Islands	2050	Deaths per 100,000	36.5
Sierra Leone	2050	Deaths per 100,000	13.5
El Salvador	2050	Deaths per 100,000	49.9
San Marino	2050	Deaths per 100,000	58.7
Somalia	2050	Deaths per 100,000	15.2
Serbia	2050	Deaths per 100,000	63.9
South Sudan	2050	Deaths per 100,000	11.6
Sao Tome and Principe	2050	Deaths per 100,000	66.1
Suriname	2050	Deaths per 100,000	34.4
Slovakia	2050	Deaths per 100,000	35.4
Slovenia	2050	Deaths per 100,000	58.3
Sweden	2050	Deaths per 100,000	51.3
Eswatini	2050	Deaths per 100,000	40.1
Seychelles	2050	Deaths per 100,000	48.8
Syrian Arab Republic	2050	Deaths per 100,000	39.5
Chad	2050	Deaths per 100,000	7.3
Togo	2050	Deaths per 100,000	18.2
Thailand	2050	Deaths per 100,000	92.6
Tajikistan	2050	Deaths per 100,000	31.7
Tokelau	2050	Deaths per 100,000	81.6
Turkmenistan	2050	Deaths per 100,000	14.4
Timor-Leste	2050	Deaths per 100,000	34.2
Tonga	2050	Deaths per 100,000	46.4
Trinidad and Tobago	2050	Deaths per 100,000	49

Tunisia	2050	Deaths per 100,000	50
Turkey	2050	Deaths per 100,000	102.5
Tuvalu	2050	Deaths per 100,000	54.8
Taiwan (Province of China)	2050	Deaths per 100,000	97.6
United Republic of Tanzania	2050	Deaths per 100,000	11.7
Uganda	2050	Deaths per 100,000	10.7
Ukraine	2050	Deaths per 100,000	35.7
Uruguay	2050	Deaths per 100,000	95.2
United States of America	2050	Deaths per 100,000	108.1
Uzbekistan	2050	Deaths per 100,000	15.9
Saint Vincent and the Grenadines	2050	Deaths per 100,000	29.8
Venezuela (Bolivarian Republic of)	2050	Deaths per 100,000	54.9
United States Virgin Islands	2050	Deaths per 100,000	62.9
Viet Nam	2050	Deaths per 100,000	76.7
Vanuatu	2050	Deaths per 100,000	50.2
Samoa	2050	Deaths per 100,000	51
Yemen	2050	Deaths per 100,000	19.5
South Africa	2050	Deaths per 100,000	52.9
Zambia	2050	Deaths per 100,000	13.4
Zimbabwe	2050	Deaths per 100,000	20.7
Egypt	2050	Deaths per 100,000	18.2
Sudan	2050	Deaths per 100,000	18.8
China	2050	Deaths per 100,000	228.4

Country	Year	Age	Sex	Cause of death or injury	Measure	Rate

Afghanistan	2050	All ages	Both	Chronic respiratory diseases	DALYs per 100,000	572
Angola	2050	All ages	Both	Chronic respiratory diseases	DALYs per 100,000	451
Albania	2050	All ages	Both	Chronic respiratory diseases	DALYs per 100,000	1296
Andorra	2050	All ages	Both	Chronic respiratory diseases	DALYs per 100,000	2132
United Arab Emirates	2050	All ages	Both	Chronic respiratory diseases	DALYs per 100,000	1514
Argentina	2050	All ages	Both	Chronic respiratory diseases	DALYs per 100,000	1296
Armenia	2050	All ages	Both	Chronic respiratory diseases	DALYs per 100,000	1105
American Samoa	2050	All ages	Both	Chronic respiratory diseases	DALYs per 100,000	1560
Antigua and Barbuda	2050	All ages	Both	Chronic respiratory diseases	DALYs per 100,000	611
Australia	2050	All ages	Both	Chronic respiratory diseases	DALYs per 100,000	1111
Austria	2050	All ages	Both	Chronic respiratory diseases	DALYs per 100,000	1142
Azerbaijan	2050	All ages	Both	Chronic respiratory diseases	DALYs per 100,000	907
Burundi	2050	All ages	Both	Chronic respiratory diseases	DALYs per 100,000	649
Belgium	2050	All ages	Both	Chronic respiratory diseases	DALYs per 100,000	1298
Benin	2050	All ages	Both	Chronic respiratory diseases	DALYs per 100,000	416
Burkina Faso	2050	All ages	Both	Chronic respiratory diseases	DALYs per 100,000	381
Bangladesh	2050	All ages	Both	Chronic respiratory diseases	DALYs per 100,000	2028
Bulgaria	2050	All ages	Both	Chronic respiratory diseases	DALYs per 100,000	1068
Bahrain	2050	All ages	Both	Chronic respiratory diseases	DALYs per 100,000	1362
Bahamas	2050	All ages	Both	Chronic respiratory diseases	DALYs per 100,000	742
Bosnia and Herzegovina	2050	All ages	Both	Chronic respiratory diseases	DALYs per 100,000	1536
Belarus	2050	All ages	Both	Chronic respiratory diseases	DALYs per 100,000	738
Belize	2050	All ages	Both	Chronic respiratory diseases	DALYs per 100,000	834
Bermuda	2050	All ages	Both	Chronic respiratory diseases	DALYs per 100,000	1162

Bolivia (Plurinational State of)	2050	All ages	Both	Chronic respiratory diseases	DALYs per 100,000	902
Brazil	2050	All ages	Both	Chronic respiratory diseases	DALYs per 100,000	1147
Barbados	2050	All ages	Both	Chronic respiratory diseases	DALYs per 100,000	749
Brunei Darussalam	2050	All ages	Both	Chronic respiratory diseases	DALYs per 100,000	1878
Bhutan	2050	All ages	Both	Chronic respiratory diseases	DALYs per 100,000	2496
Botswana	2050	All ages	Both	Chronic respiratory diseases	DALYs per 100,000	1274
Central African Republic	2050	All ages	Both	Chronic respiratory diseases	DALYs per 100,000	924
Canada	2050	All ages	Both	Chronic respiratory diseases	DALYs per 100,000	1319
Switzerland	2050	All ages	Both	Chronic respiratory diseases	DALYs per 100,000	1096
Chile	2050	All ages	Both	Chronic respiratory diseases	DALYs per 100,000	1360
CÃ´te d'Ivoire	2050	All ages	Both	Chronic respiratory diseases	DALYs per 100,000	536
Cameroon	2050	All ages	Both	Chronic respiratory diseases	DALYs per 100,000	499
Democratic Republic of the Congo	2050	All ages	Both	Chronic respiratory diseases	DALYs per 100,000	749
Congo	2050	All ages	Both	Chronic respiratory diseases	DALYs per 100,000	933
Cook Islands	2050	All ages	Both	Chronic respiratory diseases	DALYs per 100,000	1371
Colombia	2050	All ages	Both	Chronic respiratory diseases	DALYs per 100,000	1430
Comoros	2050	All ages	Both	Chronic respiratory diseases	DALYs per 100,000	846
Cabo Verde	2050	All ages	Both	Chronic respiratory diseases	DALYs per 100,000	755
Costa Rica	2050	All ages	Both	Chronic respiratory diseases	DALYs per 100,000	1267
Cuba	2050	All ages	Both	Chronic respiratory diseases	DALYs per 100,000	1475
Cyprus	2050	All ages	Both	Chronic respiratory diseases	DALYs per 100,000	1565
Czechia	2050	All ages	Both	Chronic respiratory diseases	DALYs per 100,000	1251
Germany	2050	All ages	Both	Chronic respiratory diseases	DALYs per 100,000	1358
Djibouti	2050	All ages	Both	Chronic respiratory diseases	DALYs per 100,000	683

Dominica	2050	All ages	Both	Chronic respiratory diseases	DALYs per 100,000	1043
Denmark	2050	All ages	Both	Chronic respiratory diseases	DALYs per 100,000	1811
Dominican Republic	2050	All ages	Both	Chronic respiratory diseases	DALYs per 100,000	555
Algeria	2050	All ages	Both	Chronic respiratory diseases	DALYs per 100,000	912
Ecuador	2050	All ages	Both	Chronic respiratory diseases	DALYs per 100,000	813
Eritrea	2050	All ages	Both	Chronic respiratory diseases	DALYs per 100,000	762
Spain	2050	All ages	Both	Chronic respiratory diseases	DALYs per 100,000	1780
Estonia	2050	All ages	Both	Chronic respiratory diseases	DALYs per 100,000	518
Ethiopia	2050	All ages	Both	Chronic respiratory diseases	DALYs per 100,000	410
Finland	2050	All ages	Both	Chronic respiratory diseases	DALYs per 100,000	1111
Fiji	2050	All ages	Both	Chronic respiratory diseases	DALYs per 100,000	1479
France	2050	All ages	Both	Chronic respiratory diseases	DALYs per 100,000	945
Micronesia (Federated States of)	2050	All ages	Both	Chronic respiratory diseases	DALYs per 100,000	1877
Gabon	2050	All ages	Both	Chronic respiratory diseases	DALYs per 100,000	714
United Kingdom	2050	All ages	Both	Chronic respiratory diseases	DALYs per 100,000	1894
Georgia	2050	All ages	Both	Chronic respiratory diseases	DALYs per 100,000	712
Ghana	2050	All ages	Both	Chronic respiratory diseases	DALYs per 100,000	495
Guinea	2050	All ages	Both	Chronic respiratory diseases	DALYs per 100,000	517
Gambia	2050	All ages	Both	Chronic respiratory diseases	DALYs per 100,000	571
Guinea-Bissau	2050	All ages	Both	Chronic respiratory diseases	DALYs per 100,000	676
Equatorial Guinea	2050	All ages	Both	Chronic respiratory diseases	DALYs per 100,000	534
Greece	2050	All ages	Both	Chronic respiratory diseases	DALYs per 100,000	1643
Grenada	2050	All ages	Both	Chronic respiratory diseases	DALYs per 100,000	870
Greenland	2050	All ages	Both	Chronic respiratory diseases	DALYs per 100,000	2046

Guatemala	2050	All ages	Both	Chronic respiratory diseases	DALYs per 100,000	530
Guam	2050	All ages	Both	Chronic respiratory diseases	DALYs per 100,000	1204
Guyana	2050	All ages	Both	Chronic respiratory diseases	DALYs per 100,000	735
Honduras	2050	All ages	Both	Chronic respiratory diseases	DALYs per 100,000	1062
Croatia	2050	All ages	Both	Chronic respiratory diseases	DALYs per 100,000	1654
Haiti	2050	All ages	Both	Chronic respiratory diseases	DALYs per 100,000	955
Hungary	2050	All ages	Both	Chronic respiratory diseases	DALYs per 100,000	1630
Indonesia	2050	All ages	Both	Chronic respiratory diseases	DALYs per 100,000	1798
India	2050	All ages	Both	Chronic respiratory diseases	DALYs per 100,000	3025
Ireland	2050	All ages	Both	Chronic respiratory diseases	DALYs per 100,000	1412
Iran (Islamic Republic of)	2050	All ages	Both	Chronic respiratory diseases	DALYs per 100,000	1046
Iraq	2050	All ages	Both	Chronic respiratory diseases	DALYs per 100,000	454
Iceland	2050	All ages	Both	Chronic respiratory diseases	DALYs per 100,000	1059
Israel	2050	All ages	Both	Chronic respiratory diseases	DALYs per 100,000	603
Italy	2050	All ages	Both	Chronic respiratory diseases	DALYs per 100,000	1576
Jamaica	2050	All ages	Both	Chronic respiratory diseases	DALYs per 100,000	842
Jordan	2050	All ages	Both	Chronic respiratory diseases	DALYs per 100,000	542
Japan	2050	All ages	Both	Chronic respiratory diseases	DALYs per 100,000	1223
Kazakhstan	2050	All ages	Both	Chronic respiratory diseases	DALYs per 100,000	1556
Kenya	2050	All ages	Both	Chronic respiratory diseases	DALYs per 100,000	704
Kyrgyzstan	2050	All ages	Both	Chronic respiratory diseases	DALYs per 100,000	985
Cambodia	2050	All ages	Both	Chronic respiratory diseases	DALYs per 100,000	1019
Kiribati	2050	All ages	Both	Chronic respiratory diseases	DALYs per 100,000	1834
Saint Kitts and Nevis	2050	All ages	Both	Chronic respiratory diseases	DALYs per 100,000	997

Republic of Korea	2050	All ages	Both	Chronic respiratory diseases	DALYs per 100,000	1573
Kuwait	2050	All ages	Both	Chronic respiratory diseases	DALYs per 100,000	506
Lao People's Democratic Republic	2050	All ages	Both	Chronic respiratory diseases	DALYs per 100,000	1360
Lebanon	2050	All ages	Both	Chronic respiratory diseases	DALYs per 100,000	1241
Liberia	2050	All ages	Both	Chronic respiratory diseases	DALYs per 100,000	600
Libya	2050	All ages	Both	Chronic respiratory diseases	DALYs per 100,000	1355
Saint Lucia	2050	All ages	Both	Chronic respiratory diseases	DALYs per 100,000	1334
Sri Lanka	2050	All ages	Both	Chronic respiratory diseases	DALYs per 100,000	2558
Lesotho	2050	All ages	Both	Chronic respiratory diseases	DALYs per 100,000	1480
Lithuania	2050	All ages	Both	Chronic respiratory diseases	DALYs per 100,000	933
Luxembourg	2050	All ages	Both	Chronic respiratory diseases	DALYs per 100,000	1133
Latvia	2050	All ages	Both	Chronic respiratory diseases	DALYs per 100,000	720
Morocco	2050	All ages	Both	Chronic respiratory diseases	DALYs per 100,000	1021
Monaco	2050	All ages	Both	Chronic respiratory diseases	DALYs per 100,000	1462
Republic of Moldova	2050	All ages	Both	Chronic respiratory diseases	DALYs per 100,000	1159
Madagascar	2050	All ages	Both	Chronic respiratory diseases	DALYs per 100,000	994
Maldives	2050	All ages	Both	Chronic respiratory diseases	DALYs per 100,000	1449
Mexico	2050	All ages	Both	Chronic respiratory diseases	DALYs per 100,000	1082
Marshall Islands	2050	All ages	Both	Chronic respiratory diseases	DALYs per 100,000	1681
North Macedonia	2050	All ages	Both	Chronic respiratory diseases	DALYs per 100,000	1450
Mali	2050	All ages	Both	Chronic respiratory diseases	DALYs per 100,000	609
Malta	2050	All ages	Both	Chronic respiratory diseases	DALYs per 100,000	1042
Myanmar	2050	All ages	Both	Chronic respiratory diseases	DALYs per 100,000	2421
Montenegro	2050	All ages	Both	Chronic respiratory diseases	DALYs per 100,000	501

Mongolia	2050	All ages	Both	Chronic respiratory diseases	DALYs per 100,000	581
Northern Mariana Islands	2050	All ages	Both	Chronic respiratory diseases	DALYs per 100,000	2085
Mozambique	2050	All ages	Both	Chronic respiratory diseases	DALYs per 100,000	471
Mauritania	2050	All ages	Both	Chronic respiratory diseases	DALYs per 100,000	549
Mauritius	2050	All ages	Both	Chronic respiratory diseases	DALYs per 100,000	1765
Malawi	2050	All ages	Both	Chronic respiratory diseases	DALYs per 100,000	583
Malaysia	2050	All ages	Both	Chronic respiratory diseases	DALYs per 100,000	1040
Namibia	2050	All ages	Both	Chronic respiratory diseases	DALYs per 100,000	1162
Niger	2050	All ages	Both	Chronic respiratory diseases	DALYs per 100,000	417
Nigeria	2050	All ages	Both	Chronic respiratory diseases	DALYs per 100,000	433
Nicaragua	2050	All ages	Both	Chronic respiratory diseases	DALYs per 100,000	784
Niue	2050	All ages	Both	Chronic respiratory diseases	DALYs per 100,000	1995
Netherlands	2050	All ages	Both	Chronic respiratory diseases	DALYs per 100,000	1688
Norway	2050	All ages	Both	Chronic respiratory diseases	DALYs per 100,000	1442
Nepal	2050	All ages	Both	Chronic respiratory diseases	DALYs per 100,000	2933
Nauru	2050	All ages	Both	Chronic respiratory diseases	DALYs per 100,000	1316
New Zealand	2050	All ages	Both	Chronic respiratory diseases	DALYs per 100,000	1260
Oman	2050	All ages	Both	Chronic respiratory diseases	DALYs per 100,000	786
Pakistan	2050	All ages	Both	Chronic respiratory diseases	DALYs per 100,000	1261
Panama	2050	All ages	Both	Chronic respiratory diseases	DALYs per 100,000	798
Peru	2050	All ages	Both	Chronic respiratory diseases	DALYs per 100,000	962
Philippines	2050	All ages	Both	Chronic respiratory diseases	DALYs per 100,000	1076
Palau	2050	All ages	Both	Chronic respiratory diseases	DALYs per 100,000	3316
Papua New Guinea	2050	All ages	Both	Chronic respiratory diseases	DALYs per 100,000	1839

Poland	2050	All ages	Both	Chronic respiratory diseases	DALYs per 100,000	1100
Puerto Rico	2050	All ages	Both	Chronic respiratory diseases	DALYs per 100,000	2007
Democratic People's Republic of Korea	2050	All ages	Both	Chronic respiratory diseases	DALYs per 100,000	4565
Portugal	2050	All ages	Both	Chronic respiratory diseases	DALYs per 100,000	1626
Paraguay	2050	All ages	Both	Chronic respiratory diseases	DALYs per 100,000	730
Palestine	2050	All ages	Both	Chronic respiratory diseases	DALYs per 100,000	592
Qatar	2050	All ages	Both	Chronic respiratory diseases	DALYs per 100,000	559
Romania	2050	All ages	Both	Chronic respiratory diseases	DALYs per 100,000	1292
Russian Federation	2050	All ages	Both	Chronic respiratory diseases	DALYs per 100,000	883
Rwanda	2050	All ages	Both	Chronic respiratory diseases	DALYs per 100,000	822
Saudi Arabia	2050	All ages	Both	Chronic respiratory diseases	DALYs per 100,000	1486
Senegal	2050	All ages	Both	Chronic respiratory diseases	DALYs per 100,000	576
Singapore	2050	All ages	Both	Chronic respiratory diseases	DALYs per 100,000	631
Solomon Islands	2050	All ages	Both	Chronic respiratory diseases	DALYs per 100,000	1043
Sierra Leone	2050	All ages	Both	Chronic respiratory diseases	DALYs per 100,000	525
El Salvador	2050	All ages	Both	Chronic respiratory diseases	DALYs per 100,000	1020
San Marino	2050	All ages	Both	Chronic respiratory diseases	DALYs per 100,000	1162
Somalia	2050	All ages	Both	Chronic respiratory diseases	DALYs per 100,000	718
Serbia	2050	All ages	Both	Chronic respiratory diseases	DALYs per 100,000	1403
South Sudan	2050	All ages	Both	Chronic respiratory diseases	DALYs per 100,000	527
Sao Tome and Principe	2050	All ages	Both	Chronic respiratory diseases	DALYs per 100,000	1696
Suriname	2050	All ages	Both	Chronic respiratory diseases	DALYs per 100,000	788
Slovakia	2050	All ages	Both	Chronic respiratory diseases	DALYs per 100,000	837
Slovenia	2050	All ages	Both	Chronic respiratory diseases	DALYs per 100,000	1091

Sweden	2050	All ages	Both	Chronic respiratory diseases	DALYs per 100,000	1077
Eswatini	2050	All ages	Both	Chronic respiratory diseases	DALYs per 100,000	1273
Seychelles	2050	All ages	Both	Chronic respiratory diseases	DALYs per 100,000	1067
Syrian Arab Republic	2050	All ages	Both	Chronic respiratory diseases	DALYs per 100,000	989
Chad	2050	All ages	Both	Chronic respiratory diseases	DALYs per 100,000	366
Togo	2050	All ages	Both	Chronic respiratory diseases	DALYs per 100,000	648
Thailand	2050	All ages	Both	Chronic respiratory diseases	DALYs per 100,000	1677
Tajikistan	2050	All ages	Both	Chronic respiratory diseases	DALYs per 100,000	748
Tokelau	2050	All ages	Both	Chronic respiratory diseases	DALYs per 100,000	1713
Turkmenistan	2050	All ages	Both	Chronic respiratory diseases	DALYs per 100,000	416
Timor-Leste	2050	All ages	Both	Chronic respiratory diseases	DALYs per 100,000	902
Tonga	2050	All ages	Both	Chronic respiratory diseases	DALYs per 100,000	1042
Trinidad and Tobago	2050	All ages	Both	Chronic respiratory diseases	DALYs per 100,000	959
Tunisia	2050	All ages	Both	Chronic respiratory diseases	DALYs per 100,000	1200
Turkey	2050	All ages	Both	Chronic respiratory diseases	DALYs per 100,000	2042
Tuvalu	2050	All ages	Both	Chronic respiratory diseases	DALYs per 100,000	1352
Taiwan (Province of China)	2050	All ages	Both	Chronic respiratory diseases	DALYs per 100,000	1607
United Republic of Tanzania	2050	All ages	Both	Chronic respiratory diseases	DALYs per 100,000	541
Uganda	2050	All ages	Both	Chronic respiratory diseases	DALYs per 100,000	479
Ukraine	2050	All ages	Both	Chronic respiratory diseases	DALYs per 100,000	876
Uruguay	2050	All ages	Both	Chronic respiratory diseases	DALYs per 100,000	1632
United States of America	2050	All ages	Both	Chronic respiratory diseases	DALYs per 100,000	2196
Uzbekistan	2050	All ages	Both	Chronic respiratory diseases	DALYs per 100,000	493
Saint Vincent and the Grenadines	2050	All ages	Both	Chronic respiratory diseases	DALYs per 100,000	694

Venezuela (Bolivarian Republic of)	2050	All ages	Both	Chronic respiratory diseases	DALYs per 100,000	1073
United States Virgin Islands	2050	All ages	Both	Chronic respiratory diseases	DALYs per 100,000	1064
Viet Nam	2050	All ages	Both	Chronic respiratory diseases	DALYs per 100,000	1553
Vanuatu	2050	All ages	Both	Chronic respiratory diseases	DALYs per 100,000	1352
Samoa	2050	All ages	Both	Chronic respiratory diseases	DALYs per 100,000	1212
Yemen	2050	All ages	Both	Chronic respiratory diseases	DALYs per 100,000	657
South Africa	2050	All ages	Both	Chronic respiratory diseases	DALYs per 100,000	1344
Zambia	2050	All ages	Both	Chronic respiratory diseases	DALYs per 100,000	487
Zimbabwe	2050	All ages	Both	Chronic respiratory diseases	DALYs per 100,000	701
Egypt	2050	All ages	Both	Chronic respiratory diseases	DALYs per 100,000	609
Sudan	2050	All ages	Both	Chronic respiratory diseases	DALYs per 100,000	606
China	2050	All ages	Both	Chronic respiratory diseases	DALYs per 100,000	3504

S5D: country-based burden of diabetes maleates mortality projection 2050

Location	Year	Age	Sex	Cause of death or injury	Measure	Value
Afghanistan	2050	All ages	Both	Diabetes mellitus	Deaths per 100,000	12.72961
Angola	2050	All ages	Both	Diabetes mellitus	Deaths per 100,000	16.61229
Albania	2050	All ages	Both	Diabetes mellitus	Deaths per 100,000	13.17565
Andorra	2050	All ages	Both	Diabetes mellitus	Deaths per 100,000	41.24796
United Arab Emirates	2050	All ages	Both	Diabetes mellitus	Deaths per 100,000	54.31395
Argentina	2050	All ages	Both	Diabetes mellitus	Deaths per 100,000	27.26651
Armenia	2050	All ages	Both	Diabetes mellitus	Deaths per 100,000	28.79634
American Samoa	2050	All ages	Both	Diabetes mellitus	Deaths per 100,000	102.6335
Antigua and Barbuda	2050	All ages	Both	Diabetes mellitus	Deaths per 100,000	108.1668

Australia	2050	All ages	Both	Diabetes mellitus	Deaths per 100,000	22.18551
Austria	2050	All ages	Both	Diabetes mellitus	Deaths per 100,000	27.98342
Azerbaijan	2050	All ages	Both	Diabetes mellitus	Deaths per 100,000	26.61012
Burundi	2050	All ages	Both	Diabetes mellitus	Deaths per 100,000	18.04669
Belgium	2050	All ages	Both	Diabetes mellitus	Deaths per 100,000	13.27643
Benin	2050	All ages	Both	Diabetes mellitus	Deaths per 100,000	13.47169
Burkina Faso	2050	All ages	Both	Diabetes mellitus	Deaths per 100,000	13.07714
Bangladesh	2050	All ages	Both	Diabetes mellitus	Deaths per 100,000	52.64123
Bulgaria	2050	All ages	Both	Diabetes mellitus	Deaths per 100,000	35.12636
Bahrain	2050	All ages	Both	Diabetes mellitus	Deaths per 100,000	163.6607
Bahamas	2050	All ages	Both	Diabetes mellitus	Deaths per 100,000	55.42578
Bosnia and Herzegovina	2050	All ages	Both	Diabetes mellitus	Deaths per 100,000	94.64235
Belarus	2050	All ages	Both	Diabetes mellitus	Deaths per 100,000	8.937571
Belize	2050	All ages	Both	Diabetes mellitus	Deaths per 100,000	53.60679
Bermuda	2050	All ages	Both	Diabetes mellitus	Deaths per 100,000	60.84353
Bolivia (Plurinational State of)	2050	All ages	Both	Diabetes mellitus	Deaths per 100,000	40.02805
Brazil	2050	All ages	Both	Diabetes mellitus	Deaths per 100,000	47.52023
Barbados	2050	All ages	Both	Diabetes mellitus	Deaths per 100,000	148.1395
Brunei Darussalam	2050	All ages	Both	Diabetes mellitus	Deaths per 100,000	69.21147
Bhutan	2050	All ages	Both	Diabetes mellitus	Deaths per 100,000	38.87551
Botswana	2050	All ages	Both	Diabetes mellitus	Deaths per 100,000	52.00871
Central African Republic	2050	All ages	Both	Diabetes mellitus	Deaths per 100,000	34.07679
Canada	2050	All ages	Both	Diabetes mellitus	Deaths per 100,000	22.86286
Switzerland	2050	All ages	Both	Diabetes mellitus	Deaths per 100,000	17.88052

Chile	2050	All ages	Both	Diabetes mellitus	Deaths per 100,000	26.12077
CÃ´te d'Ivoire	2050	All ages	Both	Diabetes mellitus	Deaths per 100,000	21.49194
Cameroon	2050	All ages	Both	Diabetes mellitus	Deaths per 100,000	27.34346
Democratic Republic of the Congo	2050	All ages	Both	Diabetes mellitus	Deaths per 100,000	24.04992
Congo	2050	All ages	Both	Diabetes mellitus	Deaths per 100,000	38.91625
Cook Islands	2050	All ages	Both	Diabetes mellitus	Deaths per 100,000	193.0721
Colombia	2050	All ages	Both	Diabetes mellitus	Deaths per 100,000	23.63571
Comoros	2050	All ages	Both	Diabetes mellitus	Deaths per 100,000	41.30883
Cabo Verde	2050	All ages	Both	Diabetes mellitus	Deaths per 100,000	46.19211
Costa Rica	2050	All ages	Both	Diabetes mellitus	Deaths per 100,000	37.83346
Cuba	2050	All ages	Both	Diabetes mellitus	Deaths per 100,000	28.60682
Cyprus	2050	All ages	Both	Diabetes mellitus	Deaths per 100,000	55.14991
Czechia	2050	All ages	Both	Diabetes mellitus	Deaths per 100,000	60.56058
Germany	2050	All ages	Both	Diabetes mellitus	Deaths per 100,000	26.87489
Djibouti	2050	All ages	Both	Diabetes mellitus	Deaths per 100,000	30.21527
Dominica	2050	All ages	Both	Diabetes mellitus	Deaths per 100,000	118.6151
Denmark	2050	All ages	Both	Diabetes mellitus	Deaths per 100,000	25.58883
Dominican Republic	2050	All ages	Both	Diabetes mellitus	Deaths per 100,000	45.65442
Algeria	2050	All ages	Both	Diabetes mellitus	Deaths per 100,000	26.40103
Ecuador	2050	All ages	Both	Diabetes mellitus	Deaths per 100,000	41.54438
Eritrea	2050	All ages	Both	Diabetes mellitus	Deaths per 100,000	27.9991
Spain	2050	All ages	Both	Diabetes mellitus	Deaths per 100,000	24.87766
Estonia	2050	All ages	Both	Diabetes mellitus	Deaths per 100,000	19.43829
Ethiopia	2050	All ages	Both	Diabetes mellitus	Deaths per 100,000	11.89

Finland	2050	All ages	Both	Diabetes mellitus	Deaths per 100,000	12.02965
Fiji	2050	All ages	Both	Diabetes mellitus	Deaths per 100,000	256.9808
France	2050	All ages	Both	Diabetes mellitus	Deaths per 100,000	28.75045
Micronesia (Federated States of)	2050	All ages	Both	Diabetes mellitus	Deaths per 100,000	105.8093
Gabon	2050	All ages	Both	Diabetes mellitus	Deaths per 100,000	43.37944
United Kingdom	2050	All ages	Both	Diabetes mellitus	Deaths per 100,000	13.02289
Georgia	2050	All ages	Both	Diabetes mellitus	Deaths per 100,000	29.95487
Ghana	2050	All ages	Both	Diabetes mellitus	Deaths per 100,000	30.41489
Guinea	2050	All ages	Both	Diabetes mellitus	Deaths per 100,000	16.43143
Gambia	2050	All ages	Both	Diabetes mellitus	Deaths per 100,000	19.636
Guinea-Bissau	2050	All ages	Both	Diabetes mellitus	Deaths per 100,000	20.79445
Equatorial Guinea	2050	All ages	Both	Diabetes mellitus	Deaths per 100,000	29.84261
Greece	2050	All ages	Both	Diabetes mellitus	Deaths per 100,000	22.61484
Grenada	2050	All ages	Both	Diabetes mellitus	Deaths per 100,000	132.4008
Greenland	2050	All ages	Both	Diabetes mellitus	Deaths per 100,000	16.81456
Guatemala	2050	All ages	Both	Diabetes mellitus	Deaths per 100,000	58.03705
Guam	2050	All ages	Both	Diabetes mellitus	Deaths per 100,000	28.58814
Guyana	2050	All ages	Both	Diabetes mellitus	Deaths per 100,000	94.25743
Honduras	2050	All ages	Both	Diabetes mellitus	Deaths per 100,000	22.21474
Croatia	2050	All ages	Both	Diabetes mellitus	Deaths per 100,000	51.58139
Haiti	2050	All ages	Both	Diabetes mellitus	Deaths per 100,000	55.79035
Hungary	2050	All ages	Both	Diabetes mellitus	Deaths per 100,000	27.49722
Indonesia	2050	All ages	Both	Diabetes mellitus	Deaths per 100,000	37.3522
India	2050	All ages	Both	Diabetes mellitus	Deaths per 100,000	37.18549

Ireland	2050	All ages	Both	Diabetes mellitus	Deaths per 100,000	14.0082
Iran (Islamic Republic of)	2050	All ages	Both	Diabetes mellitus	Deaths per 100,000	43.39525
Iraq	2050	All ages	Both	Diabetes mellitus	Deaths per 100,000	36.11921
Iceland	2050	All ages	Both	Diabetes mellitus	Deaths per 100,000	9.38407
Israel	2050	All ages	Both	Diabetes mellitus	Deaths per 100,000	33.03599
Italy	2050	All ages	Both	Diabetes mellitus	Deaths per 100,000	45.57136
Jamaica	2050	All ages	Both	Diabetes mellitus	Deaths per 100,000	146.0772
Jordan	2050	All ages	Both	Diabetes mellitus	Deaths per 100,000	33.67541
Japan	2050	All ages	Both	Diabetes mellitus	Deaths per 100,000	7.766606
Kazakhstan	2050	All ages	Both	Diabetes mellitus	Deaths per 100,000	10.10387
Kenya	2050	All ages	Both	Diabetes mellitus	Deaths per 100,000	21.88494
Kyrgyzstan	2050	All ages	Both	Diabetes mellitus	Deaths per 100,000	8.475752
Cambodia	2050	All ages	Both	Diabetes mellitus	Deaths per 100,000	31.78522
Kiribati	2050	All ages	Both	Diabetes mellitus	Deaths per 100,000	129.5074
Saint Kitts and Nevis	2050	All ages	Both	Diabetes mellitus	Deaths per 100,000	94.77525
Republic of Korea	2050	All ages	Both	Diabetes mellitus	Deaths per 100,000	38.44871
Kuwait	2050	All ages	Both	Diabetes mellitus	Deaths per 100,000	44.31317
Lao People's Democratic Republic	2050	All ages	Both	Diabetes mellitus	Deaths per 100,000	27.97747
Lebanon	2050	All ages	Both	Diabetes mellitus	Deaths per 100,000	54.08728
Liberia	2050	All ages	Both	Diabetes mellitus	Deaths per 100,000	25.55412
Libya	2050	All ages	Both	Diabetes mellitus	Deaths per 100,000	43.96701
Saint Lucia	2050	All ages	Both	Diabetes mellitus	Deaths per 100,000	116.0516
Sri Lanka	2050	All ages	Both	Diabetes mellitus	Deaths per 100,000	97.94908
Lesotho	2050	All ages	Both	Diabetes mellitus	Deaths per 100,000	55.015

Lithuania	2050	All ages	Both	Diabetes mellitus	Deaths per 100,000	22.76352
Luxembourg	2050	All ages	Both	Diabetes mellitus	Deaths per 100,000	14.31025
Latvia	2050	All ages	Both	Diabetes mellitus	Deaths per 100,000	30.21276
Morocco	2050	All ages	Both	Diabetes mellitus	Deaths per 100,000	36.42136
Monaco	2050	All ages	Both	Diabetes mellitus	Deaths per 100,000	14.55271
Republic of Moldova	2050	All ages	Both	Diabetes mellitus	Deaths per 100,000	21.60424
Madagascar	2050	All ages	Both	Diabetes mellitus	Deaths per 100,000	15.85306
Maldives	2050	All ages	Both	Diabetes mellitus	Deaths per 100,000	23.04953
Mexico	2050	All ages	Both	Diabetes mellitus	Deaths per 100,000	97.85691
Marshall Islands	2050	All ages	Both	Diabetes mellitus	Deaths per 100,000	115.1166
North Macedonia	2050	All ages	Both	Diabetes mellitus	Deaths per 100,000	69.75021
Mali	2050	All ages	Both	Diabetes mellitus	Deaths per 100,000	15.35832
Malta	2050	All ages	Both	Diabetes mellitus	Deaths per 100,000	32.78851
Myanmar	2050	All ages	Both	Diabetes mellitus	Deaths per 100,000	55.37845
Montenegro	2050	All ages	Both	Diabetes mellitus	Deaths per 100,000	33.72675
Mongolia	2050	All ages	Both	Diabetes mellitus	Deaths per 100,000	6.717381
Northern Mariana Islands	2050	All ages	Both	Diabetes mellitus	Deaths per 100,000	94.05301
Mozambique	2050	All ages	Both	Diabetes mellitus	Deaths per 100,000	20.84725
Mauritania	2050	All ages	Both	Diabetes mellitus	Deaths per 100,000	17.37926
Mauritius	2050	All ages	Both	Diabetes mellitus	Deaths per 100,000	205.6163
Malawi	2050	All ages	Both	Diabetes mellitus	Deaths per 100,000	20.4913
Malaysia	2050	All ages	Both	Diabetes mellitus	Deaths per 100,000	20.40932
Namibia	2050	All ages	Both	Diabetes mellitus	Deaths per 100,000	40.72204
Niger	2050	All ages	Both	Diabetes mellitus	Deaths per 100,000	13.76808

Nigeria	2050	All ages	Both	Diabetes mellitus	Deaths per 100,000	15.77362
Nicaragua	2050	All ages	Both	Diabetes mellitus	Deaths per 100,000	44.51595
Niue	2050	All ages	Both	Diabetes mellitus	Deaths per 100,000	161.7134
Netherlands	2050	All ages	Both	Diabetes mellitus	Deaths per 100,000	22.05241
Norway	2050	All ages	Both	Diabetes mellitus	Deaths per 100,000	12.50095
Nepal	2050	All ages	Both	Diabetes mellitus	Deaths per 100,000	38.4027
Nauru	2050	All ages	Both	Diabetes mellitus	Deaths per 100,000	79.90131
New Zealand	2050	All ages	Both	Diabetes mellitus	Deaths per 100,000	18.46038
Oman	2050	All ages	Both	Diabetes mellitus	Deaths per 100,000	60.41079
Pakistan	2050	All ages	Both	Diabetes mellitus	Deaths per 100,000	34.25697
Panama	2050	All ages	Both	Diabetes mellitus	Deaths per 100,000	43.16591
Peru	2050	All ages	Both	Diabetes mellitus	Deaths per 100,000	27.3778
Philippines	2050	All ages	Both	Diabetes mellitus	Deaths per 100,000	35.83791
Palau	2050	All ages	Both	Diabetes mellitus	Deaths per 100,000	185.0749
Papua New Guinea	2050	All ages	Both	Diabetes mellitus	Deaths per 100,000	54.56381
Poland	2050	All ages	Both	Diabetes mellitus	Deaths per 100,000	36.90052
Puerto Rico	2050	All ages	Both	Diabetes mellitus	Deaths per 100,000	132.0003
Democratic People's Republic of Korea	2050	All ages	Both	Diabetes mellitus	Deaths per 100,000	25.64264
Portugal	2050	All ages	Both	Diabetes mellitus	Deaths per 100,000	45.24618
Paraguay	2050	All ages	Both	Diabetes mellitus	Deaths per 100,000	73.18795
Palestine	2050	All ages	Both	Diabetes mellitus	Deaths per 100,000	45.96777
Qatar	2050	All ages	Both	Diabetes mellitus	Deaths per 100,000	75.19823
Romania	2050	All ages	Both	Diabetes mellitus	Deaths per 100,000	16.83782
Russian Federation	2050	All ages	Both	Diabetes mellitus	Deaths per 100,000	29.38175

Rwanda	2050	All ages	Both	Diabetes mellitus	Deaths per 100,000	19.2953
Saudi Arabia	2050	All ages	Both	Diabetes mellitus	Deaths per 100,000	45.97709
Senegal	2050	All ages	Both	Diabetes mellitus	Deaths per 100,000	22.59967
Singapore	2050	All ages	Both	Diabetes mellitus	Deaths per 100,000	4.796241
Solomon Islands	2050	All ages	Both	Diabetes mellitus	Deaths per 100,000	81.34148
Sierra Leone	2050	All ages	Both	Diabetes mellitus	Deaths per 100,000	16.36204
El Salvador	2050	All ages	Both	Diabetes mellitus	Deaths per 100,000	71.67764
San Marino	2050	All ages	Both	Diabetes mellitus	Deaths per 100,000	22.69101
Somalia	2050	All ages	Both	Diabetes mellitus	Deaths per 100,000	18.86486
Serbia	2050	All ages	Both	Diabetes mellitus	Deaths per 100,000	46.57164
South Sudan	2050	All ages	Both	Diabetes mellitus	Deaths per 100,000	20.34732
Sao Tome and Principe	2050	All ages	Both	Diabetes mellitus	Deaths per 100,000	15.23473
Suriname	2050	All ages	Both	Diabetes mellitus	Deaths per 100,000	64.44066
Slovakia	2050	All ages	Both	Diabetes mellitus	Deaths per 100,000	20.14015
Slovenia	2050	All ages	Both	Diabetes mellitus	Deaths per 100,000	21.05984
Sweden	2050	All ages	Both	Diabetes mellitus	Deaths per 100,000	17.59542
Eswatini	2050	All ages	Both	Diabetes mellitus	Deaths per 100,000	60.41942
Seychelles	2050	All ages	Both	Diabetes mellitus	Deaths per 100,000	32.26996
Syrian Arab Republic	2050	All ages	Both	Diabetes mellitus	Deaths per 100,000	26.9821
Chad	2050	All ages	Both	Diabetes mellitus	Deaths per 100,000	8.756827
Togo	2050	All ages	Both	Diabetes mellitus	Deaths per 100,000	21.79937
Thailand	2050	All ages	Both	Diabetes mellitus	Deaths per 100,000	57.70431
Tajikistan	2050	All ages	Both	Diabetes mellitus	Deaths per 100,000	13.42531
Tokelau	2050	All ages	Both	Diabetes mellitus	Deaths per 100,000	96.93199

Turkmenistan	2050	All ages	Both	Diabetes mellitus	Deaths per 100,000	20.91245
Timor-Leste	2050	All ages	Both	Diabetes mellitus	Deaths per 100,000	13.95062
Tonga	2050	All ages	Both	Diabetes mellitus	Deaths per 100,000	81.74031
Trinidad and Tobago	2050	All ages	Both	Diabetes mellitus	Deaths per 100,000	214.6536
Tunisia	2050	All ages	Both	Diabetes mellitus	Deaths per 100,000	38.27854
Turkey	2050	All ages	Both	Diabetes mellitus	Deaths per 100,000	49.15262
Tuvalu	2050	All ages	Both	Diabetes mellitus	Deaths per 100,000	79.6345
Taiwan (Province of China)	2050	All ages	Both	Diabetes mellitus	Deaths per 100,000	75.17839
United Republic of Tanzania	2050	All ages	Both	Diabetes mellitus	Deaths per 100,000	20.14016
Uganda	2050	All ages	Both	Diabetes mellitus	Deaths per 100,000	14.92391
Ukraine	2050	All ages	Both	Diabetes mellitus	Deaths per 100,000	5.700438
Uruguay	2050	All ages	Both	Diabetes mellitus	Deaths per 100,000	35.51761
United States of America	2050	All ages	Both	Diabetes mellitus	Deaths per 100,000	22.18185
Uzbekistan	2050	All ages	Both	Diabetes mellitus	Deaths per 100,000	30.24497
Saint Vincent and the Grenadines	2050	All ages	Both	Diabetes mellitus	Deaths per 100,000	135.4856
Venezuela (Bolivarian Republic of)	2050	All ages	Both	Diabetes mellitus	Deaths per 100,000	108.4929
United States Virgin Islands	2050	All ages	Both	Diabetes mellitus	Deaths per 100,000	110.8967
Viet Nam	2050	All ages	Both	Diabetes mellitus	Deaths per 100,000	52.82328
Vanuatu	2050	All ages	Both	Diabetes mellitus	Deaths per 100,000	56.69937
Samoa	2050	All ages	Both	Diabetes mellitus	Deaths per 100,000	58.09516
Yemen	2050	All ages	Both	Diabetes mellitus	Deaths per 100,000	9.538627
South Africa	2050	All ages	Both	Diabetes mellitus	Deaths per 100,000	77.45173
Zambia	2050	All ages	Both	Diabetes mellitus	Deaths per 100,000	18.30879
Zimbabwe	2050	All ages	Both	Diabetes mellitus	Deaths per 100,000	31.39677

Egypt	2050	All ages	Both	Diabetes mellitus	Deaths per 100,000	44.54457
Sudan	2050	All ages	Both	Diabetes mellitus	Deaths per 100,000	12.63409
China	2050	All ages	Both	Diabetes mellitus	Deaths per 100,000	22.49299
Location	Year	Age	Sex	Cause of death or injury	Measure	Value
Afghanistan	2050	All ages	Both	Diabetes mellitus	DALYs per 100,000	1444.422
Angola	2050	All ages	Both	Diabetes mellitus	DALYs per 100,000	1076.684
Albania	2050	All ages	Both	Diabetes mellitus	DALYs per 100,000	1039.167
Andorra	2050	All ages	Both	Diabetes mellitus	DALYs per 100,000	1617.885
United Arab Emirates	2050	All ages	Both	Diabetes mellitus	DALYs per 100,000	3417.268
Argentina	2050	All ages	Both	Diabetes mellitus	DALYs per 100,000	1637.492
Armenia	2050	All ages	Both	Diabetes mellitus	DALYs per 100,000	1507.94
American Samoa	2050	All ages	Both	Diabetes mellitus	DALYs per 100,000	4640.201
Antigua and Barbuda	2050	All ages	Both	Diabetes mellitus	DALYs per 100,000	4726.228
Australia	2050	All ages	Both	Diabetes mellitus	DALYs per 100,000	1154.823
Austria	2050	All ages	Both	Diabetes mellitus	DALYs per 100,000	947.4214
Azerbaijan	2050	All ages	Both	Diabetes mellitus	DALYs per 100,000	1548.702
Burundi	2050	All ages	Both	Diabetes mellitus	DALYs per 100,000	700.3434
Belgium	2050	All ages	Both	Diabetes mellitus	DALYs per 100,000	1174.246
Benin	2050	All ages	Both	Diabetes mellitus	DALYs per 100,000	978.7664
Burkina Faso	2050	All ages	Both	Diabetes mellitus	DALYs per 100,000	625.9387
Bangladesh	2050	All ages	Both	Diabetes mellitus	DALYs per 100,000	2285.596
Bulgaria	2050	All ages	Both	Diabetes mellitus	DALYs per 100,000	1955.578
Bahrain	2050	All ages	Both	Diabetes mellitus	DALYs per 100,000	6078.24
Bahamas	2050	All ages	Both	Diabetes mellitus	DALYs per 100,000	3550.097

Bosnia and Herzegovina	2050	All ages	Both	Diabetes mellitus	DALYs per 100,000	3410.268
Belarus	2050	All ages	Both	Diabetes mellitus	DALYs per 100,000	833.2279
Belize	2050	All ages	Both	Diabetes mellitus	DALYs per 100,000	2634.496
Bermuda	2050	All ages	Both	Diabetes mellitus	DALYs per 100,000	3063.312
Bolivia (Plurinational State of)	2050	All ages	Both	Diabetes mellitus	DALYs per 100,000	1963.769
Brazil	2050	All ages	Both	Diabetes mellitus	DALYs per 100,000	2316.635
Barbados	2050	All ages	Both	Diabetes mellitus	DALYs per 100,000	5124.248
Brunei Darussalam	2050	All ages	Both	Diabetes mellitus	DALYs per 100,000	4058.873
Bhutan	2050	All ages	Both	Diabetes mellitus	DALYs per 100,000	1787.376
Botswana	2050	All ages	Both	Diabetes mellitus	DALYs per 100,000	2166.006
Central African Republic	2050	All ages	Both	Diabetes mellitus	DALYs per 100,000	1914.508
Canada	2050	All ages	Both	Diabetes mellitus	DALYs per 100,000	1525.514
Switzerland	2050	All ages	Both	Diabetes mellitus	DALYs per 100,000	1396.659
Chile	2050	All ages	Both	Diabetes mellitus	DALYs per 100,000	1795.967
CÃ´te d'Ivoire	2050	All ages	Both	Diabetes mellitus	DALYs per 100,000	1262.983
Cameroon	2050	All ages	Both	Diabetes mellitus	DALYs per 100,000	1354.303
Democratic Republic of the Congo	2050	All ages	Both	Diabetes mellitus	DALYs per 100,000	1235.845
Congo	2050	All ages	Both	Diabetes mellitus	DALYs per 100,000	1935.44
Cook Islands	2050	All ages	Both	Diabetes mellitus	DALYs per 100,000	6548.345
Colombia	2050	All ages	Both	Diabetes mellitus	DALYs per 100,000	2031.862
Comoros	2050	All ages	Both	Diabetes mellitus	DALYs per 100,000	1904.473
Cabo Verde	2050	All ages	Both	Diabetes mellitus	DALYs per 100,000	2700.218
Costa Rica	2050	All ages	Both	Diabetes mellitus	DALYs per 100,000	2405.278
Cuba	2050	All ages	Both	Diabetes mellitus	DALYs per 100,000	2458.837

Cyprus	2050	All ages	Both	Diabetes mellitus	DALYs per 100,000	2036.638
Czechia	2050	All ages	Both	Diabetes mellitus	DALYs per 100,000	2130.424
Germany	2050	All ages	Both	Diabetes mellitus	DALYs per 100,000	1056.544
Djibouti	2050	All ages	Both	Diabetes mellitus	DALYs per 100,000	1110.099
Dominica	2050	All ages	Both	Diabetes mellitus	DALYs per 100,000	4938.099
Denmark	2050	All ages	Both	Diabetes mellitus	DALYs per 100,000	917.5411
Dominican Republic	2050	All ages	Both	Diabetes mellitus	DALYs per 100,000	3037.946
Algeria	2050	All ages	Both	Diabetes mellitus	DALYs per 100,000	2716.162
Ecuador	2050	All ages	Both	Diabetes mellitus	DALYs per 100,000	2528.085
Eritrea	2050	All ages	Both	Diabetes mellitus	DALYs per 100,000	1186.745
Spain	2050	All ages	Both	Diabetes mellitus	DALYs per 100,000	1801.982
Estonia	2050	All ages	Both	Diabetes mellitus	DALYs per 100,000	1339.144
Ethiopia	2050	All ages	Both	Diabetes mellitus	DALYs per 100,000	608.1298
Finland	2050	All ages	Both	Diabetes mellitus	DALYs per 100,000	1322.722
Fiji	2050	All ages	Both	Diabetes mellitus	DALYs per 100,000	7917.793
France	2050	All ages	Both	Diabetes mellitus	DALYs per 100,000	1021.08
Micronesia (Federated States of)	2050	All ages	Both	Diabetes mellitus	DALYs per 100,000	4611.309
Gabon	2050	All ages	Both	Diabetes mellitus	DALYs per 100,000	2129.839
United Kingdom	2050	All ages	Both	Diabetes mellitus	DALYs per 100,000	1237.238
Georgia	2050	All ages	Both	Diabetes mellitus	DALYs per 100,000	1498.726
Ghana	2050	All ages	Both	Diabetes mellitus	DALYs per 100,000	1544.41
Guinea	2050	All ages	Both	Diabetes mellitus	DALYs per 100,000	829.1927
Gambia	2050	All ages	Both	Diabetes mellitus	DALYs per 100,000	1111.182
Guinea-Bissau	2050	All ages	Both	Diabetes mellitus	DALYs per 100,000	1182.715

Equatorial Guinea	2050	All ages	Both	Diabetes mellitus	DALYs per 100,000	1763.501
Greece	2050	All ages	Both	Diabetes mellitus	DALYs per 100,000	1626.845
Grenada	2050	All ages	Both	Diabetes mellitus	DALYs per 100,000	5331.626
Greenland	2050	All ages	Both	Diabetes mellitus	DALYs per 100,000	859.7077
Guatemala	2050	All ages	Both	Diabetes mellitus	DALYs per 100,000	3078.412
Guam	2050	All ages	Both	Diabetes mellitus	DALYs per 100,000	1960.922
Guyana	2050	All ages	Both	Diabetes mellitus	DALYs per 100,000	4707.676
Honduras	2050	All ages	Both	Diabetes mellitus	DALYs per 100,000	1875.688
Croatia	2050	All ages	Both	Diabetes mellitus	DALYs per 100,000	2384.714
Haiti	2050	All ages	Both	Diabetes mellitus	DALYs per 100,000	3040.142
Hungary	2050	All ages	Both	Diabetes mellitus	DALYs per 100,000	1729.002
Indonesia	2050	All ages	Both	Diabetes mellitus	DALYs per 100,000	1683.09
India	2050	All ages	Both	Diabetes mellitus	DALYs per 100,000	1693.059
Ireland	2050	All ages	Both	Diabetes mellitus	DALYs per 100,000	839.6686
Iran (Islamic Republic of)	2050	All ages	Both	Diabetes mellitus	DALYs per 100,000	3048.593
Iraq	2050	All ages	Both	Diabetes mellitus	DALYs per 100,000	2581.51
Iceland	2050	All ages	Both	Diabetes mellitus	DALYs per 100,000	768.3464
Israel	2050	All ages	Both	Diabetes mellitus	DALYs per 100,000	1139.988
Italy	2050	All ages	Both	Diabetes mellitus	DALYs per 100,000	1663.025
Jamaica	2050	All ages	Both	Diabetes mellitus	DALYs per 100,000	4594.904
Jordan	2050	All ages	Both	Diabetes mellitus	DALYs per 100,000	2714.973
Japan	2050	All ages	Both	Diabetes mellitus	DALYs per 100,000	1421.805
Kazakhstan	2050	All ages	Both	Diabetes mellitus	DALYs per 100,000	1089.405
Kenya	2050	All ages	Both	Diabetes mellitus	DALYs per 100,000	868.1008

Kyrgyzstan	2050	All ages	Both	Diabetes mellitus	DALYs per 100,000	851.2256
Cambodia	2050	All ages	Both	Diabetes mellitus	DALYs per 100,000	1411.679
Kiribati	2050	All ages	Both	Diabetes mellitus	DALYs per 100,000	5233.257
Saint Kitts and Nevis	2050	All ages	Both	Diabetes mellitus	DALYs per 100,000	4682.255
Republic of Korea	2050	All ages	Both	Diabetes mellitus	DALYs per 100,000	2732.454
Kuwait	2050	All ages	Both	Diabetes mellitus	DALYs per 100,000	4376.407
Lao People's Democratic Republic	2050	All ages	Both	Diabetes mellitus	DALYs per 100,000	1589.373
Lebanon	2050	All ages	Both	Diabetes mellitus	DALYs per 100,000	3868.955
Liberia	2050	All ages	Both	Diabetes mellitus	DALYs per 100,000	1423.881
Libya	2050	All ages	Both	Diabetes mellitus	DALYs per 100,000	4162.147
Saint Lucia	2050	All ages	Both	Diabetes mellitus	DALYs per 100,000	5122.015
Sri Lanka	2050	All ages	Both	Diabetes mellitus	DALYs per 100,000	3891.432
Lesotho	2050	All ages	Both	Diabetes mellitus	DALYs per 100,000	2106.511
Lithuania	2050	All ages	Both	Diabetes mellitus	DALYs per 100,000	1330.299
Luxembourg	2050	All ages	Both	Diabetes mellitus	DALYs per 100,000	911.4437
Latvia	2050	All ages	Both	Diabetes mellitus	DALYs per 100,000	1498.764
Morocco	2050	All ages	Both	Diabetes mellitus	DALYs per 100,000	3718.688
Monaco	2050	All ages	Both	Diabetes mellitus	DALYs per 100,000	976.4132
Republic of Moldova	2050	All ages	Both	Diabetes mellitus	DALYs per 100,000	2006.378
Madagascar	2050	All ages	Both	Diabetes mellitus	DALYs per 100,000	694.4689
Maldives	2050	All ages	Both	Diabetes mellitus	DALYs per 100,000	2050.082
Mexico	2050	All ages	Both	Diabetes mellitus	DALYs per 100,000	3845.698
Marshall Islands	2050	All ages	Both	Diabetes mellitus	DALYs per 100,000	5479.026
North Macedonia	2050	All ages	Both	Diabetes mellitus	DALYs per 100,000	3084.311

Mali	2050	All ages	Both	Diabetes mellitus	DALYs per 100,000	1031.325
Malta	2050	All ages	Both	Diabetes mellitus	DALYs per 100,000	1664.802
Myanmar	2050	All ages	Both	Diabetes mellitus	DALYs per 100,000	2347.996
Montenegro	2050	All ages	Both	Diabetes mellitus	DALYs per 100,000	2077.866
Mongolia	2050	All ages	Both	Diabetes mellitus	DALYs per 100,000	624.3669
Northern Mariana Islands	2050	All ages	Both	Diabetes mellitus	DALYs per 100,000	3585.453
Mozambique	2050	All ages	Both	Diabetes mellitus	DALYs per 100,000	951.7809
Mauritania	2050	All ages	Both	Diabetes mellitus	DALYs per 100,000	846.97
Mauritius	2050	All ages	Both	Diabetes mellitus	DALYs per 100,000	6479.697
Malawi	2050	All ages	Both	Diabetes mellitus	DALYs per 100,000	758.8322
Malaysia	2050	All ages	Both	Diabetes mellitus	DALYs per 100,000	1760.249
Namibia	2050	All ages	Both	Diabetes mellitus	DALYs per 100,000	1603.371
Niger	2050	All ages	Both	Diabetes mellitus	DALYs per 100,000	574.0465
Nigeria	2050	All ages	Both	Diabetes mellitus	DALYs per 100,000	800.5483
Nicaragua	2050	All ages	Both	Diabetes mellitus	DALYs per 100,000	2658.258
Niue	2050	All ages	Both	Diabetes mellitus	DALYs per 100,000	6065.643
Netherlands	2050	All ages	Both	Diabetes mellitus	DALYs per 100,000	1039.094
Norway	2050	All ages	Both	Diabetes mellitus	DALYs per 100,000	860.02
Nepal	2050	All ages	Both	Diabetes mellitus	DALYs per 100,000	2055.775
Nauru	2050	All ages	Both	Diabetes mellitus	DALYs per 100,000	3964.674
New Zealand	2050	All ages	Both	Diabetes mellitus	DALYs per 100,000	1158.015
Oman	2050	All ages	Both	Diabetes mellitus	DALYs per 100,000	3727.354
Pakistan	2050	All ages	Both	Diabetes mellitus	DALYs per 100,000	2013.231
Panama	2050	All ages	Both	Diabetes mellitus	DALYs per 100,000	2271.501

Peru	2050	All ages	Both	Diabetes mellitus	DALYs per 100,000	1279.341
Philippines	2050	All ages	Both	Diabetes mellitus	DALYs per 100,000	1492.943
Palau	2050	All ages	Both	Diabetes mellitus	DALYs per 100,000	6548.235
Papua New Guinea	2050	All ages	Both	Diabetes mellitus	DALYs per 100,000	2452.151
Poland	2050	All ages	Both	Diabetes mellitus	DALYs per 100,000	1943.793
Puerto Rico	2050	All ages	Both	Diabetes mellitus	DALYs per 100,000	5172.176
Democratic People's Republic of Korea	2050	All ages	Both	Diabetes mellitus	DALYs per 100,000	1435.913
Portugal	2050	All ages	Both	Diabetes mellitus	DALYs per 100,000	1986.635
Paraguay	2050	All ages	Both	Diabetes mellitus	DALYs per 100,000	2841.352
Palestine	2050	All ages	Both	Diabetes mellitus	DALYs per 100,000	2425.521
Qatar	2050	All ages	Both	Diabetes mellitus	DALYs per 100,000	4118.926
Romania	2050	All ages	Both	Diabetes mellitus	DALYs per 100,000	1251.837
Russian Federation	2050	All ages	Both	Diabetes mellitus	DALYs per 100,000	1471.483
Rwanda	2050	All ages	Both	Diabetes mellitus	DALYs per 100,000	716.1125
Saudi Arabia	2050	All ages	Both	Diabetes mellitus	DALYs per 100,000	3710.967
Senegal	2050	All ages	Both	Diabetes mellitus	DALYs per 100,000	1252.937
Singapore	2050	All ages	Both	Diabetes mellitus	DALYs per 100,000	2003.366
Solomon Islands	2050	All ages	Both	Diabetes mellitus	DALYs per 100,000	3102.828
Sierra Leone	2050	All ages	Both	Diabetes mellitus	DALYs per 100,000	963.0154
El Salvador	2050	All ages	Both	Diabetes mellitus	DALYs per 100,000	2966.811
San Marino	2050	All ages	Both	Diabetes mellitus	DALYs per 100,000	1309.999
Somalia	2050	All ages	Both	Diabetes mellitus	DALYs per 100,000	796.631
Serbia	2050	All ages	Both	Diabetes mellitus	DALYs per 100,000	2773.977
South Sudan	2050	All ages	Both	Diabetes mellitus	DALYs per 100,000	706.9003

Sao Tome and Principe	2050	All ages	Both	Diabetes mellitus	DALYs per 100,000	1387.933
Suriname	2050	All ages	Both	Diabetes mellitus	DALYs per 100,000	3596.936
Slovakia	2050	All ages	Both	Diabetes mellitus	DALYs per 100,000	1385.264
Slovenia	2050	All ages	Both	Diabetes mellitus	DALYs per 100,000	1411.115
Sweden	2050	All ages	Both	Diabetes mellitus	DALYs per 100,000	945.4858
Eswatini	2050	All ages	Both	Diabetes mellitus	DALYs per 100,000	2581.262
Seychelles	2050	All ages	Both	Diabetes mellitus	DALYs per 100,000	2980.453
Syrian Arab Republic	2050	All ages	Both	Diabetes mellitus	DALYs per 100,000	2675.379
Chad	2050	All ages	Both	Diabetes mellitus	DALYs per 100,000	496.9629
Togo	2050	All ages	Both	Diabetes mellitus	DALYs per 100,000	1052.931
Thailand	2050	All ages	Both	Diabetes mellitus	DALYs per 100,000	3044.706
Tajikistan	2050	All ages	Both	Diabetes mellitus	DALYs per 100,000	842.9425
Tokelau	2050	All ages	Both	Diabetes mellitus	DALYs per 100,000	5041.065
Turkmenistan	2050	All ages	Both	Diabetes mellitus	DALYs per 100,000	991.3044
Timor-Leste	2050	All ages	Both	Diabetes mellitus	DALYs per 100,000	930.4323
Tonga	2050	All ages	Both	Diabetes mellitus	DALYs per 100,000	3374.569
Trinidad and Tobago	2050	All ages	Both	Diabetes mellitus	DALYs per 100,000	7481.583
Tunisia	2050	All ages	Both	Diabetes mellitus	DALYs per 100,000	3348.421
Turkey	2050	All ages	Both	Diabetes mellitus	DALYs per 100,000	2722.657
Tuvalu	2050	All ages	Both	Diabetes mellitus	DALYs per 100,000	3594.6
Taiwan (Province of China)	2050	All ages	Both	Diabetes mellitus	DALYs per 100,000	3392.616
United Republic of Tanzania	2050	All ages	Both	Diabetes mellitus	DALYs per 100,000	876.1856
Uganda	2050	All ages	Both	Diabetes mellitus	DALYs per 100,000	644.8862
Ukraine	2050	All ages	Both	Diabetes mellitus	DALYs per 100,000	1083.34

Uruguay	2050	All ages	Both	Diabetes mellitus	DALYs per 100,000	1688.21
United States of America	2050	All ages	Both	Diabetes mellitus	DALYs per 100,000	1816.516
Uzbekistan	2050	All ages	Both	Diabetes mellitus	DALYs per 100,000	1532.722
Saint Vincent and the Grenadines	2050	All ages	Both	Diabetes mellitus	DALYs per 100,000	5405.16
Venezuela (Bolivarian Republic of)	2050	All ages	Both	Diabetes mellitus	DALYs per 100,000	3777.392
United States Virgin Islands	2050	All ages	Both	Diabetes mellitus	DALYs per 100,000	5040.721
Viet Nam	2050	All ages	Both	Diabetes mellitus	DALYs per 100,000	1832.42
Vanuatu	2050	All ages	Both	Diabetes mellitus	DALYs per 100,000	2624.068
Samoa	2050	All ages	Both	Diabetes mellitus	DALYs per 100,000	2934.124
Yemen	2050	All ages	Both	Diabetes mellitus	DALYs per 100,000	1057.968
South Africa	2050	All ages	Both	Diabetes mellitus	DALYs per 100,000	2796.603
Zambia	2050	All ages	Both	Diabetes mellitus	DALYs per 100,000	1104.22
Zimbabwe	2050	All ages	Both	Diabetes mellitus	DALYs per 100,000	1335.78
Egypt	2050	All ages	Both	Diabetes mellitus	DALYs per 100,000	2658.621
Sudan	2050	All ages	Both	Diabetes mellitus	DALYs per 100,000	1490.497
China	2050	All ages	Both	Diabetes mellitus	DALYs per 100,000	1698.548

Supplementary figures

S1 Fig: Metabolic Risk factors Deaths by WHO regions

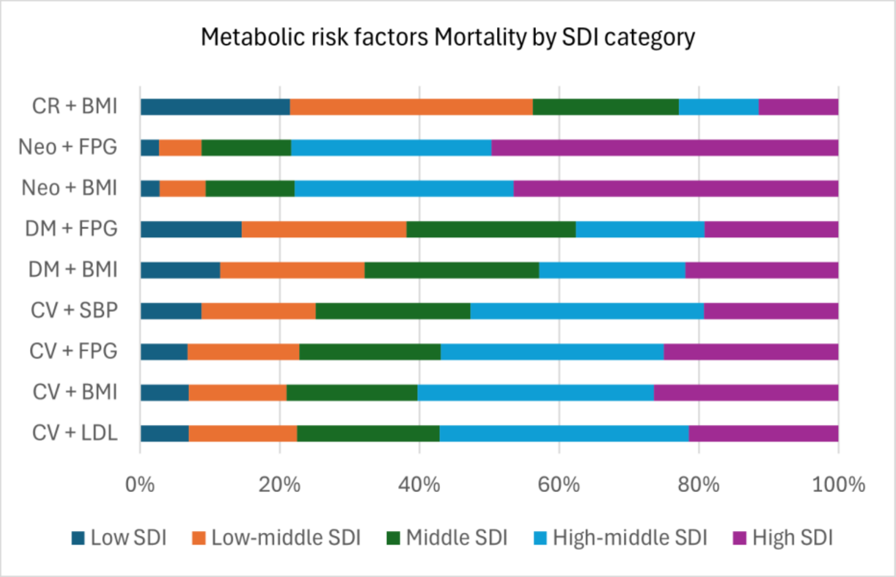

S2 Fig: Metabolic Risk factors DALYs by WHO regions

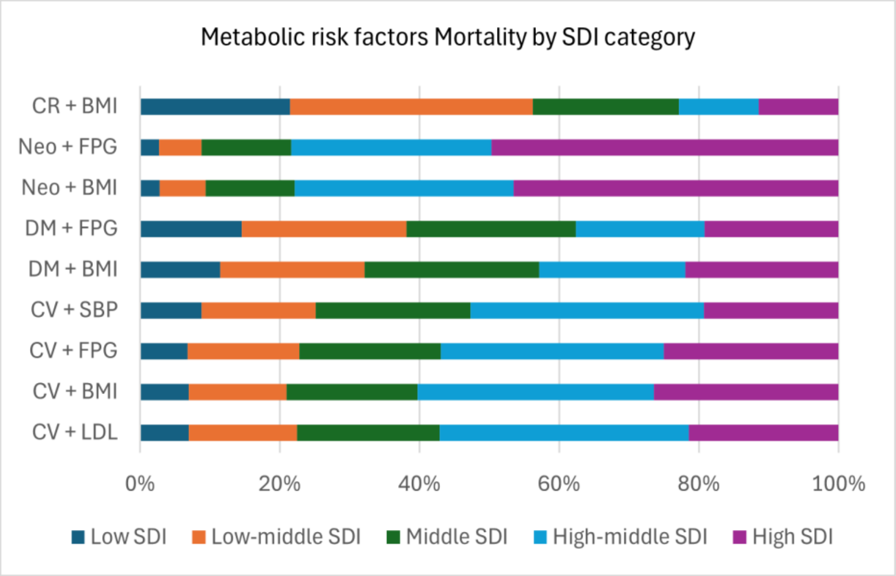

S3 Fig: Metabolic Risk factors Mortality by SDI category

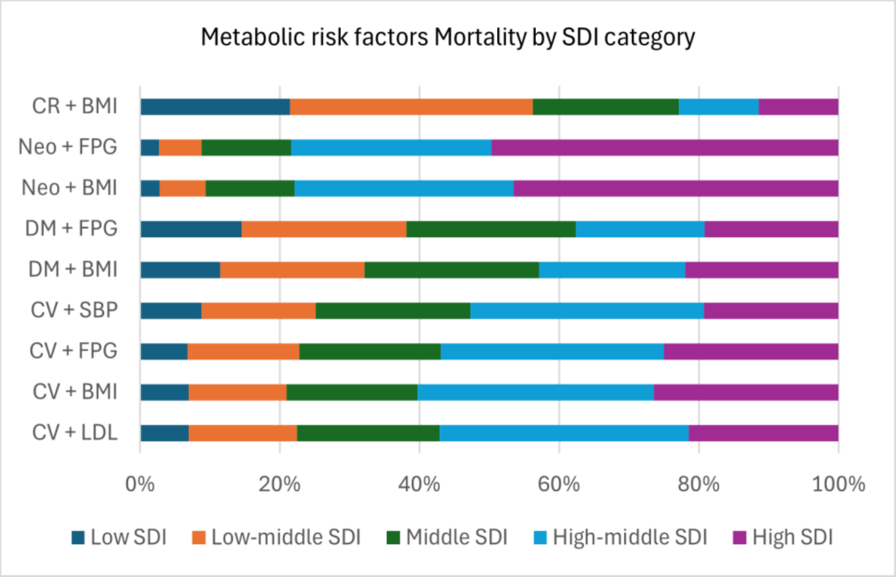

S4 Fig: Metabolic Risk factors DALYs by SDI category

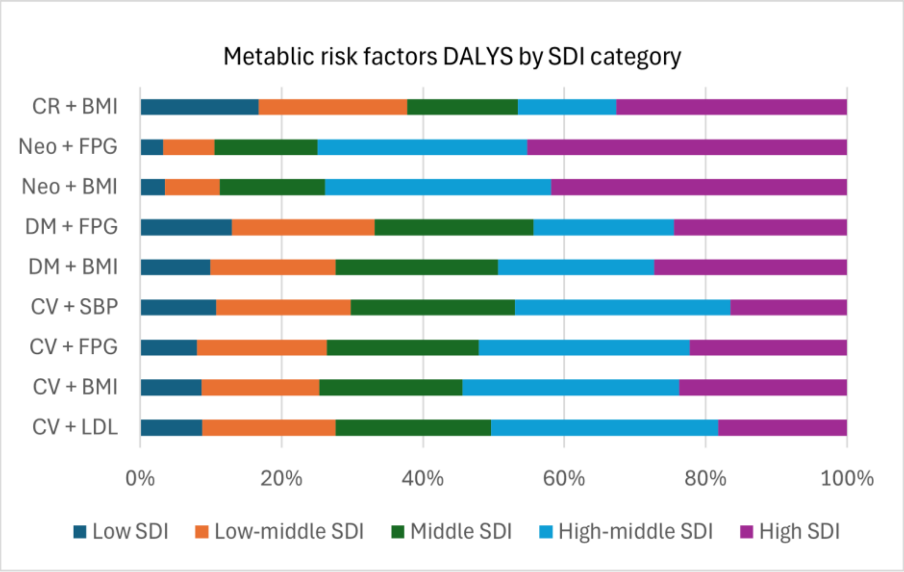

S5 Fig: Behavioural Risk factors Mortality by WHO regions

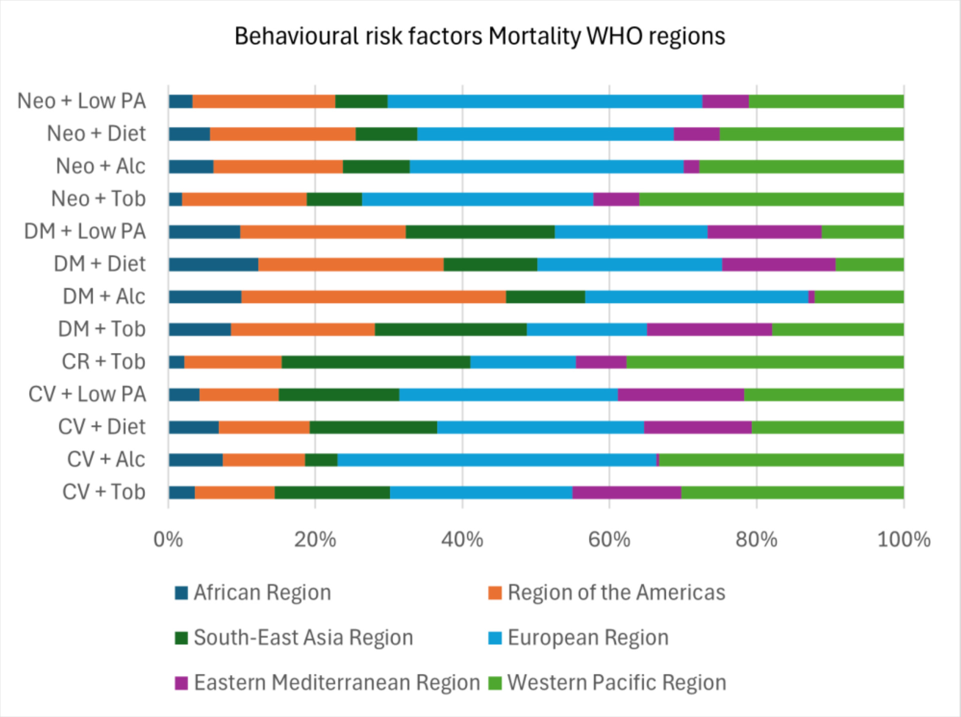

S6 Fig. Behavioural risk factors DALYs by WHO regions

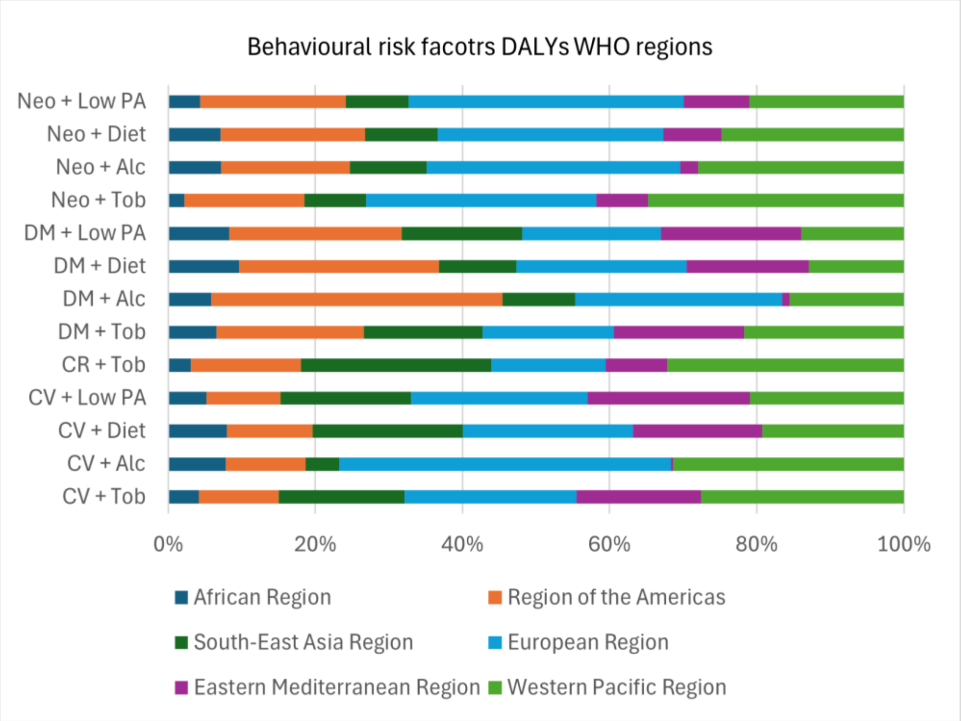

S7 Fig. Behavioural risk factors Mortality by SDI category

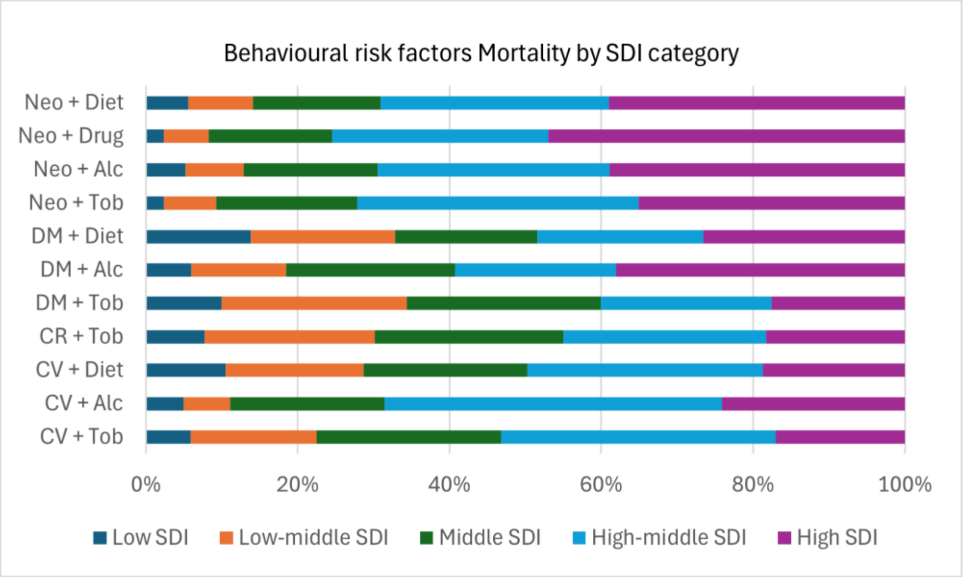

S8 Fig. behavioural risk factors DALYs by SDI category

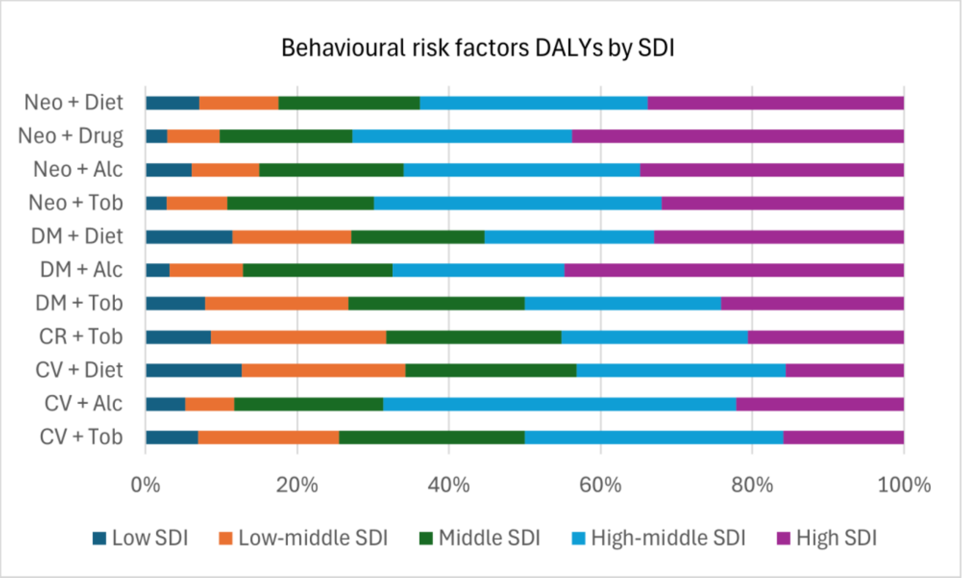

S9 Fig. Environmental risk factor Mortality by WHO regions

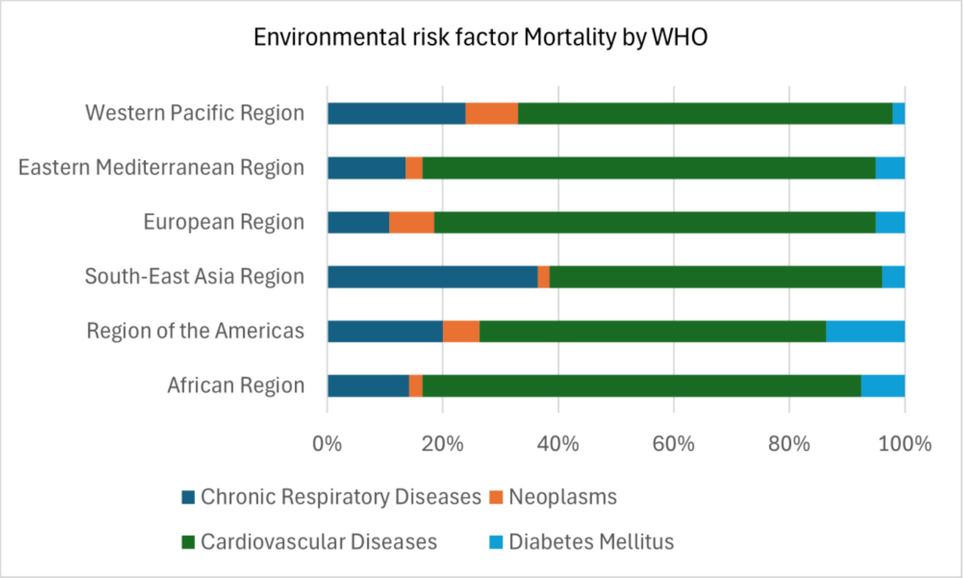

S10 Fig. Environmental risk factor DALYs by WHO regions

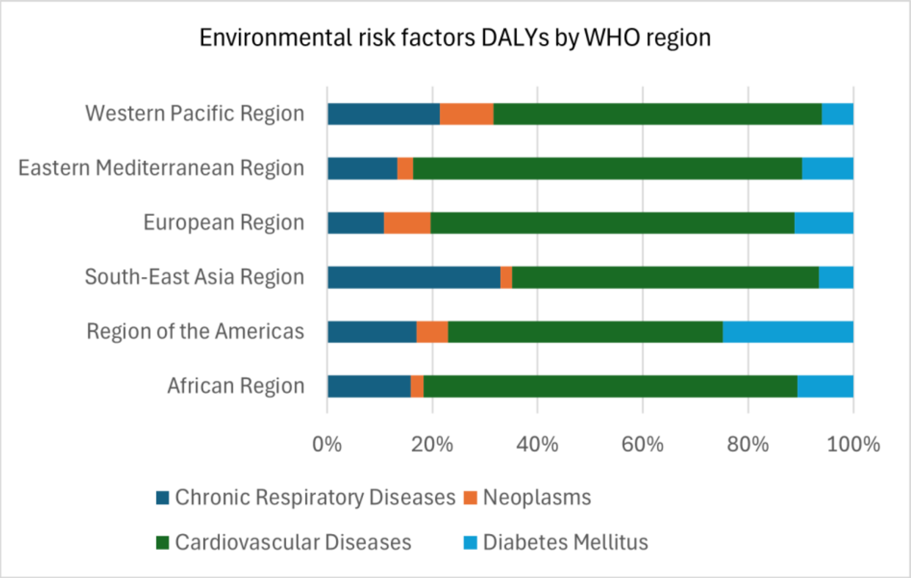

S11 Fig. Environmental risk factor Mortality by SDI category

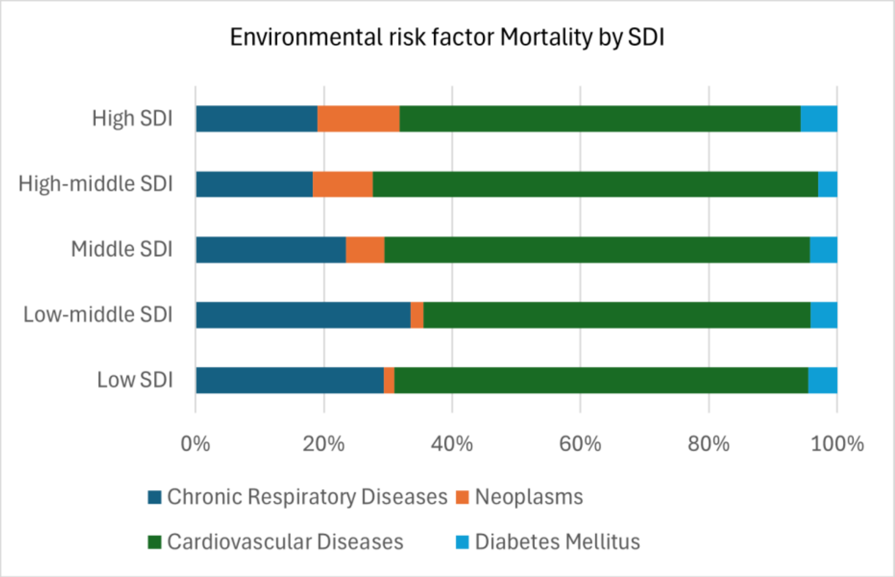

S12 Fig. Environmental risk factor DALYs by SDI category

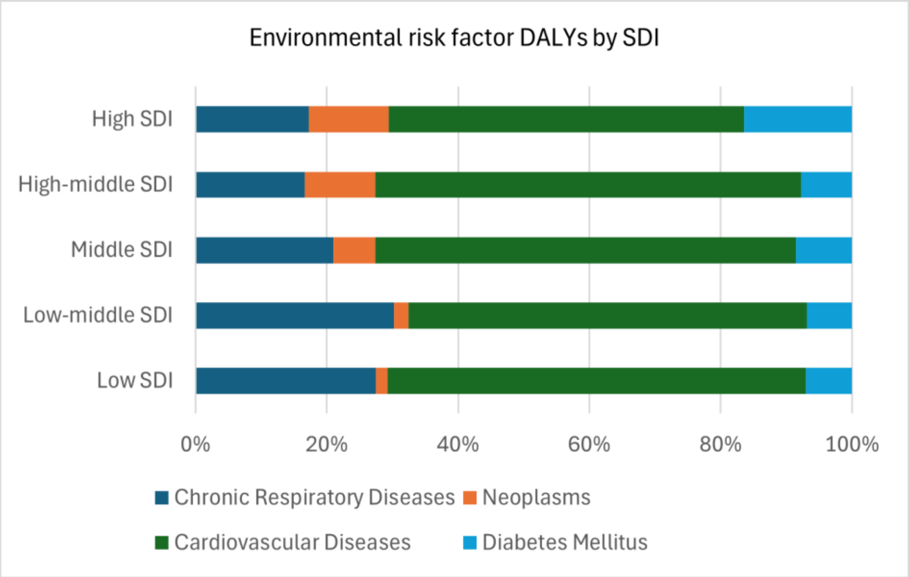

S13 Fig. Gender burden analysis by WHO regions

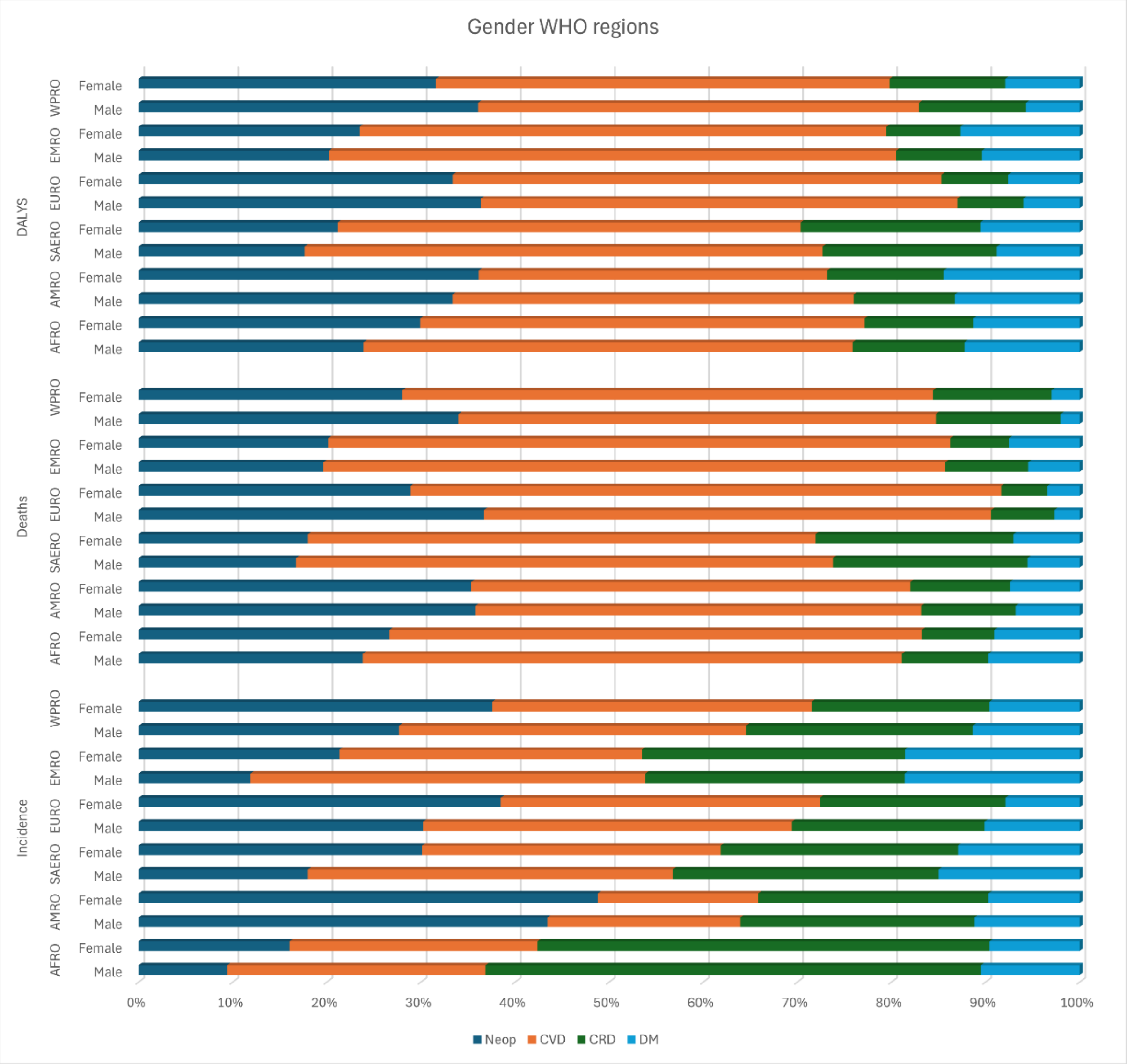

S14 Fig. Gender burden analysis by SDI category

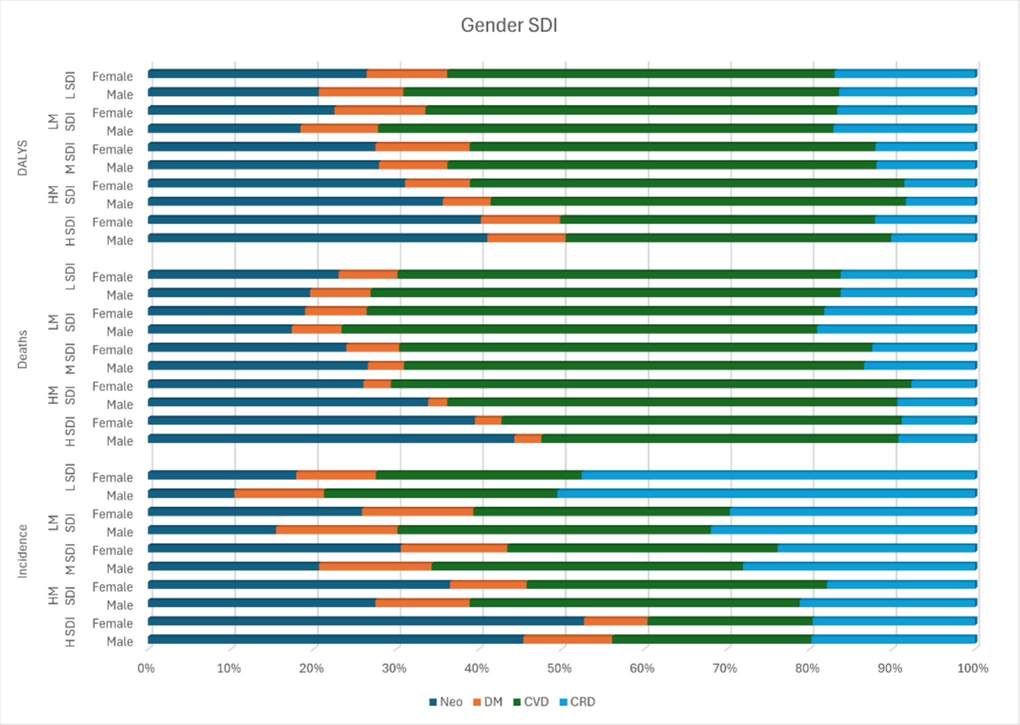

Gender SDI
DALYS
Deaths
Incidence
L SDI
LM SDI
M SDI
HM SDI
H SDI
Female
Male
0%
10%
20%
30%
40%
50%
60%
70%
80%
90%
100%
Neo
DM
CVD
CRD
