# Supplementary material for: Global burden and future projections of non-communicable diseases (2000–2050): Progress toward SDG 3.4 and disparities across regions and risk factors
Source: PLoS One. 2025 Dec 10;20(12):e0336036. doi: 10.1371/journal.pone.0336036 (PMC12694828; doi:10.1371/journal.pone.0336036)
Supplement: Attachment — Submitted filename: Response to Reviewer 2.docx [file pone.0336036.s003.docx]

Dear reviewer:

Thank you very much for your time and efforts for reviewing our paper, we appreciate your contribution to enriching our paper. As to address the important point you have raised, we kindly send the response point by point with track changes in the attached document.

1. “One of the major issues when using GBD data is addressing the technical pitfalls related to the identification of original information to construct mortality and other outcomes. The manuscript makes no mention to this issue (other than briefly outlining one of the limitations in the use of SDIs) and I would recommend that this gap is carefully addressed in the discussion.”

Reply: We thank the reviewer for this important observation. We have revised the discussion to explicitly acknowledge the technical pitfalls of GBD data, particularly the difficulty in identifying and validating the original source information used to construct mortality and outcome estimates. Text added to the discussion (last two paragraphs before the conclusion, page 32 track changes document): “A key methodological limitation of GBD data is the difficulty in tracing the original source information used to construct mortality and outcome estimates. Because GBD integrates diverse datasets through complex modeling, the transparency and reproducibility of the underlying inputs remain constrained. This limits independent validation and may obscure the extent to which local data are represented, an issue that should be considered when interpreting GBD-derived outcomes.”

2. Abstract –
   Background, please edit first line to clarify that NCDs, [including] CVD etc are the leading cause of death. It might be argued that NCDs are not the leading global health challenge

Modifications: we acknowledge the importance of this observation, we have modified the text to the following:

“Non-communicable diseases (NCDs), such as cardiovascular diseases (CVDs), cancers, chronic respiratory diseases (CRDs), and diabetes mellitus (DM), are the leading cause of mortality worldwide and represent a major barrier to achieving Sustainable Development Goal (SDG) 3.4, which targets a one-third reduction in premature mortality by 2030. This study quantifies the current and projected burden of these NCDs, ealuates disparities, and explores prevention opportunities..”

3. Introduction –
   Well-structured and places the topic into context.
   Page 9, paragraph 1 - I suggest editing the first paragraph to clarify that NCDs disproportionally affect the most vulnerable socio-economic communities (there appears to be a disconnection between sentences linking the socio-economic contexts [not driven by urbanization or ageing populations)]
   Page 9, paragraph 3 - The Intro states that projections of estimates extend to 2025, while the abstract describe them as of 2050 – other sections of the manuscript (e.g., Methods) also refer to either, please review for consistency.

Modified text:

Non-communicable diseases (NCDs), such as cardiovascular diseases, cancers, chronic respiratory conditions, and diabetes, are the predominant cause of death globally, accounting for approximately 74% of all mortality, as reported by the World Health Organization (1). This escalating burden, intensifying over recent decades, places significant pressure on health systems worldwide, particularly in low- and middle-income countries (LMICs), where limited resources and infrastructure exacerbate challenges (2). NCDs disproportionately affect the most vulnerable socioeconomic communities, including those in LMICs, due to factors such as poverty, limited healthcare access, and social inequities, rather than solely rapid urbanization or aging populations (3). In LMICs, this reflects an epidemiological transition from infectious diseases to chronic conditions, compounding existing health challenges (4,5).

Page 9, paragraph 3 - The Intro states that projections of estimates extend to 2025, while the abstract describe them as of 2050 – other sections of the manuscript (e.g., Methods) also refer to either, please review for consistency.

Modifications: 2025 modified to 2050

4. Methods –
   A description is required of what was included as NCDs to quantify incidence, mortality and DALYs – please provide details with levels of NCD classification i.e., level 1, 2 and 3, and include the information in a supplementary table

Modifications: classification table was added to supplementary data, the modified text below replaced the original

“We conducted a secondary analysis of the global burden of non-communicable diseases (NCDs), specifically cardiovascular diseases (CVD), neoplasms, diabetes mellitus (DM), and chronic respiratory diseases (CRDs), from 2000 to 2021, with projections to 2050. These NCDs were classified using the Global Burden of Disease (GBD) Study 2019 framework, which organizes diseases into three levels: Level 1 includes all NCDs as a broad group, and Level 2 includes major categories like CVD, neoplasms, DM, and CRDs. Our study focused on these Level 2 categories without analyzing specific Level 3 subcategories (e.g., ischemic heart disease or lung cancer). Supplementary Table S1 lists the NCDs included in our analysis and their GBD classification levels. Data were sourced from the GBD 2019, published by the Institute for Health Metrics and Evaluation (IHME) (9), covering 204 countries and territories. This comprehensive country selection aligns with the GBD 2019’s standardized framework, which uses consistent data collection and reporting methods across nations to ensure comparability with global health metrics standards. The GBD 2019 provides estimates of incidence, mortality, and disability-adjusted life years (DALYs) (9), a metric combining years of life lost and years lived with disability, derived from integrated health data, along with risk factor contributions and 2050 projections based on historical trends.”

Page 10, paragraph 1 – Unclear why selecting 204 countries ‘ensure methodological consistency with international standard’ (please clarify); same paragraph, please add reference for DALYs

We selected 204 countries because this classification aligns with the Global Burden of Disease (GBD) study framework, which is widely used in global health research and adopted by major international agencies (e.g., WHO, UN). Using the same country set ensures comparability and methodological consistency with internationally recognized standards, enabling our results to be directly compared with other global and regional analyses. This approach avoids selective inclusion or exclusion of countries that could bias burden estimates and maintains transparency and reproducibility of our methodology. Reference for DALYS was added.

5. Page 10, paragraph 2 – when first introducing standardized rates, please state 100,000 ‘population per year, or 100,000 ‘’people per year. For subsequent mentions in the manuscript, 100,000 would suffice’. Please add reference to cite DALYs and SDI

Modified text (methods section):

We reported age-standardized incidence rates (new cases per 100,000 population per year), mortality rates (deaths per 100,000 population per year), and disability-adjusted life years (DALYs; per 100,000 population per year), which combine years of life lost and years lived with disability, for cardiovascular diseases (CVD), neoplasms, diabetes mellitus (DM), and chronic respiratory diseases (CRDs). Outcomes were evaluated globally, by Socio-Demographic Index (SDI; Low, Low-Middle, Middle, High-Middle, High), WHO region (African, Americas, Eastern Mediterranean, European, South-East Asia, Western Pacific), sex, and country, with highest and lowest rates highlighted where applicable.

6. Results –
   The majority of results on mortality/incidence and DALYs reported for 2021 replicate those by Li et al (Int J Surgery, May 2025) – I would suggest citing them and further differentiating the reporting of findings; also please indicate in the Discussion how the two studies are complementary (where not overlapping)

Reply:

We thank the reviewer for this important suggestion. We have cited Li et al. (Int J Surgery, 2025) in the Results and added a paragraph in the Discussion to clarify how the two studies complement each other. We also note that our manuscript was completed and first submitted in March 2025, prior to the publication of Li et al., which reinforces the independent development of our work.

***Modifications:***

results part: “Our estimates for mortality, incidence, and DALYs in 2021 are broadly consistent with those reported by Li et al. (Int J Surgery, 2025), reflecting the robustness of Global Burden of Disease (GBD)-based projections.”

Discussion part: “While Li et al. (Int J Surgery, 2025) recently reported comparable estimates of 2021 NCD mortality, incidence, and DALYs, our study was conceived, completed, and initially submitted prior to its publication (March 2025). Importantly, our analysis extends beyond replication by providing long-term projections to 2050, examining disparities by region and income level, and incorporating a broader assessment of policy-relevant risk factors. Taken together, the two studies are complementary: Li et al. reinforce the reliability of near-term GBD estimates, while our work expands the horizon of understanding by focusing on long-term trajectories and prevention opportunities.”

7. Page 11, paragraph 1 – it is stated that the global incidence of NCDs was 12.4 billion new cases, and the four most common types CVD (66.8 million), cancer (66.5 million), chronic respiratory disease 55.2 million, and DM (24.4 million) represent less than 3% of all global NCD incidence. It would be helpful to report this, and to indicate what the leading incident NCDs were. Is the incidence rate correct (156,680 per 100,000 population)?

reply: we have further explained the numbers in the text, the modifications contain the leading incidence in 2021 and a breakdown for all contributed NCD’s. the incidence rate 156.680 per 100,000 is correct and additional supplementary contains the total for (incidence, mortality and DALYS) was included with a breakdown for the four major (included in our study) and other NCD’s.

8. Figure 1 – it shows incidence trends for the period 200-2021, however, the figures for the incidence in 2021 does not appear to match what is described in paragraph 1 of Results. In figure 1, the incidence of CVD for 2021 is ~850 (million?) with similarly high numbers reported for the other three causes. Please clarify.

Reply: We thank the reviewer for highlighting this source of confusion. The values shown in Figure 1 are age-standardized incidence rates per 100,000 population, whereas paragraph 1 of the Results reports the absolute number of new cases. For example, in 2021 the incidence of cardiovascular diseases was 66.8 million new cases worldwide, corresponding to an age-standardized incidence rate of 846.6 per 100,000. Figure caption was modified.

9. Figure 1 – it needs to indicate the unit of the incidence trends (e.g., age-standardised rates per …, or just raw numbers? ) if the info is incidence per 100,000 population, the numbers do not align with what is described in the first paragraph of results for incidence

Reply: the image caption was modified as the numbers represent the age-standardised rates

Reply: the section was modified as well as the figure and the caption.

10. Figures 2A and 2B – please include the year for which the estimates are provided, I assume it is 2021 ?

Reply: yes, 2021, the image caption was modified.

11. Page 12, section 3.3. – I would suggest reordering this paragraph to make the summary of findings easier to read (please note some punctuation is also missing. Rather than repeating all the info provided in Figure 2A, I suggest complementing it in the text, and summarizing in which SDI regions incidence, mortality, and DALYs for each of the four NCDs were highest and lowest

Reply: thank you very much, the results section was fully organised and arranged.

12. Page 14, country-based analysis – please indicate in this paragraph a brief summary of the types of NCDs included and mention that estimates are illustrated in Figure 3 (rather than repeating ‘figure 3’ in each sub-section of NCDs)

Reply: Thank you for the suggestion. We revised Section 3.4 to introduce the four NCDs at the start, indicate once that estimates are shown in Figure 3, and removed repeated figure mentions in each subsection. Punctuation was corrected and the text streamlined, with detailed values retained in Supplementary Tables 2A–2D.

13. Pages 15 and 23, I think the authors mean diabetes mellitus, not diabetes maleates ? The abstract, Methods and Intro sections describe it as the former.

Reply: yes, it was a typo error

14. Page 18, Figure 4 – please insert the year (2021) for which the reporting of metabolic and other risk factors is reported is presented in this figure (same applies for figure 5)

Reply: the figures were modified and the captions were updated

15. Discussion – the main findings are summarized and the impact of the magnitude of NCD burden is outlined. However, no discussion is given to the type of data used to determine NCD incidence or mortality, and this is a critical aspect when interpreting data from the GBD consortium. This should be described and discussed (in addition to being stated as a limitation of the study in the corresponding section, which is also not mentioned)

Reply: Thank you for the comment. We have now added a description in the Discussion outlining the data sources used by the GBD consortium (vital registration, registries, surveys, and modelling) and highlighted their limitations in data quality and coverage, particularly in low-resource settings. This has also been incorporated into the Limitations section.
